# Studies on the Regioselective Rearrangement of Azanorbornanic Aminyl Radicals into 2,8-Diazabicyclo[3.2.1]oct-2-ene Systems

**DOI:** 10.1021/acs.joc.2c02201

**Published:** 2022-12-01

**Authors:** Enrique Gil de Montes, Matteo A. Tallarida, Ana T. Carmona, Claudio D. Navo, Inmaculada Robina, Pilar Elías-Rodríguez, Gonzalo Jiménez-Osés, Antonio J. Moreno-Vargas

**Affiliations:** † Departamento de Química Orgánica (Facultad de Química), Universidad de Sevilla, C/ Prof. García González, 1, 41012Sevilla, Spain; ‡ Center for Cooperative Research in Biosciences (CIC bioGUNE), Basque Research and Technology Alliance (BRTA), Bizkaia Technology Park, Building 800, 48160Derio, Spain; § Department of Chemistry and Chemical Technologies, University of Calabria, Via P. Bucci, Cubo 12C, 87036Rende, Italy; ∥ Ikerbasque, Basque Foundation for Science, 48013Bilbao, Spain

## Abstract

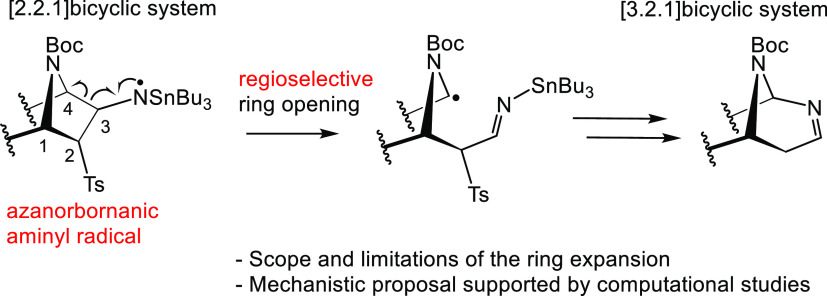

Aminyl radicals are nitrogen-centered radicals of interest in synthetic strategies involving C–N bond formation due to their high reactivity. These intermediate radicals are generated by the reaction of an organic azide with tributyltin hydride (Bu_3_SnH) in the presence of substoichiometric amounts of azobisisobutyronitrile (AIBN). In this work, we report the regioselective rearrangement of azanorbornanic ([2.2.1]azabicyclic) aminyl radicals into 2,8-diazabicyclo[3.2.1]oct-2-ene systems. With the aim to establish the structural requirements for this ring expansion, we have studied the effect of different bridgehead atoms of the [2.2.1]bicyclic system and the presence of an alkyl substituent at C4. Attempts to perform this ring expansion on a monocyclic analogue have been also explored to evaluate the influence of the bicyclic skeleton on the rearrangement. A detailed mechanistic proposal supported by computational studies is reported.

## Introduction

**Scheme 1:**
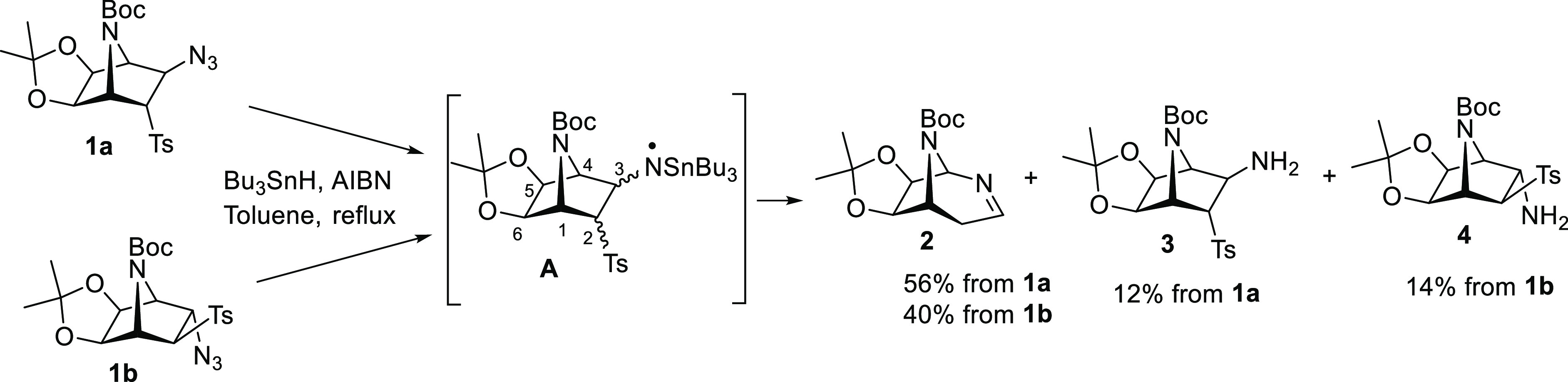
Ring Expansion of [2.2.1]Azabicyclic β-Azido Sulfones *via* Aminyl Radicals

Nitrogen-containing organic structures are commonly present in natural and non-natural biologically relevant compounds.^1^ Thus, the development of novel synthetic methods and strategies toward C–N bond formation is a very active field of research. Aminyl radicals are nitrogen-centered radicals that can be obtained from organic azides and are considered versatile intermediates for C–N bond formation due to their high reactivity.^2^ However, compared to broadly used carbon-centered radicals, aminyl radicals have received much less attention. Kim and co-workers demonstrated that tributyltin hydride (Bu_3_SnH) and substoichiometric amounts of azobisisobutyronitrile (AIBN) in refluxing benzene are excellent reagents for the generation of aminyl radicals through the homolytic addition of stannyl radicals to aromatic/aliphatic azides with simultaneous loss of N_2_.^3^ We have previously reported the unexpected formation of ring-expanded bicyclic system **2** as the major product when attempting the desulfonylation of [2.2.1]azabicyclic β-azido sulfones (3-azidoazanorbornanes) **1a** and **1b** under radical conditions (Scheme 1).^4^ The formation of this unexpected and unknown 2,8-diazabicyclo[3.2.1]oct-2-ene system was postulated to take place through the azanorbornan-3-aminyl radical intermediate **A**. Together with major compound **2**, the products from the radical reduction of the azide group, primary amines **3** and **4**, were also obtained as minor compounds. This competing radical reduction of aliphatic azides to amines under Bu_3_SnH/AIBN conditions is also known to proceed through an aminyl radical intermediate that is further reduced in the presence of an excess of Bu_3_SnH.^5^ Both stereoisomers, **1a** and **1b**, reacted similarly under these conditions, although 3-*exo*-azido **1a** was slightly more prone to undergo radical expansion than **1b** (56% from **1a** vs 40% from **1b**).

**Scheme 2:**
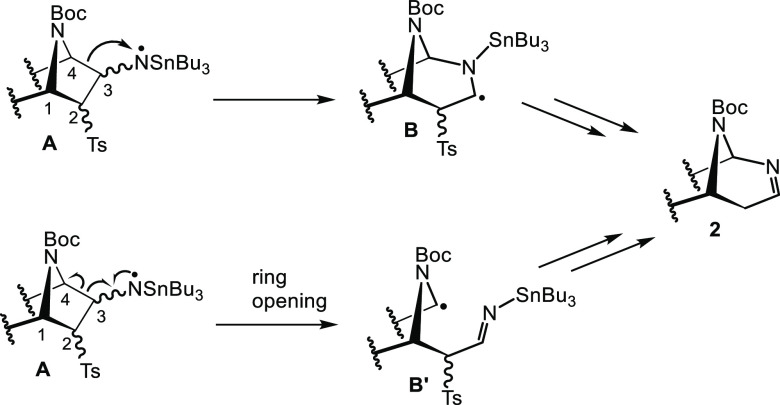
Proposed Radical Intermediate Structures **B** and **B′**

We initially proposed a regiospecific 1,2-shift of the σ (C3–C4) bond of bicyclic aminyl radical **A** to form the expanded radical intermediate **B**, which evolves to the final compound **2** (Scheme 2).^4^ However, as radical 1,2-shifts of alkyl carbons were virtually unknown, Spagnolo and co-workers proposed that a ring-opening/ring-closure sequence through intermediate **B′** should be more likely involved.^2b^ Nevertheless, a detailed mechanistic proposal has not been reported to date.

This radical ring expansion could constitute the key step of a straightforward procedure for the preparation of 2,8-diheterobicyclo[3.2.1]octanes from easily available heteronorbornane systems as starting materials. 2,8-Diheterobicyclo[3.2.1]octanes are present in the skeleton of a wide diversity of biologically active natural products and constitute interesting building blocks in organic synthesis.^6^ The development of synthetic strategies leading to heterofunctionalized bridged bicyclo[3.2.1]octanes justifies our interest in the study of this radical reaction. Therefore, this full account continues our previous publication^4^ and includes the study of the scope, limitations, and mechanism of this transformation. The effect of different bridgehead groups and the presence of an alkyl substituent at C4 of different bicyclic systems have been studied with the aim to establish the structural requirements for this singular ring expansion. Finally, the radical reaction on a monocyclic counterpart was also explored to evaluate the influence of the bicyclic skeleton on the rearrangement. A detailed mechanistic proposal supported by computational studies is also reported.

## Results and Discussion

### Experimental Studies

**Scheme 3:**
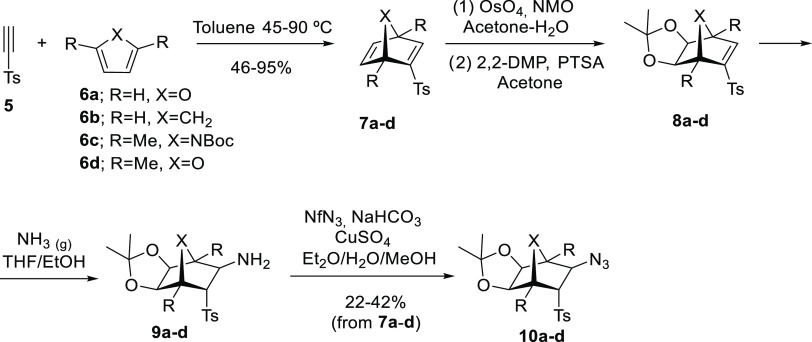
Synthesis of New [2.2.1]Bicyclic β-Azido Sulfones

We have carried out the synthesis of a variety of 3-azido(hetero)norbornane analogues of 3-*exo*-azidoazanorbornane **1a** (compounds **10a**–**d**) using a shorter and more efficient synthetic strategy than the one used previously.^7^ Moreover, this new strategy affords exclusively the *exo*-azido derivatives which are slightly more prone to the aminyl radical rearrangement than the corresponding *endo*-analogues. Thus, Diels–Alder reactions between ethynyl sulfone **5** and commercially available or easily prepared cyclic dienes **6a**–**d** (see the Supporting Information for details) were performed at different temperatures (25–90 °C). Reaction of **5** with pyrrole derivative **6c** was performed at higher temperatures than those with furan derivatives (**6a** and **6d**) and cyclopentadiene **6b** due to the poor dienic character of pyrrole. All the bicyclic systems (compounds **7a**–**d**) were isolated in moderate to good yield as racemic mixtures (Scheme 3). The selective dihydroxylation of the electron-rich double bond of bicyclic adducts **7a**–**d** afforded the corresponding diols with total *exo*-face stereoselectivity, as it was previously described for other [2.2.1]heterobicyclic systems.^8^ The resulting diols were not isolated, instead they were directly treated with 2,2-dimethoxypropane (DMP) under catalytic acid conditions to afford protected diol derivatives **8a**–**d**. Attempts to perform the addition of the azide anion to the vinyl sulfone system were unsuccessful (NaN_3_ in dimethylformamide, DMF, or TMSN_3_ in tetrahydrofuran, THF). Thus, we addressed the incorporation of the azide function in a two-step synthetic strategy. Conjugate addition of ammonia to vinyl sulfones **8a**–**d** showed to be efficient and stereoselective giving the corresponding [2.2.1]bicyclic β-amino sulfones **9a**–**d**. These compounds were directly used in a diazo transfer reaction with nonaflyl azide (NfN_3_) under mild conditions^9^ affording the desired [2.2.1]bicyclic β-azido sulfones **10a**–**d** in 22–42% overall yield (from **7a**–**d**, four steps).

With these substrates in hand, we studied the radical reaction in the presence of Bu_3_SnH/AIBN under the same reaction conditions previously used with **1a**. The results are summarized in Table 1. The substitutions of NBoc in **1a** by O or CH_2_ (compounds **10a** and **10b**) were detrimental for the ring expansion and only the non-expanded amino derivatives **9a** and **9b** were obtained. This result seems to indicate that unlike NBoc, O or CH_2_ at the bridgehead position of the bicyclic system do not stabilize the contiguous radical at C4 of the corresponding **B′** intermediate (Scheme 2). Heteroatoms containing lone electron pairs can establish stabilizing interactions with radical centers (two-center three-electron interaction), and the magnitude of this interaction is inversely proportional to the electronegativity of the heteroatom.^10^ This could explain the fact that oxanorbornane derivative **10a**, with a highly electronegative oxygen at the bridgehead position, and norbornane **10b**, with no heteroatom adjacent to the radical, do not undergo the radical rearrangement. The stabilizing effect that the NBoc group confers to the contiguous radical can explain the chemoselectivity observed. On the other hand, the radical reaction on azabicycle **10c**, which features an additional methyl group at C4 with respect to azabicycle **1a**, clearly favored the radical ring expansion with a remarkable increase in the yield of the resulting 2,8-diazabicyclo[3.2.1]oct-2-ene (82% **11c** vs 56% **2**). A CH_3_ substituent at C4 of **10c** clearly benefits the radical rearrangement as the new C4 becomes a tertiary radical on the corresponding intermediate **B′**. However, this extra stabilization was not enough in the case of the oxa-analogue **10d**, where the radical rearrangement did not take place and only amine **9d** was obtained. Of note, the desulfonylation reaction only took place during the formation of expanded [3.2.1]bicyclic systems (compounds **2** and **11c**) but not in the reduced [2.2.1]bicyclic amines (**3**, **9a**, **9b**, and **9d**). As the order of addition of reagents could influence the outcome of the radical reaction,^11^ treatment of [2.2.1]bicyclic azides **1a** and **10b** with Bu_3_SnH/AIBN was additionally performed following an alternative experimental procedure (procedure b, see the Experimental Section). However, the inverse order of addition of the reagents did not influence the final result, compounds **2** and **9b** being isolated in similar yields. The structure of ring-expanded compound **11c** could be confirmed by the appearance of an unambiguous signal at 7.66 ppm in the ^1^H NMR spectrum corresponding to imine-type proton H3. Besides, the signals at 162.3 (C3) and 39.3 ppm (C4, coupled to two adjacent protons in DEPT) were observed in the ^13^C NMR spectrum.

**Table 1:**
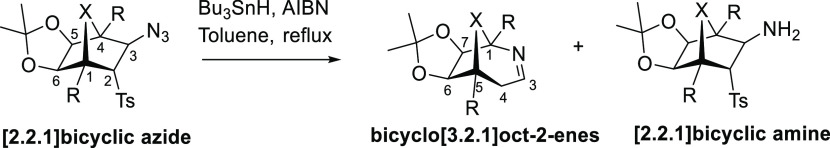
Results of the Radical Ring-Expansion Assays

entry	[2.2.1]bicyclic azide	bicyclo[3.2.1]oct-2-ene^a^	[2.2.1]bicyclic amine^a^
1^b^	**1a** (R = H, X = NBoc)	**2**, 56%	**3**, 12%
2	**10a** (R = H, X = O)	c	**9a**, 90%
3	**10b** (R = H, X = CH_2_)	c	**9b**, 74%
4	**10c** (R = Me, X = NBoc)	**11c**, 82%	c
5	**10d** (R = Me, X = O)	c	**9d**, 64%

a Yield (%) of isolated compound following experimental procedure a (see the Experimental Section).

b Data from ref (4).

c Not detected.

**Scheme 4:**
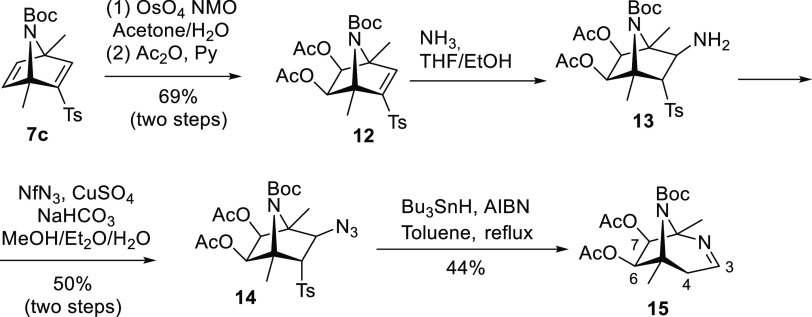
Synthesis and Radical Ring-Expansion of Less-Strained 3-Azidoazanorbornane **14**

To clarify the influence of the dioxolane fused cycle on the radical expansion of azabicyclic derivatives **1** and **10c**, we assayed the reaction on diacetylated derivative **14** (Scheme 4). After dihydroxylation of **7c**, the resulting diol was acetylated to afford **12**. Subsequent conjugate addition of ammonia followed by diazo transfer reaction gave azide **14** in an acceptable yield. Treatment of **14** with Bu_3_SnH/AIBN under standard conditions afforded a mixture of compounds where the expanded 2,8-diazabicyclo[3.2.1]oct-2-ene **15** could be isolated in 44% yield. This result indicates that less-strained 3-azidoazanorbornane **14** is less prone to radical ring expansion than acetonide derivative **10c**, which could be attributed to the extra strain conferred by the acetonide group to the azabicyclic system and the steric hindrance existing between the NBoc and the isopropylidene group.

**Scheme 5:**
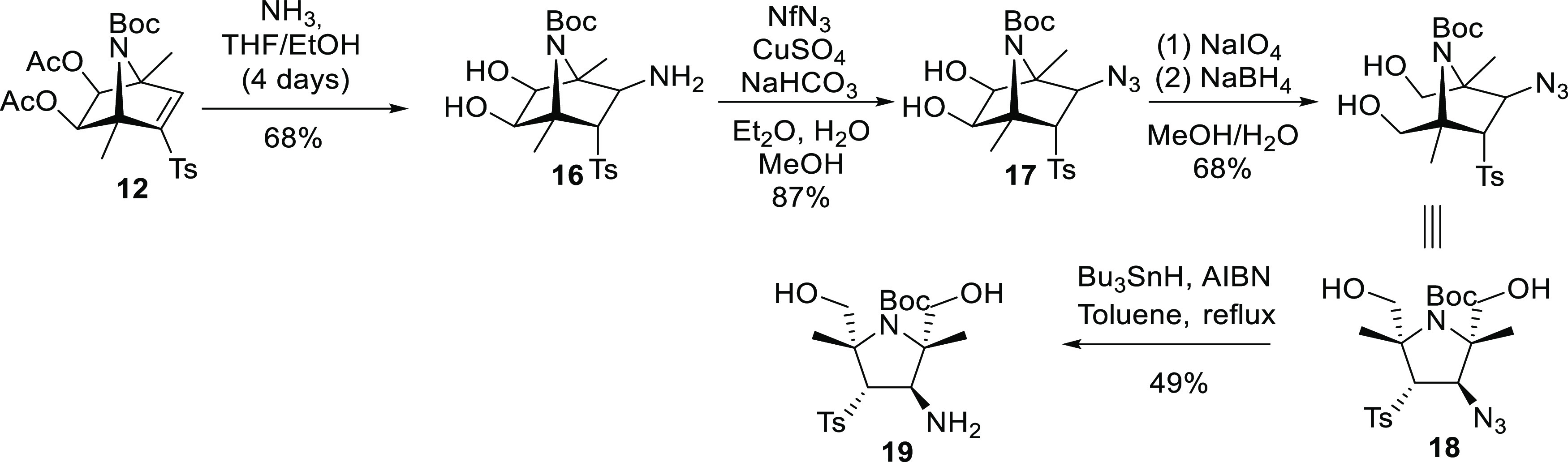
Attempt to Ring-open the 3-Azidopyrrolidine System **18**

Finally, we attempted the radical expansion on pyrrolidine derivative **18** (Scheme 5), a monocyclic analogue of bicyclic compound **14**, to fully suppress the influence of the bicyclic skeleton in the reaction. Thus, treatment of **12** with an excess of ammonia in THF/EtOH for 4 days yielded the conjugate addition of NH_3_ with concomitant ethanolysis of the ester groups. The resulting bicyclic aminodiol **16** was transformed into azido derivative **17** under standard conditions. Oxidative cleavage of diol **17** with NaIO_4_, followed by reduction of the resulting aldehydes with NaBH_4_, afforded *N*-Boc pyrrolidine derivative **18** in good yield. Treatment of this compound with Bu_3_SnH/AIBN afforded a complex mixture where no product from a ring radical expansion could be detected. Instead, amine **19** resulting from the radical reduction of the azide function was the major product and could be isolated in 49% yield. This result indicates that the rigid [2.2.1]azabicyclic skeleton clearly favors the C3–C4 bond cleavage on the corresponding aminyl radical intermediate **A** (Scheme 2) while the radical reduction of the azide function is the only reaction observed for azides embedded in a more conformationally flexible pyrrolidine skeleton (compound **18**).

**Scheme 6:**
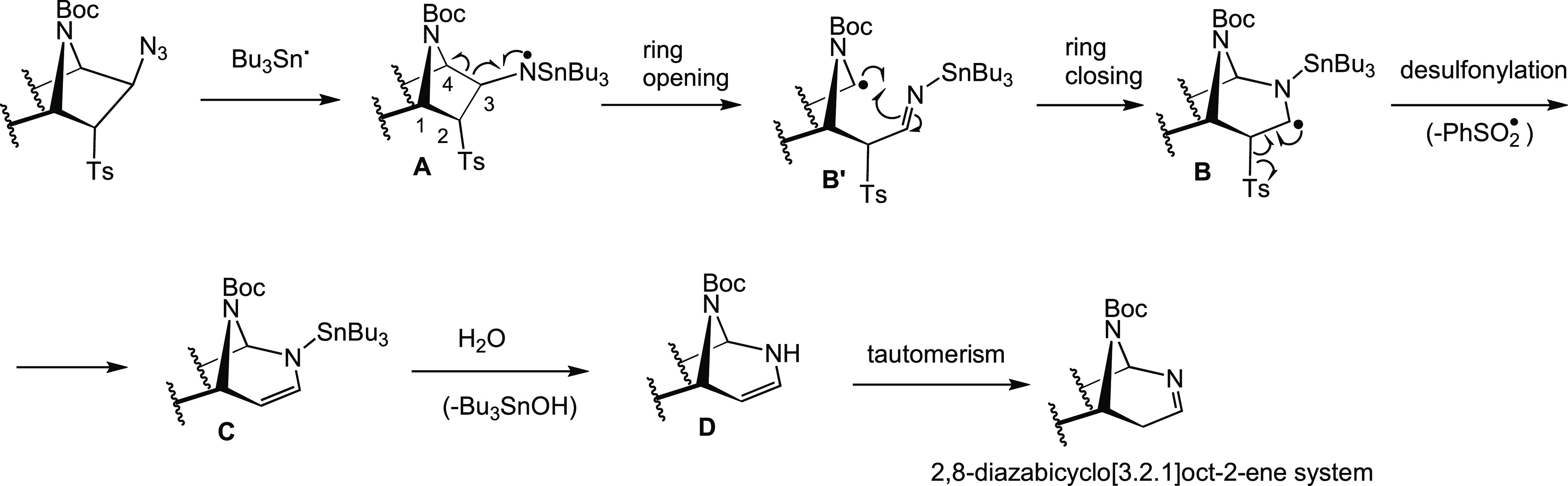
Mechanism Proposed for the Radical Ring Expansion/Desulfonylation of [2.2.1]Azabicyclic β-Azido Sulfones

Based on the experimental results, we propose a mechanism for the ring-expansion/desulfonylation sequence leading to 2,8-diazabicyclo[3.2.1]oct-2-enes (Scheme 6). Aminyl radical intermediate **A** would undergo a regioselective ring opening leading to carbon-centered radical **B′** which, after ring closing, would yield the expanded system **B**. A desulfonylation reaction, similar to that reported for allylic sulfones under Bu_3_SnH/AIBN conditions,^12^ would afford stannyl enamine **C** that after hydrolysis and tautomerization would originate the 2,8-diazabicyclo[3.2.1]oct-2-ene.

### Computational Analysis

**Figure 1:**
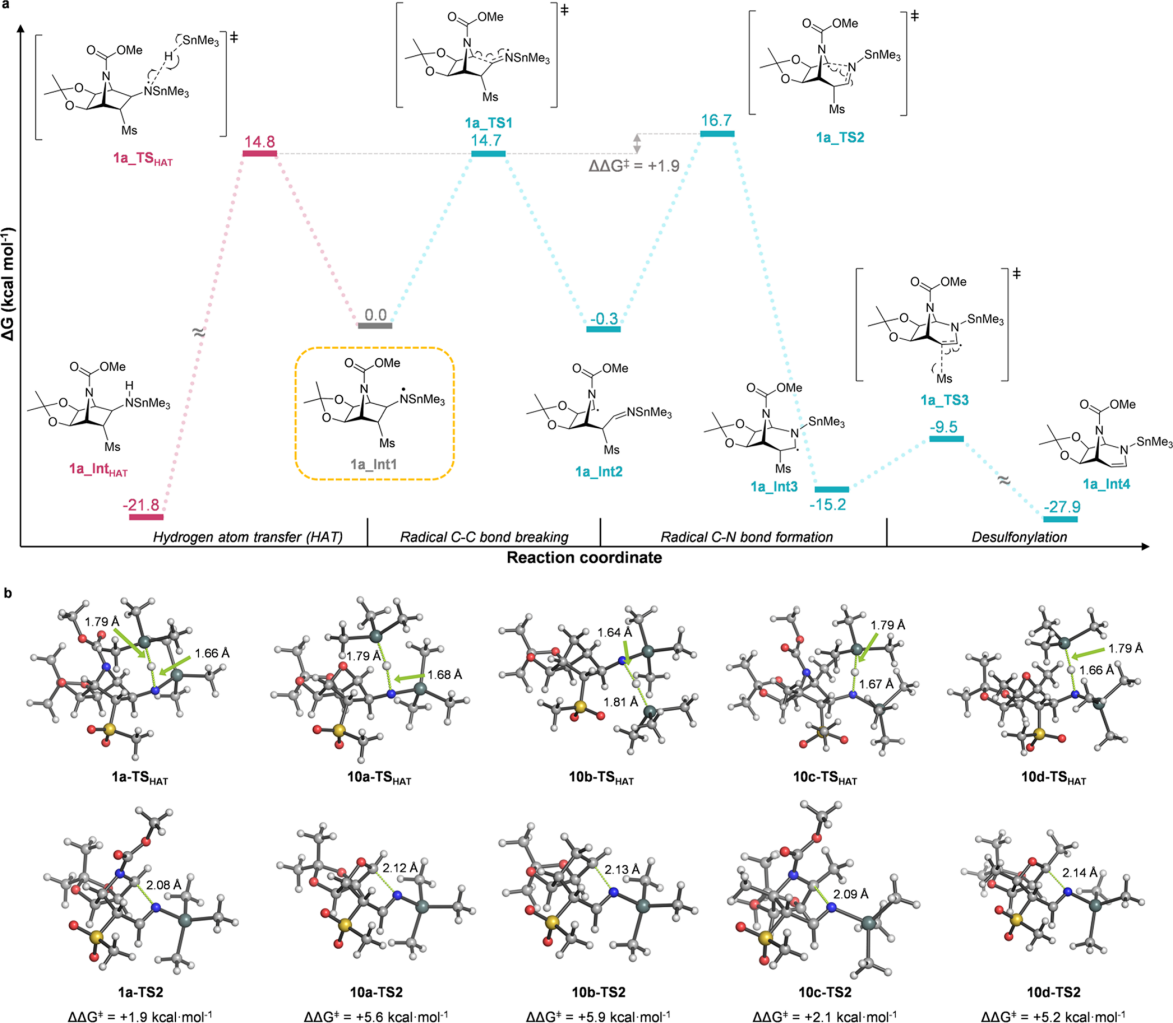
(a) Minimum energy reaction pathway for the model of azanorbornane derivative **1a** calculated with PCM(toluene)/M06-2X/6-31G(d,p)+LanL2DZ(Sn). (b) Transition structures (TSs) and relative activation energies for the competing HAT and ring-expansion reactions (ΔΔ*G*^‡^_TS2–TS_HAT__) for all calculated models of bicycles **1a** and **10a**–**d**.

The mechanism of the ring expansion for the formation of [3.2.1]bicyclic systems (**2**, **11a**–**d**) was examined computationally (see the Computational Analysis details). The calculated mechanism starts from norbornan-3-aminyl radical intermediates (**Int1**) and analyzes a plausible competition between a stepwise ring expansion, to afford compounds **2** and **11a**–**d**, and a radical reduction, i. e., hydrogen atom transfer (HAT),^13^ to give amines **3** and **9a**–**d** (Figures 1a and S1–S4). Abbreviated models were used in all cases by replacing the tosyl and tri-*n*-butylstannane groups by mesyl and trimethylstannane groups, respectively. Methoxycarbamate (Moc) was also employed instead of the Boc group as a simpler model for the protecting group of azanorbornanes **1a** and **10c**. Starting from *N*-centered norbornan-3-aminyl radical intermediates (**Int1**) formed upon reaction with Bu_3_SnH/AIBN, the first step of the ring-expansion reaction is the ring opening by a homolytic cleavage of the C3–C4 bond (**TS1**, see Table 1 for atom labeling) with activation barriers (Δ*G*^‡^) ranging from 12 to 19 kcal mol^–1^. The subsequent ring-closing step takes place from the carbon-centered radical (**Int2**) to form a bond between C3 and the exocyclic nitrogen (**TS2**). This step was calculated to be rate-limiting for all considered substrates with activation energies (Δ*G*^‡^) ranging from 17 to 22 kcal mol^–1^. Of note, formation of bicyclic carbon-centered radical **Int3** upon ring expansion is significantly exergonic with free energies (Δ*G*) between −12 and −15 kcal mol^–1^. A final desulfonylation step through a radical elimination reaction from **Int3** was calculated to have very low activation barriers (Δ*G*^‡^ ≈ 2–5 kcal mol^–1^) as a result of the high stability of the leaving sulfonyl radical.

On the other hand, the competing HAT reactions from a trimethylstannane hydride molecule to radical intermediates **Int1** through transition states **TS**_**HAT**_, leading to reduced intermediates (**Int**_**HAT**_), have similar activation barriers (Δ*G*^‡^ ≈ 14–16 kcal mol^–1^) to those calculated for the ring-expansion process, although different trends were obtained (Figure 1b). Norbornane (**10b**) and oxonorbornane (**10a** and **10d**) derivatives showed a preference for the HAT reaction as judged by the difference in the energies of the transition structures for both competing pathways (ΔΔ*G*^‡^_TS2–TS_HAT__ ≈ 5–6 kcal mol^–1^), in line with the experimental observations. However, these differences in energies were minimal for azanorbornane derivatives **1a** and **10c** (ΔΔ*G*^‡^_TS__2–TS_HAT__ ≈ 2 kcal mol^–1^), suggesting that both reactions are energetically feasible and, therefore, competitive. The possibility of C-centered radicals **Int2** undergoing a HAT reaction—which was never observed experimentally—was discarded considering the relatively high activation energy calculated for this process for compound **1a** (Δ*G*^‡^ ≈ 20 kcal mol^–1^, see the Supporting Information), due to the large steric hindrance exerted by the trialkyl tin hydride.

These computed trends can be attributed to the relative stability of the *N*- and *C*-centered radicals (**Int1** and **Int2**, respectively) for each substrate. The *C*-centered radicals are thermoneutral with respect to the *N*-centered radicals for azanorbornane compounds (**1a** and **10c**), while being significantly less stable for oxanorbornene and norbornane. The *C*-centered radical **Int2** for norbornane **10b** is unstable due to the lack of an adjacent heteroatom with lone pairs at the bridgehead position, whereas the same *C*-centered radicals for azanorbornanes **1a** and **10c** are stabilized by delocalization of the spin density along the adjacent carbamate group (Figure S5). Consequently, and according to Hammond’s postulate, the activation barriers for the ring-closing transition state (**TS2**) for oxanorbornanes **10a** and **10d** and, more significantly, for norbornane **10b** are higher than those for the competitive **TS**_**HAT**_, thus exhibiting a preference for the HAT reaction (Figures S1, S2, and S4). In contrast, azanorbornanes **1a** and **10c** display lower activation energies for the ring expansion, which becomes competitive with HAT. The homolytic cleavage of the C2–C3 bond on **Int1** leads to a transition state ca. 4 kcal mol^–1^ higher in energy than that calculated for the homolytic cleavage of the C3–C4 bond, which explains the exceptional regioselectivity observed experimentally in the ring expansion.

## Conclusions

We have demonstrated that 3-azidoazanorbornanes are excellent substrates for the preparation of 2,3-diazabicyclo[3.2.1]oct-2-enes through a regioselective rearrangement of azanorbornanic aminyl radicals. These systems were previously unknown and can be considered as precursors of the 2,8-diheterobicyclo[3.2.1]octane skeleton, which is present in natural products. Experimental and computational studies allowed us to establish the scope of the reaction and provide a mechanistic proposal to this unusual radical rearrangement. The scope of this reaction is limited to the aza-analogues where the intermediate radicals are adequately stabilized. Moreover, the rigidity of the azabicyclic skeleton showed to be crucial for the rearrangement to proceed efficiently. With the discovery of this new radical rearrangement, new synthetic strategies involving compounds containing a rigid azido-functionalized azacyclic core could be explored.

## Experimental Section

### General Methods

^1^H and ^13^C NMR spectra were recorded with a Bruker AMX300 spectrometer for solutions in CDCl_3_ and DMSO-*d*_6_. δ is given in ppm and *J* in Hz. All of the assignments were confirmed by two-dimensional (2D) spectra (COSY and HSCQ). High-resolution mass spectra were recorded on a Q Exactive quadrupole mass spectrometer. Thin layer chromatography (TLC) was performed on silica gel 60 F_254_ (Merck), with detection by UV light charring with *p*-anisaldehyde, KMnO_4_, ninhydrin, phosphomolybdic acid, or with reagent [(NH_4_)_6_MoO_4_, Ce(SO_4_)_2_, H_2_SO_4_, H_2_O]. Silica gel 60 (Merck, 40–60 and 63–200 μm) was used for preparative chromatography. Infrared spectra were recorded with a Jasco FTIR-410 spectrophotometer and processed with Jasco Spectra Manager program, using solid and oily compounds in an ATR MIRacle. Maximum absorption wave numbers are indicated.

### Synthesis of [2.2.1]Bicyclic β-Azido Sulfones

Synthesis of (7-Hetero)norbornadienic β-Azido Sulfones **10a,b** from (7-Hetero)norbornadienes **7a,b**. To a solution of (7-hetero)norbornadiene (**7a** or **7b**) (2.2 mmol) in acetone (47 mL) and water (5.1 mL), NMO (3.2 mmol) and OsO_4_ (4 wt % in H_2_O, 0.079 mmol) were added. The mixture was stirred for 50 min, and then, a saturated solution of NaHSO_3_ in water was added at 0 °C. After a few minutes, the reaction mixture was diluted with EtOAc and washed with water. The organic layer was dried over Na_2_SO_4_ and filtered, and the solvent was removed under reduced pressure to give the dihydroxylated compound that was used without purification in the next step. To a solution of this compound in acetone (26 mL), 2,2-dimethoxypropane (13 mmol) and PTSA (0.2 mmol) were added. The reaction was stirred at room temperature (rt) for 2 h and then diluted with CH_2_Cl_2_ and washed with an aqueous saturated solution of NaHCO_3_. The organic layer was dried over Na_2_SO_4_ and filtered, and the solvent was removed under reduced pressure to give **8a** or **8b**. To a solution of **8a** or **8b** in EtOH (18 mL) and THF (7 mL) at 0 °C, NH_3_ was bubbled. The reaction mixture was allowed to warm at rt for 4 h, and then, the solvent was removed under reduced pressure to give the crude protected amine **9a** or **9b** that was directly dissolved in MeOH (8 mL) and water (3 mL). Then, NaHCO_3_ (800 mg, 9.5 mmol), a solution of nonaflyl azide (1.1 g, 3.2 mmol) in Et_2_O (5.7 mL), and CuSO_4_·5H_2_O (57 mg, 0.2 mmol) were added and the reaction mixture was stirred at rt for 3 h. The organic solvents were removed and the resultant aqueous mixture was extracted with dichloromethane (DCM) and washed with an aqueous saturated solution of NaHCO_3_. The organic layer was dried over Na_2_SO_4_ and filtered. The solvent was removed under reduced pressure, and the crude product was purified by column chromatography on silica gel (EtOAc/Cy 1:6). Compound **10a** was obtained as a white powder (280.6 mg, 24%, 4 steps); compound **10b** was obtained as a white foam (335.8 mg, 42%, 4 steps).

#### Data for **10a**

IR (υ̅, cm^–1^) 2975, 2087 (N_3_), 1305 1209, 1139, 669. ^1^H NMR (300 MHz, CDCl_3_) δ 7.80 (d, *J* = 8.4 Hz, 2H, Ar*H*), 7.42 (m, 2H, Ar*H*), 5.21 (d, *J* = 5.5 Hz, 1H, H4), 4.65 (dd, *J* = 5.5, 1.4 Hz, 1H, H1), 4.48–4.42 (m, 2H, H2, H3), 3.88 (d, *J* = 4.0 Hz, 1H, H5), 3.55–3.45 (m, 1H, H6), 2.47 (s, 3H, C*H_3_* of Ts), 1.45 (s, 3H, C*H_3_*), 1.33 (s, 3H, C*H_3_*). ^13^C {^1^H} NMR (76 MHz, CDCl_3_) δ 146.2 (*C*Ar), 136.1 (*C*Ar), 130.7, 128.0 (*C*HAr), 112.6 (*C*(CH_3_)_2_), 86.1, 79.7 (C2, C3), 79.6 (C4), 78.5 (C1), 70.6 (C6), 60.9 (C5), 25.8, 25.3 (*C*H_3_), 21.9 (*C*H_3_ of Ts). HRMS (ESI) *m*/*z*: [M + Na]^+^ calcd for C_16_H_19_O_5_N_3_NaS: 388.0938; found: 388.0930.

#### Data for **10b**

IR (υ̅, cm^–1^) 2978, 2917, 2104 (N_3_), 1595, 1277, 1143, 1041, 865, 663. ^1^H NMR (300 MHz, CDCl_3_) δ 7.79 (d, *J* = 8.3 Hz, 2H, Ar*H*), 7.45–7.34 (m, 2H, Ar*H*), 5.10–4.99 (m, 1H, H2 or H3), 4.31–4.19 (m, 1H, H2 or H3), 3.88 (dd, *J* = 4.2, 1.9 Hz, 1H, H5), 3.18 (t, *J* = 4.2 Hz, 1H, H6), 2.67 (dt, *J* = 4.2, 1.3 Hz, 1H, H1), 2.46 (s, 4H, C*H*_*3*_ of Ts, H4), 1.96 (dq, *J* = 11.2, 1.7 Hz, 1H, H7a), 1.43 (s, 3H, C*H*_*3*_), 1.38 (dd, *J* = 11.2, 1.6 Hz, 1H, H7b), 1.33 (s, 3H, C*H*_*3*_). ^13^C {^1^H} NMR (76 MHz, CDCl_3_) δ 145.6, 136.3 (*C*Ar), 130.4, 128.2 (*C*HAr), 110.1 (*C*(CH_3_)_2_), 78.7, 76.4 (C2, C3), 70.8 (C6), 60.2 (C5), 48.1 (C4), 43.3 (C1), 31.1 (C7), 25.3, 24.3 (*C*H_3_), 21.8 (CH_3_ of Ts). HRMS (ESI) *m*/*z*: [M + Na]^+^ calcd for C_17_H_21_O_4_N_3_NaS: 386.1145; found: 386.1139.

#### Synthesis of (7-Hetero)norbornadienic Vinyl Sulfones **8c,d** from (7-Hetero)norbornadienes **7c,d**

To a solution of **7c** or **7d** (0.8 mmol) in acetone (18 mL) and water (2 mL), NMO (1.4 mmol) and OsO_4_ (4 wt % in H_2_O, 0.004 mmol) were added. The mixture was stirred overnight, and then, a saturated aqueous solution of NaHSO_3_ was added at 0 °C. After a few minutes, the crude was diluted with EtOAc and washed with water. The organic layer was dried over Na_2_SO_4_ and filtered. The solvent was removed under reduced pressure. To the resulting residue dissolved in acetone (10 mL), 2,2-dimethoxypropane (5.5 mmol) and PTSA (0.1 mmol) were added. The reaction was stirred at rt overnight; then diluted with dichloromethane and washed with an aqueous saturated solution of NaHCO_3_. The organic layer was dried over Na_2_SO_4_ and filtered. The solvent was removed under reduced pressure and purified by column chromatography on silica gel (EtOAc/Cy 1:5). Compound **8c** was obtained as a white powder (241.0 mg, 67%, two steps); compound **8d** was obtained as a white powder (162.6 mg, 58%, two steps).

#### Data for **8c**

IR (υ̅ cm^–1^) 2979, 1699, 1596, 1450, 1362, 1266, 1056, 976, 868, 755. ^1^H NMR (300 MHz, CDCl_3_) δ 7.89–7.61 (m, 2H, Ar*H*), 7.40–7.30 (m, 2H, Ar*H*), 6.83 (s, 1H, H3), 4.29 (d, *J* = 5.5 Hz, 1H, H5 or H6), 4.12 (d, *J* = 5.5 Hz, 1H, H5 or H6), 2.46 (s, 3H, C*H*_*3*_ of Ts), 1.80 (s, 3H, C*H*_*3*_), 1.68 (s, 3H, C*H*_*3*_), 1.44 (s, 3H, C(C*H*_*3*_)_2_), 1.35 (s, 9H, C(C*H*_*3*_)_3_), 1.32 (s, 3H, C*H*_*3*_). ^13^C {^1^H} NMR (76 MHz, CDCl_3_) δ 153.8 (C=O), 152.7 (C3), 150.6 (C2), 145.2, 136.6 (*C*Ar), 130.1, 128.3 (*C*HAr), 117.1 (*C*(CH_3_)_2_), 83.2, 82.7 (C5, C6), 80.5 (*C*(CH_3_)_3_), 72.8, 71.9 (C1, C4), 28.5 (C(*C*H_3_)_3_), 26.5, 25.8 (*C*H_3_), 21.8 (*C*H_3_ of Ts), 15.3 (*C*H_3_), 13.4 (*C*H_3_). HRMS (ESI) *m*/*z*: [M + Na]^+^ calcd for C_23_H_31_NO_6_SNa: 472.1770; found: 472.1758.

#### Data for **8d**

IR (υ̅ cm^–1^) 2986, 2853, 1598, 1448, 1269, 1208, 1083, 970, 876. ^1^H NMR (300 MHz, CDCl_3_) δ 7.77 (d, *J* = 8.3 Hz, 2H, Ar*H*), 7.36 (m, 2H, Ar*H*), 6.84 (d, *J* = 0.5 Hz, 1H, H3), 4.43 (d, *J* = 5.3 Hz, 1H, H5 or H6), 4.30 (d, *J* = 5.2 Hz, 1H, H5 or H6), 2.46 (s, 3H, C*H*_*3*_ of Ts), 1.55 (s, 3H, CH_3_), 1.48 (s, 3H, C*H*_*3*_), 1.43 (s, 3H, CH_3_), 1.35 (s, 3H, C*H*_*3*_). ^13^C {^1^H} NMR (76 MHz, CDCl_3_) δ 153.1 (C2), 148.8 (C3), 145.3, 136.6 (*C*Ar), 130.2, 128.3 (*C*HAr), 117.7 (*C*(CH_3_)_2_), 88.7, 88.2 (C1, C4), 83.2, 82.7 (C5, C6), 26.6 (*C*H_3_), 21.8 (*C*H_3_ of Ts), 13.7(CH_3_), 12.5 (CH_3_). HRMS (ESI) *m*/*z*: [M + Na]^+^ calcd For C_18_H_22_O_5_SNa: 373.1086; found: 373.1074.

#### Synthesis of (7-Hetero)norbornadienic β-Azido Sulfones **10c,d** from (7-Hetero)norbornadienic Vinyl Sulfones **8c,d**

Compound **8c** or **8d** (1.3 mmol) was dissolved in ethanol (3 mL) and THF (6 mL) at 0 °C. Then, NH_3_ was bubbled for 5 min. The reaction was allowed to warm at rt for 4 h, and then, the solvent was removed under reduced pressure. The resulting amine **9c** or **9d** was dissolved in MeOH (5.5 mL) and H_2_O(2 mL); NaHCO_3_ (5.9 mmol), a solution of nonaflyl azide (2.96 mmol) in Et_2_O (4 mL), and CuSO_4_·5H_2_O (0.2 mmol) were added and the mixture was stirred at rt for 3 h. The organic solvents were removed, and the resulting aqueous mixture was extracted with dichloromethane and washed with an aqueous saturated solution of NaHCO_3_. The organic layer was dried over Na_2_SO_4_ and filtered. The solvent was removed under reduced pressure, and the reaction mixture was purified by chromatography column on silica gel (EtOAc/Cy 1:10 for **10c**, dichloromethane/Cy 1:10 for **10d**). Compound **10c** was obtained as a white foam (269.0 mg, 42%, two steps); compound **10d** was obtained as a white powder (189.2 mg, 37%, two steps).

#### Data for **10c**

IR (υ̅ cm^–1^) 2979, 2103 (N_3_), 1700, 1597, 1292, 1211, 1082, 849, 707. ^1^H NMR (300 MHz, CDCl_3_) δ 7.84–7.72 (m, 2H, Ar*H*), 7.45–7.36 (m, 2H, Ar*H*), 4.92 (d, *J* = 5.7 Hz, 1H, H5 or H6), 4.10 (d, *J* = 5.7 Hz, 1H, H5 or H6), 3.61 (d, *J* = 4.1 Hz, 1H, H2), 3.22 (d, *J* = 4.1 Hz, 1H, H3), 2.47 (s, 3H, C*H*_*3*_ of Ts), 1.85 (s, 3H, C*H*_*3*_), 1.64 (s, 3H, C*H*_*3*_), 1.42 (bs, 12H, C(C*H*_*3*_)_3_, C*H*_*3*_), 1.34 (s, 3H, C*H*_*3*_). ^13^C {^1^H} NMR (76 MHz, CDCl_3_) δ 154.3 (C=O), 146.0, 136.6 (*C*Ar), 130.5, 128.3 (*C*HAr), 112.0 (*C*(CH_3_)_2_), 82.3 (C5 or C6), 80.7 (*C*(CH_3_)_3_), 80.2 (C5 or C6), 74.5 (C3), 72.3, 70.8 (C1, C4), 66.9 (C2), 28.5 (C(*C*H_3_)_3_), 26.0 (*C*H_3_), 25.4 (*C*H_3_), 21.9 (*C*H_3_ of Ts), 17.3, 14.3 (*C*H_3_). HRMS (ESI) *m*/*z*: [M + Na]^+^ calcd for C_23_H_32_N_4_O_6_SNa: 515.1941; found: 515.1931.

#### Data for **10d**

IR (υ̅, cm^–1^) 2993, 2937, 2103 (N_3_), 1596, 1454, 1256, 1010, 777, 650. ^1^H NMR (300 MHz, CDCl_3_) δ 7.80 (d, *J* = 8.3 Hz, 2H, Ar*H*), 7.49–7.33 (m, 2H, Ar*H*), 5.07 (d, *J* = 5.5 Hz, 1H, H5 or H6), 4.28 (d, *J* = 5.5 Hz, 1H, H5 or H6), 3.78 (d, *J* = 4.2 Hz, 1H, H3), 3.21 (d, *J* = 4.3 Hz, 1H, H2), 2.47 (s, 3H, CH_3_ of Ts), 1.54 (s, 3H, C*H*_*3*_), 1.47 (s, 3H, C*H*_*3*_), 1.42 (s, 3H, C*H*_*3*_), 1.35 (s, 3H, C*H*_*3*_). ^13^C {^1^H} NMR (76 MHz, CDCl_3_) δ 146.0, 136.5 (*C*Ar), 130.6, 128.2 (*C*HAr), 112.9 (*C*(CH_3_)_2_), 89.0, 86.7 (C1, C4), 83.1, 81.0 (C5, C6), 76.0 (C2), 66.6 (C3), 26.0, 25.8 (*C*H_3_), 21.9 (*C*H_3_ of Ts), 16.1, 12.0 (*C*H_3_). HRMS (ESI) *m*/*z*: [M + Na]^+^ calcd for C_18_H_23_O_5_N_3_NaS: 416.1256; found: 416.1244.

### Aminyl Radical Ring-Expansion/Azide Radical Reduction

#### Procedure a

In a two-necked round-bottom flask, AIBN (0.026 mmol, 0.1 equiv) and Bu_3_SnH (0.52 mmol, 2 equiv) were dissolved in dry toluene (1.0 mL) under Ar atmosphere. The solution was heated at 110 °C in an oil bath for two min. Then, a solution of the corresponding bicyclic substrate (0.26 mmol, 1 equiv) in dry toluene was added (1.5 mL). The reaction was stirred at 110 °C for 3 h and then quenched with an aqueous solution of NaF (1M). The resultant mixture was extracted twice with AcOEt and washed with brine. The organic layer was dried over Na_2_SO_4_ and filtered, and the solvent was removed under reduced pressure. The resulting crude mixture was purified by column chromatography.

#### Procedure b

In a two-necked round-bottom flask, bicyclic substrate (0.23 mmol, 1 equiv) was dissolved in dry toluene (4.0 mL) under Ar atmosphere. The solution was heated at 110 °C in an oil bath and a solution of AIBN (0.12 equiv, 0.029 mmol) and Bu_3_SnH (1.25 equiv, 0.29 mmol) in toluene (1.6 mL) under Ar atmosphere was gradually added (0.4 mL each 30 min). The mixture was refluxed for a total time 2.5 h. Then, the solvent was evaporated and the resulting crude mixture was purified by column chromatography

##### (*rac*)-*N*-Boc-6,7-*exo*-isopropylidendioxy-2,8-diazabicyclo[3.2.1]oct-2-ene (**2**)

The synthesis of **2** was carried out from **1a** (108 mg, 0.23 mmol) according to the general procedure b described above. Compound **2** (33 mg, 51%) was obtained after purification by column chromatography (toluene/acetone 5:1) as a pale yellow syrup. ^1^H NMR data for this compound correlates with those previously reported by us^4^ using synthetic procedure a.

##### (*rac*)-*N*-Boc-6,7-*exo*-isopropylidendioxy-1,5-dimethyl-2,8-diazabicyclo[3.2.1]oct-2-ene (**11c**)

The synthesis of **11c** was carried out from **10c** (130.0 mg, 0.3 mmol) according to the general procedure a described above. Compound **11c** (66.2 mg, 82%) was obtained after purification by column chromatography (toluene/acetone 10:1 → 5:1) as a yellow syrup. IR (υ̅, cm^–1^) 2979, 2934, 1699 (C=O), 1456, 1367, 1283, 1249, 1211, 1158, 1088, 1065, 998, 877, 848, 776, 740, 605. ^1^H NMR (300 MHz, CDCl_3_) δ 7.66 (m, 1H, H3), 4.15 (d, *J* = 6.2 Hz, 1H, H6 or H7), 4.05 (d, *J* = 6.2 Hz, 1H, H6 or H7), 2.98–2.84 (m, 1H, H4a), 2.01–1.88 (m, 1H, H4b), 1.86 (s, 3H, C*H*_*3*_) 1.53 (s, 3H, C*H*_*3*_), 1.48 (s, 3H, C*H*_*3*_), 1.44 (s, 9H, C(C*H*_*3*_)_3_), 1.31 (s, 3H, C*H*_*3*_). ^13^C {^1^H} NMR (76 MHz, CDCl_3_) δ 162.3 (C3), 156.1 (C=O), 111.1 (*C*(CH_3_)_2_), 85.5, 84.5 (C6, C7), 81.3 (C5 or C1), 80.8 (*C*(CH_3_)_3_), 63.2 (C1 or C5), 39.8 (C4), 28.5 (C(*C*H_3_)_3_), 26.8, 25.9, 23.5, 21.4,(*C*H_3_). HRMS (ESI) *m*/*z*: [M + H]^+^ calcd for C_16_H_26_O_4_N_2_: 311.1965; found: 311.1963.

##### (*rac*)-*N*-Boc-6,7-*exo*-diacetyl-1,4-dimethyl-2,8-diazabicyclo[3.2.1]oct-2-ene (**15**)

The synthesis of **15** was carried out from **14** (94.4 mg, 0.2 mmol) according to the general procedure. Compound **15** (25.9 mg, 44%) was obtained after purification by column chromatography (DCM/acetone 10:1 → 5:1) as a yellow oil. IR (υ̅, cm^–1^) 2977, 2927, 1748 (C=O), 1699 (C=O), 1456, 1366, 1240, 1160, 1062, 946, 906, 848, 774, 748, 624. ^1^H NMR (300 MHz, CDCl_3_) δ 7.73–7.72 (m, 1H, H3), 5.05 (d, *J* = 6.9 Hz, 1H, H6 or H7), 5.00 (d, *J* = 6.9 Hz, 1H, H6 or H7), 3.04 (dd, 1H, *J* = 19.4, *J* = 1.1, H4a), 2.20–2.06 (m, 10H, H4b, 2CH_3_ of AcO, CH_3_), 1.80 (s, 3H, CH_3_), 1.44 (s, 9H, C(*CH*_3_)_3_). ^13^C {^1^H} NMR (76 MHz, CDCl_3_) δ 169.9, 169.8 (*C*OOCH_3_), 162.5 (C3), 155.3 (C=O of Boc), 81.4, 80.8, 62.6 (C1, C5, C(CH_3_)_3_), 41.5 (C4), 28.4 (C(*C*H_3_)_3_), 23.7 (CH_3_), 20.7, 20.6 (COOC*H*_3_), 20.5 (CH_3_). HRMS (ESI) *m*/*z*: [M + H]^+^ calcd for C_17_H_27_O_6_N_2_: 355.1864; found: 355.1854.

##### (2*S*,3*R*,4*S*,5*S*) and (2*R*,3*S*,4*R*,5*R*)-*N*-Boc-3-amino-2,5-bis(hydroxymethyl)-2,5-dimethyl-4-tosylpyrrolidine (**19**)

The synthesis of **19** was carried out from **18** (93.0 mg, 0.2 mmol) according to the general procedure. Compound **19** (42.4 mg, 49%) was obtained after purification by column chromatography (DCM/methanol 30:1) as a white foam. IR (υ̅, cm^–1^) 3388, 2975, 2933, 1688, 1597, 1474, 1351, 1288, 1140, 1062, 846, 817, 771, 734, 706, 671, 621. ^1^H NMR (300 MHz, DMSO-*d*_6_, 353 K) δ 7.89 (d, *J* = 8.3 Hz, 2H, Ar*H*), 7.43–7.40 (m, 2H, Ar*H*), 4.02 (d, J = 11.6 Hz, 1H, CH*H*OH), 3.95–3.84 (m, 2H, CH*H*OH), 3.79 (d, *J* = 10.2 Hz, 1H, C*H*HOH), 3.57 (dd, *J* = 12.0, 0.8 Hz, 1H, H4), 3.42 (d, J = 10.2 Hz, 1H, CH*H*OH), 3.08 (s, 2H, NH_2_), 2.42 (s, 3H, C*H*_*3*_ of Ts), 1.53 (s, 3H, C*H*_*3*_), 1.42 (s, 9H, C(C*H*_*3*_)_3_), 1.03 (s, 3H, C*H*_*3*_). ^13^C {^1^H} NMR (76 MHz, DMSO-*d*_6_, 353 K) δ 152.0 (C=O), 143.6, 138.3 (CAr), 128.9, 128.2 (CHAr), 79.0 (C(CH_3_)_3_), 74.6 (C4), 65.6, 64.9 (C2, C5), 63.0 (CH_2_OH), 62.2 (CH_2_OH), 53.6 (C3), 27.8 (C(CH_3_)_3_), 24.0 (CH_3_), 20.6 (CH_3_ of Ts), 16.0 (CH_3_). HRMS (ESI) *m*/*z*: [M + H]^+^ calcd for C_20_H_33_O_6_N_2_S: 429.2054; found: 429.2047.

##### (*rac*)-3-*exo*-Amino-5,6-*exo*-isopropylidendioxy-2-*endo*-tosyl-7-oxabicyclo[2.2.1]heptane (**9a**)

The synthesis of **9a** was carried out from **10a** (159.4 mg, 0.4 mmol) according to the general procedure. Compound **9a** (133.2 mg, 90%) was obtained after purification by column chromatography (DCM → DCM/methanol 60:1 → 40:1) as a white foam. IR (υ̅, cm^–1^) 2980, 2927, 1596, 1457, 1375, 1302, 1288, 1208, 1142, 1058, 999, 930. ^1^H NMR (300 MHz, CDCl_3_) δ 7.79 (d, *J* = 8.3 Hz, 2H, Ar*H*), 7.45–7.34 (m, 2H, Ar*H*), 5.17 (d, *J* = 5.5 Hz, 1H, H5 or H6), 4.49 (dd, *J* = 5.3, 1.3 Hz, 1H, H1), 4.43 (d, *J* = 5.6 Hz, 1H, H5 or H6), 4.18 (d, *J* = 1.3 Hz, 1H, H4), 3.48 (d, *J* = 4.2 Hz, 1H, H3), 3.17 (dd, *J* = 5.3, 4.2 Hz, 1H, H2), 2.45 (s, 3H, C*H*_*3*_ of Ts), 1.44 (s, 5H, C(C*H*_*3*_)_2_, N*H*_*2*_), 1.32 (s, 3H, C(C*H*_*3*_)_2_). ^13^C {^1^H} NMR (76 MHz, CDCl_3_) δ 145.6, 136.8 (*C*Ar), 130.5, 127.9 (*C*HAr), 112.1 (*C*(CH_3_)_2_), 88.8 (C4), 80.0 (C5 or C6), 79.8 (C1), 78.3 (C5 or C6), 74.3 (C2), 53.9 (C3), 25.8, 25.2 (C(*C*H_3_)_2_), 21.8 (*C*H_3_ of Ts). HRMS (ESI) *m*/*z*: [M + H]^+^ calcd for C_16_H_22_O_5_NS: 340.1213; found: 340.1207.

##### (*rac*)-3-*exo*-Amino-5,6-*exo*-isopropylidendioxy-2-*endo*-tosyl-bicyclo[2.2.1]heptane (**9b**)

General procedure a: The synthesis of **9b** was carried out from **10b** (132.7 mg, 0.37 mmol). Compound **9b** (92.2 mg, 74%) was obtained after purification by column chromatography (DCM/methanol 40:1) as a white foam. General procedure b: The synthesis of **9b** was carried out from **10b** (84.3 mg, 0.23 mmol). Compound **9b** (59.1 mg, 79%) was obtained after purification by column chromatography (DCM/methanol 40:1). IR (υ̅, cm^–1^) 2985, 2927, 1596, 1456, 1374, 1281, 1142, 1087, 1036, 917. ^1^H NMR (300 MHz, CDCl_3_) δ 7.80 (d, *J* = 8.3 Hz, 2H, Ar*H*), 7.42–7.32 (m, 2H, Ar*H*), 5.08–4.97 (m, 1H, H5 or H6), 4.24 (dt, *J* = 5.4, 1.2 Hz, 1H, H5 or H6), 3.40 (dd, *J* = 4.7, 1.7 Hz, 1H, H3), 2.95 (t, *J* = 4.3 Hz, 1H, H2), 2.46–2.45 (m, 4H, C*H*_*3*_ of Ts, H1), 2.21–2.16 (m, 1H, H4), 1.91–1.87 (m, 1H, H7a), 1.49–1.48 (m, 1H, H7b), 1.42 (m, 5H, C(C*H*_3_)_2_, NH_2_), 1.32 (s, 3H, C(C*H*_3_)_2_). ^13^C {^1^H} NMR (76 MHz, CDCl_3_) δ 145.1, 136.7 (*C*Ar), 130.3, 128.1 (*C*HAr), 109.7 (*C*(CH_3_)_2_), 79.5, 76.2 (C5, C6), 73.7 (C2), 51.2 (C3), 50.1 (C4), 43.6 (C1), 30.7 (C7), 25.4, 24.3. (C(*C*H_3_)_2_), 21.8 (*C*H_3_ of Ts). HRMS (ESI) *m*/*z*: [M + H]^+^ calcd for C_17_H_24_O_4_NS: 338.1421; found: 338.1421.

##### (*rac*)-3-*exo*-Amino-5,6-*exo*-isopropylidendioxy-1,4-dimethyl-2-*endo*-tosyl-7-oxabicyclo[2.2.1]heptane (**9d**)

The synthesis of **9d** was carried out from **10d** (100.0 mg, 0.3 mmol) according to the general procedure a described above. Compound **9d** (59.7 mg, 64%) was obtained after purification by column chromatography (DCM → DCM/methanol 60:1 → 40:1) as a white powder. IR (υ̅, cm^–1^) 2979, 2929, 1599, 1454, 1276, 1068, 930, 884, 709. ^1^H NMR (300 MHz, CDCl_3_) δ 7.81 (d, *J* = 8.4 Hz, 2H, Ar*H*), 7.39 (d, *J* = 7.9 Hz, 2H, Ar*H*), 5.05 (d, *J* = 5.5 Hz, 1H, H5 or H6), 4.27 (d, *J* = 5.5 Hz, 1H, H5 or H6), 3.37 (d, *J* = 4.3 Hz, 1H, H3), 2.95 (d, *J* = 4.4 Hz, 1H, H2), 2.46 (s, 3H, C*H*_*3*_ of Ts), 1.52 (bs, 2H, NH_2_), 1.47 (s, 3H, C*H*_*3*_), 1.41 (s, 3H, C*H*_*3*_), 1.35 (s, 3H, C*H*_*3*_), 1.33 (s, 3H, C*H*_*3*_). ^13^C {^1^H} NMR (76 MHz, CDCl_3_) δ 145.4, 137.1 (*C*Ar), 130.4, 128.3 (*C*HAr), 112.6 (*C*(CH_3_)_2_), 88.5, 86.0 (C1, C4), 83.7, 81.1 (C5, C6), 79.6 (C2), 58.3 (C3), 26.1, 25.8 (*C*H_3_), 21.9 (*C*H_3_ of Ts), 16.3, 11.6 (*C*H_3_). HRMS (ESI) *m*/*z*: [M + H]^+^ calcd for C_18_H_25_O_5_NS: 368.1526; found: 368.1522.

### Quantum Mechanical Calculations

Full geometry optimizations and transition structure (TS) searches were carried out with Gaussian 16^14^ using the M06-2X hybrid functional,^15^ 6-31G(d,p) basis set for C, N, O, S, and H, and LanL2DZ^16^ effective core potential for the Sn atoms with ultrafine integration grids. Bulk solvent effects in toluene were considered implicitly through the integral equation formalism polarizable continuum model (IEF-PCM).^17^ The possibility of different conformations was considered for all structures. All stationary points were characterized by a frequency analysis performed at the same level used in the geometry optimizations from which thermal corrections were obtained at 383.75 K. The quasi-harmonic approximation reported by Truhlar et al. was used to replace the harmonic oscillator approximation for the calculation of the vibrational contribution to enthalpy and entropy.^18^ Scaled frequencies were not considered. Mass-weighted intrinsic reaction coordinate (IRC) calculations were carried out using the Hratchian and Schlegel scheme^19^ to ensure that the TSs indeed connected the appropriate reactants and products. For the calculation of bond dissociation energies (BDEs), single-point energy calculations using the correlated ab initio spin-component-scaled SCS-MP2 method, in combination with the cc-pVTZ basis set,^20^ were performed on the M06-2X/6-31G(d)-optimized geometries. BDEs were defined as the difference in zero-point energies between the neutral species and the sum of the isolated radicals generated upon homolytic C–H cleavage (Table S2). Gibbs free energies (Δ*G*) were used for the discussion on the relative stabilities of the considered structures. The lowest energy conformer for each calculated stationary point (Figure S6) was considered in the discussion; all the computed structures can be obtained from authors upon request. Electronic energies, entropies, enthalpies, Gibbs free energies, and lowest frequencies of the calculated structures are summarized in Table S1. Cartesian coordinates of the lowest energy structures calculated with PCM(toluene)/M06-2X/6-31G(d,p) are shown in Table S3.

## Supporting Information Available

Experimental protocols, compound characterization data, computational details, and copy of ^1^H and ^13^C NMR spectra for new compounds (PDF)
