# Supplementary material for: Studies on the Regioselective Rearrangement of Azanorbornanic Aminyl Radicals into 2,8-Diazabicyclo[3.2.1]oct-2-ene Systems
Source: J Org Chem. 2022 Dec 1;87(24):16483–91. doi: 10.1021/acs.joc.2c02201 (PMC9764359; doi:10.1021/acs.joc.2c02201)
Supplement: Supplementary file 1 — jo2c02201_si_001.pdf [file jo2c02201_si_001.pdf]

SUPPORTING INFORMATION

Studies on the regioselective rearrangement of azanorbornanic aminyl radicals into 2,8-diazabicyclo[3.2.1]oct-2-ene systems

Enrique Gil de Montes,[a] Matteo A. Tallarida,[b,c] Ana T. Carmona,[a] Claudio D. Navo,[b] Inmaculada Robina,[a] Pilar Elías-Rodríguez,[a] Gonzalo Jiménez-Osés[b,d]* and Antonio J. Moreno-Vargas[a]*

[a]Departamento de Química Orgánica (Facultad de Química), Universidad de Sevilla, C/ Prof. García González, 1, 41012-Sevilla (Spain). E-mail: ajmoreno@us.es
[b]Center for Cooperative Research in Biosciences (CIC bioGUNE), Basque Research and Technology Alliance (BRTA), Bizkaia Technology Park, Building 800, 48160 Derio, Spain. E-mail: gjoses@cicbiogune.es
[c]Department of Chemistry and Chemical Technologies, University of Calabria, Via P. Bucci, Cubo 12C, 87036, Rende, Italy.
[d] Ikerbasque, Basque Foundation for Science, 48013 Bilbao, Spain.

Table of contents

1. Synthesis of alkyne **5** and pyrrole **6c**... S2

2. Synthesis of (7-hetero)norbornadienes **7a-d**.. S3

3. Synthesis of azanorbornadienic β-azido sulfone **14** and intermediate compounds... S4

4. Synthesis of pyrrolidinic β-azido sulfone **18** and intermediate compounds…..... S6

5. Quantum Mechanical calculations…………………………………………………. S8

6. References………………………………………………………………………… S39

7. ^{1}H- and ^{13}C-NMR spectra for new compounds…………………………………… S40

8. COSY and HSQC spectra for new compounds…………………………………… S59

1. Synthesis of alkyne 5 and pyrrole 6c

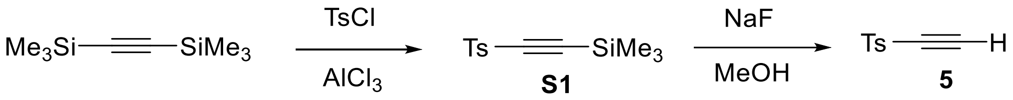

p-Tolyl [(2-trimethylsilyl)ethynyl] sulfone (S1)[1]

A mixture of $AlCl_3$ (14.7 g, 110 mmol) and tosyl chloride (21.9 g, 114 mmol) was dissolved in dry DCM (100 mL) and stirred under argon atmosphere for 20 min at r.t. After this time, the crude was filtered through celite and the resultant liquid was added dropwise to a solution of commercial bis(trimethylsilyl) acetylene (23 mL, 102 mmol) in dry DCM (100 mL) at 0 °C for 1 h. After the addition, the reaction was allowed to warm at r.t. overnight. Once the reaction has finished, the mixture was poured into a solution of HCl in cold water (20%). The organic layer was washed with an aqueous solution of HCl (1M), with brine, and dried over anhydrous Na_2SO_4. The solvent was removed under reduced pressure and the resultant solid was recrystallized from Cy affording the desired product **S1** as a grey powder (14.1 g, 55%). ^{1}H-NMR (300 MHz, $CDCl_3$, 298K, δ ppm, J Hz) δ 7.90-7.87 (m, 2H, ArH), 7.39-7.36 (m, 2H, ArH), 2.47, (s, 3H, CH_3), 0.12 (s, 9H, $Si(CH_3)_3$).

Ethynyl *p*-tolyl sulfone (5)[2]

To a solution of *p*-tolyl [(2-trimethylsilyl)ethynyl] sulfone **S1** (2.0 g, 8.1 mmol) in methanol (16 mL), a solution of NaF (504 mg, 12.0 mmol) in water (8 mL) was added dropwise at 0 °C, and the reaction was stirred at this temperature for 30 min. Then, the reaction was diluted with diethyl ether and washed with an aqueous saturated solution of $NaHCO_3$. The organic layer was separated, dried over anhydrous Na_2SO_4 and the solvent was removed under reduced pressure, affording **5** as a white powder (1.4 g, 94%) without the need of a further purification step. ^{1}H-NMR (300 MHz, $CDCl_3$, 298 K, *δ* ppm, *J* Hz) *δ* 7.92-7.89 (m, 2H, Ar*H*), 7.41-7.38 (m, 2H, Ar*H*), 3.45 (s, 1H, C≡C-*H*), 2.47 (s, 3H, CH_3 of Ts).

N-Boc-2,5-dimethylpyrrole (6c)[3]

To a stirred solution of commercial 2,5-dimethylpyrrole (1.9 mL, 21 mmol) in acetonitrile (20 mL), 4-dimethylaminopyridine (260 mg, 2.1 mmol) and Boc_2O (4.82 g, 22 mmol) were added. After 24 h at r.t., all the starting material was consumed (TLC) and the mixture was diluted with Et_2O and washed with a 1M aqueous solution of $NaHSO_4$,

water, and a 1M solution of $NaHCO_3$. The organic layer was dried over anhydrous Na_2SO_4 and filtered. The solvent was removed under reduced pressure. The compound was purified by silica gel column chromatography (EtOAc/Cy 1:40) affording the desired product **6c** (3.0 g, 73%) as a brown oil. ^{1}H-NMR (300 MHz, $CDCl_3$, 298 K, *δ* ppm, *J* Hz) *δ* 5.79 (s, 2H, Ar*H*), 2.38 (s, 6H, C*H₃*), 1.60 (s, 9H, C(C*H₃*)₃).

2. Synthesis of (7-hetero)norbornadienes 7a-d

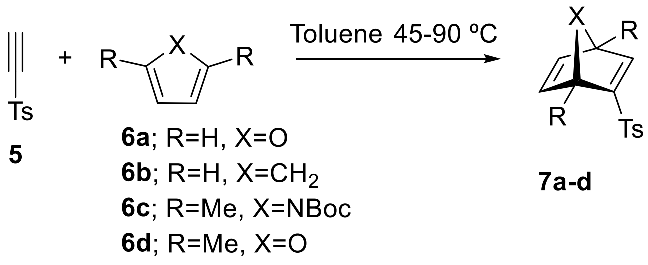

***(rac)*-2-Tosyl-7-oxabicyclo[2.2.1]hepta-2,5-diene (7a).**[4] To a solution of ethynyl *p*-tolyl sulfone **5** (1.7 g, 9.6 mmol) in dry toluene (13 mL) under argon atmosphere, commercial furan **6a** (7.0 mL, 96 mmol) was added. The mixture was heated at 70 °C for 4 h. Then, the solvent was removed and the reaction mixture was purified by silica gel column chromatography (DCM→ DCM/Acetone 40:1), affording **7a** as a brown powder (1.1 g, 46%). IR (ῡ, cm^{-1}) 1593, 1314, 1305, 1291, 1270, 1148, 1120, 1108, 1085, 1041, 1018, 1003, 874, 864, 813, 798, 753, 704, 664, 652. ^{1}H-NMR (300 MHz, $CDCl_3$) *δ* 7.76-7.73 (m, 2H, Ar*H*), 7.60 (d, 1H, *J*= 1.9, H3), 7.37-7.34 (m, 2H, Ar*H*), 7.05 (dd, 1H, *J*= 5.3, *J* = 1.9, H5 or H6), 6.97 (dd, 1H, *J* = 5.3, *J* = 1.9, H5 or H6), 5.63-5.62 (m, 1H, H1 or H4), 5.37 (m, 1H, H1 or H4), 2.44 (s, 3H, CH_3 of Ts). ^{13}C {^{1}H} NMR (75.4 MHz, $CDCl_3$, 298 K, *δ* ppm) *δ* 160.0, 152.2 (C5 or C6), 145.1 (C2 or C3), 143.9 (*C*HAr), 142.3 (*C*HAr), 135.8 (C2 or C3), 130.2 (*C*Ar), 128.1 (*C*Ar), 84.3, 82.6 (C1, C4), 21.8 (C*H₃* of Ts). HRMS (ESI) *m/z:* $[M+Na]^+$ calcd. for $C_{13}H_{12}O_3SNa$: 271.0396, found: 271.0399.

(*rac*)-2-Tosylbicyclo[2.2.1]hepta-2,5-diene (7b).[5] Commercially available dicyclopentadiene **6b** (3.9 g, 29.5 mmol) was cracked at 200 ºC and condensed over a solution of ethynyl *p*-tolyl sulfone **5** (0.4 g, 2.3 mmol) in dry toluene (3 mL) under argon atmosphere. Once all the dicyclopentadiene was cracked, the toluene was removed and the reaction mixture was purified by column chromatography on silica gel (Et_2O/Cy 1:2) affording **7b** as a white powder (0.5 g, 95%). ^{1}H-NMR (300 MHz, $CDCl_3$, 298 K *δ* ppm, *J* Hz) *δ* 7.70-7.68 (m, 2H, Ar*H*), 7.46-7.45 (m, 1H, H3), 7.32-7.29 (m, 2H, Ar*H*), 6.61 (m, 2H, H5, H6), 3.80-3.77 (m, 1H, H4), 3.68 (m, 1H, H1), 2.40 (s, 3H, C*H*₃ of Ts), 2.19-2.06 (m, 2H, H7a, H7b).

***(rac)*-*N*-Boc-1,4-dimethyl-2-tosyl-7-azabicyclo[2.2.1]hepta-2,5-diene (7c).** To a solution of

N-Boc-2,5-dimethylpyrrole **6c** (3.0 g, 15 mmol) in dry toluene under Ar atmosphere, ethynyl *p*-tolyl sulfone **5** (570 mg, 3.1 mmol) was added. The mixture was heated at 90 °C overnight. Then, toluene was removed under reduced pressure and the reaction mixture was purified by silica gel column chromatography (EtOAc/Cy 1:5) to afford **7c** as a yellowish powder (970 mg, 85%). IR ($\bar{\upsilon}$ cm^{-1}) 3082, 2978, 1695, 1603, 1556, 1456, 1307, 1143, 822, 790. ^1H NMR (300 MHz, $CDCl_3$) δ 7.72 (d, *J* = 8.3 Hz, 2H, Ar*H*), 7.49 (s, 1H, H3), 7.39 – 7.30 (m, 2H, Ar*H*), 6.72 (d, *J* = 5.2 Hz, 1H, H5 or H6), 6.59 (d, *J* = 5.3 Hz, 1H, H5 or H6), 2.44 (s, 3H, C*H₃* or Ts), 1.97 (s, 3H, CH_3), 1.83 (s, 3H, CH_3), 1.32 (s, 9H, $C(CH_3)_3$). ^{13}C {^1H} NMR (76 MHz, $CDCl_3$) δ 160.5 (C=O), 159.7 (C3), 154.7 (C2), 149.3, 146.9 (C5, C6), 144.8, 136.6 (*CAr*), 129.9, 128.3 (*C*HAr), 81.4 ($C(CH_3)_3$), 77.7, 76.6 (C1, C4), 28.3 ($C(CH_3)_3$), 21.8 (CH_3 of Ts), 17.4, 16.3 (CH_3). HRMS (ESI) *m/z:* $[M+Na]^+$ calcd. for $C_{20}H_{25}NO_4SNa$: 398.1402; found: 398.1391.

***(rac)*-1,4-Dimethyl-2-tosyl-7-oxabicyclo[2.2.1]hepta-2,5-diene (7d).** To a solution of ethynyl *p*-tolyl sulfone **5** (1.2 g, 6.4 mmol) in dry toluene (20 mL) under argon atmosphere, commercial 2,5-dimethylfuran **6d** (3.4 mL, 32 mmol) was added. The mixture was heated at 50 °C overnight. Then, the solvent was removed and the reaction mixture was purified by silica gel column chromatography (Et_2O/Cy 1:6), affording **7d** as a brown solid (1.4 g, 79%). IR ($\bar{\upsilon}$, cm^{-1}) 1596, 1446, 1384, 1301, 1224, 1145, 1086, 982, 815, 749. ^1H NMR (300 MHz, $CDCl_3$) δ 7.71 (d, *J* = 8.3 Hz, 2H, Ar*H*), 7.41 (s, 1H, H3), 7.33 (d, *J* = 8.3 Hz, 2H, Ar*H*), 6.82 (d, *J* = 5.1 Hz, 1H, H5 or H6), 6.73 (d, *J* = 5.1 Hz, 1H, H5 or H6), 2.44 (s, 3H, CH_3 of Ts), 1.76 (s, 3H, CH_3), 1.62 (s, 3H, CH_3). ^{13}C {^1H} NMR (76 MHz, $CDCl_3$) δ 161.1 (C2), 157.6 (C3), 148.6, 146.2(C5, C6), 144.8, 136.2 (*CAr*), 130.0, 128.2 (*C*HAr), 91.9, 91.3 (C1, C4), 21.8 (CH_3 of Ts), 16.5 (CH_3), 15.6 (CH_3). HRMS (ESI) *m/z:* $[M+Na]^+$ calcd. for $C_{15}H_{16}O_3SNa$: 299.0718, found: 299.0713.

3. Synthesis of azanorbornadienic β-azido sulfone 14 and intermediate compounds

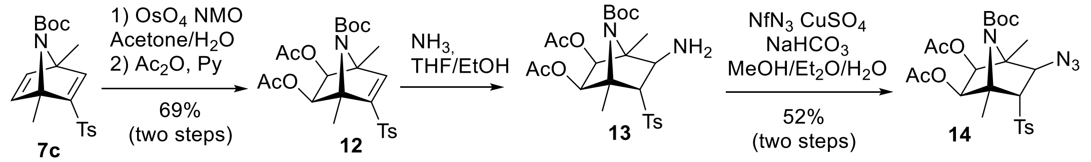

Caution: *For organic azides to be non-explosive, the number of nitrogen atoms must not exceed that of carbon and that (N_C + N_O)/N_N ≥3 (N= number of atoms).*

***(rac)*-*N*-Boc-5,6-*exo*-di-O-diacetyl-1,4-dimethyl-2-tosyl-7-azabicyclo[2.2.1]hept-2-ene (12).** To a solution of **7c** (500.0 mg, 1.3 mmol) in acetone (33 mL) and water (4 mL), NMO (324.0 mg, 2.4 mmol), and OsO_4 (4 wt. % in H_2O, 0.8 mL, 0.13 mmol) were added. The mixture was stirred for 1 h at r.t. Then, the solution was cooled at 0 ºC and a saturated aqueous solution

of $NaHSO_3$ was added. The reaction mixture was extracted with EtOAc and the organic layer was washed with brine and dried over Na_2SO_4. The solvent was removed *in vacuo* and the resultant residue was dissolved in pyridine (20 mL). Then, acetic anhydride (10 mL) was added. The reaction was stirred at room temperature overnight. The solvent was removed under reduced pressure and the reaction mixture was purified by chromatography column on silica gel (EtOAc/Cy 1:3 → 1:2) affording **12** (450 mg, 69%) as a white powder. IR ($\bar{\upsilon}$ cm^{-1}) 3083, 2981, 2941, 1738 (C=O), 1710 (C=O), 1596, 1369, 1331, 1245, 1222, 1156, 1090, 959, 943, 820, 700, 665, 604. ^1H NMR (300 MHz, $CDCl_3$) δ 7.75 (d, *J* = 8.3 Hz, 2H, Ar*H*), 7.42 – 7.31 (m, 2H, Ar*H*), 6.81 (s, 1H, H3), 4.91 (d, *J* = 6.1 Hz, 1H, H5 or H6), 4.80 (d, *J* = 6.1 Hz, 1H, H5 or H6), 2.45 (s, 3H, C*H₃* of Ts), 2.09 (s, 3H, COOCH_3), 2.08 (s, 3H, COOCH_3), 1.71 (s, 3H, CH_3), 1.70 (s, 3H, CH_3), 1.35 (s, 9H, C(CH_3)$_3$)). ^{13}C {^1H} NMR (76 MHz, $CDCl_3$) δ 169.89, 169.86 (*C*OOCH$_3$), 155.0 (C=O Boc), 152.5 (C2), 149.7 (C3), 145.5, 136.2 (*C*Ar), 130.2, 128.4 (*C*HAr), 81.6 (*C*(CH$_3$)$_3$), 72.82, 72.80 (C1, C4), 72.3, 70.8 (C5, C6), 28.3 (C(*C*H$_3$)$_3$), 21.8 (*C*H$_3$ of Ts), 20.62, 20.58 (COO*C*H$_3$), 15.5, 14.1 (*C*H$_3$). HRMS (ESI) *m/z:* $[M+Na]^+$ calcd. for $C_{24}H_{31}NO_8SNa$: 516.1663; found: 516.1661.

***(rac)-N*-Boc-3-*exo*-azido-5,6-*exo*-di-*O*-acetyl-1,4-dimethyl-2-*endo*-tosyl-7-azabicyclo-[2.2.1]heptane (14).** Compound **12** (358 mg, 0.8 mmol) was dissolved in EtOH (20 mL) and THF (20 mL) at 0 ºC. Then, NH_3 gas was bubbled for 2 min and the reaction was allowed to warm to r.t. for 10 min. Then, the solvent was removed under reduced pressure to give bicyclic amine **13** that was used without purification in the next step. To a solution of **13** in MeOH (3 mL) and water (1 mL), $NaHCO_3$ (497 mg, 5.9 mmol), a solution of nonaflyl azide (540 mg, 1.7 mmol) in Et_2O (2.3 mL) and $CuSO_4·5H_2O$ (50 mg, 0.2 mmol) were added and the mixture was stirred at r.t. for 24 h. The organic solvents were removed under reduced pressure and the resultant aqueous mixture was extracted with DCM and washed with an aqueous saturated solution of $NaHCO_3$. The organic layer was dried over Na_2SO_4, filtered, the solvent was removed under reduced pressure. The crude product was purified by column chromatography on silica gel (EtOAc/ Cy 1:4 →1:2) affording **14** as a white powder (231.7 mg, 50%). IR ($\bar{\upsilon}$ cm^{-1}) 2979, 2936, 2107 (N_3), 1751 (C=O), 1703 (C=O), 1596, 1457, 1366, 1342, 1291, 1238, 1146, 1083, 848. ^1H NMR (300 MHz, $CDCl_3$) δ 7.92 – 7.79 (m, 2H, Ar*H*), 7.47 – 7.38 (m, 2H, Ar*H*), 5.78 (d, *J* = 6.6 Hz, 1H, H5 or H6), 5.13 (d, *J* = 6.6 Hz, 1H, H5 or H6), 4.05 (d, *J* = 4.0 Hz, 1H, H2 or H3), 3.44 (d, *J* = 4.0 Hz, 1H, H2 or H3), 2.46 (s, 3H, *C*H$_3$ of Ts), 2.10 (s, 3H, COOCH_3), 2.09 (s, 3H, COOCH_3), 1.59 (s, 3H, CH_3), 1.53 (s, 3H, CH_3), 1.43 (s, 9H, C(CH_3)$_3$). ^{13}C {^1H} NMR (76 MHz, $CDCl_3$) δ 169.7, 169.5 (*C*OOCH$_3$), 155.3 (C=O Boc), 146.2, 136.0 (*CAr*), 130.5, 128.7 (*C*HAr), 81.7 (*C*(CH$_3$)$_3$), 74.6 (C2 or C3), 73.7 (C5 or C6), 72.7 (C1 or C4), 72.3 (C5 or C6), 70.1 (C1 or C4), 68.5 (C2 or C3), 28.3 (C(*C*H$_3$)$_3$), 21.9 (*C*H$_3$ of Ts), 20.6, 20.5

($COOCH_3$), 17.3, 14.2 (CH_3). HRMS (ESI) *m/z:* $[M+Na]^+$ calcd. for $C_{24}H_{32}N_4O_8SNa$: 559.1833; found: 559.1823.

4. Synthesis of pyrrolidinic β-azido sulfone 18 and intermediate compounds

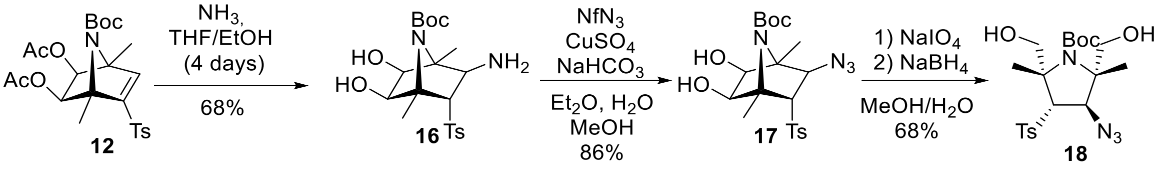

***(rac)-N-Boc*-1,4-dimethyl-2-*endo*-tosyl-3-*exo*-amino-7-azabicyclo[2.2.1]heptane-exo-5,6-diol (16).** To a solution of **12** (225.2 mg, 0.5 mmol) in THF (20 mL) and EtOH (20 mL), NH_3 was bubbled at 0 ºC for 5 min. Then the solution was allowed to warm to r.t. After 4 days, the solvent was removed and the reaction mixture was purified by a chromatography column onsilica gel (DCM/MeOH 50:1) affording **16** (147 mg, 68%) as a white foam. IR ($\bar{\upsilon}$ cm^{-1}) 3403, 2971, 2359, 1687 (C=O), 1596, 1456, 1364, 1138, 1085, 1058, 1024, 984, 953, 917, 807, 707, 661. ^1H-NMR (300 MHz, DMSO-d_6, 353 K) δ 7.80 (d, *J* = 8.3 Hz, 2H, Ar*H*), 7.46 (d, *J* = 8.1 Hz, 2H, Ar*H*), 5.09 (d, *J* = 6.2 Hz, 1H, O*H*), 4.63 (d, *J* = 6.6 Hz, 1H, O*H*), 4.26 (t, *J* = 6.3 Hz, 1H, H5 or H6), 3.48 (t, *J* = 6.5 Hz, 1H, H5 or H6), 3.08 (d, *J* = 4.3 Hz, 1H, H2 or H3), 3.06 (d, *J* = 4.3 Hz, 1H H2 or H3), 2.42 (s, 3H, C*H₃* of Ts), 1.60 (s, 3H, CH_3), 1.33 (s, 12H, $C(CH_3)_3$, CH_3). ^{13}C {^1H} NMR (76 MHz, DMSO-d_6, 353 K) δ 155.5 (C=O), 144.6, 137.3 (*C*Ar), 130.0, 128.1 (*C*HAr), 78.8 (*C*(CH_3)₃), 76.2 (C2 or C3), 73.0 (C5 or C6), 70.4 (C5 or C6), 69.9 (C1, C4), 59.2 (C2 or C3), 28.0 ($C(CH_3)_3$), 21.1 (CH_3 of Ts), 17.8, 13.8 (CH_3). HRMS (ESI) *m/z:* $[M+H]^+$ calcd. for $C_{20}H_{31}N_2O_6S$: 427.1897; found: 427.1888.

***(rac)-N*-Boc-3-*exo*-azido-1,4-dimethyl-2-*endo*-tosyl-7-azabicyclo[2.2.1]heptane-5,6-exo-diol (17).** To a solution of **16** (131.1 mg, 0.31 mmol) in MeOH (3.5 mL) and water (0.5 mL), $NaHCO_3$ (104.2 mg, 1.2 mmol), $CuSO_4·5H_2O$ (20.0 mg, 0.1 mmol) and a solution of nonaflyl azide (201.6 mg, 0.6 mmol) in Et_2O (2.1 mL) were added. The reaction was stirred at r.t. for 3.5 h. Then, the reaction was diluted with DCM, washed with water and a saturated solution of $NaHCO_3$.The organic layer was dried over Na_2SO_4, filtered and the solvent was removed under reduced pressure. The crude product was purified by column chromatography on silica gel (EtOAc/Cy 1:3→1:2), affording **17** (113.3 mg, 87%) as a white powder. IR ($\bar{\upsilon}$ cm^{-1}) 3388, 2979, 2930, 2103 (N_3), 1699 (C=O), 1597, 1455, 1366, 1289, 1255, 1143, 1083, 951. ^1H NMR (300 MHz, $CDCl_3$) δ 7.80 (d, *J* = 8.4 Hz, 2H, Ar*H*), 7.46 – 7.37 (m, 2H, Ar*H*), 4.64 (d, *J* = 6.1 Hz, 1H, H5 or H6), 3.84 (d, *J* = 6.1 Hz, 1H, H5 or H6), 3.75 (d, *J* = 3.9 Hz, 1H, H3), 3.34 (d, *J* = 3.9 Hz, 1H, H2), 2.47 (s, 3H, CH_3 of Ts), 1.81 (s, 3H, CH_3), 1.60 (s, 3H, CH_3), 1.42 (s,

9H, C(CH_3)$_3$). ^{13}C {^{1}H} NMR (76 MHz, $CDCl_3$) δ 157.3 (C=O), 146.1, 136.5 (*C*Ar), 130.6, 128.4 (*C*HAr), 81.8 (*C*(CH_3)$_3$), 74.7(C2), 74.4 (C1 or C4), 74.3 (C5 or C6), 71.8 (C1 or C4), 71.6 (C5 or C6), 68.0 (C3), 28.3 (*C*(CH_3)$_3$), 21.9 (*C*H_3 of Ts), 17.4, 14.2 (*C*H_3). HRMS (ESI) *m/z:* $[M+Na]^+$ calcd. for $C_{20}H_{28}N_4O_6SNa$: 475.1622; found: 475.1614.

(2*S*,3*R*,4*S*,5*S*) and (2*R*,3*S*,4*R*,5*R*)-*N*-Boc-3-azido-2,5-bis(hydroxymethyl)-2,5-dimethyl-4-tosyl-pyrrolidine (18). To a solution of **17** (96.0 mg, 0.2 mmol) in MeOH (2 mL) and water (0.6 mL) at 0 ºC, $NaIO_4$ (98.4 mg, 0.5 mmol) was added. After stirring the reaction at r.t. for 1 h, the reaction mixture was filtered over celite. Then, $NaBH_4$ (17.4 mg, 0.5 mmol) was added to the filtrate and the reaction was stirred at r.t. for 15 min. Next, a saturated aq. solution of citric acid was added, and the mixture was diluted with EtOAc and washed with water. The organic layer was dried over Na_2SO_4, filtered and the solvent was removed under reduced pressure. The product was purified through column chromatography on silica gel (EtOAc/Cy 1:3→1:2) affording **18** (71.5 mg, 68%) as a white foam. IR ($\bar{\upsilon}$ cm^{-1}) 3484, 2974, 2928, 2114 (N_3), 1687 (C=O), 1598, 1473, 1390 1353, 1294, 1254, 1138, 1083, 1056, 1000, 870. ^{1}H NMR (300 MHz, DMSO-*d*$_6$, 353K) δ 8.13 – 7.67 (m, 2H, Ar*H*), 7.49 (d, *J* = 8.2 Hz, 2H, Ar*H*), 4.73 (d, *J* = 11.9 Hz, 1H, H3), 4.07-3.92 (m, 2H, C*H*$_2$OH), 3.84 (d, *J* = 10.5 Hz, 1H, C*H*HOH), 3.69 (d, *J* = 5.2 Hz, 1H, H4), 3.65 (d, *J* = 3.8 Hz, 1H, C*H*HOH), 2.44 (s, 3H, CH_3 of Ts), 1.61 (s, 3H, CH_3), 1.43 (s, 9H, C(CH_3)$_3$), 1.12 (s, 3H, CH_3). ^{13}C {^{1}H} NMR (75.5 MHz, DMSO-*d*$_6$, 353K) δ 151.5 (C=O), 144.2, 138.3 (*C*Ar), 129.3, 127.2 (*C*HAr), 79.4 (*C*(CH_3)$_3$), 70.7 (C4), 66.6 (C2 or C5), 64.9 (*C*H_2OH), 64.5 (C2 or C5), 63.6 (C3), 61.9 (*C*H_2OH), 27.8 (*C*(CH_3)$_3$), 23.6 (*C*H_3), 20.6 (*C*H_3 of Ts), 17.4 (*C*H_3). HRMS (ESI) *m/z:* $[M+Na]^+$ calcd. for $C_{20}H_{30}N_4O_6SNa$: 477.1778; found: 477.1773.

5.Quantum Mechanical calculations

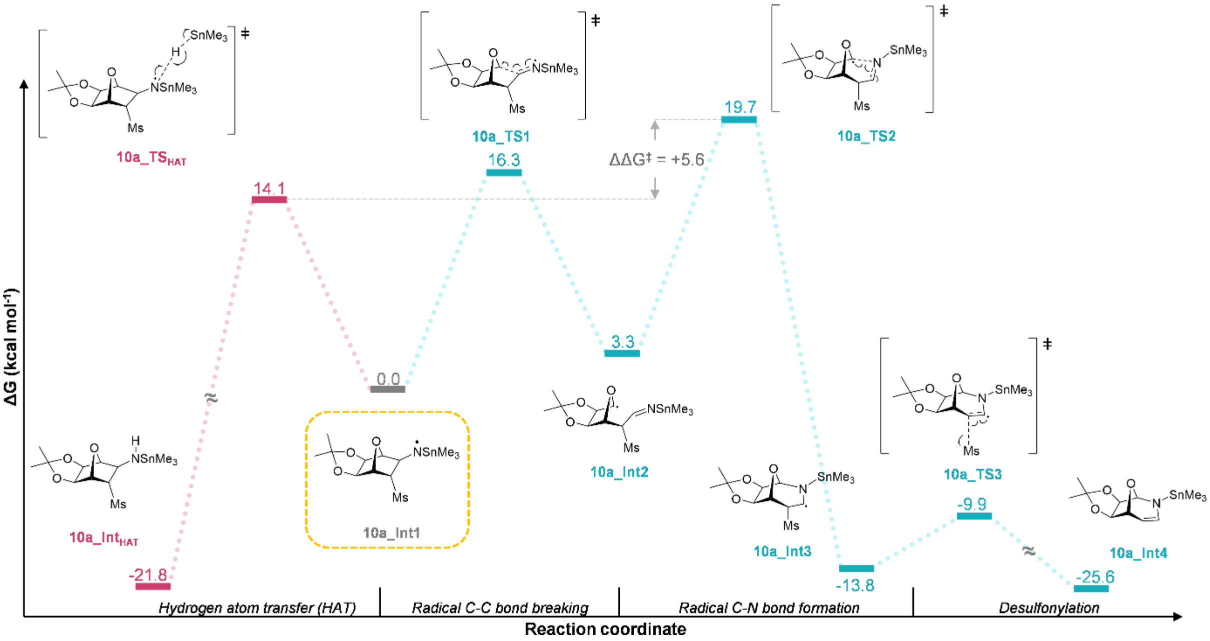

Figure S1. Minimum energy reaction pathway for the model of oxonorbornane derivative **10a** calculated with PCM(toluene)/M06-2X/6-31G(d,p)+LanL2DZ(Sn).

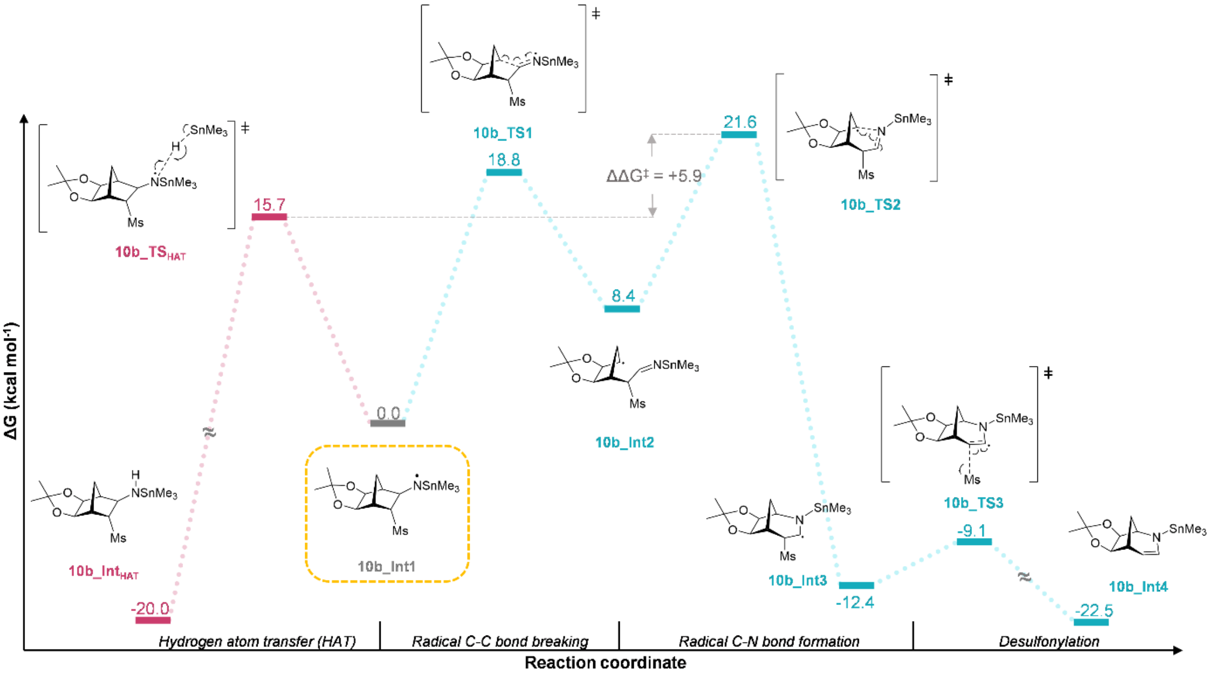

Figure S2. Minimum energy reaction pathway for the model of norbornane derivative **10b** calculated with PCM(toluene)/M06-2X/6-31G(d,p)+LanL2DZ(Sn).

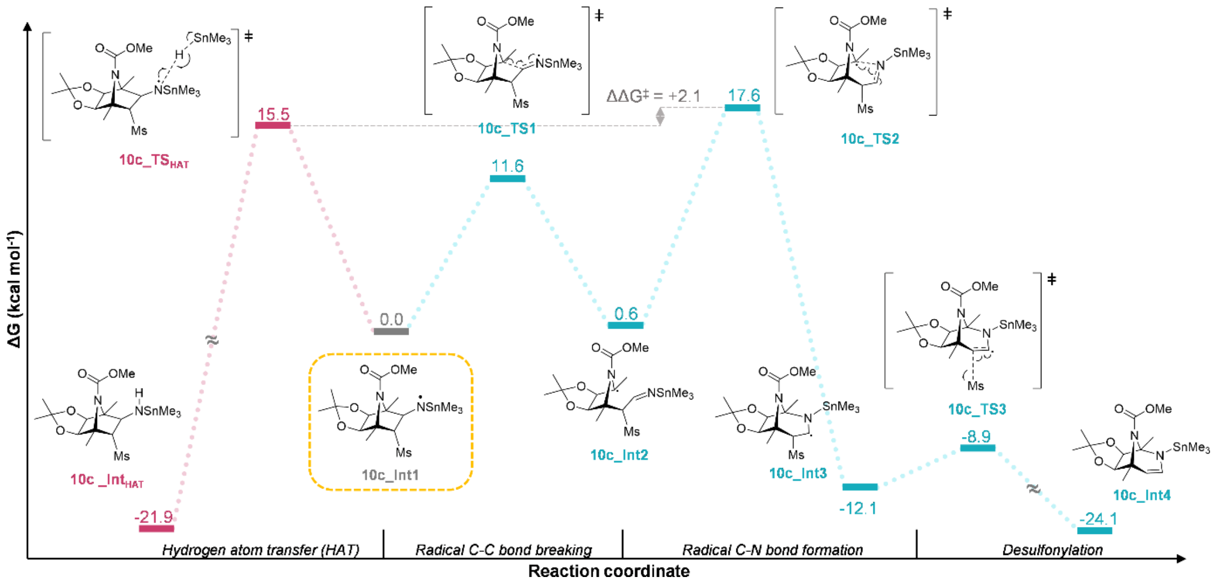

Figure S3. Minimum energy reaction pathway for the model of 1,4-dimethylazanorbornane derivative **10c** calculated with PCM(toluene)/M06-2X/6-31G(d,p)+LanL2DZ(Sn).

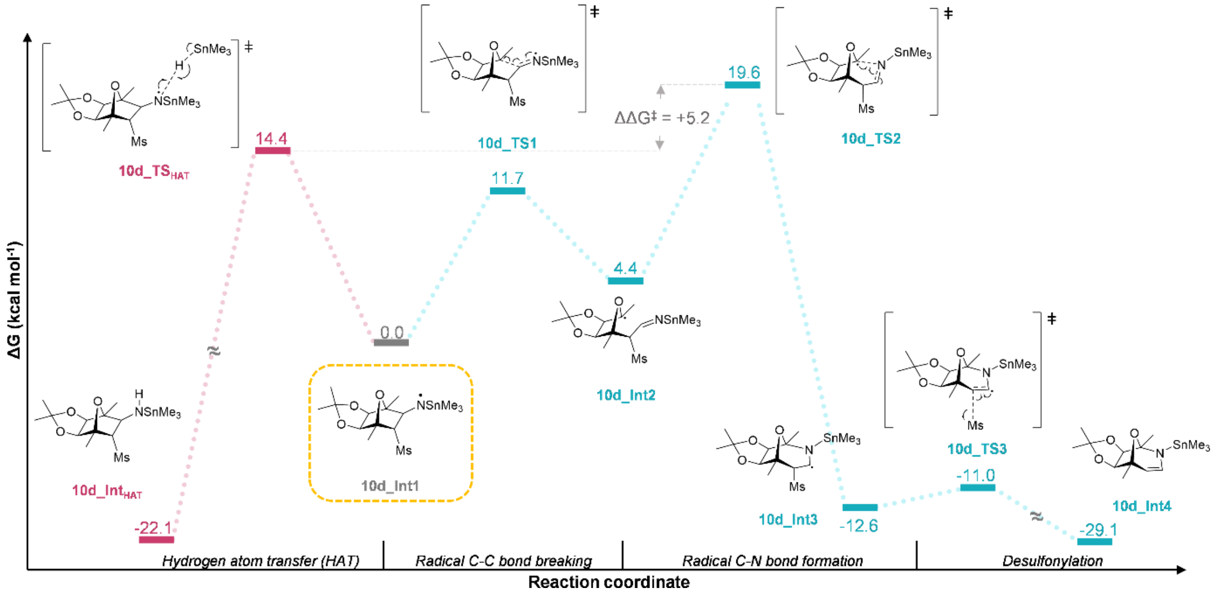

Figure S4. Minimum energy reaction pathway for the model of 1,4-dimethyloxonorbornane derivative **10d** calculated with PCM(toluene)/M06-2X/6-31G(d,p)+LanL2DZ(Sn).

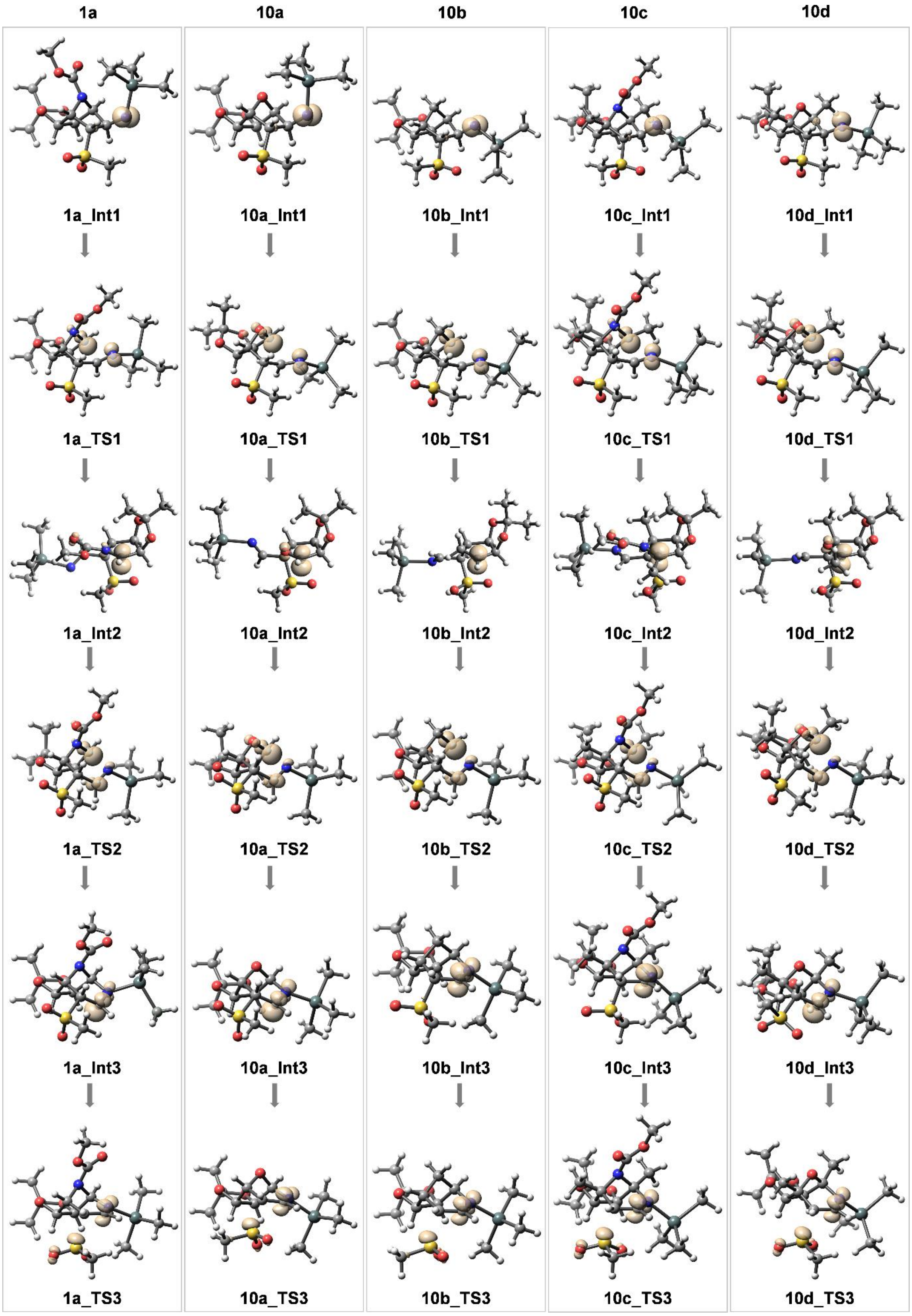

Figure S5. Spin density of the radicals intermediates and transition structures for the ring expansion reaction calculated with PCM(toluene)/M06-2X/6-31G(d,p)+LanL2DZ(Sn).

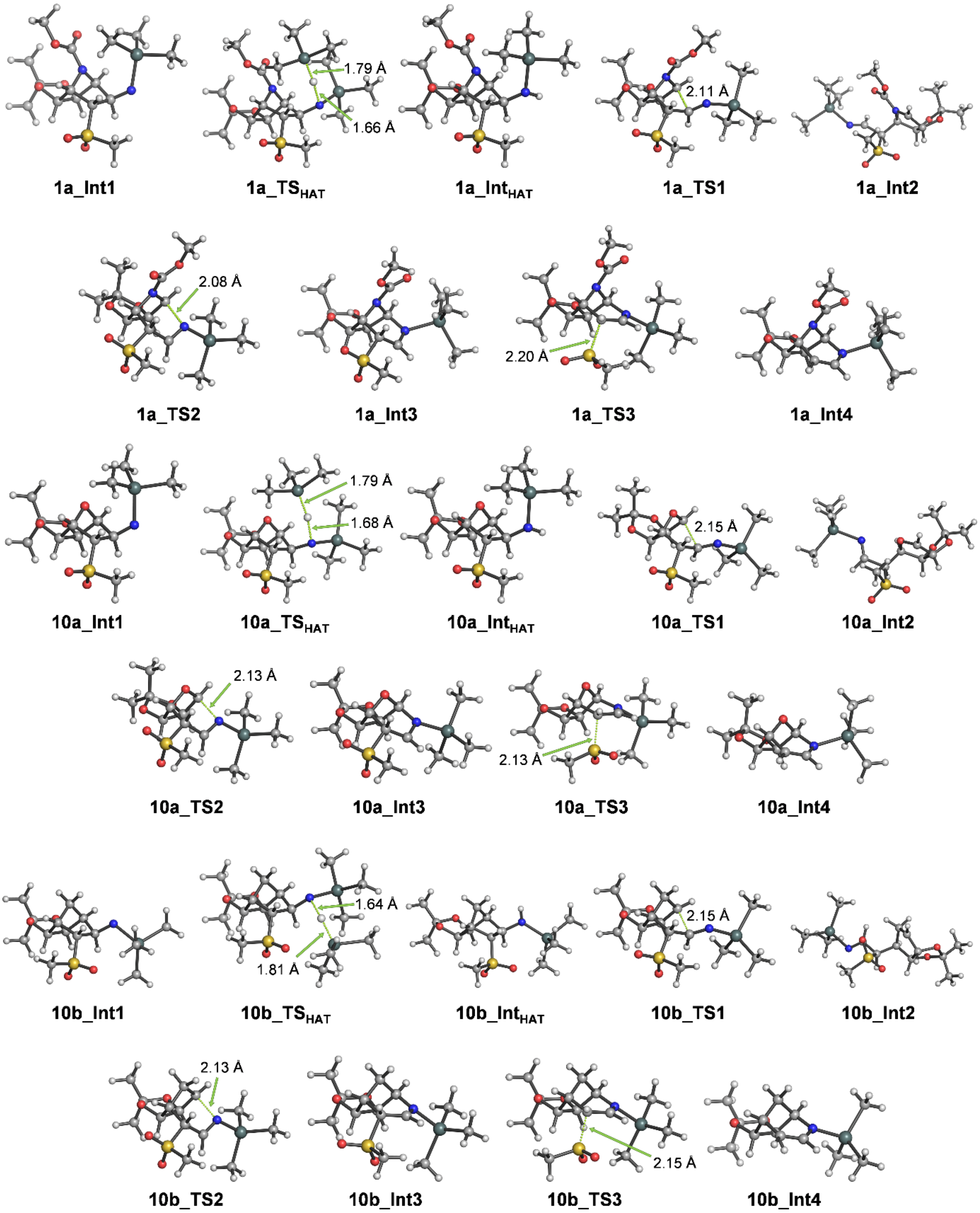

1.79 Å
1.66 Å
2.11 Å
1a_Int1
1a_TS$_{HAT}$
1a_Int$_{HAT}$
1a_TS1
1a_Int2
2.08 Å
2.20 Å
1a_TS2
1a_Int3
1a_TS3
1a_Int4
1.79 Å
1.68 Å
2.15 Å
10a_Int1
10a_TS$_{HAT}$
10a_Int$_{HAT}$
10a_TS1
10a_Int2
2.13 Å
2.13 Å
10a_TS2
10a_Int3
10a_TS3
10a_Int4
1.64 Å
1.81 Å
2.15 Å
10b_Int1
10b_TS$_{HAT}$
10b_Int$_{HAT}$
10b_TS1
10b_Int2
2.13 Å
2.15 Å
10b_TS2
10b_Int3
10b_TS3
10b_Int4

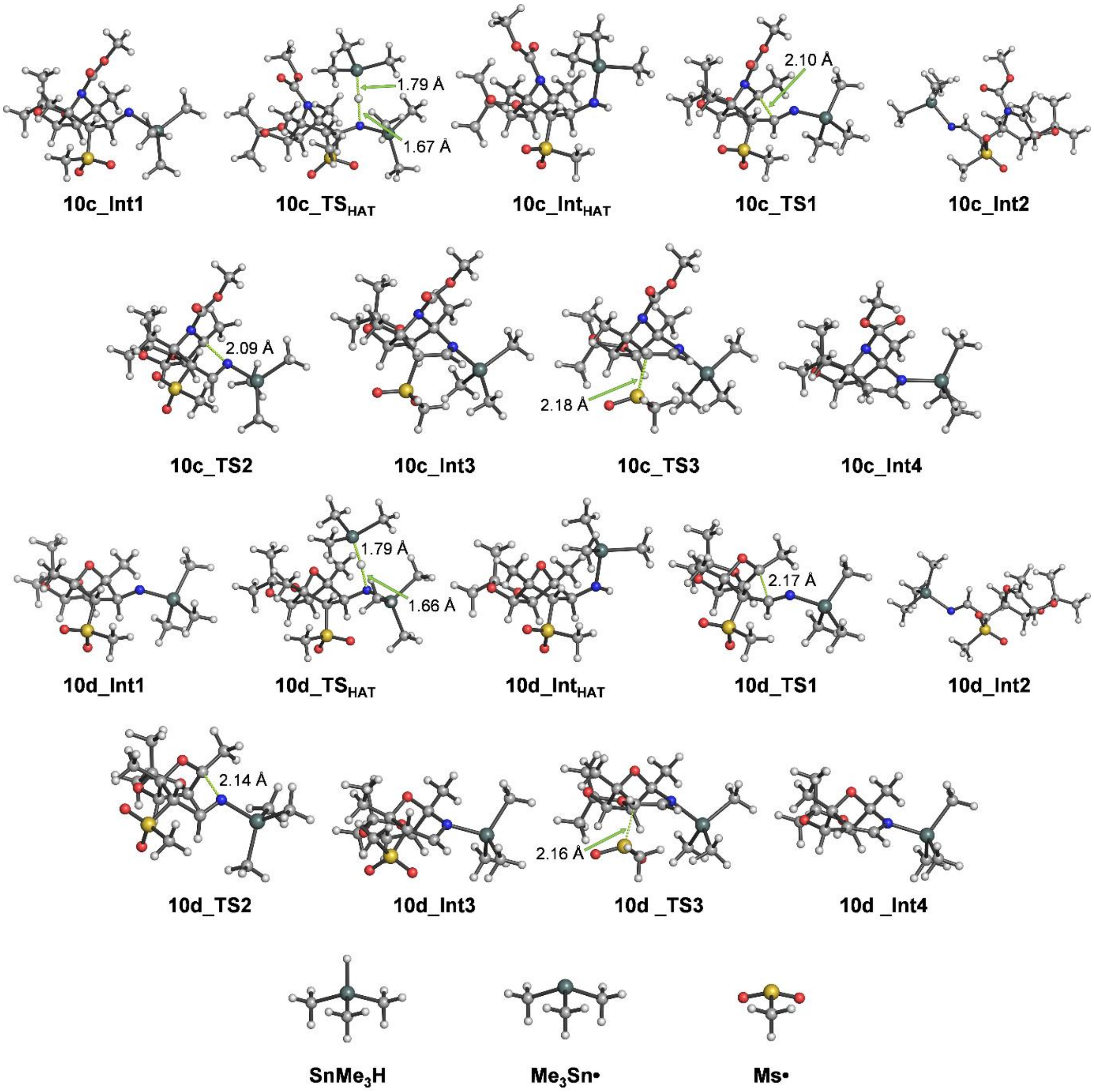

Figure S6. Geometries for the reactants, transition states, intermediates and products for the computed reaction pathways calculated with PCM(toluene)/M06-2X/6-31G(d,p)+LanL2DZ(Sn).

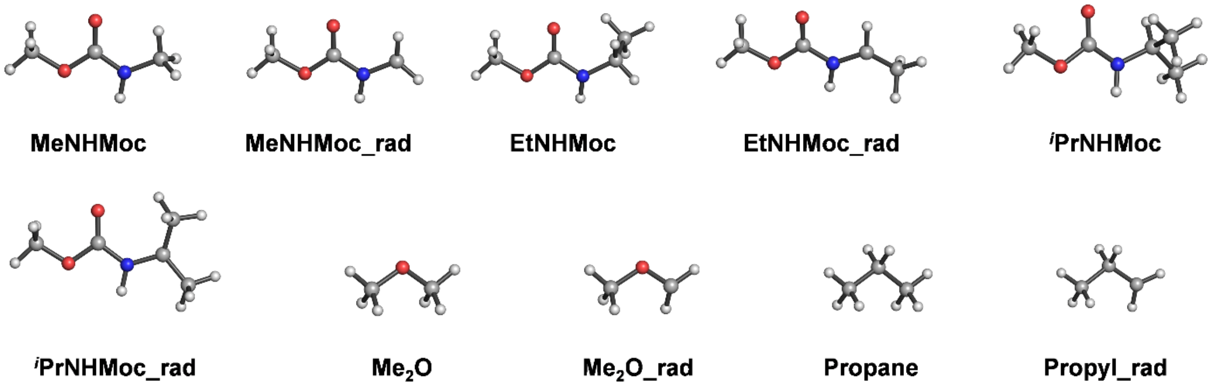

Figure S7. Geometries for neutral and radical species calculated with M06-2X/6-31G(d,p) for the estimation of the bond dissociation energies (BDE).

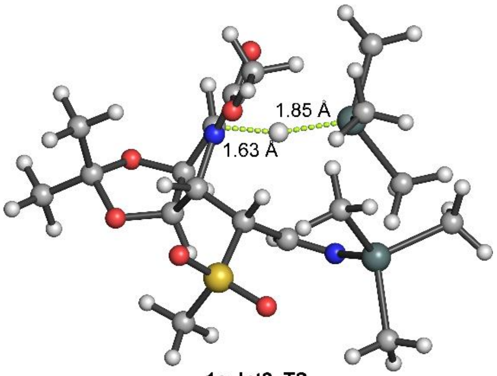

1a_Int2_TS$_{HAT}$

$\Delta G^{\ddagger}$ = 20.0 kcal mol^{-1}

Figure S8. Lowest-energy transition state calculated with PCM(toluene)/M06-2X/6-31G(d,p)+LanL2DZ(Sn) for the HAT reaction from intermediate **1a_Int2**.

Table S1. Energies, entropies, and lowest frequencies of the lowest energy calculated structures.[a]

Structure[b]	E_{elec} (Hartree)	ZPE (Hartree)	H (Hartree)	S (cal mol^{-1} K^{-1})	G (Hartree)	Lowest freq. (cm^{-1})	# imag. freq.
**1a_Int1**	–1549.665235	0.425211	–1549.193085	213.6	–1549.323691	14.2	0
**1a_Int2**	–1549.660593	0.422000	–1549.189979	219.3	–1549.324111	17.5	0
**1a_Int3**	–1549.688642	0.424827	–1549.216532	214.9	–1549.347936	30.8	0
**1a_Int4**	–961.340678	0.376207	–960.925484	185.6	–961.039004	37.4	0
**1a_Int_{HAT}**	–1550.337062	0.438529	–1549.851563	211.8	–1549.981061	37.7	0
**1a_TS1**	–1549.637764	0.421875	–1549.168611	215.2	–1549.300200	–448.9	1
**1a_TS2**	–1549.634191	0.421312	–1549.165418	215.3	–1549.297056	–584.2	1
**1a_TS3**	–1549.678970	0.423846	–1549.208159	213.8	–1549.338901	–150.3	1
**1a_TS_{HAT}**	–1673.286532	0.540273	–1672.685099	262.5	–1672.845620	–626.2	1
**1a_Int2_TS_{HAT}**	–1673.276207	0.538620	–1672.675306	265.1	–1672.837417	–1006.3	1
**10a_Int1**	–1341.718822	0.369116	–1341.309759	189.4	–1341.425584	36.9	0
**10a_Int2**	–1341.706778	0.365373	–1341.299334	197.9	–1341.420372	19.4	0
**10a_Int3**	–1341.739484	0.368570	–1341.330401	191.7	–1341.447614	23.3	0
**10a_Int4**	–753.389231	0.319497	–753.037213	163.6	–753.137244	16.6	0
**10a_Int_{HAT}**	–1342.389823	0.381953	–1341.967615	188.8	–1342.083060	34.8	0
**10a_TS1**	–1341.687784	0.365426	–1341.281693	192.9	–1341.399650	–438.1	1
**10a_TS2**	–1341.682700	0.365043	–1341.277178	191.3	–1341.394180	–572.1	1
**10a_TS3**	–1341.733335	0.367807	–1341.325684	189.2	–1341.441371	–168.4	1
**10a_TS_{HAT}**	–1465.339379	0.483564	–1464.801189	241.2	–1464.948677	–539.0	1
**10b_Int1**	–1305.831647	0.393125	–1305.397905	191.8	–1305.515217	25.9	0
**10b_Int2**	–1305.810856	0.388739	–1305.379319	200.4	–1305.501870	22.8	0
**10b_Int3**	–1305.851066	0.392526	–1305.417580	191.9	–1305.534928	24.7	0
**10b_Int4**	–717.498259	0.343737	–717.121670	163.9	–717.221926	20.0	0
**10b_Int_{HAT}**	–1306.499764	0.405942	–1306.052891	191.2	–1306.169797	28.6	0
**10b_TS1**	–1305.797327	0.389263	–1305.366946	193.5	–1305.485310	–536.1	1
**10b_TS2**	–1305.792846	0.388720	–1305.363043	192.7	–1305.480860	–548.1	1
**10b_TS3**	–1305.845832	0.392189	–1305.413377	190.1	–1305.529643	–154.6	1
**10b_TS_{HAT}**	–1429.451578	0.508242	–1428.888502	240.8	–1429.035740	–713.6	1
**10c_Int1**	–1628.262098	0.480625	–1627.729561	228.5	–1627.869286	16.4	0
**10c_Int2**	–1628.256592	0.478130	–1627.725146	234.1	–1627.868283	15.7	0
**10c_Int3**	–1628.283262	0.480923	–1627.750788	225.2	–1627.888520	30.2	0
**10c_Int4**	–1039.933542	0.432333	–1039.457797	197.5	–1039.578561	32.2	0
**10c_Int_{HAT}**	–1628.936853	0.495149	–1628.390444	223.1	–1628.526856	39.7	0
**10c_TS1**	–1628.240670	0.478006	–1627.710578	229.3	–1627.850827	–387.3	1
**10c_TS2**	–1628.231837	0.477936	–1627.702149	227.4	–1627.841214	–544.4	1
**10c_TS3**	–1628.277982	0.480248	–1627.746728	223.7	–1627.883509	–156.0	1
**10c_TS_{HAT}**	–1751.885233	0.596759	–1751.223245	273.0	–1751.390189	–552.8	1
**10d_Int1**	–1420.323581	0.423948	–1419.854466	205.9	–1419.980410	27.3	0
**10d_Int2**	–1420.311562	0.421667	–1419.843153	212.9	–1419.973364	18.1	0
**10d_Int3**	–1420.346288	0.425094	–1419.876763	202.3	–1420.000451	32.6	0
**10d_Int4**	–831.996617	0.375448	–831.584354	175.5	–831.691665	19.6	0
**10d_Int_{HAT}**	–1420.996920	0.437670	–1420.514188	203.0	–1420.638337	35.2	0
**10d_TS1**	–1420.301989	0.421469	–1419.835191	206.9	–1419.961726	–351.6	1
**10d_TS2**	–1420.289812	0.421020	–1419.823667	205.2	–1419.949182	–521.7	1
**10d_TS3**	–1420.342798	0.423613	–1419.874905	201.1	–1419.997865	–176.2	1
**10d_TS_{HAT}**	–1543.946665	0.539337	–1543.348125	253.3	–1543.503035	–601.9	1
**Ms·**	–588.340007	–588.294052	–588.285989	75.5	–588.332135	200.9	0
**$SnMe_3H$**	–123.618016	–123.501958	–123.487836	99.3	–123.548586	90.6	0
**$SnMe_3$·**	–122.986716	–122.878633	–122.864881	99.8	–122.925916	76.5	0

[a]Energy values calculated at the PCM(toluene)/M06-2X/6-31G(d,p)+LanL2DZ(Sn) level. 1 Hartree = 627.51 kcal mol^{-1}. Thermal corrections at 383.75 K.
[b]Except for **Int_{HAT}**, **Int4** and **$SnMe_3H$**, all species correspond to doublet spin states; all species are neutral

Table S2. Energies, enthalpies, free energies, and entropies of the QM structures calculated for the estimation of the BDE of various C–H bonds.[a]

Structure[b]	E_{elec}[c] (Hartree)	ZPE correction[d] (Hartree)	H correction[d] (Hartree)	S[d] (cal mol^{-1} K^{-1})	G correction[d] (Hartree)	Lowest freq.[d] (cm^{-1})
**H**	–0.499810	0.000000	0.002360	27.4	–0.010654	
**MeNHMoc**	–323.158690	0.109158	0.117459	84.2	0.077940	60.2
**MeNHMoc_rad**	–322.495210	0.094992	0.103067	82.7	0.063771	112.0
**Me_2O**	–154.727123	0.080930	0.086135	64.3	0.055602	216.8
**Me_2O_rad**	–154.061022	0.066901	0.072193	65.6	0.041011	180.6
**Propane**	–118.862186	0.104601	0.110048	65.4	0.078968	220.5
**Propyl_rad**	–118.187676	0.089235	0.095061	68.2	0.062670	142.4
**EtNHMoc**	–362.384877	0.138138	0.147454	89.4	0.105500	74.3
**EtNHMoc_rad**	–361.721858	0.123662	0.133234	91.5	0.090006	78.2
***^{i}*PrNHMoc**	–401.611532	0.166206	0.176867	95.9	0.132107	61.2
***^{i}*PrNHMoc_rad**	–400.946475	0.152248	0.163285	100.2	0.116874	31.1

[a]1 Hartree = 627.51 kcal mol^{-1}.
[b]Except for **MeNHMoc**, **Me_2O**, **Propane**, **EtNHMoc**, and **^{i}PrNHMoc**, all species correspond to doublet spin states; all species are neutral.
[c]Energy obtained from single-point calculations with SCS-MP2/cc-pVTZ.
[d]Vibrational, thermal and entropic corrections obtained from frequency calculations on geometries optimized at the M06-2X/6-31G(d,p) level. Thermal corrections at 383.75 K.

Table S3. Cartesian coordinates of the lowest energy structures calculated with PCM(toluene)/M06-2X/6-31G(d,p)+LanL2DZ(Sn)

Structure **1a_Int1**

C	2.390323	0.509459	0.122894
C	1.087627	0.489417	0.942617
C	0.391250	-0.136911	-1.071600
C	1.883637	0.169668	-1.311235
H	2.960611	1.438127	0.201325
H	2.045810	0.973325	-2.036829
C	0.162332	1.648215	0.512834
H	-0.672485	1.730176	1.215628
C	-0.363687	1.208133	-0.902408
H	-0.033102	1.921337	-1.670859
H	1.221389	0.379984	2.015840
H	-0.041812	-0.834654	-1.787643
N	-1.789592	1.206028	-0.816476
Sn	-2.899436	-0.456422	-0.250691
C	-2.502143	-1.014542	1.771290
H	-3.434127	-1.121857	2.330727
H	-1.974348	-1.971734	1.784976
H	-1.873091	-0.280156	2.279341
C	-2.546947	-2.061316	-1.601099
H	-2.471637	-1.697310	-2.628612
H	-1.627124	-2.589165	-1.339869
H	-3.381956	-2.765076	-1.553560
C	-4.875702	0.298798	-0.444657
H	-5.026681	1.152613	0.219805
H	-5.061179	0.628946	-1.469061
H	-5.614387	-0.465655	-0.191817
S	0.926567	3.274732	0.499249
C	-0.484053	4.345187	0.292269
H	-1.086048	3.968776	-0.537498
H	-1.058766	4.352816	1.218086
H	-0.089763	5.337700	0.070916
O	1.765728	3.394624	-0.697653
O	1.526634	3.494102	1.816069
C	0.554454	-1.939157	0.655834
O	0.364828	-2.881174	-0.085843
N	0.359153	-0.620777	0.313860
O	0.878068	-2.051747	1.951801
C	1.042008	-3.392734	2.411761
H	1.248072	-3.314294	3.477216
H	0.133282	-3.971408	2.236600
H	1.877739	-3.872081	1.897841
O	3.162890	-0.618600	0.473099
O	2.575626	-1.007825	-1.663517
C	3.637647	-1.194384	-0.737361
C	4.890653	-0.471765	-1.215878
H	5.692539	-0.586512	-0.483265
H	5.215796	-0.887241	-2.171911
H	4.691206	0.594869	-1.351926
C	3.842136	-2.677420	-0.522982
H	4.228627	-3.133924	-1.436480
H	4.559157	-2.838812	0.284710
H	2.884688	-3.139094	-0.274129

Structure **1a_Int2**

C	2.984731	-0.762288	-0.828989
C	1.531350	-0.262045	-0.675350
C	2.662602	0.160346	1.363785
C	3.664294	-0.598705	0.554755
H	3.021857	-1.783487	-1.211376
H	3.951179	-1.544563	1.014389
C	0.438816	-1.348948	-0.714073
H	0.642091	-1.978005	-1.592828
C	-0.937356	-0.722623	-0.901157
H	-0.956476	-0.119400	-1.828673
H	1.314505	0.428231	-1.497494
H	2.860480	0.716211	2.266480
N	-1.896458	-0.839081	-0.094318
Sn	-3.566753	0.318200	-0.432031
C	-5.244470	-0.978317	-0.330370
H	-5.254878	-1.513835	0.621116
H	-6.175532	-0.413757	-0.418374
H	-5.210853	-1.714359	-1.136060
C	-3.346378	1.239882	-2.338341
H	-3.237829	0.498436	-3.133288
H	-4.222779	1.852347	-2.562888
H	-2.466986	1.888595	-2.343251
C	-3.527947	1.759654	1.127127
H	-3.346158	1.276689	2.090446
H	-2.712494	2.457592	0.925766
H	-4.467267	2.313058	1.188586
N	1.556210	0.491162	0.582711
C	0.654550	1.496532	0.829507
O	-0.221843	1.836885	0.056328
S	0.519228	-2.591120	0.608070
C	-0.033529	-1.857647	2.134412
H	0.680169	-1.093587	2.441610
H	-1.034804	-1.463855	1.969383
H	-0.036673	-2.686084	2.845733
O	1.942351	-2.923521	0.763979
O	-0.411275	-3.655626	0.239058
O	0.875747	2.049241	2.030786
C	-0.017711	3.108496	2.380611
H	0.368449	3.522457	3.309723
H	-0.034251	3.867887	1.597510
H	-1.026629	2.718012	2.528048
O	3.718123	0.089508	-1.684153
O	4.860641	0.116971	0.271563
C	4.692692	0.823005	-0.942099
C	4.207407	2.248839	-0.701863
H	3.258032	2.251001	-0.162105
H	4.946209	2.796459	-0.112208
H	4.067232	2.758118	-1.658198
C	6.005001	0.782170	-1.700599
H	5.890296	1.267719	-2.672011
H	6.780236	1.301490	-1.133198
H	6.300328	-0.257761	-1.847755

Structure **1a_Int3**

C	2.400974	-0.232655	-0.403275
C	1.580313	0.426260	0.712885
C	0.127866	-1.126639	-0.230667
C	1.420107	-1.246829	-1.055370
H	2.825521	0.487260	-1.107864
H	1.229640	-1.065926	-2.117114
H	2.201369	0.727517	1.555163
H	-0.363964	-2.092645	-0.114040
C	0.714086	1.597748	0.197443
C	-0.273864	1.143687	-0.841369
N	-0.776740	-0.149842	-0.802176
H	-0.919236	1.890749	-1.297016
H	0.234274	2.044417	1.087209
N	0.649158	-0.653126	1.060056
C	-0.232642	-0.576842	2.101439
O	-1.280855	-1.196193	2.155615
O	0.196733	0.255722	3.062484
C	-0.664596	0.349508	4.199114
H	-0.175870	1.042177	4.881031
H	-1.644734	0.728413	3.902011
H	-0.785371	-0.628883	4.666286
S	1.772136	2.950112	-0.396446
O	2.221378	2.685752	-1.765075
O	2.766684	3.193014	0.652385
C	0.667906	4.351958	-0.455922
H	1.274682	5.195087	-0.789570
H	-0.126256	4.154390	-1.175958
H	0.271334	4.540351	0.541625
Sn	-2.824594	-0.405822	-0.593924
C	-3.602012	0.348902	-2.423693
H	-4.694559	0.329340	-2.418824
H	-3.284874	1.381653	-2.588221
H	-3.250171	-0.252473	-3.264441
C	-3.436890	0.815603	1.037067
H	-2.683567	1.592064	1.204799
H	-4.394759	1.301695	0.840564
H	-3.509238	0.211469	1.942309
C	-3.117766	-2.489744	-0.361229
H	-2.695009	-2.806585	0.593663
H	-4.182483	-2.733088	-0.375171
H	-2.631804	-3.040536	-1.169989
O	3.394002	-1.044388	0.186476
O	2.007711	-2.507094	-0.822798
C	3.365701	-2.307857	-0.453853
C	3.751734	-3.367098	0.554410
H	3.680745	-4.357901	0.101232
H	4.776295	-3.203042	0.894086
H	3.071404	-3.305294	1.405305
C	4.249323	-2.295166	-1.694970
H	5.286471	-2.098736	-1.414995
H	4.190876	-3.258106	-2.207070
H	3.921231	-1.512787	-2.385579

Structure **1a_Int4**

C	-2.607387	-0.109746	1.213624
C	-1.608487	1.045689	1.304387
C	-0.591921	-0.421000	-0.167914
C	-1.866958	-1.142304	0.308060
H	-2.872596	-0.500536	2.201835
H	-1.626124	-2.088936	0.801571
H	-2.083977	2.010497	1.478442
H	-0.378688	-0.665637	-1.209369
C	-0.544214	0.690650	2.320273
C	0.451135	-0.133905	1.941407
N	0.556646	-0.710702	0.676261
H	1.253488	-0.386474	2.634305
H	-0.608784	1.059711	3.336056
Sn	2.445161	-0.723098	-0.170438
C	2.160888	-1.182888	-2.218400
H	3.121354	-1.340891	-2.714250
H	1.565665	-2.092017	-2.332833
H	1.644816	-0.352480	-2.702879
C	3.429402	-2.295729	0.877330
H	4.472345	-2.382138	0.562122
H	3.419092	-2.113907	1.955128
H	2.933328	-3.250467	0.689805
C	3.398290	1.129768	0.258957
H	2.853670	1.632676	1.064023
H	4.433517	0.986121	0.575556
H	3.366936	1.774239	-0.620484
N	-0.999025	0.985112	-0.036729
C	-0.108658	1.920223	-0.478022
O	0.789790	1.686815	-1.271271
O	-0.350989	3.136462	0.032472
C	0.540383	4.156866	-0.415814
H	0.227235	5.066159	0.093562
H	1.570292	3.905352	-0.153726
H	0.467721	4.279825	-1.497891
O	-3.749803	0.249256	0.468345
O	-2.734221	-1.325971	-0.790532
C	-4.006586	-0.781962	-0.467563
C	-4.596268	-0.163724	-1.716127
H	-4.746118	-0.930808	-2.478630
H	-5.556575	0.301967	-1.485357
H	-3.904716	0.592894	-2.090261
C	-4.889222	-1.857279	0.155294
H	-5.854556	-1.433531	0.441003
H	-5.050081	-2.670117	-0.556259
H	-4.409264	-2.267912	1.048616

Structure **1a_Int**$_{HAT}$

C	2.326684	0.684008	0.181072
C	0.979513	0.484875	0.896607
C	0.512776	-0.148483	-1.182701
C	1.972557	0.336053	-1.296690
H	2.772155	1.671209	0.327284
H	2.088752	1.180610	-1.984131
C	-0.044625	1.532888	0.419439
H	-0.934952	1.486703	1.052302
C	-0.407902	1.093955	-1.034226
H	-0.092586	1.859849	-1.752445
H	1.045880	0.356781	1.974121
H	0.208409	-0.865845	-1.944256
N	-1.832887	0.882079	-1.147251
Sn	-2.849862	-0.626255	-0.208799

C	-2.512198	-0.534836	1.891843
H	-2.694160	0.471089	2.279439
H	-3.204009	-1.212205	2.399614
H	-1.492690	-0.835513	2.144880
C	-2.427979	-2.542710	-1.031882
H	-2.716313	-2.566031	-2.086124
H	-1.370703	-2.804689	-0.955281
H	-3.011499	-3.305868	-0.509427
C	-4.869337	-0.106137	-0.645095
H	-5.130704	0.847421	-0.180792
H	-5.026774	-0.013586	-1.722718
H	-5.557107	-0.868175	-0.270407
S	0.516204	3.233536	0.524472
C	-1.001537	4.137541	0.280103
H	-1.507745	3.727326	-0.596138
H	-1.620658	4.031165	1.170589
H	-0.723521	5.179973	0.120837
O	1.397688	3.514529	-0.615087
O	1.015251	3.466594	1.881079
C	0.816883	-1.971381	0.499301
O	0.873071	-2.891228	-0.289250
N	0.433531	-0.684386	0.186168
O	1.011943	-2.095634	1.821373
C	1.387921	-3.404234	2.249137
H	1.434927	-3.356346	3.335153
H	0.649499	-4.139430	1.925917
H	2.364844	-3.669969	1.839539
H	-2.124217	0.979873	-2.115237
O	2.834001	-0.731738	-1.626593
O	3.207327	-0.354163	0.557794
C	3.844439	-0.807818	-0.630323
C	4.245818	-2.254540	-0.448541
H	4.759373	-2.610859	-1.343904
H	4.920208	-2.345680	0.405681
H	3.350805	-2.858277	-0.289298
C	5.013609	0.101887	-0.989001
H	5.768117	0.070901	-0.199874
H	5.463438	-0.226564	-1.928239
H	4.676582	1.135163	-1.109911

Structure **1a_TS1**

C	2.695432	0.233491	-0.218873
C	1.855751	0.339713	1.057725
C	0.660824	-1.012446	-0.267620
C	1.786635	-0.585622	-1.179863
H	3.017066	1.199015	-0.615771
H	1.444179	-0.029961	-2.058219
C	0.692774	1.362065	0.913114
H	0.229631	1.484078	1.897186
C	-0.384344	0.811685	-0.039468
H	-0.257577	1.092142	-1.098110
H	2.444286	0.489669	1.960115
H	-0.030638	-1.806899	-0.522301
N	-1.530037	0.491604	0.505742
Sn	-3.022461	-0.217402	-0.698635
C	-3.361417	-2.206850	-0.023676
H	-3.809121	-2.195947	0.973002
H	-4.032859	-2.743450	-0.697637
H	-2.415244	-2.751047	0.033865
C	-2.336009	-0.188209	-2.718996
H	-1.428352	-0.786972	-2.835259
H	-3.097972	-0.602164	-3.383683
H	-2.116775	0.830149	-3.049222
C	-4.726009	1.021491	-0.416209
H	-4.970935	1.095307	0.645403
H	-4.541115	2.028254	-0.796813
H	-5.592202	0.611681	-0.940683
N	1.154688	-0.948119	1.040634
C	0.391180	-1.302811	2.143921
O	0.516213	-0.795494	3.235281
S	1.271430	2.985535	0.410620
C	-0.089618	4.054354	0.837062
H	-0.992900	3.674769	0.357582
H	-0.196983	4.076359	1.921228
H	0.164206	5.043156	0.452842
O	2.430196	3.330325	1.236191
O	1.412460	2.993612	-1.050762
O	-0.458945	-2.289128	1.841579
C	-1.340919	-2.650967	2.906960
H	-1.950178	-3.465435	2.520024
H	-0.770523	-2.973570	3.779063
H	-1.966186	-1.796810	3.175470
O	3.783666	-0.627550	0.022148
O	2.573040	-1.699397	-1.560583
C	3.903533	-1.502691	-1.086152
C	4.444133	-2.826255	-0.592879
H	4.482305	-3.542784	-1.415726
H	5.450092	-2.691977	-0.190239
H	3.785982	-3.204892	0.190889
C	4.748363	-0.865751	-2.181485
H	5.759150	-0.676759	-1.813610
H	4.798852	-1.528813	-3.047761
H	4.306544	0.083928	-2.496637

Structure **1a_TS2**

C	1.868981	-0.180528	-1.062567
C	1.842116	0.425994	0.347081
C	0.216002	-1.236140	0.334188
C	0.662537	-1.155441	-1.105889
H	1.854986	0.584241	-1.844619
H	-0.141116	-0.818196	-1.769225
C	0.921047	1.671178	0.459151
H	0.878788	1.923053	1.526109
C	-0.459799	1.369949	-0.095651
H	-0.735032	1.861047	-1.039410
H	2.840649	0.653845	0.721191
H	-0.304110	-2.094139	0.739112
N	-1.097864	0.373384	0.442593
Sn	-2.916744	-0.190242	-0.355090
C	-2.822513	-2.300615	-0.591582
H	-2.725356	-2.793862	0.378466
H	-3.733989	-2.668871	-1.068302
H	-1.973195	-2.594527	-1.214062
C	-3.135980	0.799881	-2.227958
H	-2.285147	0.593453	-2.882302
H	-4.040756	0.451380	-2.731486

H	-3.213810	1.881979	-2.100880
C	-4.380646	0.396929	1.064765
H	-4.162863	-0.047360	2.038161
H	-4.394299	1.482850	1.178619
H	-5.375616	0.071656	0.752601
N	1.220600	-0.649697	1.120396
C	1.215302	-0.593305	2.489764
O	1.852447	0.212863	3.139501
S	1.683582	3.087708	-0.350528
C	0.799468	4.473278	0.342390
H	-0.267444	4.332307	0.163985
H	1.020713	4.538542	1.407387
H	1.159639	5.359584	-0.181539
O	1.374156	3.032063	-1.783748
O	3.081067	3.156640	0.077513
C	0.421279	-1.585229	4.440805
H	-0.235754	-2.409878	4.709727
H	1.424204	-1.748529	4.838975
H	0.031933	-0.642169	4.828377
O	0.448866	-1.563221	3.013108
O	2.989570	-1.023081	-1.216914
O	1.188170	-2.345823	-1.650052
C	2.593603	-2.375235	-1.436732
C	2.944293	-3.222313	-0.221391
H	4.024007	-3.199926	-0.056611
H	2.444419	-2.835080	0.668343
H	2.632016	-4.255728	-0.388959
C	3.250155	-2.878549	-2.708429
H	4.336061	-2.871696	-2.594074
H	2.921296	-3.898004	-2.921697
H	2.971228	-2.227667	-3.538812

Structure **1a_TS3**

C	2.156939	0.579374	-0.345960
C	1.781827	0.118043	1.071271
C	0.284387	-0.983902	-0.288673
C	1.058237	-0.052991	-1.246057
H	2.273720	1.659083	-0.452168
H	0.403843	0.685975	-1.719173
H	2.653655	0.064518	1.720735
H	-0.010536	-1.915940	-0.774621
C	0.630407	0.915936	1.717208
C	-0.662132	0.457384	1.376009
N	-0.892581	-0.335412	0.298406
H	-1.533106	0.800611	1.927933
H	0.781478	1.206294	2.755498
N	1.263330	-1.221975	0.757006
C	0.975924	-2.164172	1.714217
O	0.198980	-3.082770	1.562246
O	1.698061	-1.959812	2.829186
C	1.513188	-2.949413	3.841492
H	2.150781	-2.646967	4.669833
H	0.467804	-2.984649	4.153314
H	1.807649	-3.932871	3.471055
S	0.666363	2.955970	0.896653
O	-0.126644	2.870940	-0.354458
O	1.947762	3.700611	0.855273
C	-0.375127	3.808250	2.092549
H	-0.568960	4.801611	1.685203
H	-1.309134	3.257561	2.205886
H	0.160563	3.880497	3.039038
Sn	-2.719764	-0.381081	-0.679863
C	-4.094736	0.165266	0.839946
H	-3.975059	1.216512	1.111800
H	-5.120817	0.021047	0.494807
H	-3.944263	-0.448822	1.730610
C	-2.563672	1.056248	-2.230767
H	-3.548459	1.400167	-2.554272
H	-1.991935	1.908054	-1.851707
H	-2.040297	0.641366	-3.095127
C	-2.848051	-2.400086	-1.314146
H	-2.413581	-3.044602	-0.545547
H	-3.886529	-2.698181	-1.471604
H	-2.302215	-2.554977	-2.247215
O	3.334189	-0.090662	-0.740063
O	1.764641	-0.830224	-2.186110
C	3.154837	-0.540466	-2.070331
C	3.934016	-1.824503	-2.250963
H	3.759189	-2.229789	-3.249654
H	5.001888	-1.634028	-2.126079
H	3.602322	-2.544852	-1.501146
C	3.536217	0.551746	-3.061107
H	4.592437	0.806947	-2.951037
H	3.353249	0.211031	-4.082538
H	2.938005	1.449722	-2.881754

Structure **1a_TS$_{HAT}$**

C	-3.041032	-0.348355	0.436699
C	-1.795404	0.354039	1.004446
C	-0.974765	-0.693061	-0.774053
C	-2.446636	-1.161809	-0.752255
H	-3.597638	-0.941018	1.166685
H	-2.547486	-2.246389	-0.637609
C	-0.821585	-0.681629	1.608972
H	-0.019210	-0.166232	2.142460
C	-0.218933	-1.425638	0.368489
H	-0.539629	-2.481668	0.371423
H	-2.001477	1.217385	1.632824
H	-0.521066	-0.729287	-1.764010
N	1.217090	-1.431126	0.410187
Sn	2.278263	-1.735943	-1.332362
C	2.228157	0.009254	-2.557126
H	2.439873	-0.246928	-3.598441
H	1.254868	0.508369	-2.518555
H	2.986705	0.723831	-2.224277
C	1.369125	-3.397659	-2.310099
H	1.358917	-4.274516	-1.658932
H	0.337791	-3.167457	-2.589988
H	1.915041	-3.654714	-3.220888
C	4.245110	-2.126265	-0.637575
H	4.575884	-1.309520	0.009536
H	4.273462	-3.051241	-0.058180
H	4.951095	-2.214383	-1.466165
S	-1.557538	-1.789292	2.810924
C	-0.118131	-2.544023	3.544268
H	0.542636	-2.875544	2.740313

H	0.380335	-1.807997	4.174501
H	-0.473901	-3.388096	4.136109
O	-2.302105	-2.834756	2.097769
O	-2.254752	-0.979238	3.812032
C	-1.278469	1.757343	-1.020858
O	-1.071582	1.791572	-2.217844
N	-1.046001	0.664655	-0.222801
O	-1.648979	2.818009	-0.287311
C	-1.849211	4.020635	-1.030642
H	-2.092519	4.782789	-0.293062
H	-0.942517	4.287551	-1.576598
H	-2.673597	3.898716	-1.736169
H	1.658671	0.161552	0.553849
Sn	1.985915	1.897278	0.864226
C	0.621536	2.594610	2.358378
H	1.016653	3.507027	2.813393
H	-0.337254	2.834841	1.892435
H	0.455678	1.870075	3.159619
C	1.801188	3.146164	-0.861081
H	2.760637	3.216371	-1.379556
H	1.058316	2.776471	-1.570660
H	1.511750	4.151892	-0.542083
C	3.995691	2.031247	1.589463
H	4.137740	1.403939	2.472181
H	4.704404	1.706698	0.823265
H	4.237238	3.062486	1.859805
O	-3.857140	0.612104	-0.199755
O	-3.134916	-0.687085	-1.887435
C	-4.261865	0.058173	-1.444778
C	-5.463030	-0.859997	-1.254881
H	-6.314792	-0.289219	-0.878504
H	-5.732362	-1.320189	-2.207797
H	-5.231349	-1.654456	-0.539969
C	-4.514825	1.184802	-2.421235
H	-4.850668	0.778105	-3.377310
H	-5.287071	1.848957	-2.027566
H	-3.586777	1.737197	-2.578223

Structure **1a_Int2_TS_{HAT}**

C	-2.656200	-0.968100	-0.957300
C	-1.782000	-0.960700	0.323100
C	-2.537600	1.290900	-0.080100
C	-3.142600	0.487600	-1.194100
H	-2.110800	-1.364800	-1.815500
H	-2.866200	0.880300	-2.178000
C	-0.306900	-1.316100	0.084300
H	0.249400	-1.116700	1.008700
C	0.363000	-0.594300	-1.084800
H	-0.347200	-0.168700	-1.817200
H	-2.197400	-1.670100	1.046200
H	-3.079000	2.124000	0.355600
N	1.596700	-0.442000	-1.277100
Sn	3.222200	-1.011200	-0.128800
C	4.390400	0.771100	-0.257000
H	4.624300	1.169400	0.733300
H	5.330200	0.580400	-0.781100
H	3.836700	1.530800	-0.815400
C	4.214200	-2.569500	-1.168100
H	4.125000	-2.405000	-2.244600
H	5.276100	-2.579600	-0.911700
H	3.771500	-3.534700	-0.924100
C	2.762500	-1.420800	1.915100
H	2.172400	-0.614700	2.361100
H	2.223700	-2.364400	2.017000
H	3.696900	-1.492000	2.479100
N	-1.927700	0.406300	0.832400
C	-1.436600	0.868900	2.022000
O	-1.522700	2.025300	2.386600
S	-0.078100	-3.118100	-0.003100
C	-0.969100	-3.707500	-1.429600
H	-2.040900	-3.574500	-1.273100
H	-0.609800	-3.182200	-2.316100
H	-0.724300	-4.768900	-1.499600
O	-0.694600	-3.688500	1.193800
O	1.350200	-3.342500	-0.253100
O	-0.854400	-0.118700	2.727500
C	-0.297600	0.288600	3.980200
H	0.101200	-0.618600	4.429600
H	0.497800	1.019400	3.817600
H	-1.069600	0.728200	4.613100
O	-3.831400	-1.725600	-0.812600
O	-4.558500	0.383400	-1.173600
C	-4.927500	-0.841400	-0.563200
C	-5.151200	-0.671300	0.934500
H	-4.260200	-0.267200	1.420500
H	-5.980300	0.019000	1.105100
H	-5.392300	-1.636500	1.385600
C	-6.146400	-1.386600	-1.277100
H	-6.415300	-2.361300	-0.864700
H	-6.987500	-0.702700	-1.148100
H	-5.923800	-1.489900	-2.340100
Sn	0.354700	3.016400	-0.508700
H	-1.252100	2.118500	-0.655300
C	-0.087300	5.056300	-0.035100
H	-0.615200	5.548100	-0.855000
H	-0.715400	5.102900	0.857600
H	0.832100	5.613200	0.163300
C	1.545200	2.910400	-2.282400
H	2.480000	3.460500	-2.139800
H	1.782600	1.866000	-2.498800
H	1.021100	3.345500	-3.136200
C	1.405400	2.162000	1.158800
H	1.204700	1.087900	1.226500
H	2.484700	2.306100	1.071400
H	1.050900	2.632900	2.078200

Structure **10a_Int1**

C	2.408533	0.034230	0.375675
C	1.027763	0.228903	1.012681
C	0.487467	-0.668960	-0.864113
C	2.009836	-0.548851	-1.008933
H	3.026933	0.933786	0.334555
H	2.323499	0.066213	-1.858232
C	0.252241	1.375326	0.313701
H	-0.619467	1.652774	0.914753

C	-0.190233	0.711231	-1.035776
H	0.226727	1.253539	-1.895725
H	1.023944	0.266212	2.100459
H	0.047309	-1.476732	-1.449752
N	-1.621008	0.732747	-1.063342
Sn	-2.738174	-0.642576	0.023660
C	-4.699193	-0.245154	-0.692442
H	-4.768406	-0.458063	-1.761548
H	-5.438593	-0.859815	-0.173454
H	-4.957036	0.804969	-0.538490
C	-2.559687	-0.126735	2.083360
H	-1.571975	-0.410398	2.452902
H	-2.707507	0.944152	2.242904
H	-3.312926	-0.658831	2.669449
C	-2.206211	-2.671064	-0.340139
H	-2.130336	-2.882368	-1.409289
H	-1.251699	-2.899275	0.137610
H	-2.974536	-3.327266	0.076778
S	1.162539	2.904993	0.075008
C	-0.118616	4.031510	-0.442753
H	-0.670895	3.576845	-1.267342
H	-0.774573	4.233786	0.403571
H	0.385456	4.942108	-0.768960
O	2.093721	2.734233	-1.044900
O	1.683023	3.319319	1.378840
O	0.310761	-0.911529	0.537318
O	3.064081	-1.029456	1.023938
O	2.588649	-1.833854	-1.035614
C	3.522627	-1.935356	0.032268
C	3.457692	-3.336809	0.597320
H	2.433928	-3.536965	0.917697
H	3.750224	-4.060790	-0.165825
H	4.131953	-3.427166	1.451322
C	4.909409	-1.531422	-0.450384
H	5.621477	-1.570192	0.376743
H	5.241581	-2.205707	-1.242708
H	4.888414	-0.511956	-0.846590

Structure **10a_Int2**

C	-2.911725	0.015211	-0.740236
C	-1.436554	-0.060789	-0.298249
C	-2.656349	0.294482	1.610818
C	-3.713682	0.275122	0.560113
H	-3.073834	0.773020	-1.507604
H	-4.303114	1.193285	0.536322
C	-0.529229	1.043955	-0.827195
H	-0.707066	1.178006	-1.904022
C	0.960782	0.765122	-0.631975
H	1.600197	1.613826	-0.935681
H	-1.000780	-1.007099	-0.630699
H	-2.811092	0.105371	2.664916
N	1.367675	-0.344790	-0.195102
Sn	3.385709	-0.710076	0.010240
C	3.667177	-1.165653	2.063879
H	2.995417	-1.970328	2.369126
H	4.695062	-1.483390	2.252302
H	3.459394	-0.291445	2.684451
C	4.437449	1.047097	-0.574461
H	4.161138	1.899871	0.050273
H	5.513586	0.888462	-0.472118
H	4.230865	1.303697	-1.616042
C	3.797608	-2.358574	-1.261026
H	3.145887	-3.199719	-1.016547
H	3.633897	-2.088870	-2.306360
H	4.834724	-2.682249	-1.148634
S	-0.923940	2.704889	-0.219192
C	-0.348860	2.790720	1.466131
H	-0.979534	2.161270	2.090028
H	0.695206	2.478387	1.503684
H	-0.438728	3.842470	1.742543
O	-2.381515	2.855878	-0.223577
O	-0.100468	3.623228	-1.009788
O	-1.425303	-0.068768	1.136249
O	-3.396454	-1.221372	-1.217866
O	-4.627020	-0.816824	0.631453
C	-4.132498	-1.865804	-0.180813
C	-3.231652	-2.808950	0.611744
H	-3.802290	-3.266772	1.423213
H	-2.854118	-3.596609	-0.044854
H	-2.381889	-2.273879	1.041568
C	-5.310901	-2.581125	-0.808975
H	-4.956325	-3.354920	-1.493127
H	-5.921284	-3.046533	-0.032294
H	-5.914787	-1.858741	-1.359954

Structure **10a_Int3**

C	-2.086335	-0.481570	0.171139
C	-1.816805	0.359825	-1.084442
C	0.024361	-0.902583	-0.930403
C	-0.818057	-1.365888	0.275075
H	-2.297853	0.108393	1.066851
H	-0.279185	-1.267925	1.224307
H	-2.731806	0.551762	-1.644741
H	0.580654	-1.726363	-1.385042
C	-1.040543	1.660091	-0.805526
C	0.294294	1.360093	-0.199393
N	0.919680	0.177465	-0.570233
H	0.954850	2.191746	0.033513
H	-0.974169	2.199590	-1.765544
S	-2.027290	2.780521	0.224882
O	-1.946481	2.389179	1.634522
O	-3.341781	2.889546	-0.414981
C	-1.192148	4.349525	0.058813
H	-1.773578	5.061012	0.646980
H	-0.183337	4.264547	0.462914
H	-1.181830	4.642082	-0.990916
Sn	2.823184	-0.228024	0.100927
C	3.785563	1.661407	0.036318
H	3.451682	2.298418	0.858349
H	4.868289	1.544291	0.117321
H	3.564665	2.164557	-0.907644
C	2.677628	-1.004246	2.074227
H	3.664063	-1.063436	2.540095
H	2.045806	-0.356681	2.686885
H	2.241754	-2.005933	2.067047
C	3.574112	-1.648822	-1.284084

H	3.365422	-1.325764	-2.306166
H	4.654959	-1.759078	-1.171733
H	3.117262	-2.628843	-1.129759
O	-0.947449	-0.455959	-1.862884
O	-3.122915	-1.397941	-0.121170
O	-1.277460	-2.680085	0.057208
C	-2.686706	-2.694502	0.244132
C	-3.018500	-2.974265	1.704706
H	-4.099667	-2.944110	1.855691
H	-2.642145	-3.957776	1.994682
H	-2.556908	-2.219417	2.348042
C	-3.289665	-3.694780	-0.717130
H	-2.922634	-4.698268	-0.492150
H	-4.377968	-3.685805	-0.630379
H	-3.003155	-3.420053	-1.733567

Structure **10a_Int4**

C	-2.559097	1.225249	0.483200
C	-1.662543	1.820599	-0.607622
C	-0.590337	-0.073647	-0.125166
C	-1.741594	-0.022639	0.907831
H	-2.752251	1.931325	1.295847
H	-1.359096	0.000587	1.930753
H	-2.219081	2.377969	-1.363177
H	-0.409310	-1.104578	-0.444153
C	-0.546138	2.607256	0.032514
C	0.528572	1.922234	0.458815
N	0.625150	0.533975	0.383932
H	1.388000	2.429692	0.894887
H	-0.621660	3.679516	0.159194
Sn	2.444398	-0.334796	0.005957
C	2.167716	-2.375731	0.522292
H	3.129930	-2.886710	0.601899
H	1.655756	-2.454310	1.483849
H	1.570640	-2.897025	-0.229358
C	3.791196	0.693490	1.286652
H	4.743916	0.162756	1.347692
H	3.989984	1.703371	0.921150
H	3.374148	0.764942	2.293612
C	2.929579	-0.088998	-2.049569
H	3.049842	0.969729	-2.289000
H	3.858558	-0.608438	-2.296320
H	2.130960	-0.490328	-2.677534
O	-1.103954	0.656901	-1.234547
O	-3.779928	0.711326	-0.003025
O	-2.637045	-1.101036	0.770239
C	-3.777646	-0.709452	0.014185
C	-5.017401	-1.190573	0.747255
H	-5.915040	-0.866541	0.216149
H	-5.016786	-2.281111	0.810411
H	-5.024265	-0.771745	1.755357
C	-3.688141	-1.250453	-1.405437
H	-3.642194	-2.342089	-1.384587
H	-4.570321	-0.944841	-1.973504
H	-2.795260	-0.851772	-1.889585

Structure **10a_Int$_{HAT}$**

C	2.432386	0.086647	0.372433
C	1.039941	0.221097	0.999083
C	0.552029	-0.688183	-0.888833
C	2.070273	-0.513130	-1.014756
H	3.009720	1.013575	0.335671
H	2.371127	0.112192	-1.861588
C	0.216750	1.326693	0.296413
H	-0.678085	1.552148	0.883429
C	-0.186870	0.660924	-1.053094
H	0.217942	1.221590	-1.903559
H	1.028702	0.251845	2.087083
H	0.142592	-1.508591	-1.480009
N	-1.629292	0.571566	-1.151434
Sn	-2.718465	-0.684376	0.044862
C	-2.524128	-0.037549	2.062258
H	-2.716574	1.034777	2.150678
H	-3.248332	-0.559469	2.692760
H	-1.524760	-0.253300	2.446670
C	-2.215289	-2.740346	-0.196467
H	-2.220753	-3.023762	-1.252141
H	-1.223699	-2.935665	0.216312
H	-2.941725	-3.370145	0.323662
C	-4.697279	-0.339298	-0.664076
H	-4.966873	0.713612	-0.555086
H	-4.789474	-0.606876	-1.719738
H	-5.420606	-0.936182	-0.103238
S	1.053338	2.896934	0.088430
C	-0.282968	3.975781	-0.391760
H	-0.830021	3.504765	-1.210451
H	-0.932700	4.136220	0.468123
H	0.173042	4.912278	-0.715062
O	1.979332	2.795235	-1.046066
O	1.572864	3.314816	1.392205
H	-1.910507	0.500405	-2.124888
O	0.372550	-0.946799	0.513257
O	3.133178	-0.945854	1.025341
O	2.699966	-1.774983	-1.033277
C	3.632720	-1.833850	0.038091
C	5.003548	-1.373414	-0.440317
H	5.713491	-1.379754	0.389554
H	5.367195	-2.034905	-1.229680
H	4.940858	-0.356643	-0.839067
C	3.624577	-3.235373	0.606978
H	2.608703	-3.476424	0.924175
H	3.949687	-3.949003	-0.152775
H	4.298663	-3.295132	1.463853

Structure **10a_TS1**

C	-2.558293	0.205450	0.362920
C	-1.998532	0.732688	-0.970011
C	-0.932351	-1.140844	-0.705085
C	-1.758685	-1.109973	0.562233
H	-2.462536	0.923532	1.180331
H	-1.157899	-1.155210	1.473240
C	-0.644322	1.448902	-0.810344
H	-0.290097	1.748137	-1.801269
C	0.384965	0.481969	-0.219692
H	0.260033	0.314491	0.863388
N	1.524557	0.341316	-0.839285

Sn	2.983372	-0.777966	0.060819
C	3.441567	-2.355521	-1.287801
H	3.754134	-1.954717	-2.254120
H	4.248312	-2.978693	-0.895161
H	2.566919	-2.990180	-1.448001
C	2.212176	-1.535504	1.900670
H	1.367870	-2.207635	1.724637
H	2.984381	-2.099817	2.429091
H	1.877837	-0.727869	2.556909
C	4.640055	0.517095	0.374560
H	4.943835	0.984274	-0.564515
H	4.382327	1.306391	1.084301
H	5.492798	-0.036094	0.774710
S	-0.772479	2.955617	0.160320
C	0.748890	3.802922	-0.218544
H	1.579755	3.119521	-0.036056
H	0.724960	4.122508	-1.260066
H	0.796254	4.662901	0.450794
O	-1.900514	3.728710	-0.362436
O	-0.750015	2.598538	1.583199
O	-1.609838	-0.455167	-1.672923
H	-0.401333	-2.020086	-1.055586
H	-2.727677	1.292189	-1.553633
O	-3.897564	-0.202488	0.234014
O	-2.736545	-2.126031	0.618382
C	-4.004809	-1.622877	0.211013
C	-5.034809	-2.044333	1.242717
H	-6.012100	-1.632488	0.982305
H	-5.105623	-3.133586	1.276753
H	-4.736154	-1.670519	2.223652
C	-4.347375	-2.102703	-1.191073
H	-4.405716	-3.193536	-1.204013
H	-5.313584	-1.692860	-1.494165
H	-3.582605	-1.773574	-1.895847

Structure **10a_TS2**

C	-1.946771	-0.425190	0.425624
C	-1.865131	0.400259	-0.867663
C	-0.280367	-1.178615	-1.109222
C	-0.789901	-1.449950	0.283058
H	-1.889294	0.202434	1.319149
H	-0.014866	-1.343607	1.049573
C	-0.975765	1.659362	-0.731090
H	-0.829524	2.050560	-1.744413
C	0.353390	1.284092	-0.102204
H	0.510425	1.576062	0.946803
N	1.063905	0.426233	-0.761333
Sn	2.797379	-0.311932	0.079927
C	2.741517	-2.409529	-0.256144
H	2.708898	-2.622738	-1.327254
H	3.633279	-2.886846	0.156550
H	1.866025	-2.866503	0.212396
C	2.769161	0.166374	2.155539
H	1.860990	-0.209221	2.633705
H	3.626917	-0.288180	2.656715
H	2.816374	1.246063	2.314899
C	4.393588	0.625988	-0.957721
H	4.300342	0.450960	-2.031490
H	4.387868	1.704018	-0.784054
H	5.356338	0.230086	-0.626857
S	-1.838098	2.945363	0.183953
C	-0.853084	4.398426	-0.131913
H	0.172193	4.203812	0.185278
H	-0.903024	4.638975	-1.193584
H	-1.290927	5.199306	0.465428
O	-1.743724	2.644092	1.616366
O	-3.155878	3.127186	-0.426784
O	-1.218030	-0.458206	-1.812061
H	0.231191	-1.925810	-1.705023
H	-2.853449	0.658698	-1.252349
O	-3.112125	-1.215447	0.480140
O	-1.403712	-2.709941	0.474651
C	-2.801495	-2.587050	0.248770
C	-3.522067	-3.424423	1.287315
H	-4.601788	-3.303974	1.177764
H	-3.267590	-4.478719	1.159011
H	-3.221568	-3.097684	2.284094
C	-3.166168	-2.979848	-1.176785
H	-2.883587	-4.019688	-1.357147
H	-4.244158	-2.875573	-1.321311
H	-2.651447	-2.336148	-1.892040

Structure **10a_TS3**

C	-2.234970	0.087473	-0.167049
C	-1.714335	0.570193	-1.529595
C	-0.284211	-1.053939	-1.006566
C	-1.180700	-0.969765	0.246754
H	-2.386726	0.862992	0.583806
H	-0.631285	-0.707234	1.157152
C	-0.619499	1.642536	-1.472491
C	0.683565	1.101978	-1.321752
N	0.883786	-0.180251	-0.920065
H	1.553789	1.743869	-1.419432
H	-0.708779	2.463836	-2.181398
S	-0.754855	2.881777	0.255533
O	0.216784	3.943106	-0.073974
O	-0.453245	2.015927	1.421509
C	-2.307442	3.735334	0.633810
H	-2.110883	4.360741	1.506990
H	-2.574760	4.352501	-0.223654
H	-3.087163	3.006034	0.852915
Sn	2.552930	-0.689755	0.208223
C	2.054978	-0.260832	2.223889
H	2.950604	-0.045545	2.810812
H	1.397670	0.613467	2.239029
H	1.533418	-1.103020	2.684439
C	4.067381	0.561484	-0.587079
H	3.915939	1.597844	-0.278006
H	5.050484	0.241672	-0.234683
H	4.062455	0.516803	-1.678364
C	2.811709	-2.755405	-0.205053
H	2.835857	-2.926775	-1.283501
H	3.750434	-3.116398	0.221022
H	1.998188	-3.346033	0.222675
O	-1.121952	-0.622254	-2.054695
H	0.031560	-2.080691	-1.208817

H	-2.533917	0.859734	-2.188146
O	-3.408348	-0.667269	-0.380703
O	-1.906900	-2.171695	0.381429
C	-3.297136	-1.870706	0.359375
C	-3.805294	-1.670926	1.781541
H	-4.862972	-1.398534	1.768800
H	-3.679026	-2.590467	2.357114
H	-3.242160	-0.873157	2.274808
C	-4.017383	-2.965176	-0.396574
H	-3.596015	-3.030438	-1.401133
H	-3.889813	-3.920253	0.116909
H	-5.082925	-2.735910	-0.462337

Structure **10a_TS$_{HAT}$**

C	-3.111081	-0.105420	-0.013665
C	-2.037077	0.506335	0.897058
C	-0.853390	-0.087223	-0.800389
C	-2.252033	-0.541339	-1.237772
H	-3.695704	-0.907736	0.442901
H	-2.313663	-1.609267	-1.474513
C	-1.118420	-0.574918	1.500929
H	-0.430769	-0.113808	2.216576
C	-0.306576	-1.078690	0.270627
H	-0.633745	-2.096636	-0.002016
H	-2.406542	1.251803	1.598929
H	-0.172974	0.145707	-1.622735
N	1.104368	-1.125009	0.540610
Sn	2.344837	-1.634517	-1.026214
C	3.056872	0.115747	-2.010809
H	3.588599	0.769454	-1.313831
H	3.744685	-0.153432	-2.816353
H	2.228034	0.679859	-2.446707
C	1.174745	-2.802061	-2.376200
H	0.742766	-3.672407	-1.876406
H	0.357198	-2.208180	-2.794829
H	1.787853	-3.158964	-3.207149
C	3.929203	-2.734210	-0.139577
H	4.427075	-2.131928	0.623463
H	3.548551	-3.640753	0.335509
H	4.671062	-3.023051	-0.887540
S	-1.957709	-1.883393	2.387519
C	-0.613040	-2.695002	3.230668
H	0.185694	-2.871661	2.507008
H	-0.265876	-2.052113	4.039167
H	-1.008701	-3.633462	3.620685
O	-2.513466	-2.828943	1.410787
O	-2.855039	-1.268621	3.367907
O	-1.141625	1.096873	-0.049979
H	1.462126	0.507023	0.675213
Sn	1.476528	2.297621	0.718821
C	3.456430	2.889348	1.292539
H	3.733384	2.458272	2.257325
H	4.195038	2.570034	0.552789
H	3.515171	3.977898	1.377714
C	0.090425	3.002330	2.185966
H	0.473519	3.899980	2.677385
H	-0.857468	3.248816	1.702346
H	-0.100666	2.254336	2.959952
C	1.059560	3.140434	-1.198658
H	1.914871	3.035105	-1.869656
H	0.186111	2.662484	-1.647705
H	0.841616	4.206411	-1.090452
O	-3.927030	0.929236	-0.512242
O	-2.708874	0.265299	-2.296942
C	-3.959088	0.833455	-1.927281
C	-5.096638	-0.078726	-2.367160
H	-6.055238	0.336269	-2.048536
H	-5.094130	-0.185254	-3.454127
H	-4.979477	-1.069642	-1.918626
C	-4.040432	2.230276	-2.501553
H	-4.010863	2.188453	-3.592199
H	-4.970491	2.708890	-2.188552
H	-3.191025	2.808900	-2.134542

Structure **10b_Int1**

C	2.773662	0.462034	0.127776
C	1.811401	0.495255	1.324058
C	1.236263	-1.415656	0.255114
C	2.349440	-0.837068	-0.626806
H	2.758060	1.359401	-0.495662
H	2.036280	-0.656934	-1.662063
C	0.370982	0.756869	0.846339
H	-0.295231	0.865528	1.708847
C	-0.026463	-0.544323	0.047537
H	-0.155926	-0.303537	-1.022280
H	2.137394	1.155461	2.130368
H	1.049216	-2.476072	0.081099
N	-1.186213	-1.095143	0.685377
Sn	-3.053144	-0.631130	-0.085671
C	-2.874570	0.010924	-2.105850
H	-2.395638	-0.756771	-2.718575
H	-3.866457	0.204537	-2.522168
H	-2.288753	0.930076	-2.160067
C	-3.961428	0.776509	1.222320
H	-3.862246	0.449225	2.259812
H	-3.482234	1.748741	1.104213
H	-5.025828	0.871969	0.994899
C	-4.077914	-2.495739	0.020828
H	-3.607805	-3.236688	-0.629452
H	-4.062779	-2.880219	1.043051
H	-5.120081	-2.383290	-0.288162
S	0.014450	2.195885	-0.163380
C	0.679097	3.571024	0.755224
H	0.220924	3.590747	1.744304
H	1.762561	3.463238	0.819165
H	0.418158	4.469806	0.194500
O	-1.449119	2.316661	-0.194344
O	0.729794	2.104914	-1.439955
O	4.085177	0.182523	0.582171
O	3.502472	-1.658454	-0.573509
C	4.611989	-0.831345	-0.261347
C	5.619619	-1.641605	0.523656
H	5.139668	-2.051706	1.414064
H	5.997586	-2.460893	-0.091044
H	6.456121	-1.007125	0.823340
C	5.193023	-0.219617	-1.531729

H	6.018497	0.451021	-1.282832
H	5.558762	-1.008706	-2.192288
H	4.428120	0.351618	-2.065382
C	1.691213	-1.005653	1.661124
H	2.651180	-1.438114	1.947537
H	0.935492	-1.217771	2.420416

Structure **10b_Int2**

C	-3.011322	-0.197552	-0.558140
C	-1.631393	-0.502360	0.058796
C	-2.960132	0.605017	1.713064
C	-3.848224	0.552288	0.519266
H	-2.940560	0.357397	-1.500744
H	-4.198286	1.532768	0.185058
C	-0.483668	0.356863	-0.495495
H	-0.498565	0.350593	-1.591194
C	0.881330	-0.134802	-0.031551
H	0.861064	-0.623469	0.959039
H	-1.370725	-1.528195	-0.223488
H	-3.222616	1.114087	2.630411
N	1.915099	0.027346	-0.738689
Sn	3.749011	-0.601784	-0.039015
C	4.494929	-1.958668	-1.490901
H	4.493692	-1.495818	-2.479841
H	5.518514	-2.255452	-1.251549
H	3.877743	-2.858443	-1.531730
C	3.461258	-1.518230	1.860955
H	2.797635	-2.382993	1.788277
H	4.419709	-1.860351	2.258389
H	3.031349	-0.811796	2.574855
C	4.905288	1.174134	0.091474
H	4.963461	1.665031	-0.882334
H	4.454157	1.870117	0.802159
H	5.921369	0.953705	0.426080
S	-0.634187	2.114243	-0.058630
C	0.555685	2.919944	-1.115439
H	1.535046	2.466692	-0.967952
H	0.228491	2.817440	-2.149803
H	0.549564	3.967995	-0.812141
O	-0.200410	2.286630	1.331394
O	-1.964817	2.579938	-0.464532
O	-3.700428	-1.423890	-0.738199
O	-4.964666	-0.320901	0.757679
C	-5.044388	-1.218984	-0.330287
C	-5.601798	-2.537458	0.161246
H	-6.618480	-2.396399	0.533532
H	-5.618796	-3.264697	-0.653287
H	-4.970170	-2.911378	0.968861
C	-5.860525	-0.612715	-1.468622
H	-5.880597	-1.292963	-2.323315
H	-6.883557	-0.425456	-1.135087
H	-5.423047	0.337297	-1.788428
C	-1.852441	-0.388714	1.588651
H	-0.956254	-0.091344	2.140597
H	-2.164421	-1.371278	1.974445

Structure **10b_Int3**

C	-2.143327	0.076434	0.013938
C	-1.778623	0.809450	-1.290828
C	-0.267955	-0.996392	-1.149523
C	-1.116013	-1.083601	0.136960
H	-2.202112	0.710231	0.900509
H	-0.505084	-1.025585	1.047610
H	-2.668121	1.297209	-1.692262
H	0.065830	-1.995575	-1.448209
C	-0.629942	1.838020	-1.165027
C	0.710466	1.221162	-1.161434
N	0.895638	-0.130519	-0.968536
H	1.598730	1.838520	-1.251830
H	-0.733439	2.586656	-1.962703
S	-0.899923	2.888255	0.347635
O	-0.401869	2.173846	1.533550
O	-2.283512	3.383199	0.347563
C	0.188161	4.282377	0.081830
H	0.089742	4.915805	0.964281
H	1.211657	3.918993	-0.009731
H	-0.127928	4.820383	-0.811869
Sn	2.497861	-0.759494	0.160868
C	4.159408	0.283550	-0.655475
H	4.117186	1.345275	-0.401743
H	5.096406	-0.118740	-0.263494
H	4.171843	0.187510	-1.743261
C	2.116670	-0.212898	2.178777
H	1.646717	-1.036884	2.720849
H	3.038737	0.060308	2.696975
H	1.434889	0.642588	2.188108
C	2.560985	-2.858215	-0.157985
H	2.619494	-3.082909	-1.225407
H	3.431385	-3.298286	0.333800
H	1.664963	-3.335833	0.246406
O	-3.376019	-0.596446	-0.199144
O	-1.900298	-2.262128	0.113950
C	-3.250835	-1.891165	0.357749
C	-3.527431	-1.863741	1.857065
H	-4.551490	-1.532905	2.043273
H	-3.389184	-2.860800	2.281051
H	-2.842713	-1.174739	2.359443
C	-4.160360	-2.835578	-0.397295
H	-4.035805	-3.853223	-0.021719
H	-5.201396	-2.533398	-0.267052
H	-3.906762	-2.809294	-1.458653
C	-1.242495	-0.351962	-2.144447
H	-2.052293	-1.047651	-2.375613
H	-0.753664	-0.033834	-3.067784

Structure **10b_Int4**

C	2.382119	0.985124	-0.625210
C	1.799849	2.136386	0.206608
C	0.481670	0.276428	0.785055
C	1.460627	-0.234043	-0.293863
H	2.429972	1.217908	-1.695335
H	0.933813	-0.642812	-1.165804
H	2.556615	2.895734	0.417598
H	0.222946	-0.537288	1.471858
C	0.598786	2.705076	-0.516067
C	-0.562147	2.025733	-0.483061

N	-0.733511	0.820563	0.181199
H	-1.449827	2.413567	-0.980174
H	0.668105	3.641206	-1.056257
Sn	-2.372037	-0.369729	-0.016850
C	-1.730644	-2.158674	-0.976651
H	-2.523628	-2.910300	-0.974208
H	-1.447993	-1.958847	-2.012785
H	-0.862254	-2.577940	-0.461168
C	-3.757109	0.748219	-1.175286
H	-4.684984	0.186102	-1.303195
H	-3.998823	1.694707	-0.686439
H	-3.351753	0.964192	-2.166343
C	-3.073820	-0.783084	1.946794
H	-3.415945	0.133774	2.431117
H	-3.904840	-1.491888	1.920719
H	-2.278814	-1.216267	2.558437
O	3.653781	0.588127	-0.132490
O	2.338162	-1.190780	0.274035
C	3.666849	-0.824545	-0.067864
C	4.046125	-1.421380	-1.419923
H	5.052055	-1.102914	-1.702612
H	4.015353	-2.512199	-1.371439
H	3.345962	-1.088543	-2.191469
C	4.591756	-1.254371	1.050261
H	4.570416	-2.340969	1.156397
H	5.613815	-0.938518	0.831338
H	4.263993	-0.793705	1.983924
C	1.298625	1.392427	1.454261
H	2.133930	0.954515	2.007646
H	0.696668	2.014442	2.119641

Structure **10b_Int$_{HAT}$**

C	-2.794909	0.460959	-0.118630
C	-1.844959	0.509739	-1.324190
C	-1.250059	-1.413281	-0.280720
C	-2.361759	-0.847911	0.613550
H	-2.772168	1.349559	0.516600
H	-2.042019	-0.684251	1.649250
C	-0.397719	0.760779	-0.860690
H	0.261781	0.880298	-1.726190
C	0.018101	-0.549981	-0.078020
H	0.137731	-0.317451	0.988490
H	-2.185358	1.175829	-2.119870
H	-1.058919	-2.473731	-0.114020
N	1.227461	-1.177692	-0.537540
Sn	3.074641	-0.614212	0.090000
C	2.807261	0.001218	2.104280
H	2.316921	-0.789252	2.677990
H	3.773191	0.209187	2.570770
H	2.199061	0.906458	2.150280
C	3.977611	0.847417	-1.170240
H	4.013591	0.500537	-2.206160
H	3.411952	1.778348	-1.121790
H	5.003571	1.039547	-0.845000
C	4.233951	-2.400903	-0.016610
H	3.828810	-3.157663	0.658770
H	4.232180	-2.813243	-1.028810
H	5.272261	-2.208913	0.265660
S	-0.047898	2.194429	0.152950
C	-0.763228	3.577009	-0.717040
H	-0.330798	3.626899	-1.716700
H	-1.845748	3.449499	-0.757340
H	-0.505778	4.468849	-0.143770
O	1.412332	2.355088	0.158220
O	-0.731598	2.063819	1.445070
H	1.142401	-1.516762	-1.490490
O	-4.112449	0.187940	-0.561660
O	-3.513429	-1.671260	0.554060
C	-4.627739	-0.840310	0.270980
C	-5.193199	-0.251240	1.559050
H	-6.022629	0.422330	1.332130
H	-5.549459	-1.051990	2.210710
H	-4.422309	0.312050	2.092460
C	-5.645169	-1.638140	-0.514280
H	-5.177529	-2.031720	-1.418630
H	-6.013529	-2.468810	0.090910
H	-6.486599	-0.999750	-0.791030
C	-1.734939	-0.988551	-1.676790
H	-2.702309	-1.417641	-1.943200
H	-1.014529	-1.190131	-2.474790

Structure **10b_TS1**

C	-2.581768	0.150149	0.058278
C	-1.937735	0.747596	-1.204413
C	-0.788044	-1.203314	-0.800709
C	-1.752600	-1.139923	0.356940
H	-2.635077	0.844789	0.901908
H	-1.276702	-1.147164	1.344364
C	-0.621170	1.470506	-0.884615
H	-0.190232	1.864442	-1.810894
C	0.405080	0.508646	-0.274693
H	0.230711	0.292165	0.792330
N	1.568380	0.396302	-0.849099
Sn	2.989744	-0.742869	0.091327
C	3.576921	-2.236767	-1.301988
H	3.923199	-1.777214	-2.229987
H	4.387439	-2.846458	-0.896125
H	2.739517	-2.896873	-1.539025
C	2.105343	-1.600566	1.833003
H	1.248327	-2.226500	1.569069
H	2.831037	-2.227864	2.356102
H	1.764612	-0.828565	2.527609
C	4.593132	0.565217	0.579704
H	4.942414	1.091712	-0.310930
H	4.274255	1.307027	1.315352
H	5.432862	0.007131	1.000124
S	-0.851271	2.907042	0.179735
C	0.676356	3.805649	-0.026376
H	1.508385	3.127207	0.168824
H	0.721329	4.198981	-1.041596
H	0.657178	4.616570	0.702707
O	-1.950569	3.707640	-0.363102
O	-0.918710	2.455922	1.575571
H	-0.111954	-2.044871	-0.919906
H	-2.634525	1.390422	-1.744374
O	-3.863295	-0.348166	-0.280832

O	-2.698094	-2.198061	0.251755
C	-3.997759	-1.624067	0.319333
C	-4.440227	-1.491798	1.772234
H	-5.419292	-1.010517	1.822801
H	-4.500297	-2.478664	2.236148
H	-3.726012	-0.885763	2.336733
C	-4.941884	-2.457277	-0.518672
H	-5.012557	-3.466284	-0.107749
H	-5.935122	-2.004013	-0.522055
H	-4.565007	-2.509436	-1.541677
C	-1.455681	-0.509025	-1.952974
H	-2.311681	-1.089341	-2.319875
H	-0.782769	-0.293104	-2.785748

Structure **10b_TS2**

C	-1.993181	-0.443427	0.112620
C	-1.843241	0.493060	-1.096274
C	-0.187985	-1.192360	-1.316480
C	-0.829772	-1.478469	0.011573
H	-2.027869	0.087791	1.070674
H	-0.138064	-1.422544	0.864942
C	-0.935420	1.704631	-0.783718
H	-0.730203	2.222785	-1.729132
C	0.359415	1.265132	-0.128584
H	0.506022	1.535791	0.927524
N	1.087468	0.430325	-0.791583
Sn	2.784972	-0.327622	0.109062
C	2.759457	-2.432571	-0.178782
H	2.842218	-2.679487	-1.239693
H	3.602518	-2.892061	0.342841
H	1.837717	-2.875706	0.206147
C	2.691168	0.182307	2.175322
H	1.751388	-0.158547	2.617243
H	3.511805	-0.294869	2.716147
H	2.767506	1.261485	2.326169
C	4.421513	0.587619	-0.887343
H	4.368111	0.394356	-1.960806
H	4.414414	1.668596	-0.732805
H	5.369865	0.193894	-0.514522
S	-1.810583	2.917630	0.225853
C	-0.790269	4.376097	0.084920
H	0.221033	4.134908	0.414848
H	-0.800071	4.720481	-0.948929
H	-1.235255	5.124191	0.742356
O	-1.773968	2.487127	1.628297
O	-3.101534	3.195431	-0.406183
H	0.488588	-1.915321	-1.759634
H	-2.821918	0.857092	-1.418411
O	-3.149957	-1.239388	-0.077094
O	-1.479857	-2.747804	0.002970
C	-2.841013	-2.549106	0.360328
C	-3.009518	-2.667938	1.871481
H	-4.046912	-2.470987	2.150978
H	-2.733032	-3.672276	2.199859
H	-2.367493	-1.947017	2.385264
C	-3.696904	-3.528910	-0.412540
H	-3.431394	-4.552009	-0.138774
H	-4.752274	-3.362453	-0.187201
H	-3.527496	-3.385178	-1.481091
C	-1.158874	-0.406914	-2.147950
H	-1.914919	-1.063739	-2.601464
H	-0.675560	0.159727	-2.949568

Structure **10b_TS3**

C	-2.211856	0.082500	-0.203367
C	-1.755401	0.612635	-1.574423
C	-0.249191	-1.119404	-1.051698
C	-1.166651	-1.002279	0.183779
H	-2.334702	0.843477	0.568479
H	-0.616916	-0.757016	1.100073
C	-0.613779	1.629322	-1.495620
C	0.683454	1.079954	-1.332838
N	0.894724	-0.208364	-0.958379
H	1.550731	1.732319	-1.387214
H	-0.673658	2.472182	-2.182641
S	-0.736066	2.870063	0.252316
O	0.208874	3.956229	-0.083608
O	-0.390550	2.002515	1.406833
C	-2.291766	3.694656	0.684650
H	-2.082311	4.310858	1.561393
H	-2.592541	4.319278	-0.156279
H	-3.053639	2.949404	0.912980
Sn	2.539207	-0.684347	0.216595
C	1.984055	-0.355509	2.236827
H	2.865046	-0.168598	2.855165
H	1.326083	0.517254	2.275571
H	1.452783	-1.219521	2.642555
C	4.035689	0.650662	-0.472935
H	3.825360	1.670598	-0.143972
H	5.013492	0.362001	-0.080813
H	4.086923	0.638505	-1.563962
C	2.911826	-2.720913	-0.255178
H	2.952986	-2.858534	-1.338022
H	3.865576	-3.043849	0.168260
H	2.127753	-3.366330	0.147316
H	0.117887	-2.144872	-1.158154
H	-2.605812	1.024467	-2.122024
O	-3.411457	-0.654955	-0.373504
O	-1.923906	-2.191753	0.320149
C	-3.294430	-1.829167	0.411710
C	-3.668347	-1.546516	1.862850
H	-4.709057	-1.220783	1.926249
H	-3.538401	-2.448972	2.464099
H	-3.029304	-0.761134	2.276556
C	-4.132135	-2.920458	-0.217502
H	-3.811635	-3.071700	-1.249899
H	-4.008629	-3.851958	0.338451
H	-5.186199	-2.636079	-0.204261
C	-1.172812	-0.673448	-2.192704
H	-1.962949	-1.413834	-2.336371
H	-0.640867	-0.510762	-3.131954

Structure **10b_TS$_{HAT}$**

C	3.456125	-0.872651	-0.145879
C	2.626699	-0.793299	-1.436685
C	1.911882	1.001609	-0.258574

C	2.917064	0.329713	0.687622
H	3.399847	-1.828435	0.380746
H	2.479808	0.035198	1.648695
C	1.144085	-1.103099	-1.138159
H	0.563481	-1.144995	-2.063885
C	0.628178	0.127064	-0.304942
H	0.359217	-0.181654	0.719530
H	3.046696	-1.370583	-2.262791
H	1.727042	2.044989	0.005594
N	-0.446426	0.787781	-1.009110
Sn	-1.286226	2.455098	-0.138817
C	-0.010979	4.117226	-0.549361
H	0.514795	3.964460	-1.495358
H	-0.597273	5.035846	-0.630977
H	0.732802	4.257429	0.238642
C	-1.444344	2.149946	1.969431
H	-1.791873	1.139985	2.205740
H	-0.467792	2.288024	2.441368
H	-2.142368	2.862052	2.416845
C	-3.146865	2.693507	-1.139400
H	-3.061895	3.458823	-1.914227
H	-3.432629	1.752136	-1.614837
H	-3.942072	2.983884	-0.449560
S	0.778364	-2.648882	-0.303278
C	1.724982	-3.882149	-1.177609
H	1.429567	-3.870738	-2.227265
H	2.788985	-3.673804	-1.061582
H	1.470758	-4.839371	-0.720113
O	-0.643741	-2.951965	-0.519650
O	1.268762	-2.580825	1.079482
H	-1.709045	-0.199068	-0.645213
Sn	-2.933721	-1.182533	0.246310
C	-4.522002	0.170861	0.773647
H	-4.141573	1.055625	1.292687
H	-5.076865	0.506920	-0.105599
H	-5.222751	-0.333982	1.445313
C	-2.113055	-1.891534	2.088766
H	-2.360273	-2.948757	2.215158
H	-1.023936	-1.809884	2.101322
H	-2.526803	-1.334516	2.933372
C	-3.786472	-2.718231	-0.961364
H	-4.204256	-2.302341	-1.880954
H	-3.023282	-3.453036	-1.218755
H	-4.592653	-3.217493	-0.417269
O	4.801490	-0.525696	-0.418154
O	4.048707	1.162407	0.865757
C	5.207387	0.390413	0.587696
C	5.659856	-0.354571	1.838820
H	6.523452	-0.983140	1.610433
H	5.932138	0.358755	2.619687
H	4.854733	-0.990839	2.217072
C	6.274195	1.298283	0.016897
H	6.561302	2.046538	0.758293
H	7.154450	0.713015	-0.256388
H	5.885175	1.801516	-0.870131
C	2.534171	0.733093	-1.636816
H	1.875483	1.021750	-2.458693
H	3.518932	1.186643	-1.759350

Structure **10c_Int1**

C	2.579464	1.095534	-0.092724
C	1.655590	0.517499	0.997807
C	1.137451	-0.706396	-0.820780
C	2.160669	0.317583	-1.351425
H	2.501229	2.181244	-0.179830
H	1.723411	0.941240	-2.137460
C	0.176521	0.912402	0.701418
H	-0.438398	0.587338	1.548828
C	-0.200086	0.059241	-0.564424
H	-0.453353	0.702366	-1.425279
N	-1.231937	-0.840110	-0.150113
Sn	-3.222374	-0.323897	-0.425755
C	-4.089943	-2.220858	-0.853973
H	-3.880232	-2.922402	-0.043171
H	-5.173880	-2.140843	-0.966256
H	-3.681044	-2.635670	-1.778044
C	-3.413096	0.992263	-2.084009
H	-2.965218	1.958106	-1.846423
H	-2.922212	0.576540	-2.967489
H	-4.468775	1.140874	-2.324092
C	-3.957055	0.421734	1.423268
H	-3.561456	-0.173174	2.249926
H	-3.649350	1.459806	1.550942
H	-5.047540	0.361694	1.450147
S	-0.323668	2.606409	0.391417
C	0.112249	3.528638	1.852570
H	-0.276343	3.012630	2.731357
H	1.195267	3.639116	1.897072
H	-0.365135	4.503637	1.741354
O	-1.789569	2.563951	0.303787
O	0.426339	3.149531	-0.744725
C	1.025406	-1.826910	1.415142
O	0.630005	-1.603509	2.540764
N	1.624667	-0.899531	0.573368
O	1.020389	-3.044139	0.856942
C	0.286573	-4.024350	1.588563
H	0.354403	-4.940106	1.004220
H	0.720084	-4.167921	2.579509
H	-0.755167	-3.712018	1.691687
O	3.915411	0.720476	0.143339
O	3.341026	-0.277760	-1.829766
C	4.402478	-0.124982	-0.894496
C	5.559394	0.570462	-1.592579
H	6.379395	0.731753	-0.889338
H	5.918416	-0.040804	-2.423545
H	5.221476	1.534981	-1.977662
C	4.786806	-1.476260	-0.315035
H	5.148821	-2.130267	-1.112104
H	5.580116	-1.350153	0.425900
H	3.913896	-1.927129	0.160377
C	0.995481	-1.917395	-1.713531
H	0.183039	-2.559174	-1.376455
H	0.781309	-1.566768	-2.728628
H	1.927977	-2.482582	-1.734436
C	2.170193	0.789474	2.399053
H	3.069993	0.193491	2.561289

H	2.455826	1.841365	2.487741
H	1.434362	0.538174	3.160391

Structure **10c_Int2**

C	-2.669480	0.678230	-1.053222
C	-1.211717	0.206969	-0.759293
C	-2.512387	-0.094480	1.212309
C	-3.428101	0.632541	0.289405
H	-2.669601	1.661908	-1.526716
H	-3.712693	1.613853	0.674173
C	-0.223007	1.405255	-0.583495
H	-0.126836	1.914297	-1.548638
C	1.146193	0.901397	-0.146346
H	1.169952	0.584662	0.909446
N	2.098504	0.807932	-0.969009
Sn	3.715555	-0.319418	-0.361582
C	3.524317	-2.109499	-1.485415
H	3.488722	-1.891673	-2.554901
H	4.358410	-2.789190	-1.297559
H	2.594136	-2.603886	-1.196520
C	3.542238	-0.673909	1.729639
H	2.633619	-1.251741	1.914627
H	4.400203	-1.245863	2.090635
H	3.489131	0.257925	2.297548
C	5.457929	0.790960	-0.862450
H	5.446483	1.060008	-1.920816
H	5.515246	1.710234	-0.275660
H	6.359590	0.205652	-0.667792
N	-1.374507	-0.531606	0.515695
C	-0.551694	-1.565339	0.883641
O	0.546911	-1.797761	0.405595
S	-0.734869	2.689636	0.592342
C	0.534156	3.926412	0.373689
H	1.506300	3.496785	0.613989
H	0.504179	4.289441	-0.653648
H	0.284572	4.728451	1.070013
O	-0.619266	2.160321	1.954520
O	-2.003995	3.285567	0.162366
O	-1.109997	-2.315630	1.848565
C	-0.279370	-3.366648	2.341375
H	-0.871990	-3.876611	3.098196
H	-0.011678	-4.051866	1.535644
H	0.632439	-2.958518	2.782515
O	-3.363601	-0.231603	-1.879135
O	-4.643034	-0.053168	-0.020877
C	-4.417208	-0.866397	-1.152082
C	-4.012735	-2.283789	-0.758157
H	-3.130633	-2.276388	-0.114433
H	-4.832531	-2.761644	-0.216419
H	-3.793842	-2.868520	-1.655572
C	-5.666856	-0.845696	-2.011704
H	-5.503070	-1.422286	-2.924671
H	-6.504420	-1.280707	-1.462239
H	-5.903672	0.186902	-2.272630
C	-2.676256	-0.184075	2.684284
H	-3.382454	0.587294	3.003097
H	-3.057771	-1.156673	3.013740
H	-1.721319	-0.007119	3.188198
C	-0.705305	-0.658785	-1.914839
H	-0.881557	-0.121748	-2.850284
H	0.353605	-0.886668	-1.822084
H	-1.280776	-1.584558	-1.948725

Structure **10c_Int3**

C	-1.865165	-0.773896	-0.855976
C	-1.752675	-0.805103	0.687609
C	-0.391652	0.993743	0.004145
C	-0.890961	0.318115	-1.305966
H	-1.699321	-1.749439	-1.312737
H	-0.042367	-0.067636	-1.873359
C	-0.498508	-1.593111	1.210746
C	0.695595	-0.727574	1.313670
N	0.852563	0.349607	0.469422
H	1.564237	-1.068250	1.864932
H	-0.742638	-2.060821	2.171371
N	-1.469693	0.626717	0.954701
C	-1.417482	1.024580	2.281137
O	-1.766102	0.353214	3.229544
O	-0.983766	2.288369	2.402916
C	-0.915164	2.765133	3.746433
H	-0.526265	3.779393	3.678235
H	-1.906555	2.764435	4.202611
H	-0.249172	2.137252	4.340955
S	-0.088987	-3.046730	0.132380
O	0.680467	-2.555284	-1.023793
O	-1.279166	-3.874607	-0.110942
C	1.030283	-3.996599	1.152802
H	1.344275	-4.843194	0.540647
H	1.889489	-3.379590	1.415577
H	0.497752	-4.342833	2.038412
Sn	2.673205	0.614377	-0.479366
C	2.402538	0.577029	-2.589584
H	3.355533	0.785984	-3.082651
H	2.062903	-0.414202	-2.899396
H	1.674353	1.317566	-2.928992
C	3.798389	-1.061427	0.172300
H	3.263120	-1.966513	-0.127471
H	4.783461	-1.069808	-0.299706
H	3.939908	-1.068029	1.255505
C	3.459514	2.478919	0.173082
H	3.340522	2.583579	1.253843
H	4.523359	2.552513	-0.065331
H	2.941442	3.309377	-0.311736
O	-3.136691	-0.304213	-1.253272
O	-1.645516	1.174541	-2.128027
C	-3.036063	0.921660	-1.963001
C	-3.696856	2.032291	-1.159373
H	-3.564387	2.990862	-1.667702
H	-4.766208	1.829550	-1.060632
H	-3.252014	2.077764	-0.163227
C	-3.651557	0.749116	-3.340576
H	-4.711425	0.502738	-3.248071
H	-3.549853	1.672309	-3.915712
H	-3.138496	-0.059562	-3.864580
C	-0.154714	2.482332	-0.219058
H	0.405537	2.914266	0.607829

H	0.406799	2.609553	-1.152020
H	-1.101126	3.008961	-0.338771
C	-3.040856	-1.345019	1.294973
H	-3.222645	-2.334769	0.865712
H	-2.966977	-1.423579	2.376869
H	-3.871555	-0.690616	1.029378

Structure **10c_Int4**

C	-2.270224	0.622871	-1.322391
C	-1.801395	-0.841591	-1.221175
C	-0.369538	0.344878	0.229177
C	-1.286104	1.410903	-0.436830
H	-2.279415	0.936771	-2.370278
H	-0.660061	2.129679	-0.972833
C	-0.632438	-1.009664	-2.166920
C	0.568730	-0.520815	-1.803425
N	0.816971	0.144887	-0.612341
H	1.430429	-0.648281	-2.454778
H	-0.771866	-1.495306	-3.125294
Sn	2.751772	0.480068	0.003602
C	3.287215	-0.817496	1.598600
H	4.274512	-1.251553	1.424320
H	3.308690	-0.275008	2.546914
H	2.547478	-1.617063	1.674182
C	2.893835	2.534836	0.542838
H	3.886193	2.923017	0.300367
H	2.153673	3.120793	-0.007878
H	2.721950	2.678582	1.611861
C	3.893822	0.081679	-1.746444
H	3.545423	0.678567	-2.592624
H	4.940628	0.336989	-1.562304
H	3.849867	-0.974003	-2.023727
N	-1.229726	-0.862782	0.164301
C	-0.723777	-2.015902	0.722978
O	0.195256	-2.069157	1.517899
O	-1.403189	-3.110450	0.340964
C	-0.944522	-4.327850	0.924037
H	-1.581845	-5.108571	0.512516
H	0.099835	-4.508907	0.663035
H	-1.037102	-4.291346	2.010987
O	-3.543768	0.859080	-0.760430
O	-2.101333	2.108255	0.476261
C	-3.442882	1.637643	0.421054
C	-3.764259	0.783362	1.639015
H	-3.632850	1.369701	2.552220
H	-4.800512	0.439939	1.585580
H	-3.101936	-0.084338	1.660503
C	-4.361160	2.840835	0.293662
H	-5.398105	2.511468	0.198463
H	-4.270428	3.477795	1.176413
H	-4.082261	3.413175	-0.593138
C	-0.000731	0.778095	1.639570
H	0.728762	0.103327	2.085992
H	0.387117	1.801403	1.607370
H	-0.894267	0.788130	2.263735
C	-2.959468	-1.788667	-1.495256
H	-3.454245	-1.455992	-2.413447
H	-2.621816	-2.814079	-1.628353
H	-3.685527	-1.743552	-0.682110

Structure **10c_Int$_{HAT}$**

C	2.336359	0.664563	0.199947
C	0.978503	0.484429	0.904906
C	0.549802	-0.163700	-1.220975
C	2.016661	0.333193	-1.276676
H	2.789254	1.646334	0.367937
H	2.144628	1.180803	-1.958618
C	-0.013601	1.547323	0.365274
H	-0.907949	1.544095	0.995323
C	-0.369359	1.085949	-1.072223
H	-0.055473	1.832537	-1.812693
N	-1.794401	0.871729	-1.189229
Sn	-2.899111	-0.508793	-0.158485
C	-2.551285	-0.396606	1.937921
H	-2.493564	0.627811	2.313697
H	-3.378531	-0.891231	2.454695
H	-1.627666	-0.923680	2.189746
C	-2.669688	-2.537467	-0.775490
H	-2.918714	-2.659304	-1.833002
H	-1.655734	-2.912349	-0.617128
H	-3.362971	-3.155074	-0.196663
C	-4.874682	0.129421	-0.636201
H	-5.061079	1.132259	-0.245462
H	-5.029086	0.153058	-1.718011
H	-5.616132	-0.548965	-0.206984
S	0.549709	3.256979	0.392877
C	-0.960372	4.134538	0.026963
H	-1.432949	3.670104	-0.840557
H	-1.614049	4.082447	0.897355
H	-0.675046	5.165784	-0.184367
O	1.479346	3.470351	-0.722984
O	0.986580	3.603914	1.747898
C	0.691771	-2.010803	0.476915
O	0.780063	-2.920859	-0.322881
N	0.431781	-0.694917	0.163808
O	0.716015	-2.205754	1.806380
C	0.937998	-3.558121	2.204577
H	0.928852	-3.547484	3.292808
H	0.147958	-4.204862	1.818834
H	1.902291	-3.912440	1.834868
H	-2.075331	0.912576	-2.164774
O	2.890002	-0.729301	-1.602455
O	3.195711	-0.383464	0.599949
C	3.857757	-0.847397	-0.569146
C	4.203546	-2.308283	-0.385762
H	4.710887	-2.682418	-1.277430
H	4.866082	-2.427157	0.474235
H	3.284276	-2.876507	-0.233637
C	5.066744	0.027472	-0.877775
H	5.791528	-0.032512	-0.062843
H	5.539693	-0.306774	-1.803562
H	4.763346	1.070872	-1.001158
C	1.080492	0.447372	2.416371
H	0.122670	0.190925	2.871797
H	1.830197	-0.275496	2.731509
H	1.372910	1.446011	2.748723

C	0.179369	-1.051834	-2.386737
H	-0.809597	-1.491660	-2.259456
H	0.167882	-0.427883	-3.287678
H	0.908124	-1.848901	-2.509947

Structure **10c_TS1**

C	2.470915	0.716637	-0.678532
C	1.883728	0.677978	0.750731
C	0.773877	-0.946587	-0.390659
C	1.611523	-0.285878	-1.468485
H	2.486615	1.728654	-1.089259
H	0.993546	0.152839	-2.256077
C	0.503754	1.399970	0.805203
H	0.166347	1.378286	1.847174
C	-0.546811	0.653658	-0.038279
H	-0.585032	0.974108	-1.093068
N	-1.579313	0.155917	0.596617
Sn	-3.217100	-0.422164	-0.481511
C	-3.580078	-2.485307	-0.098856
H	-3.256309	-2.743211	0.912699
H	-4.646001	-2.709444	-0.182957
H	-3.039054	-3.118026	-0.805924
C	-2.862225	-0.044738	-2.552805
H	-1.965716	-0.556375	-2.912534
H	-3.709825	-0.395645	-3.146628
H	-2.738249	1.024783	-2.740149
C	-4.815743	0.779748	0.243633
H	-4.921377	0.662202	1.324213
H	-4.633079	1.835678	0.030856
H	-5.760059	0.496132	-0.227127
N	1.462523	-0.741575	0.823460
C	1.085117	-1.296106	2.041769
O	1.072021	-0.702560	3.096213
S	0.540238	3.136307	0.329943
C	-1.053570	3.726813	0.872365
H	-1.833696	3.124883	0.405250
H	-1.103669	3.658715	1.958808
H	-1.117016	4.766049	0.546829
O	1.575616	3.829533	1.099221
O	0.577316	3.214153	-1.136234
O	0.797666	-2.596100	1.907158
C	0.335676	-3.227399	3.104314
H	0.125608	-4.258712	2.828792
H	1.105419	-3.183392	3.876087
H	-0.567175	-2.732739	3.467173
O	3.767896	0.164872	-0.702007
O	2.529927	-1.171396	-2.073465
C	3.810845	-1.034782	-1.468214
C	4.104047	-2.221787	-0.562779
H	4.140623	-3.138417	-1.156366
H	5.069197	-2.080826	-0.070343
H	3.325959	-2.315253	0.197132
C	4.839934	-0.872795	-2.572743
H	5.831885	-0.727765	-2.139551
H	4.854444	-1.762873	-3.205528
H	4.581756	-0.003074	-3.179771
C	2.894570	1.116714	1.795637
H	3.316814	2.076230	1.492945
H	2.434457	1.222563	2.775833
H	3.696885	0.378468	1.844479
C	0.005438	-2.181459	-0.709433
H	-0.762875	-2.379336	0.039188
H	-0.462065	-2.054520	-1.691118
H	0.670853	-3.049178	-0.759417

Structure **10c_TS2**

C	1.856685	-0.113483	-1.122973
C	1.838312	0.457556	0.313657
C	0.252725	-1.305260	0.247286
C	0.725761	-1.156431	-1.178246
H	1.764633	0.692312	-1.855699
H	-0.097794	-0.871488	-1.840957
C	0.839512	1.662089	0.412572
H	0.757551	1.896236	1.480496
C	-0.518309	1.295192	-0.169464
H	-0.809578	1.801704	-1.101050
N	-1.129403	0.265221	0.331816
Sn	-3.012627	-0.172055	-0.401124
C	-3.022587	-1.946955	-1.578782
H	-3.112632	-2.835813	-0.951516
H	-3.884366	-1.914301	-2.250633
H	-2.123575	-2.045456	-2.191497
C	-3.573316	1.498464	-1.597522
H	-2.946012	1.566781	-2.489926
H	-4.611675	1.401034	-1.922914
H	-3.476633	2.432012	-1.037971
C	-4.205702	-0.387206	1.340790
H	-3.823010	-1.200144	1.962309
H	-4.188152	0.530766	1.931906
H	-5.242761	-0.611831	1.081604
N	1.195988	-0.646908	1.079332
C	1.072699	-0.518223	2.447561
O	1.544183	0.392042	3.102291
S	1.425304	3.193038	-0.349433
C	0.144688	4.356587	0.097903
H	-0.811749	4.031717	-0.311347
H	0.107137	4.437054	1.184327
H	0.446459	5.307574	-0.343681
O	1.396978	3.030283	-1.808140
O	2.661152	3.636896	0.294913
C	0.245866	-1.459243	4.411187
H	-0.319237	-2.344854	4.695543
H	1.219083	-1.450019	4.904666
H	-0.299405	-0.552909	4.680256
O	0.404592	-1.544361	2.994994
O	3.026876	-0.854228	-1.399526
O	1.323726	-2.315052	-1.726401
C	2.729558	-2.239835	-1.534476
C	3.156869	-3.001024	-0.285700
H	4.239909	-2.924689	-0.160808
H	2.672390	-2.586035	0.600692
H	2.884896	-4.054914	-0.383988
C	3.405121	-2.758333	-2.789339
H	4.488462	-2.657325	-2.697232
H	3.157856	-3.811476	-2.939669
H	3.060449	-2.179098	-3.647376

C	-0.442557	-2.566388	0.641213
H	-1.249845	-2.377698	1.355303
H	-0.846230	-3.035975	-0.258192
H	0.247550	-3.275134	1.111329
C	3.246860	0.772099	0.805127
H	3.784762	1.333247	0.039115
H	3.227751	1.353378	1.723682
H	3.770983	-0.170980	0.972534

Structure **10c_TS3**

C	2.012800	0.817378	-0.328601
C	1.777108	0.317808	1.107677
C	0.387542	-1.018355	-0.235466
C	0.977222	0.069196	-1.181335
H	1.987118	1.906560	-0.411405
H	0.185367	0.718185	-1.562330
C	0.522730	0.946451	1.760703
C	-0.693365	0.282309	1.484495
N	-0.856132	-0.524094	0.398704
H	-1.582072	0.506235	2.066812
H	0.663788	1.279193	2.789054
N	1.425422	-1.098756	0.816848
C	1.291966	-1.958135	1.893274
O	1.631697	-1.713950	3.031664
O	0.776649	-3.142610	1.527393
C	0.635173	-4.081873	2.592558
H	0.195212	-4.970301	2.143304
H	1.608434	-4.314179	3.028022
H	-0.016587	-3.682125	3.371459
S	0.138898	2.913142	0.905197
O	-0.785844	2.641479	-0.227794
O	1.250734	3.867667	0.677859
C	-0.875220	3.625113	2.209780
H	-1.299768	4.545781	1.806692
H	-1.669380	2.923162	2.466566
H	-0.241902	3.839078	3.070667
Sn	-2.647199	-0.344966	-0.662348
C	-3.794928	0.955410	0.556373
H	-3.266328	1.908955	0.632889
H	-4.771278	1.136113	0.100743
H	-3.958692	0.547384	1.556416
C	-2.232049	0.507661	-2.561794
H	-3.153213	0.565994	-3.147191
H	-1.842187	1.517319	-2.413489
H	-1.502270	-0.075074	-3.128908
C	-3.457186	-2.304627	-0.799019
H	-3.349662	-2.824043	0.155979
H	-4.519851	-2.262554	-1.049612
H	-2.945640	-2.887588	-1.567766
O	3.248753	0.317917	-0.797863
O	1.713363	-0.511275	-2.234271
C	3.072728	-0.090978	-2.139403
C	3.971701	-1.281192	-2.397336
H	3.796735	-1.670769	-3.402280
H	5.019369	-0.986664	-2.307145
H	3.749089	-2.055148	-1.660117
C	3.310546	1.072683	-3.092413
H	4.340290	1.425942	-3.004654
H	3.125411	0.757597	-4.121690
H	2.633522	1.897697	-2.853985
C	0.101890	-2.302980	-1.000789
H	-0.522357	-2.975792	-0.416354
H	-0.403738	-2.049346	-1.939481
H	1.037987	-2.796229	-1.259021
C	3.022609	0.539637	1.953611
H	3.266565	1.606502	1.910497
H	2.861476	0.243235	2.987217
H	3.856679	-0.024027	1.534723

Structure **10c_TS$_{HAT}$**

C	-2.730327	-1.646899	0.163177
C	-1.730769	-0.761409	0.956508
C	-1.364904	-0.189124	-1.203661
C	-2.527374	-1.192467	-1.303833
H	-2.589267	-2.713799	0.343254
H	-2.325562	-2.004307	-2.012779
C	-0.279123	-1.221944	0.695944
H	0.387596	-0.523441	1.214904
C	-0.084165	-1.031458	-0.842781
H	-0.176391	-2.010742	-1.347992
N	1.131949	-0.457516	-1.308457
Sn	3.054001	-1.004265	-0.826279
C	4.103566	0.564631	-1.825312
H	4.962972	0.910713	-1.245151
H	4.460541	0.232784	-2.803407
H	3.432324	1.413445	-1.982972
C	3.528670	-2.881175	-1.700972
H	3.667498	-3.632887	-0.923018
H	2.705143	-3.208323	-2.339859
H	4.433535	-2.809442	-2.308072
C	3.499559	-0.863136	1.262721
H	2.786308	-0.214668	1.785194
H	3.475200	-1.854755	1.718872
H	4.493985	-0.432047	1.406623
S	0.252655	-2.846074	1.252743
C	0.713191	-2.632576	2.962853
H	1.488773	-1.869154	3.028698
H	-0.164057	-2.362526	3.548801
H	1.103868	-3.599874	3.283755
O	1.483722	-3.138792	0.503188
O	-0.845325	-3.813106	1.192676
C	-2.494075	1.538508	0.321514
O	-2.979582	1.844143	1.394099
N	-1.669766	0.460025	0.105432
O	-2.631194	2.303367	-0.775402
C	-3.361486	3.512072	-0.568306
H	-3.328757	4.038115	-1.520909
H	-4.394248	3.294244	-0.288797
H	-2.896669	4.110528	0.217626
H	1.201069	1.002314	-0.507469
Sn	1.298246	2.491287	0.475750
C	0.436416	2.138056	2.402571
H	0.698582	2.955946	3.079204
H	-0.653387	2.080762	2.335022
H	0.811712	1.210672	2.847653
C	0.268213	4.066948	-0.532767

H	0.902044	4.500235	-1.309710
H	-0.640181	3.681750	-1.000030
H	-0.007202	4.857909	0.169477
C	3.365481	2.985567	0.726105
H	3.929192	2.120542	1.085801
H	3.806877	3.306931	-0.219934
H	3.479632	3.793788	1.453143
O	-4.045648	-1.237355	0.481440
O	-3.721079	-0.510004	-1.622895
C	-4.730618	-0.951376	-0.728487
C	-5.402607	-2.208425	-1.266170
H	-6.139063	-2.575193	-0.548198
H	-5.901218	-1.991199	-2.213370
H	-4.659743	-2.993522	-1.433850
C	-5.692722	0.189716	-0.479940
H	-6.132545	0.517360	-1.424028
H	-6.490540	-0.134455	0.191362
H	-5.152594	1.016868	-0.014386
C	-2.102191	-0.630317	2.419009
H	-2.117316	-1.638326	2.847395
H	-1.383611	-0.011789	2.962040
H	-3.093149	-0.198091	2.523304
C	-1.180883	0.668897	-2.433946
H	-0.482492	1.484136	-2.240503
H	-0.743976	0.038988	-3.213439
H	-2.135235	1.068481	-2.770733

Structure **10d_Int1**

C	2.629051	0.285596	-0.400167
C	2.004959	0.708703	0.950401
C	0.687569	-0.917708	0.342689
C	1.694504	-0.870000	-0.830549
H	2.707070	1.103424	-1.119447
H	1.207481	-0.730822	-1.800612
C	0.658059	1.439262	0.699926
H	0.261957	1.767618	1.665782
C	-0.270569	0.327957	0.139230
H	-0.420655	0.462574	-0.945160
N	-1.483096	0.221671	0.882108
Sn	-3.116634	-0.645701	-0.039262
C	-3.575544	-2.421619	1.035255
H	-3.367064	-2.281457	2.098285
H	-4.632577	-2.673002	0.923096
H	-2.980277	-3.262837	0.674477
C	-2.656905	-1.019067	-2.088209
H	-2.444981	-0.086707	-2.617650
H	-1.790945	-1.676646	-2.199761
H	-3.508065	-1.498274	-2.578137
C	-4.645689	0.819010	0.152136
H	-4.748796	1.124016	1.195509
H	-4.414354	1.704715	-0.444048
H	-5.605153	0.422227	-0.187537
S	0.717342	2.898274	-0.337280
C	-0.895795	3.620031	-0.088530
H	-1.657087	2.878150	-0.335721
H	-0.985032	3.940128	0.949207
H	-0.957853	4.474126	-0.764381
O	0.809176	2.481132	-1.741952
O	1.728291	3.813797	0.197811
O	1.491757	-0.529172	1.452526
O	3.891170	-0.311860	-0.226343
O	2.513447	-2.010393	-0.874008
C	3.824002	-1.721938	-0.395356
C	4.059282	-2.420199	0.934255
H	3.314227	-2.082028	1.655850
H	3.979073	-3.502411	0.804218
H	5.059612	-2.182620	1.304619
C	4.825599	-2.129379	-1.461837
H	5.838320	-1.879638	-1.138180
H	4.764965	-3.205032	-1.641424
H	4.602852	-1.596239	-2.388190
C	2.942922	1.352920	1.937434
H	3.262824	2.326545	1.561212
H	2.437383	1.490386	2.896151
H	3.815233	0.711947	2.074312
C	0.033680	-2.250563	0.594174
H	-0.591060	-2.203130	1.489187
H	-0.581562	-2.542918	-0.262823
H	0.808871	-3.007211	0.726623

Structure **10d_Int2**

C	-2.905138	0.095834	-0.949821
C	-1.523030	-0.430089	-0.466864
C	-2.568427	0.391564	1.387211
C	-3.551897	0.714557	0.312078
H	-2.803943	0.792099	-1.784032
H	-3.751648	1.783215	0.234921
C	-0.333703	0.505957	-0.806008
H	-0.223812	0.583554	-1.892953
C	0.968221	-0.017217	-0.209017
H	0.836077	-0.421262	0.808516
N	2.058322	0.060472	-0.842815
Sn	3.800480	-0.578661	0.054810
C	4.635689	-2.024480	-1.256463
H	4.760760	-1.604378	-2.256501
H	5.613368	-2.352773	-0.896547
H	3.986603	-2.899459	-1.330066
C	3.315947	-1.391993	1.961066
H	2.633614	-2.240077	1.867740
H	4.223200	-1.740064	2.460415
H	2.844596	-0.639904	2.597870
C	4.991704	1.173254	0.203596
H	5.132133	1.623870	-0.781282
H	4.508683	1.906656	0.853122
H	5.975194	0.943142	0.619173
S	-0.480488	2.232832	-0.236069
C	0.791917	3.052349	-1.182821
H	1.738785	2.530759	-1.045751
H	0.495195	3.059830	-2.231295
H	0.837157	4.068653	-0.788596
O	-0.103443	2.294518	1.177801
O	-1.766477	2.789891	-0.668460
O	-1.638965	-0.529930	0.957710
O	-3.783812	-0.942812	-1.325879
O	-4.818970	0.060098	0.417619
C	-4.764360	-1.150128	-0.306642

C	-4.372272	-2.314140	0.598006
H	-5.125852	-2.436278	1.379927
H	-4.314921	-3.238749	0.016952
H	-3.403450	-2.129712	1.067593
C	-6.104077	-1.369594	-0.981785
H	-6.069149	-2.269456	-1.599763
H	-6.886092	-1.486249	-0.228500
H	-6.333025	-0.507231	-1.609700
C	-1.194458	-1.811562	-1.016731
H	-1.065573	-1.764699	-2.101099
H	-0.273668	-2.191367	-0.568399
H	-2.009336	-2.499193	-0.796804
C	-2.810666	0.438426	2.845846
H	-3.459836	1.283382	3.084858
H	-3.301719	-0.475928	3.210003
H	-1.867152	0.555494	3.386252

Structure **10d_Int3**

C	-1.896131	-0.548992	0.623410
C	-1.859679	0.216018	-0.725468
C	0.099829	-0.958474	-0.704139
C	-0.596199	-1.379665	0.617184
H	-2.002359	0.128994	1.469893
H	0.057638	-1.199416	1.475227
C	-1.103508	1.560797	-0.602494
C	0.259006	1.336531	-0.019430
N	0.943512	0.203888	-0.442799
H	0.847246	2.213509	0.223442
H	-1.051710	1.977127	-1.625703
S	-1.943050	2.859824	0.359642
O	-0.922583	3.789649	0.849861
O	-2.875901	2.258876	1.318956
C	-2.910937	3.745026	-0.853090
H	-3.442597	4.517892	-0.296143
H	-2.237896	4.196352	-1.581714
H	-3.617673	3.066982	-1.330024
Sn	2.929087	0.030617	0.101732
C	3.488574	2.003302	0.640602
H	2.934658	2.343733	1.518011
H	4.555071	2.036634	0.875624
H	3.299135	2.699855	-0.179164
C	3.033887	-1.313572	1.748472
H	4.075436	-1.477754	2.035554
H	2.504929	-0.896641	2.608852
H	2.590255	-2.283812	1.511939
C	4.011138	-0.669768	-1.587790
H	3.624973	-0.204405	-2.497512
H	5.068173	-0.409969	-1.492652
H	3.931166	-1.753491	-1.693570
O	-0.990725	-0.577160	-1.544099
O	-2.931681	-1.507633	0.673767
O	-1.013955	-2.723902	0.629592
C	-2.421894	-2.812792	0.444451
C	-2.990345	-3.739516	1.503291
H	-4.077075	-3.792636	1.408501
H	-2.574215	-4.742462	1.385935
H	-2.733841	-3.354557	2.491755
C	-2.735849	-3.279922	-0.970822
H	-2.330069	-4.282903	-1.127217
H	-3.818296	-3.311111	-1.121027
H	-2.288338	-2.595039	-1.694041
C	-3.208268	0.328955	-1.402971
H	-3.913555	0.843859	-0.745026
H	-3.116117	0.869099	-2.349534
H	-3.602398	-0.667687	-1.600406
C	0.852130	-2.071072	-1.399881
H	1.302136	-1.694745	-2.321306
H	1.633570	-2.481955	-0.752890
H	0.159831	-2.880458	-1.630638

Structure **10d_Int4**

C	2.265614	0.729385	-0.952998
C	1.823753	1.780573	0.089694
C	0.473978	0.017665	0.552542
C	1.309204	-0.458424	-0.671001
H	2.200626	1.127047	-1.969138
H	0.658947	-0.734948	-1.506517
C	0.651708	2.545075	-0.472345
C	-0.550779	1.948688	-0.425865
N	-0.752661	0.679570	0.097295
H	-1.442862	2.459050	-0.782476
H	0.778743	3.540595	-0.878903
Sn	-2.568999	-0.252383	-0.065145
C	-2.315678	-1.949474	-1.325485
H	-3.252460	-2.505986	-1.409278
H	-2.016731	-1.631601	-2.327252
H	-1.550695	-2.629134	-0.942330
C	-3.823579	1.203972	-0.969962
H	-4.831226	0.798410	-1.089289
H	-3.893052	2.103281	-0.353772
H	-3.454308	1.487510	-1.958111
C	-3.281681	-0.794878	1.863241
H	-3.056602	-0.004663	2.583058
H	-4.364912	-0.937046	1.837584
H	-2.820899	-1.721104	2.212585
O	1.327248	0.967562	1.172897
O	3.562232	0.206087	-0.753778
O	2.161884	-1.542694	-0.378591
C	3.500077	-1.095145	-0.192663
C	4.418264	-2.007958	-0.985499
H	5.450645	-1.660918	-0.904942
H	4.356168	-3.028386	-0.600848
H	4.115694	-1.998876	-2.034189
C	3.855351	-1.051100	1.287495
H	3.762459	-2.049402	1.723811
H	4.886645	-0.709036	1.408026
H	3.185582	-0.360792	1.804023
C	0.175174	-1.075248	1.557110
H	-0.366533	-0.652139	2.406271
H	-0.422192	-1.873170	1.103446
H	1.107567	-1.521951	1.900065
C	2.938545	2.655886	0.614630
H	3.323980	3.287745	-0.190449
H	2.558796	3.297402	1.412921
H	3.754586	2.038548	0.994314

Structure **10d_Int$_{HAT}$**

C	2.521082	0.431006	0.221685
C	1.148114	0.359944	0.918795
C	0.757861	-0.747206	-0.921621
C	2.222422	-0.282015	-1.112600
H	2.913181	1.445892	0.126655
H	2.344785	0.350910	-1.996168
C	0.138594	1.270161	0.164650
H	-0.763208	1.373500	0.776244
C	-0.172886	0.485256	-1.137513
H	0.164093	1.044504	-2.019534
N	-1.591623	0.229059	-1.252275
Sn	-2.774733	-0.730477	0.107441
C	-2.973423	0.521039	1.821107
H	-3.076765	1.568455	1.525194
H	-3.876682	0.238320	2.368382
H	-2.129138	0.433996	2.508316
C	-2.175767	-2.691032	0.685413
H	-2.302267	-3.402975	-0.133802
H	-1.129203	-2.690331	0.995359
H	-2.792526	-3.025088	1.524192
C	-4.622353	-0.802276	-0.947540
H	-4.941457	0.203994	-1.228125
H	-4.535912	-1.399886	-1.858388
H	-5.403439	-1.248078	-0.327150
S	0.660427	2.957753	-0.145767
C	-0.864843	3.727979	-0.659368
H	-1.325706	3.100403	-1.424885
H	-1.517914	3.826275	0.207772
H	-0.600622	4.707488	-1.059804
O	1.580563	2.974617	-1.288956
O	1.098043	3.547630	1.122395
H	-1.824651	-0.035853	-2.204849
O	0.664250	-0.925912	0.505190
O	3.466431	-0.385550	0.870566
O	3.141527	-1.346574	-1.172609
C	3.790552	-1.524723	0.081233
C	5.290229	-1.543202	-0.157916
H	5.819550	-1.633338	0.793123
H	5.557798	-2.389097	-0.795143
H	5.588078	-0.614694	-0.648915
C	3.286812	-2.785224	0.766800
H	2.212218	-2.698913	0.935572
H	3.494558	-3.656639	0.140621
H	3.793266	-2.911807	1.726962
C	0.403979	-1.998602	-1.678869
H	1.088525	-2.803000	-1.405473
H	-0.621362	-2.308189	-1.474057
H	0.505513	-1.813198	-2.752663
C	1.170895	0.488518	2.418876
H	1.889017	-0.223224	2.828909
H	1.466367	1.503615	2.693266
H	0.180981	0.281918	2.833067

Structure **10d_TS1**

C	-2.487535	0.201303	0.512670
C	-1.967079	0.711811	-0.853946
C	-0.857266	-1.167892	-0.541534
C	-1.659382	-1.086744	0.738258
H	-2.388194	0.950406	1.301139
H	-1.035605	-1.092579	1.635483
C	-0.606930	1.445850	-0.697177
H	-0.274753	1.749954	-1.695131
C	0.460196	0.502616	-0.126726
H	0.383227	0.354987	0.963685
N	1.561120	0.338273	-0.799832
Sn	3.114540	-0.664107	0.071658
C	3.612307	-2.293237	-1.203240
H	3.471214	-2.006469	-2.247967
H	4.655716	-2.586666	-1.066700
H	2.982495	-3.161505	-0.997526
C	2.525532	-1.327257	2.013539
H	1.703242	-2.045812	1.963364
H	3.367186	-1.816116	2.510474
H	2.207706	-0.488508	2.638186
C	4.706698	0.741326	0.195922
H	4.919023	1.165287	-0.787897
H	4.447313	1.558736	0.872881
H	5.617293	0.266656	0.568941
S	-0.662065	2.956187	0.286893
C	0.920468	3.704539	-0.054186
H	1.710029	2.989780	0.179256
H	0.953207	3.994925	-1.104032
H	0.981874	4.581793	0.591419
O	-1.709822	3.837388	-0.233953
O	-0.692099	2.582324	1.706137
O	-1.538780	-0.495951	-1.515572
O	-3.823243	-0.238300	0.449963
O	-2.615608	-2.122429	0.861146
C	-3.895509	-1.660841	0.444988
C	-4.915460	-2.090295	1.482891
H	-5.902939	-1.708798	1.214717
H	-4.957981	-3.180220	1.536166
H	-4.627111	-1.691229	2.456800
C	-4.226229	-2.175448	-0.948722
H	-4.263782	-3.267467	-0.938940
H	-5.200379	-1.791720	-1.262110
H	-3.467191	-1.847869	-1.661087
C	-0.097531	-2.363178	-0.979456
H	0.598209	-2.089786	-1.778060
H	0.461889	-2.780595	-0.137167
H	-0.777716	-3.139193	-1.349222
C	-2.990497	1.416608	-1.710654
H	-3.315367	2.337057	-1.224525
H	-2.555186	1.664646	-2.681458
H	-3.849498	0.759550	-1.852599

Structure **10d_TS2**

C	-1.900502	-0.328551	0.606319
C	-1.864365	0.451895	-0.730199
C	-0.347428	-1.245377	-0.975528
C	-0.803447	-1.410745	0.452457
H	-1.766341	0.340846	1.459577
H	0.016755	-1.307688	1.170859
C	-0.874231	1.657780	-0.666558
H	-0.733382	1.985582	-1.703203

C	0.451387	1.218563	-0.067583
H	0.655739	1.540673	0.965345
N	1.106496	0.304182	-0.705741
Sn	2.897398	-0.307636	0.124023
C	2.906382	-2.412664	0.434126
H	3.031920	-2.953160	-0.505990
H	3.746306	-2.666183	1.086428
H	1.990079	-2.760839	0.916883
C	3.085154	0.695259	1.995104
H	2.242341	0.457163	2.649461
H	4.001544	0.377599	2.498151
H	3.125044	1.779818	1.869797
C	4.371996	0.276612	-1.286211
H	4.162080	-0.174646	-2.258449
H	4.380977	1.361703	-1.408157
H	5.366281	-0.042562	-0.965760
S	-1.508037	3.096912	0.220294
C	-0.174572	4.275987	0.067452
H	0.724883	3.883119	0.540412
H	-0.009617	4.487252	-0.989203
H	-0.518420	5.173078	0.584672
O	-1.640182	2.740091	1.637611
O	-2.661448	3.652678	-0.488540
O	-1.250383	-0.462703	-1.657789
O	-3.091521	-1.063358	0.785859
O	-1.458658	-2.633337	0.739998
C	-2.857506	-2.452816	0.575143
C	-3.567757	-3.227675	1.667335
H	-4.644676	-3.058967	1.603236
H	-3.368429	-4.295776	1.557362
H	-3.206450	-2.889621	2.639730
C	-3.302194	-2.870418	-0.821231
H	-3.075226	-3.927813	-0.978561
H	-4.379870	-2.720509	-0.926360
H	-2.788817	-2.277243	-1.580828
C	-3.232691	0.811178	-1.274070
H	-3.792771	1.410049	-0.554969
H	-3.133860	1.383320	-2.198931
H	-3.782287	-0.110069	-1.469329
C	0.254855	-2.328316	-1.793148
H	0.957959	-1.908161	-2.521093
H	0.777426	-3.042622	-1.155507
H	-0.518921	-2.873613	-2.348796

Structure **10d_TS3**

C	2.180970	0.251528	-0.042653
C	1.740040	0.717647	1.358593
C	0.356980	-1.018711	0.936935
C	1.126748	-0.817416	-0.403918
H	2.301210	1.056249	-0.771331
H	0.468877	-0.514639	-1.221498
C	0.551516	1.706618	1.332421
C	-0.714814	1.077156	1.368075
N	-0.876318	-0.210157	0.958929
H	-1.612015	1.648600	1.586438
H	0.673074	2.580684	1.973457
S	0.509804	2.762622	-0.539613
O	-0.279187	1.895657	-1.452368
O	1.759368	3.366303	-1.060800
C	-0.569044	4.135381	-0.105880
H	-0.801415	4.651478	-1.038618
H	-1.481179	3.743940	0.345622
H	-0.039851	4.799557	0.577488
Sn	-2.553056	-0.692833	-0.174386
C	-1.879971	-1.451399	-2.040753
H	-2.724292	-1.859222	-2.601893
H	-1.434807	-0.638639	-2.619349
H	-1.135117	-2.241759	-1.919136
C	-3.543892	1.170544	-0.365624
H	-2.844835	1.874964	-0.824534
H	-4.419231	1.075729	-1.012018
H	-3.875003	1.560483	0.599713
C	-3.643826	-2.135020	0.941025
H	-3.778191	-1.794781	1.970117
H	-4.630681	-2.293988	0.499660
H	-3.119908	-3.092992	0.962210
O	1.239729	-0.504445	1.915975
O	3.375307	-0.495438	0.073358
O	1.870266	-1.970313	-0.736217
C	3.254988	-1.634804	-0.755432
C	3.682245	-1.311913	-2.181343
H	4.733113	-1.014933	-2.201071
H	3.544239	-2.187208	-2.819931
H	3.077312	-0.492540	-2.579538
C	4.041133	-2.766276	-0.129413
H	3.693787	-2.911708	0.895252
H	3.892234	-3.684759	-0.700723
H	5.105537	-2.522585	-0.118629
C	2.870789	1.235238	2.219847
H	3.256075	2.165580	1.792764
H	2.505969	1.432187	3.230787
H	3.672104	0.497730	2.256911
C	0.042498	-2.461112	1.270931
H	-0.489892	-2.503172	2.223345
H	-0.577940	-2.911621	0.489303
H	0.968976	-3.030692	1.329389

Structure **10d_TS$_{HAT}$**

C	-3.107661	-1.022600	0.213456
C	-1.910019	-0.345070	0.913060
C	-1.496237	0.089875	-1.189490
C	-2.791359	-0.752855	-1.272444
H	-3.233093	-2.075593	0.470055
H	-2.661105	-1.654483	-1.878036
C	-0.599315	-1.136844	0.654079
H	0.204739	-0.693379	1.253132
C	-0.314924	-0.885689	-0.875913
H	-0.469265	-1.821760	-1.441159
N	0.965713	-0.390807	-1.257416
Sn	2.791872	-1.119837	-0.648239
C	3.114440	-3.043675	-1.490010
H	2.956745	-3.006528	-2.570423
H	4.144399	-3.361611	-1.308629
H	2.434237	-3.772739	-1.050847
C	3.083448	-0.963632	1.461779
H	2.916134	0.065727	1.795072

H	2.414482	-1.634914	2.002668
H	4.113927	-1.228509	1.713885
C	4.086725	0.282892	-1.593880
H	4.035797	0.172038	-2.679591
H	3.794944	1.306261	-1.344373
H	5.124976	0.139983	-1.283474
S	-0.506482	-2.896848	0.968277
C	-0.935018	-3.136481	2.681375
H	-0.328888	-2.471174	3.297983
H	-2.000808	-2.951422	2.814345
H	-0.700807	-4.179006	2.903644
O	0.910146	-3.250129	0.806977
O	-1.496676	-3.595648	0.143726
O	-1.666666	0.782745	0.064746
H	1.101620	1.035543	-0.422386
Sn	1.086536	2.591254	0.460858
C	3.019513	3.495257	0.273521
H	3.795033	2.843189	0.684256
H	3.266692	3.703875	-0.769947
H	3.043155	4.439789	0.824131
C	0.757401	2.293921	2.560181
H	1.439262	2.938201	3.121769
H	-0.266116	2.559583	2.833632
H	0.945168	1.262165	2.870101
C	-0.401805	3.883339	-0.354025
H	-0.186388	4.122205	-1.398036
H	-1.374803	3.389920	-0.303625
H	-0.448250	4.818604	0.209609
O	-4.301965	-0.322371	0.470396
O	-3.895392	-0.026194	-1.754472
C	-4.751680	0.369478	-0.690963
C	-6.162289	-0.090703	-1.015700
H	-6.837507	0.162618	-0.195479
H	-6.513593	0.396043	-1.928157
H	-6.163752	-1.172609	-1.162512
C	-4.657574	1.870245	-0.466538
H	-4.967061	2.400510	-1.370560
H	-5.312399	2.164452	0.357594
H	-3.626948	2.132792	-0.222087
C	-1.287030	1.043262	-2.333153
H	-0.375575	1.622523	-2.186998
H	-1.182767	0.475917	-3.261996
H	-2.150431	1.705650	-2.420030
C	-2.172110	0.102989	2.330199
H	-2.978275	0.838289	2.317704
H	-2.494077	-0.735628	2.952089
H	-1.278691	0.548344	2.771004

Structure **Ms·**

S	0.203991	-0.000005	-0.279797
O	0.737933	-1.284377	0.210024
O	0.738059	1.284314	0.210029
C	-1.569149	0.000065	0.098285
H	-2.001480	0.902594	-0.329359
H	-2.001730	-0.901997	-0.330100
H	-1.653691	-0.000408	1.186075

Structure **SnMe3H**

Sn	0.000016	0.000040	-0.232867
C	2.010444	0.185061	0.460748
H	2.038305	0.188184	1.552763
H	2.622439	-0.647356	0.106891
H	2.460675	1.114639	0.105979
C	-1.165605	1.648321	0.460749
H	-2.196027	1.572486	0.106989
H	-1.181236	1.671683	1.552779
H	-0.751563	2.594490	0.105831
C	-0.844907	-1.833591	0.460676
H	-1.871979	-1.947094	0.107207
H	-0.265265	-2.688302	0.105492
H	-0.855747	-1.859447	1.552694
H	0.000002	-0.000014	-1.946300

Structure **SnMe3·**

Sn	-0.000065	-0.000015	-0.289065
C	0.478567	1.955104	0.486976
H	0.467549	1.923797	1.580375
H	1.470209	2.273325	0.160215
H	-0.250262	2.696685	0.154925
C	-1.932467	-0.563206	0.487106
H	-2.209885	-1.565457	0.155568
H	-1.899958	-0.556493	1.580518
H	-2.704072	0.136045	0.159870
C	1.454227	-1.391828	0.486954
H	1.234672	-2.409680	0.159617
H	2.460934	-1.130740	0.155569
H	1.432082	-1.367158	1.580369

Structure **H**

H	0.000000	0.000000	0.000000

Structure **MeNHMoc**

C	-2.425401	0.005811	0.000300
H	-2.263560	1.083465	-0.000547
H	-3.000275	-0.267019	-0.889374
N	-1.124825	-0.623357	-0.000511
C	0.020691	0.103389	-0.000139
O	0.097943	1.313481	-0.000125
O	1.093441	-0.723199	0.000066
C	2.345971	-0.046103	0.000144
H	2.444565	0.581563	0.888186
H	3.104773	-0.827141	-0.001386
H	2.443372	0.584009	-0.886272
H	-1.034940	-1.626401	0.000292
H	-2.998797	-0.265814	0.891318

Structure **MeNHMoc_rad**

C	2.440547	-0.001084	-0.048863
H	2.454902	1.071329	0.060306
H	3.300656	-0.619214	0.152084
N	1.197018	-0.604049	0.009076
C	0.032460	0.115834	0.002657
O	-0.040971	1.323333	0.003200
O	-1.025827	-0.724053	-0.000729
C	-2.288036	-0.062433	-0.000170
H	-2.391054	0.566256	-0.886888

H	-3.036684	-0.852764	-0.001894
H	-2.392119	0.563235	0.888570
H	1.109725	-1.608646	-0.017238

Structure **Me$_2$O**

C	-1.160823	0.195228	-0.000004
H	-1.212123	0.838978	-0.891021
H	-2.018107	-0.479675	-0.000220
C	1.160763	0.195242	0.000006
H	1.211533	0.839550	0.890748
H	2.018282	-0.479403	0.000814
H	1.212348	0.838368	-0.891561
O	0.000090	-0.592408	-0.000004
H	-1.212291	0.838626	0.891263

Structure **Me$_2$O_rad**

C	-1.191899	0.229332	0.079776
H	-1.126571	1.258766	-0.267086
H	-2.116349	-0.322130	-0.027948
C	1.126825	0.168985	0.015738
H	1.240253	0.663185	0.987150
H	1.933283	-0.550456	-0.124651
H	1.172846	0.926352	-0.777287
O	-0.089127	-0.545702	-0.045408

Structure **Propane**

C	1.266590	-0.260760	0.000004
H	1.300339	-0.907001	-0.882863
H	2.169294	0.355659	-0.000196
C	-0.000020	0.591367	0.000009
H	-0.000039	1.248682	0.876731
H	-0.000046	1.248638	-0.876760
C	-1.266557	-0.260759	-0.000002
H	-1.300158	-0.907342	0.882626
H	-2.169322	0.355557	0.000590
H	-1.300706	-0.906493	-0.883216
H	1.300558	-0.906789	0.883021

Structure **Propyl_rad**

C	-1.293488	-0.297218	-0.040575
H	-1.282142	-1.300838	0.369615
H	-2.253707	0.124470	-0.309677
C	-0.078495	0.559763	0.056236
H	-0.101335	1.326291	-0.727871
H	-0.080268	1.117861	1.007557
C	1.218404	-0.245625	-0.040385
H	1.275590	-0.772224	-0.996747
H	2.097012	0.397850	0.049787
H	1.266328	-0.994928	0.755685

Structure **EtNHMoc**

C	-1.941411	-0.295400	-0.492848
H	-1.849650	0.412528	-1.319777
N	-0.615662	-0.836432	-0.246076
C	0.453392	-0.000089	-0.193206
O	0.439081	1.175767	-0.491369
O	1.556796	-0.663842	0.222288
C	2.731387	0.139703	0.274060
H	2.593184	0.980197	0.957294
H	3.523928	-0.516463	0.630429
H	2.974316	0.530220	-0.716282
H	-0.508342	-1.744966	0.177373
H	-2.578461	-1.118056	-0.828014
C	-2.537067	0.401784	0.726155
H	-2.633116	-0.291939	1.566084
H	-1.889416	1.228753	1.026251
H	-3.527628	0.803364	0.496846

Structure **EtNHMoc_rad**

C	-1.861058	0.372290	-0.150942
H	-1.718250	1.432856	-0.000637
N	-0.694090	-0.378538	-0.066437
C	0.548936	0.188630	-0.014985
O	0.775242	1.376869	0.029978
O	1.495737	-0.776865	-0.017771
C	2.828841	-0.278206	0.044168
H	2.979976	0.300326	0.957799
H	3.473845	-1.155234	0.035536
H	3.039180	0.361619	-0.815269
H	-0.733462	-1.385776	-0.129291
C	-3.154833	-0.316224	0.096152
H	-3.292451	-0.601221	1.150791
H	-3.988040	0.332433	-0.179507
H	-3.241311	-1.234211	-0.498380

Structure **^{i}PrNHMoc**

C	1.610846	-0.059760	0.257664
H	1.473674	-0.453171	1.269813
N	0.354336	0.592461	-0.092611
C	-0.830822	-0.019331	0.159226
O	-0.972425	-1.038005	0.803383
O	-1.852838	0.675355	-0.393234
C	-3.136935	0.111745	-0.146068
H	-3.343341	0.079300	0.925704
H	-3.848598	0.762351	-0.652028
H	-3.195312	-0.903387	-0.544147
H	0.352668	1.345830	-0.764451
C	2.721194	0.983113	0.261100
H	2.858006	1.402432	-0.742453
H	2.491359	1.799307	0.950016
H	3.667979	0.528348	0.560914
C	1.916961	-1.225772	-0.680919
H	2.834813	-1.736736	-0.376304
H	1.095450	-1.945093	-0.659353
H	2.047583	-0.865176	-1.706644

Structure **^{i}PrNHMoc_rad**

C	1.667626	-0.062271	-0.177635
N	0.378446	-0.589028	-0.005960
C	-0.806273	0.090382	-0.052721
O	-0.961290	1.283057	-0.195628
O	-1.825911	-0.791718	0.094646
C	-3.117642	-0.194057	0.058332
H	-3.281346	0.310863	-0.895974
H	-3.825631	-1.011557	0.184621
H	-3.226606	0.535257	0.863657

H	0.282739	-1.593880	0.037680	C	2.756099	-1.074103	-0.027048
C	1.924364	1.367375	0.168687	H	3.692691	-0.685710	-0.433332
H	1.259810	2.046146	-0.365579	H	2.942500	-1.337653	1.027052
H	1.775083	1.555331	1.243789	H	2.522285	-2.002723	-0.559848
H	2.961920	1.612451	-0.070178				

6. References

[1] Chen, Z.; Trudell, M.L. A Simplified Method for the Preparation of Ethynyl p-Tolyl Sulfone and Ethynyl Phenyl Sulfone. *Synth. Commun*. **1994**, *21*, 3149-3155.

[2] Eisch, J.J.; Shafii, B.; Odon, J.D.; Rheingold, A.L. Aromatic Stabilization of the Triarylborirene Ring System by Tricoordinate Boron and Facile Ring Opening with Tetracoordinate Boron. *J. Am. Chem. Soc.* **1990**, *112*, 1847-1857.

[3] Kaiser, H.P.; Muchowski J.M. Catalytic hydrogenation of pyrroles at atmospheric pressure. *J. Org. Chem*. **1984**, *49*, 4203-4209.

[4] Arjona, O.; Iradier, F.; Medel, R.; Plumet, J. The Diels-Alder Reaction of Phenylsulfonylacetylene and Furan Derivatives. Normal vs. Tandem 'Pincer' Reactions. *Heterocycles*., **1999**, *50*, 653-656.

[5] Kobayashi, T.; Uchiyama, Y. Neighboring effect of pyrazole rings: regio- and stereoselective Wagner- Meerwein rearrangement in electrophilic addition reactions of norbornadiene-fused pyrazoles. *J. Chem. Soc. Trans.*, **2000**, *1*, 2731-2739.

7. ¹H- and ¹³C-NMR spectra for new compounds.

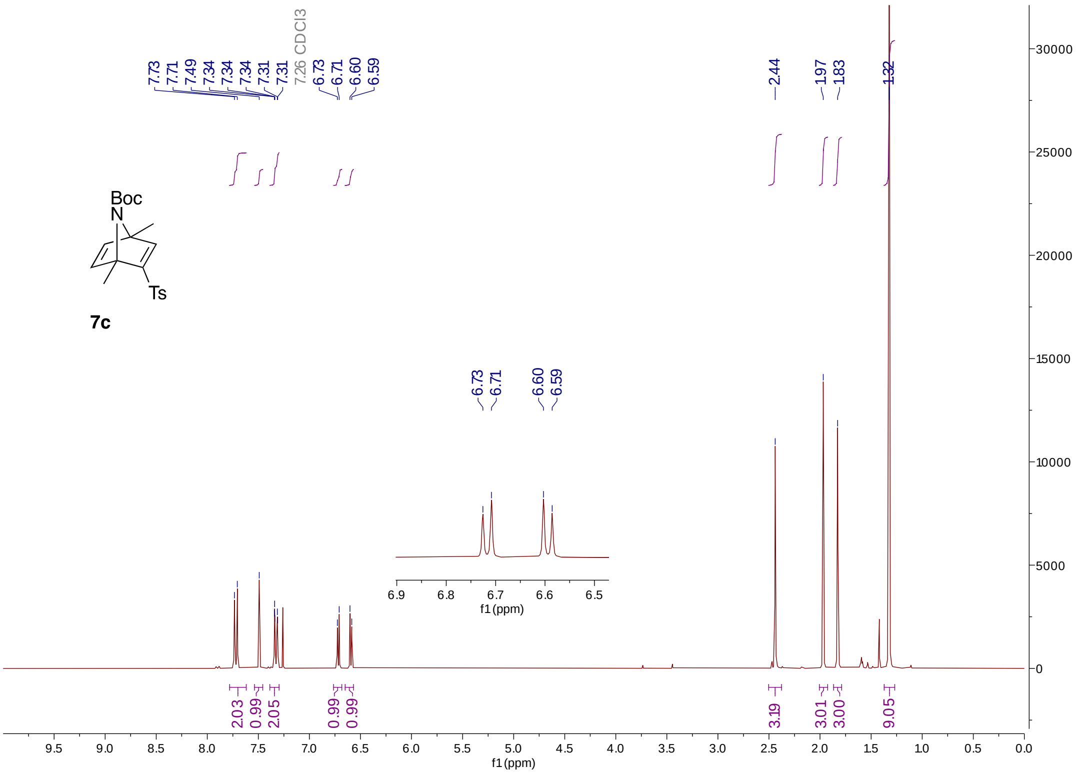

¹H-NMR, 300 MHz in $CDCl_3$ of **7c**.

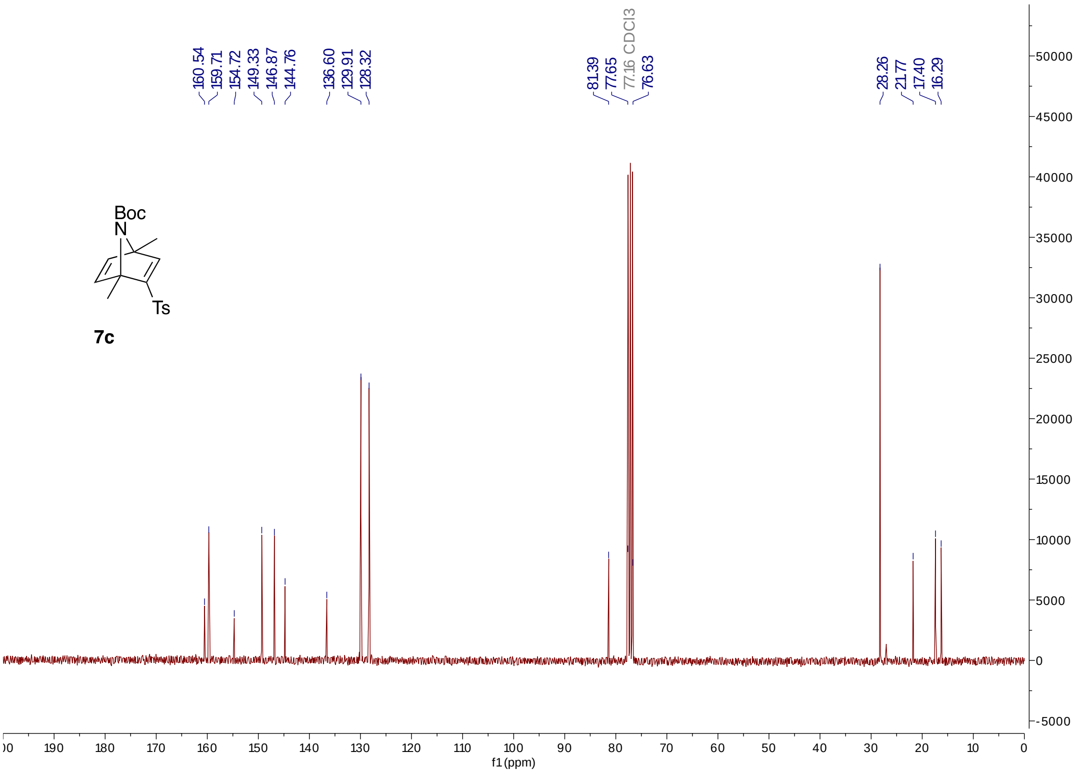

¹³C-NMR, 76 MHz in $CDCl_3$ of **7c**.

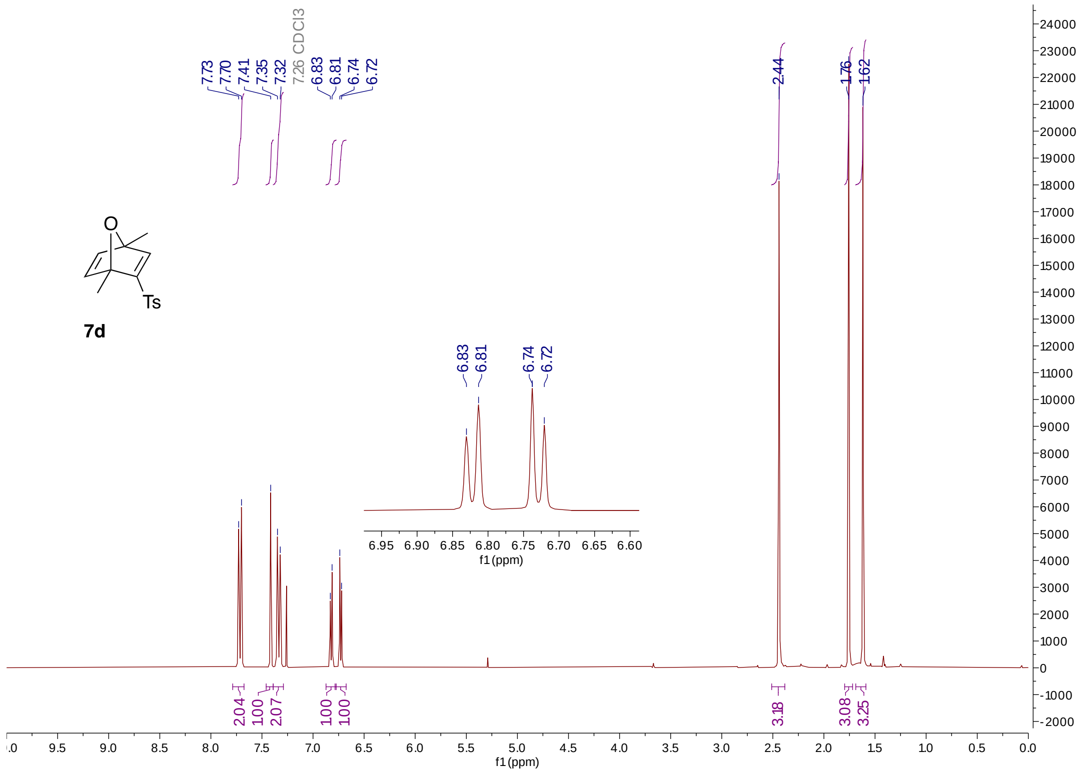

^{1}H-NMR, 300 MHz in $CDCl_3$ of **7d**.

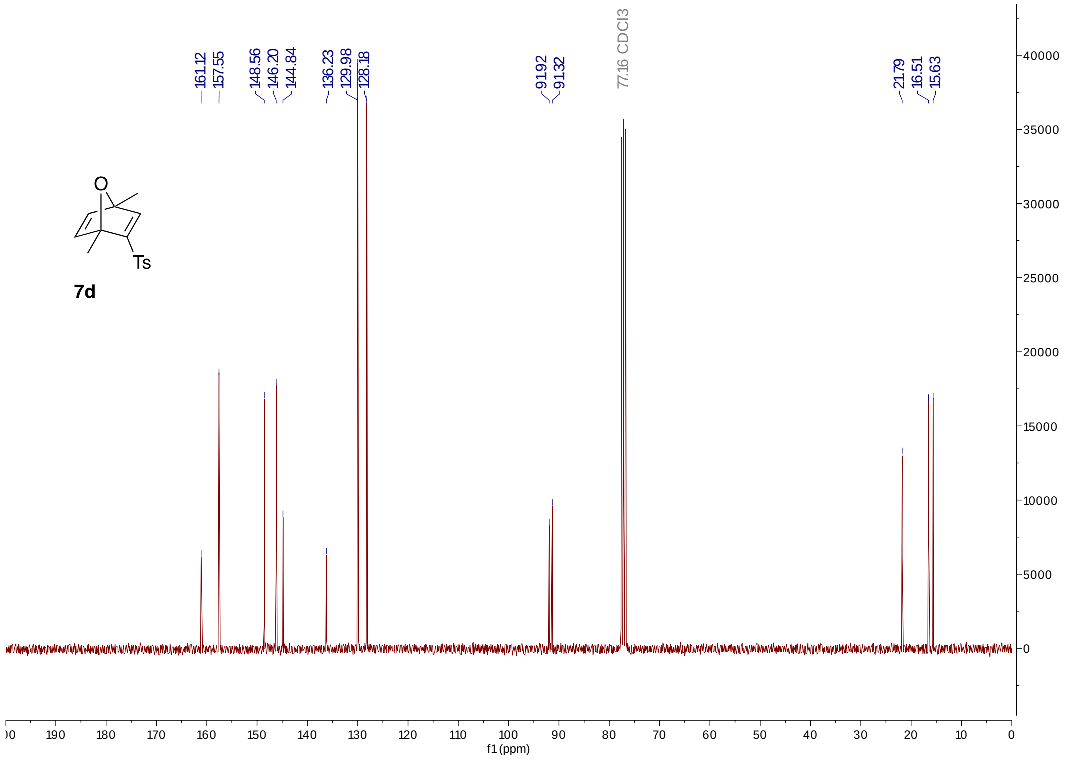

^{13}C-NMR, 76 MHz in $CDCl_3$ of **7d**.

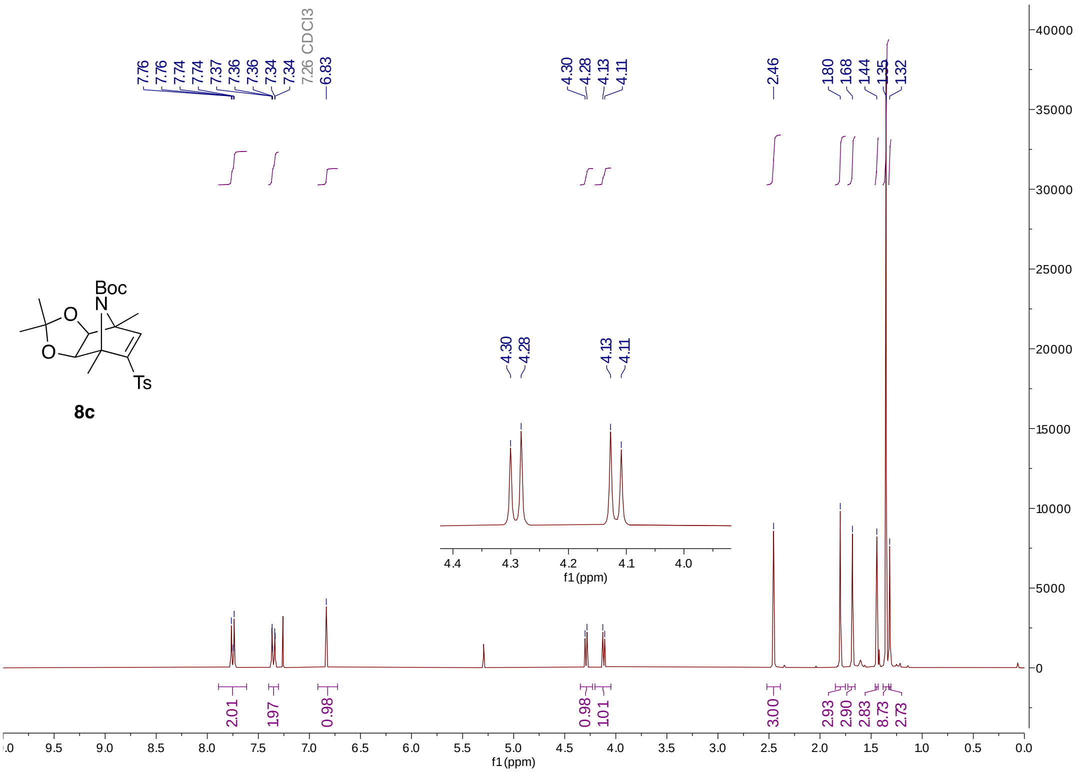

^{1}H-NMR, 300 MHz in $CDCl_3$ of **8c**.

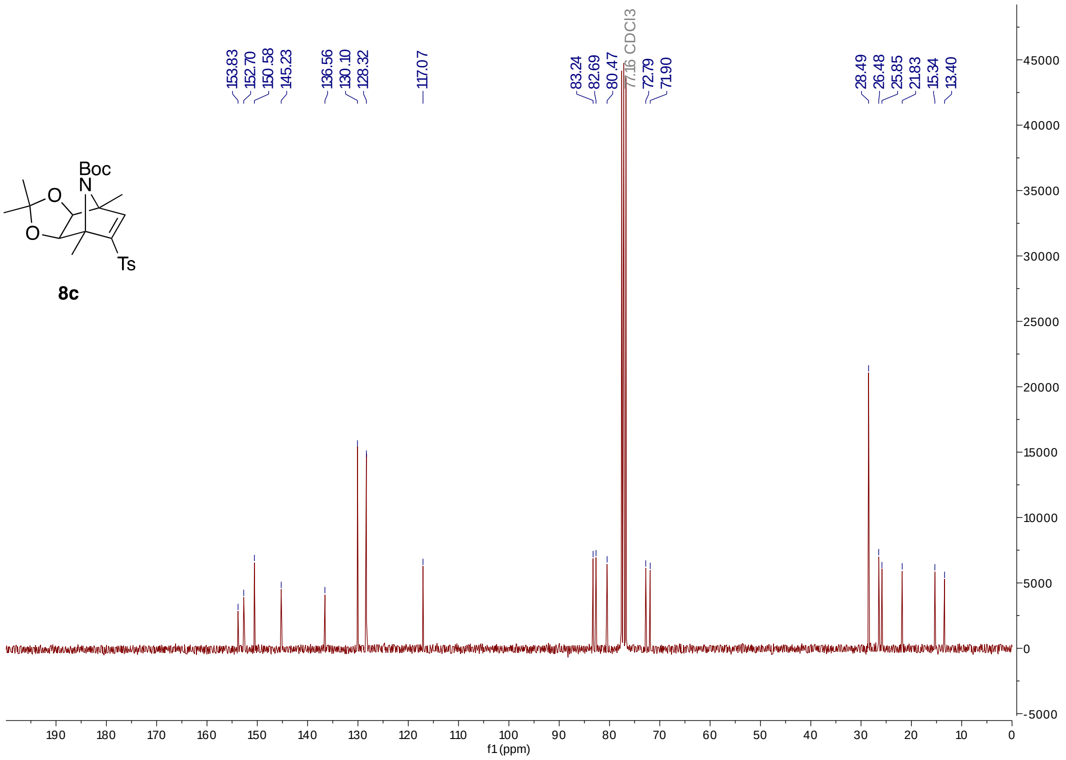

^{13}C-NMR, 76 MHz in $CDCl_3$ of **8c**.

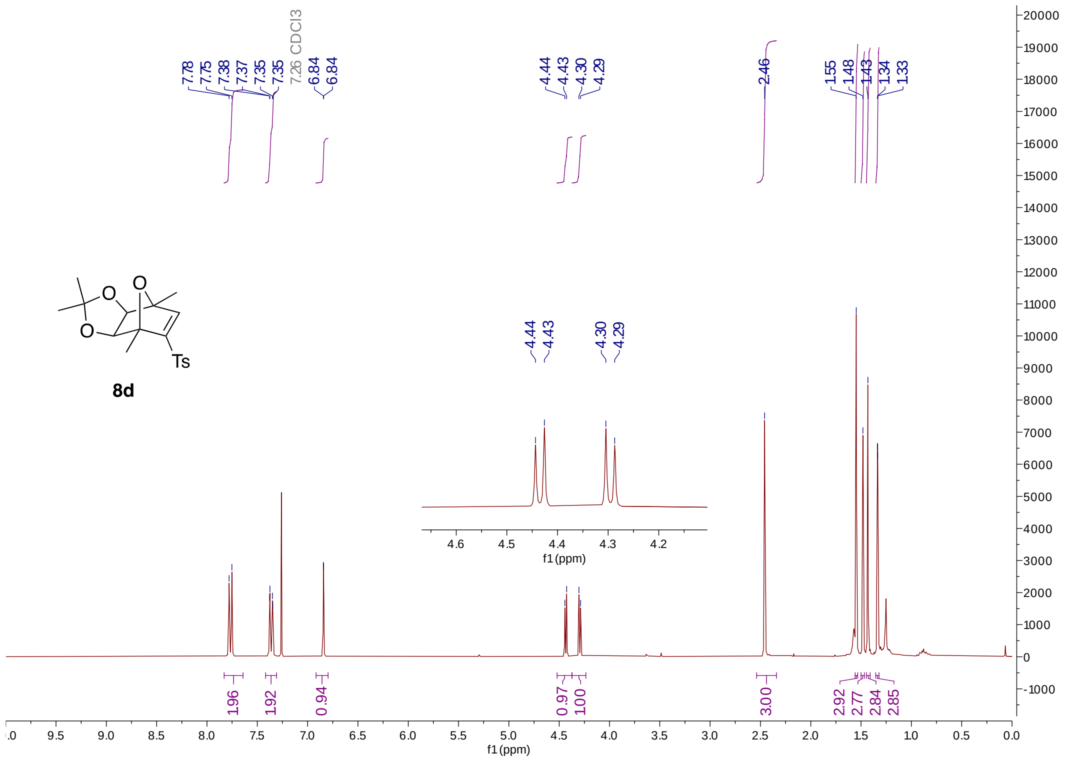

^{1}H-NMR, 300 MHz in $CDCl_3$ of **8d**.

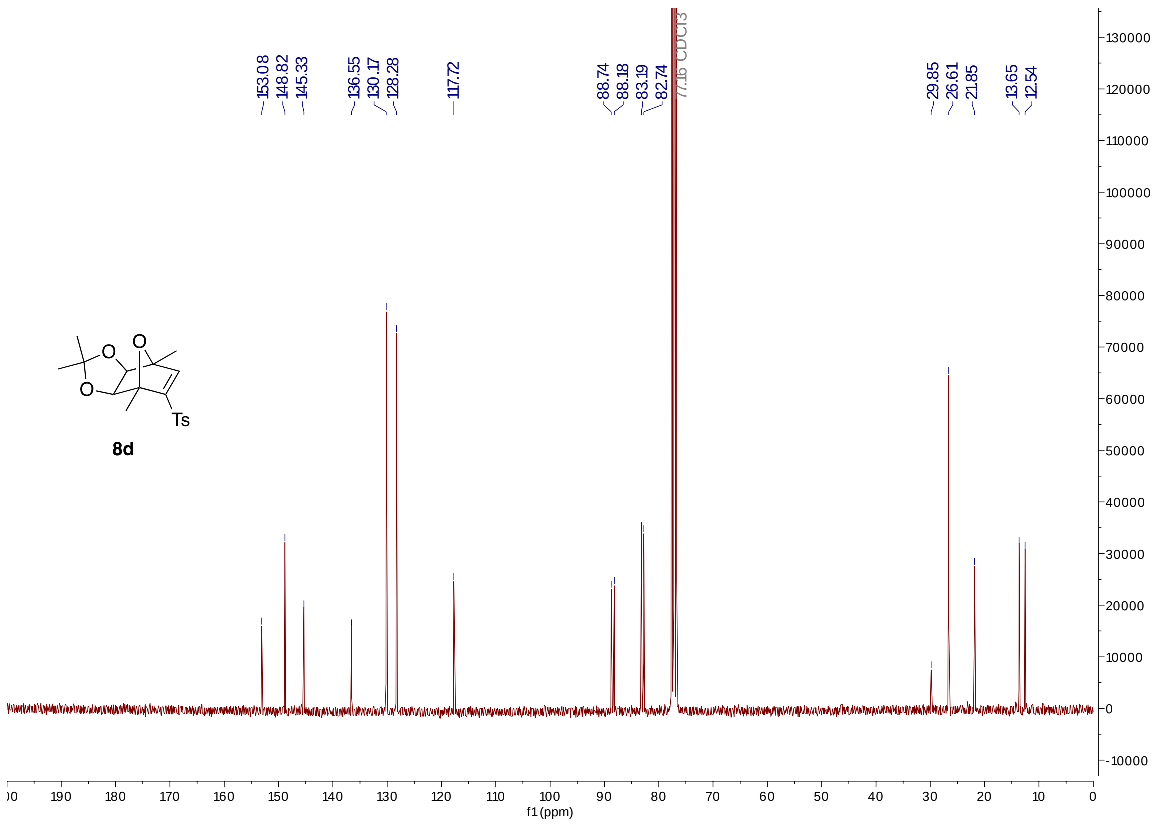

^{13}C-NMR, 76 MHz in $CDCl_3$ of **8d**.

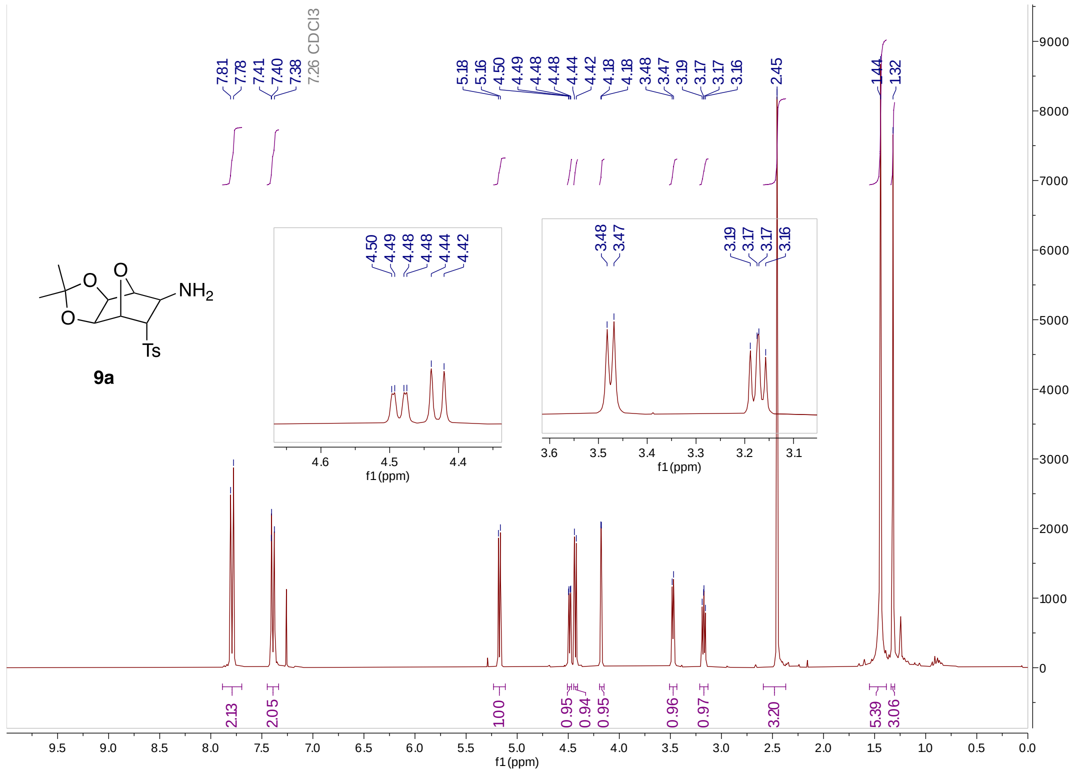

^{1}H-NMR, 300 MHz in $CDCl_3$ of **9a**.

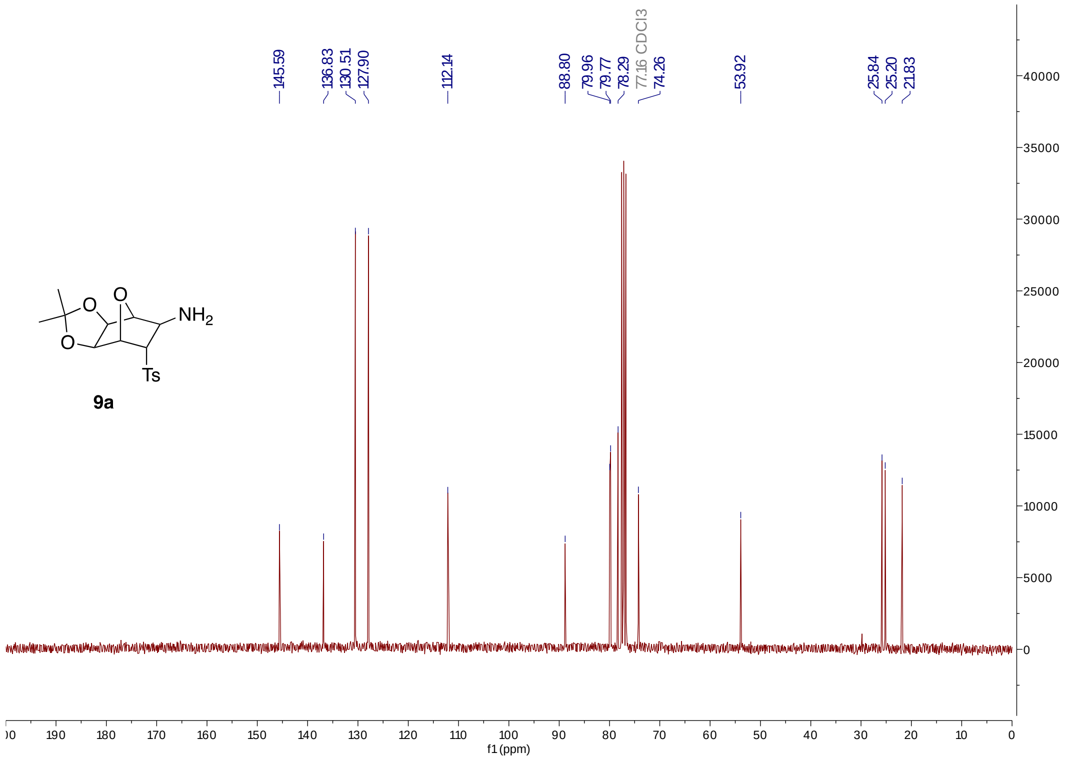

^{13}C-NMR, 76 MHz in $CDCl_3$ of **9a**.

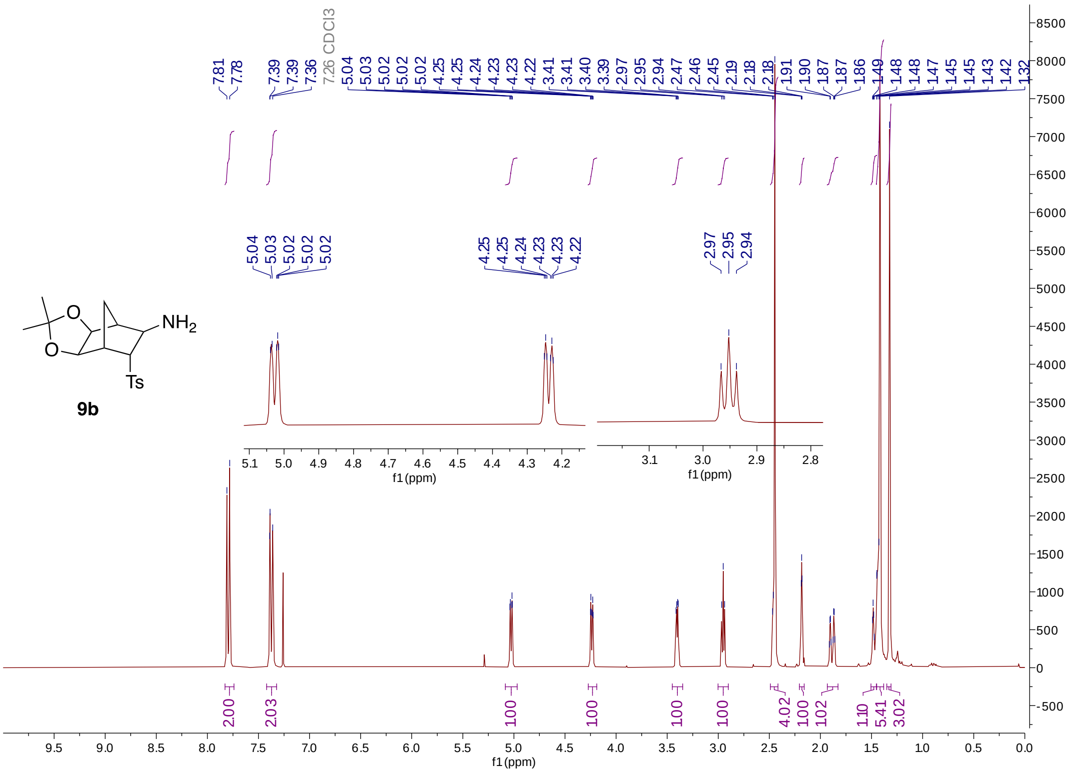

^{1}H-NMR, 300 MHz in $CDCl_3$ of **9b**.

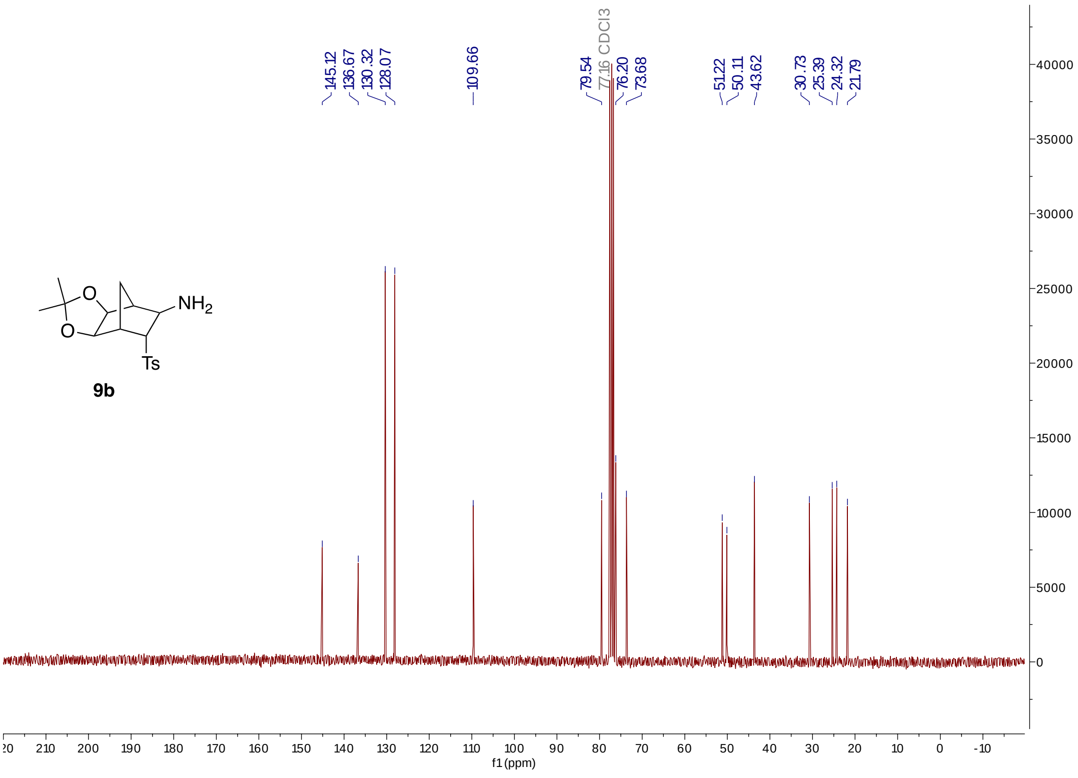

^{13}C-NMR, 76 MHz in $CDCl_3$ of **9b**.

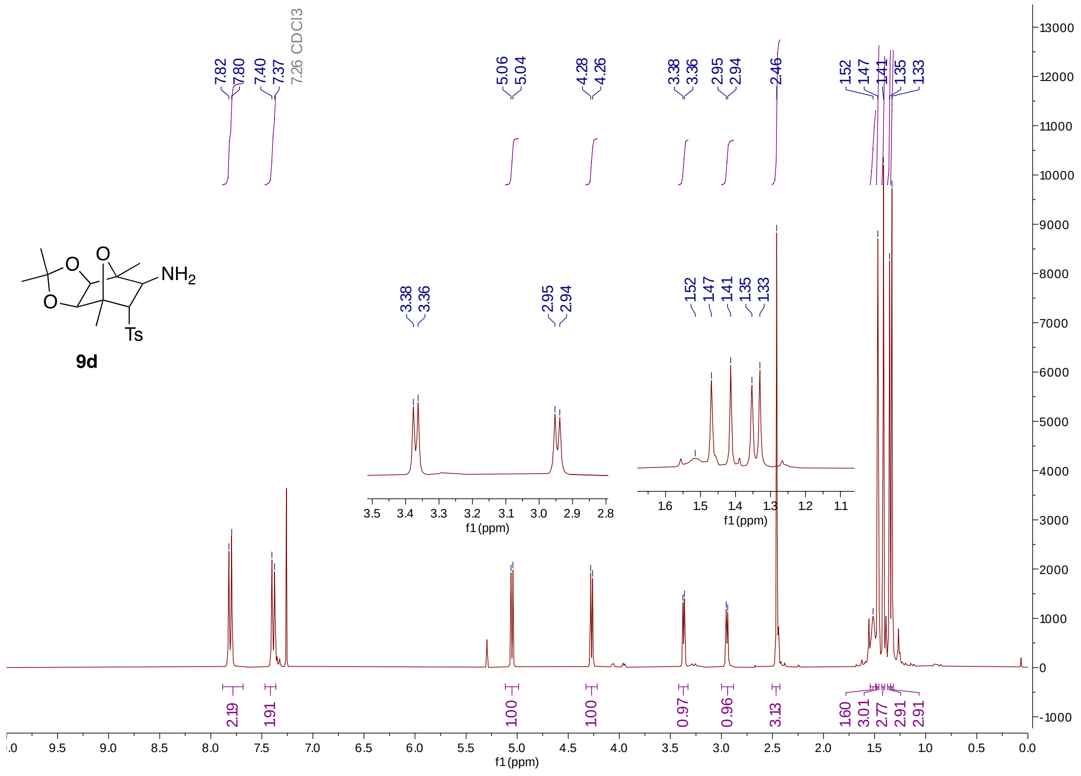

^{1}H-NMR, 300 MHz in $CDCl_3$ of **9d**.

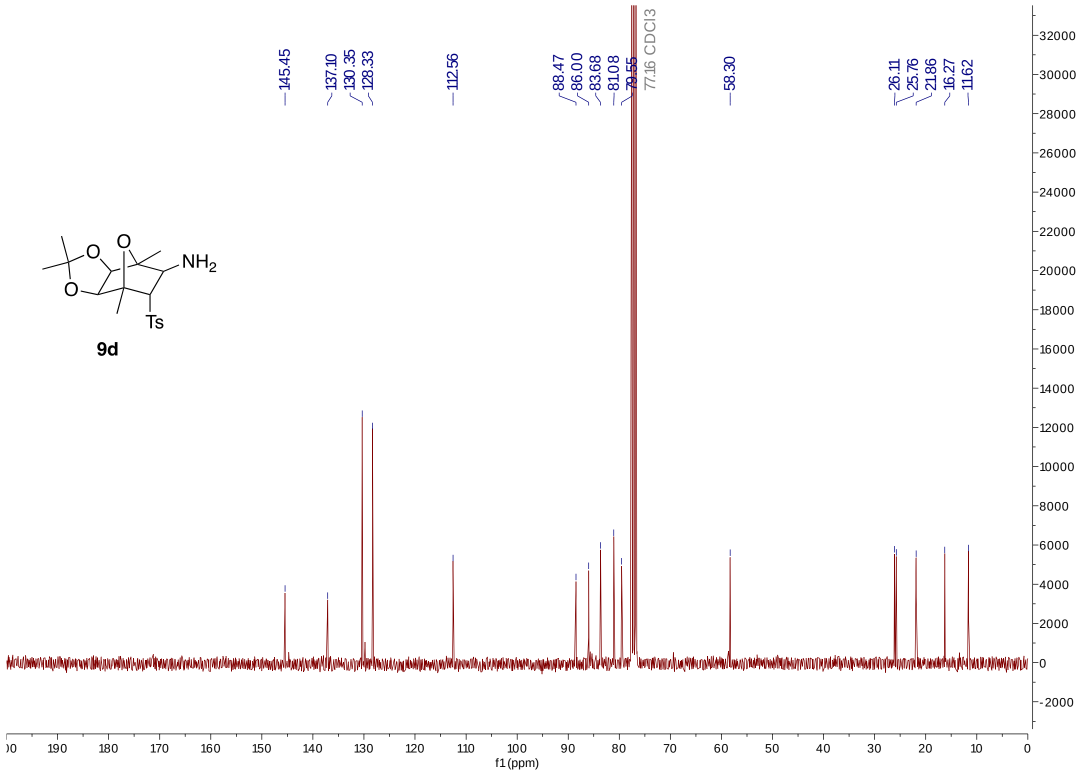

^{13}C-NMR, 76 MHz in $CDCl_3$ of **9d**.

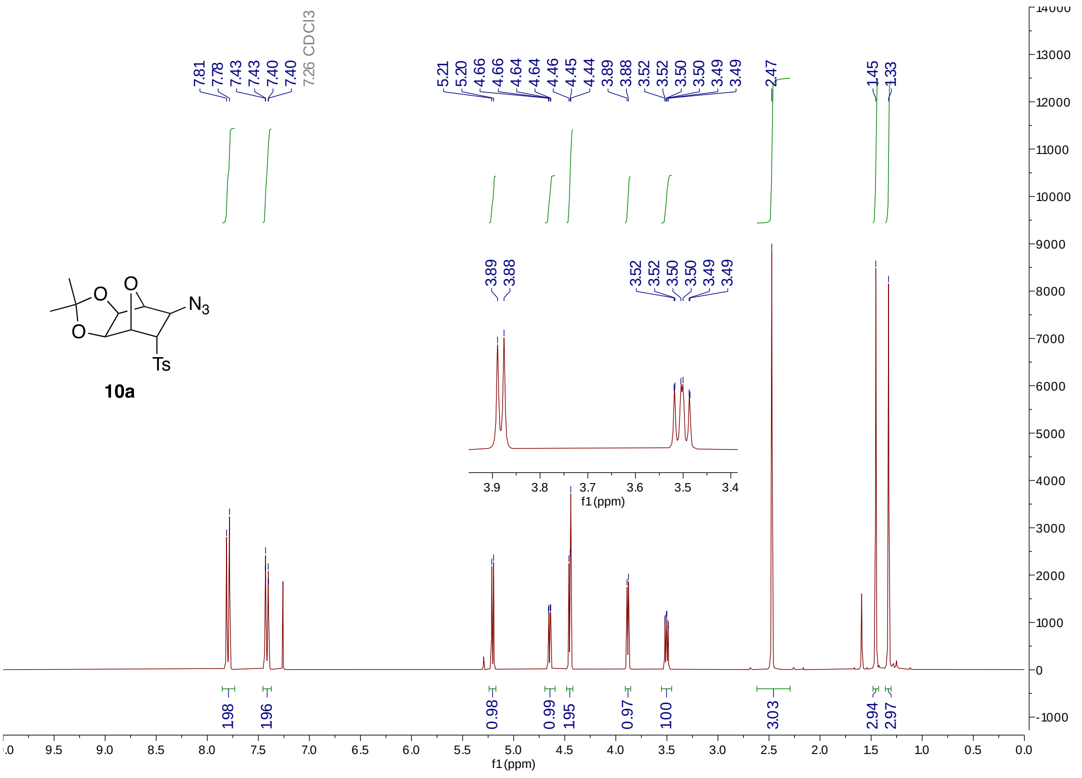

^{1}H-NMR, 300 MHz in $CDCl_3$ of **10a**.

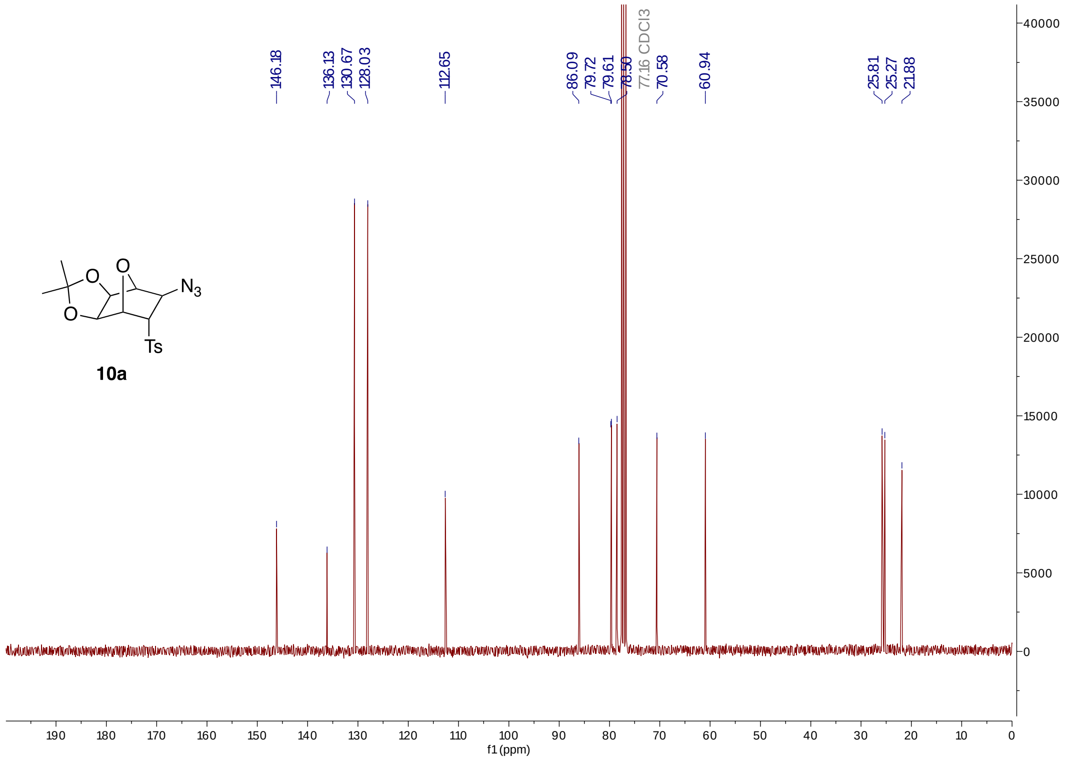

^{13}C-NMR, 76 MHz in $CDCl_3$ of **10a**.

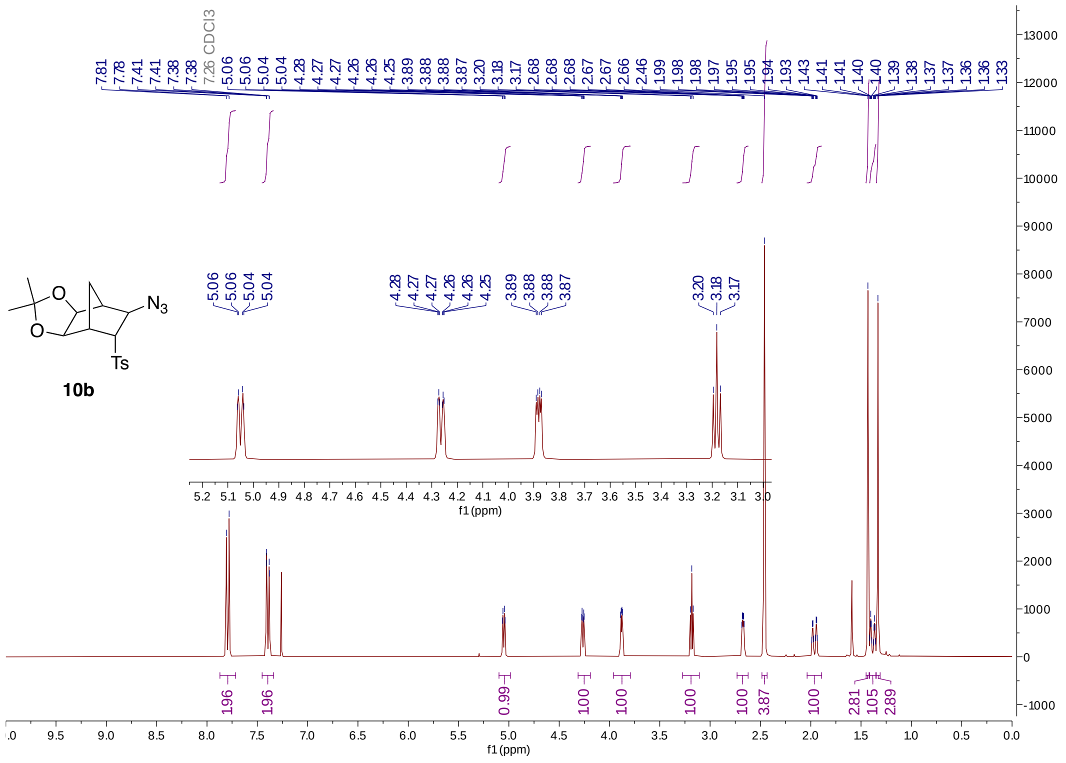

^{1}H-NMR, 300 MHz in $CDCl_3$ of **10b**.

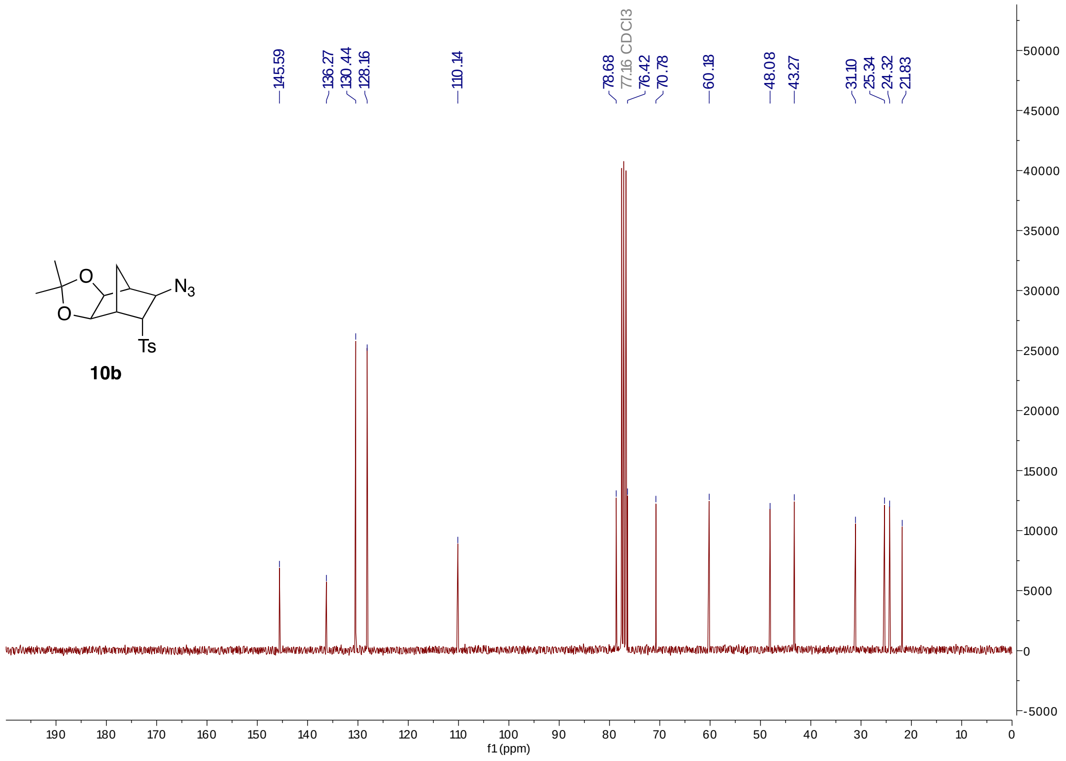

^{13}C-NMR, 76 MHz in $CDCl_3$ of **10b**.

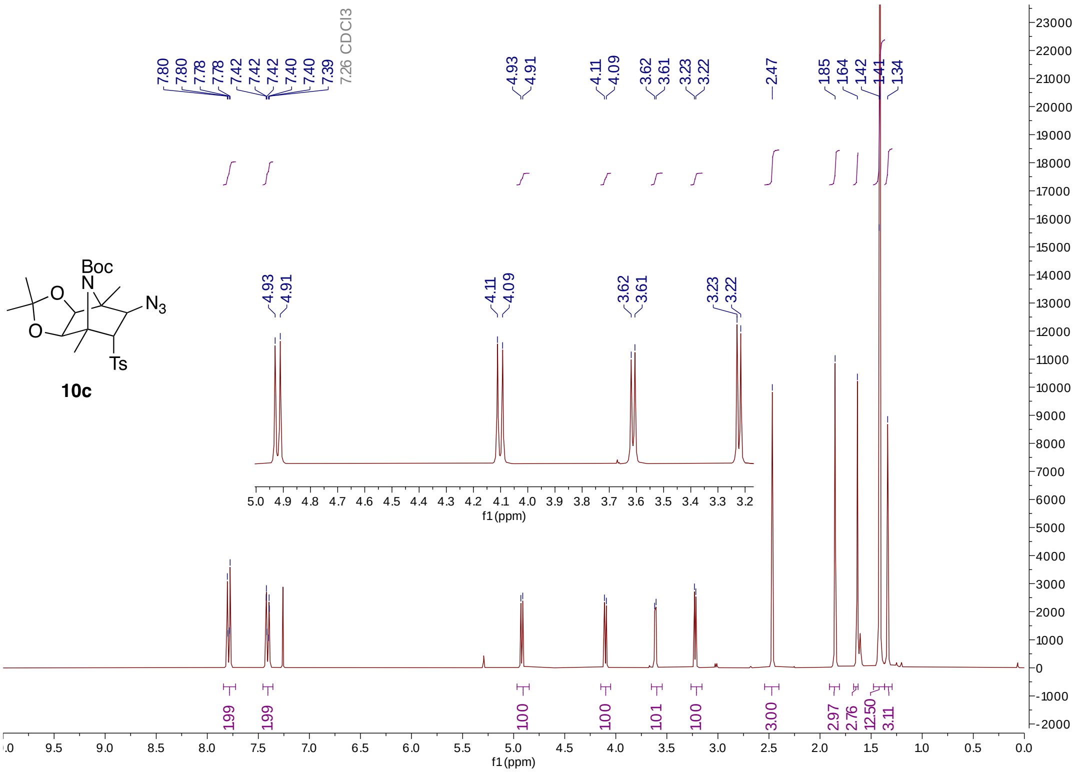

^{1}H-NMR, 300 MHz in $CDCl_3$ of **10c**.

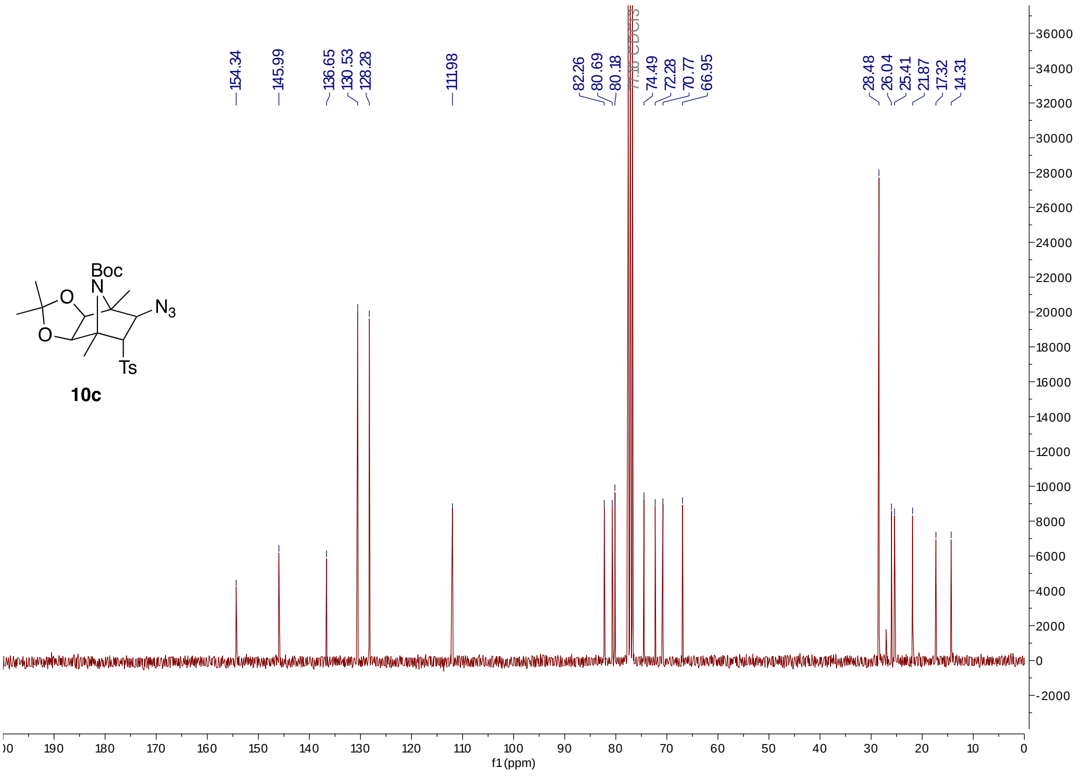

^{13}C-NMR, 76 MHz in $CDCl_3$ of **10c**.

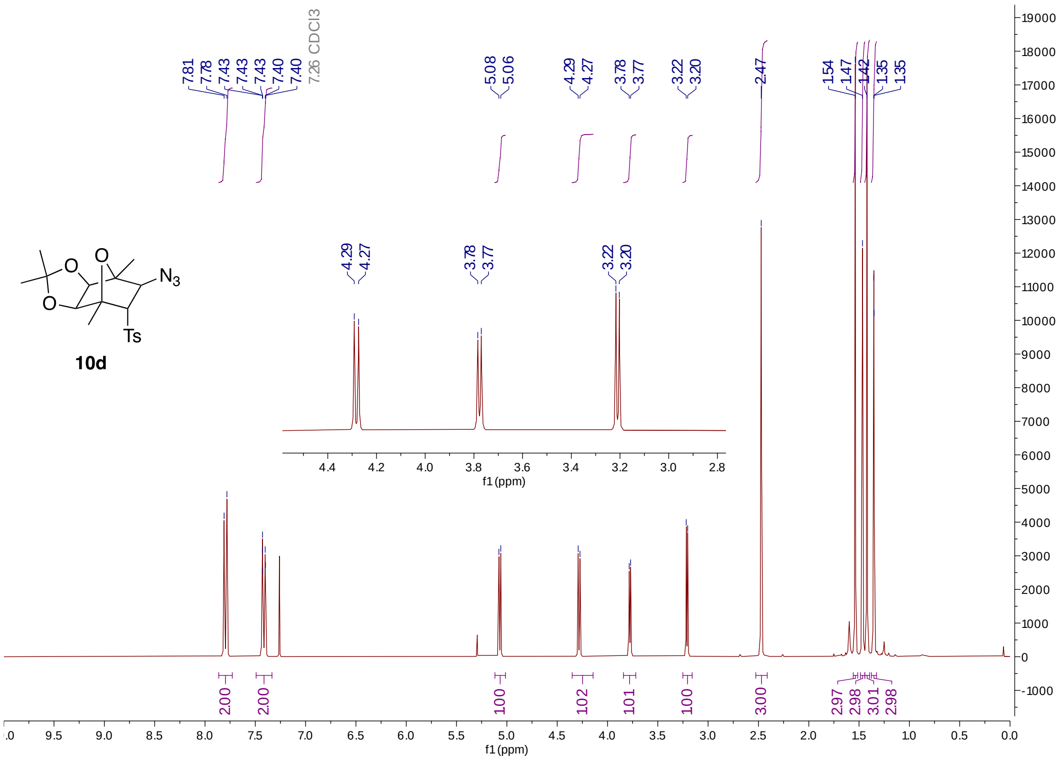

^{1}H-NMR, 300 MHz in $CDCl_3$ of **10d**.

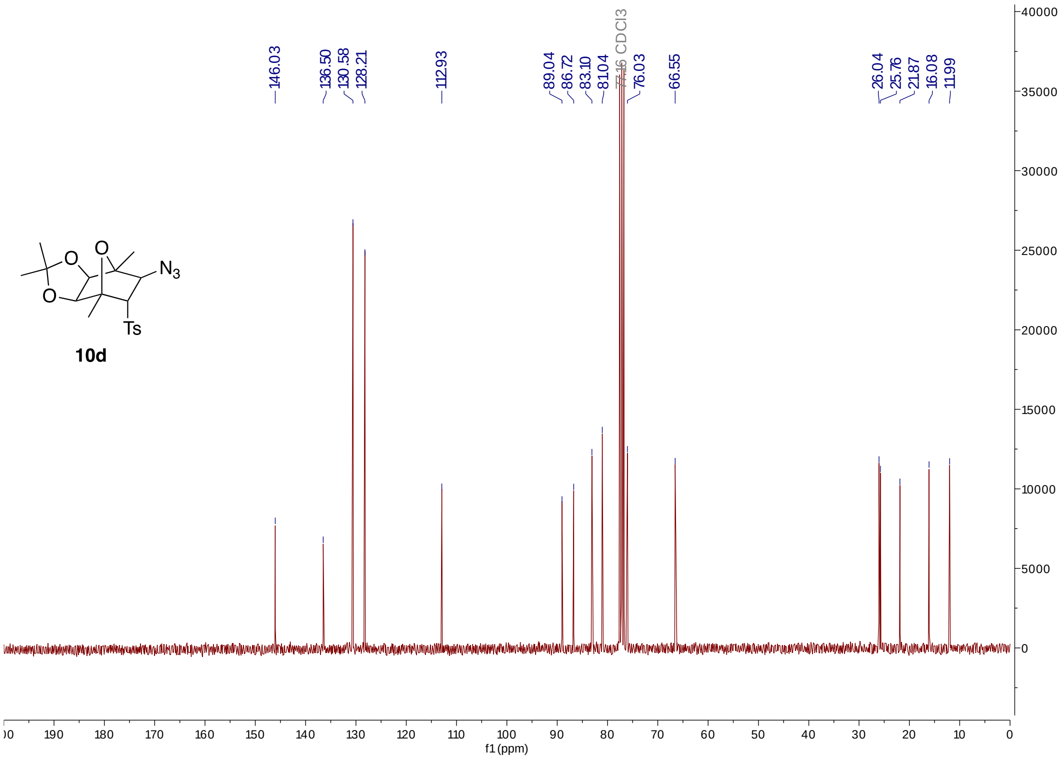

^{13}C-NMR, 76 MHz in $CDCl_3$ of **10d**.

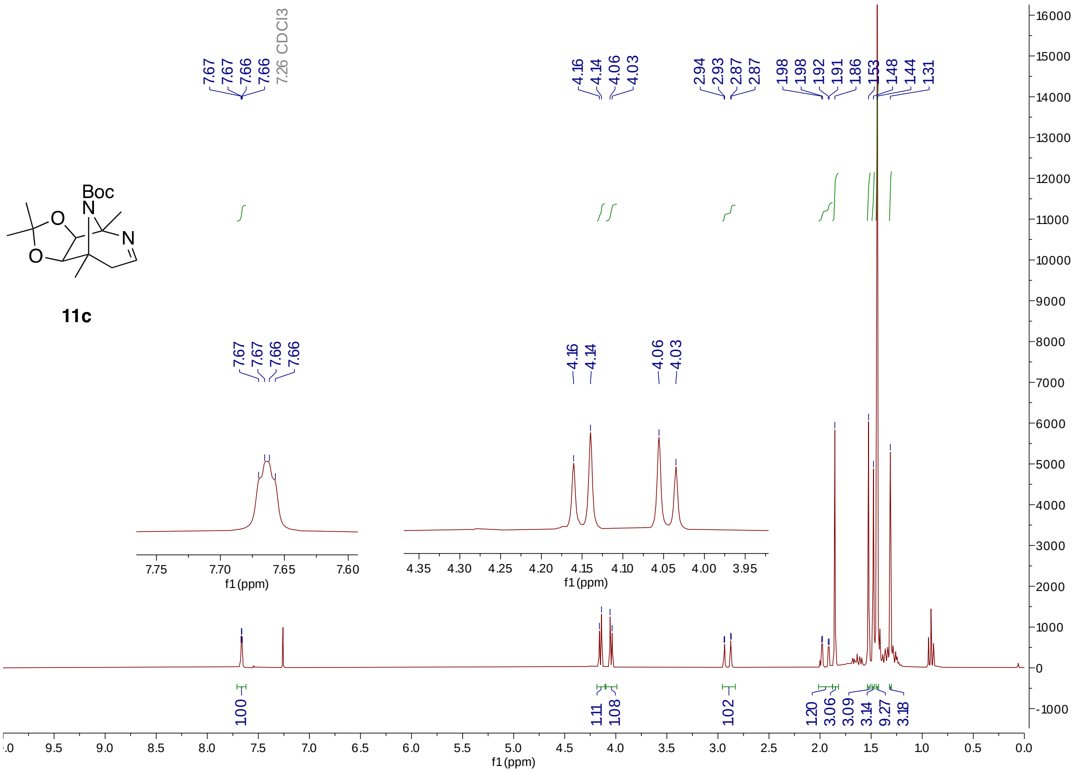

^{1}H-NMR, 300 MHz in $CDCl_3$ of **11c**.

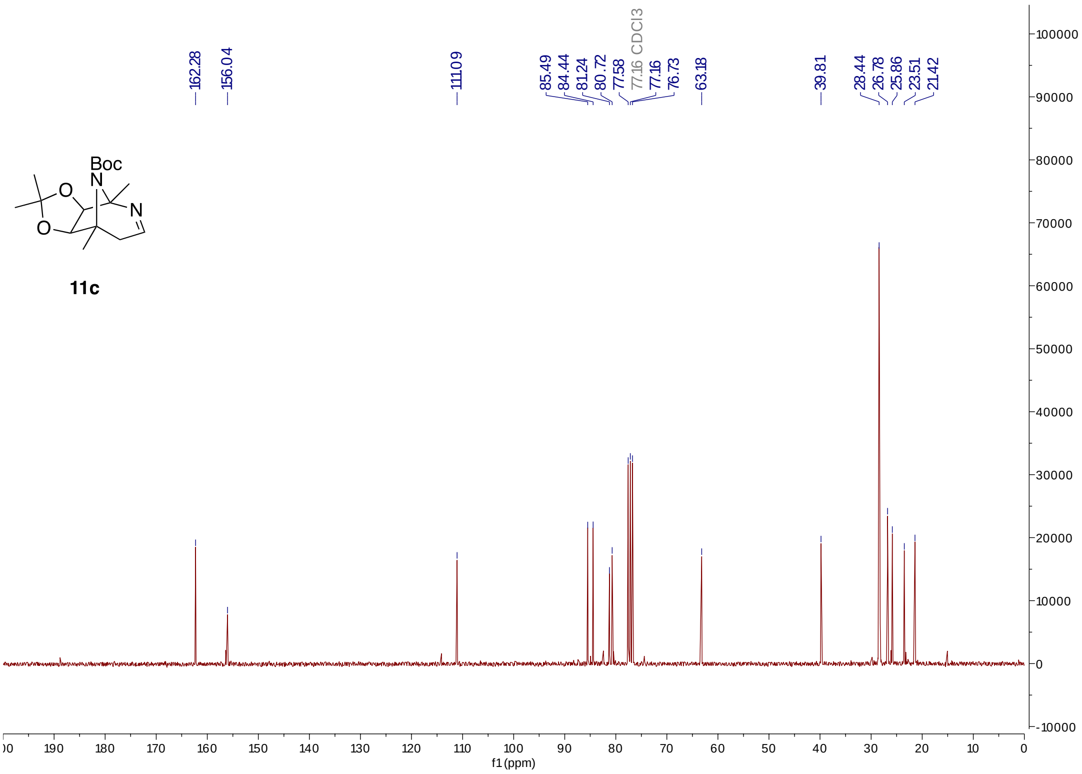

^{13}C-NMR, 76 MHz in $CDCl_3$ of **11c**.

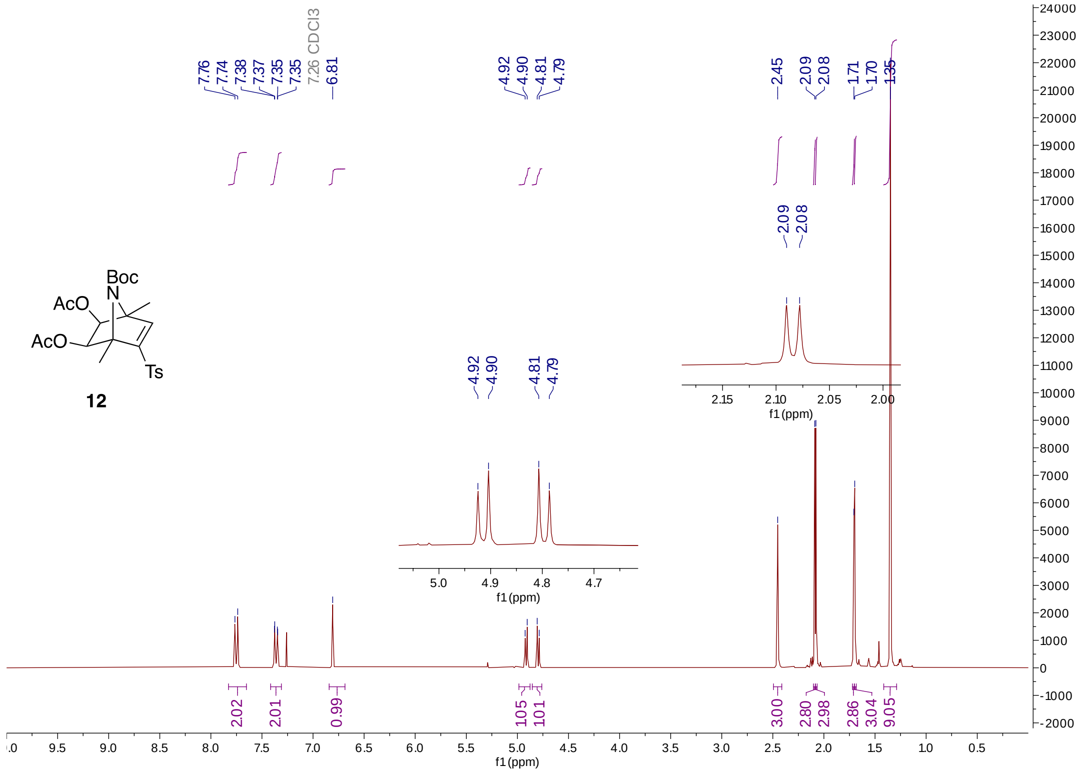

^{1}H-NMR, 300 MHz in $CDCl_3$ of **12**.

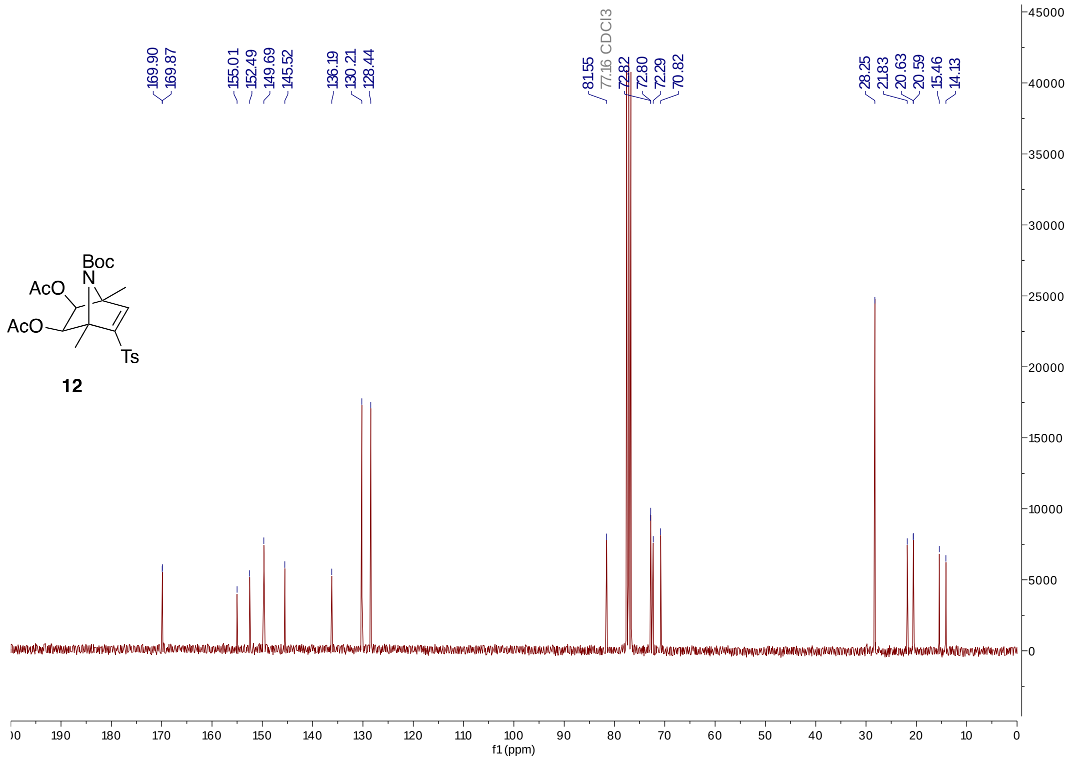

^{13}C-NMR, 76 MHz in $CDCl_3$ of **12**.

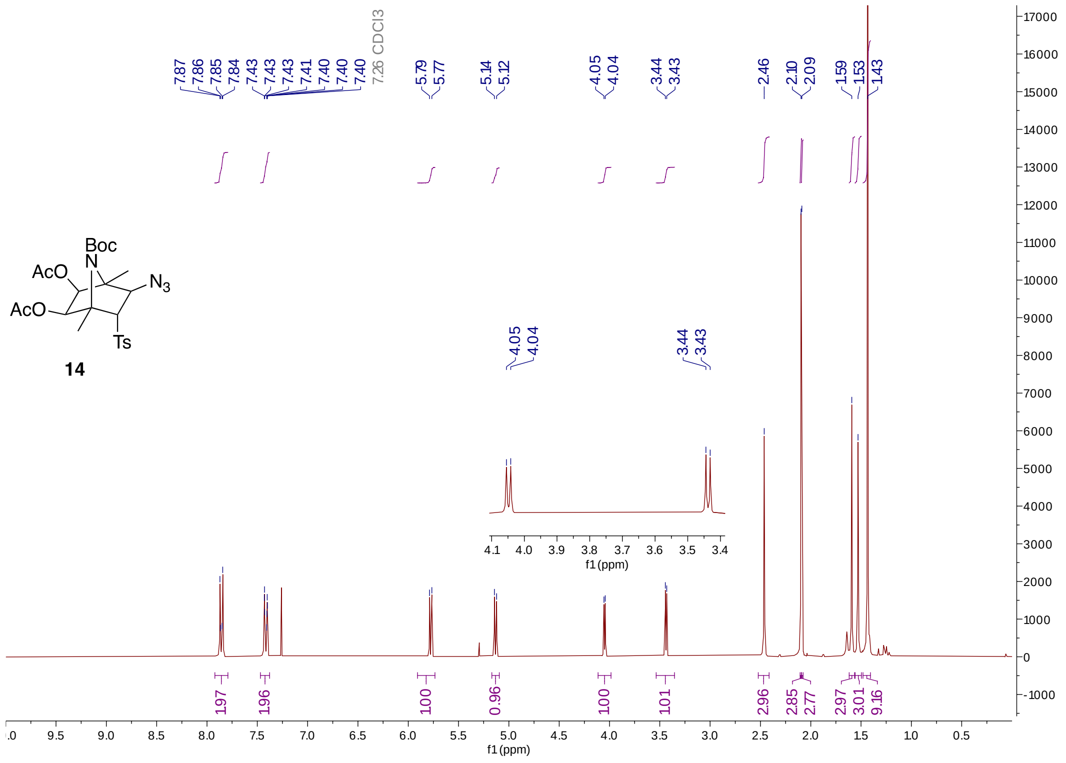

^{1}H-NMR, 300 MHz in $CDCl_3$ of **14**.

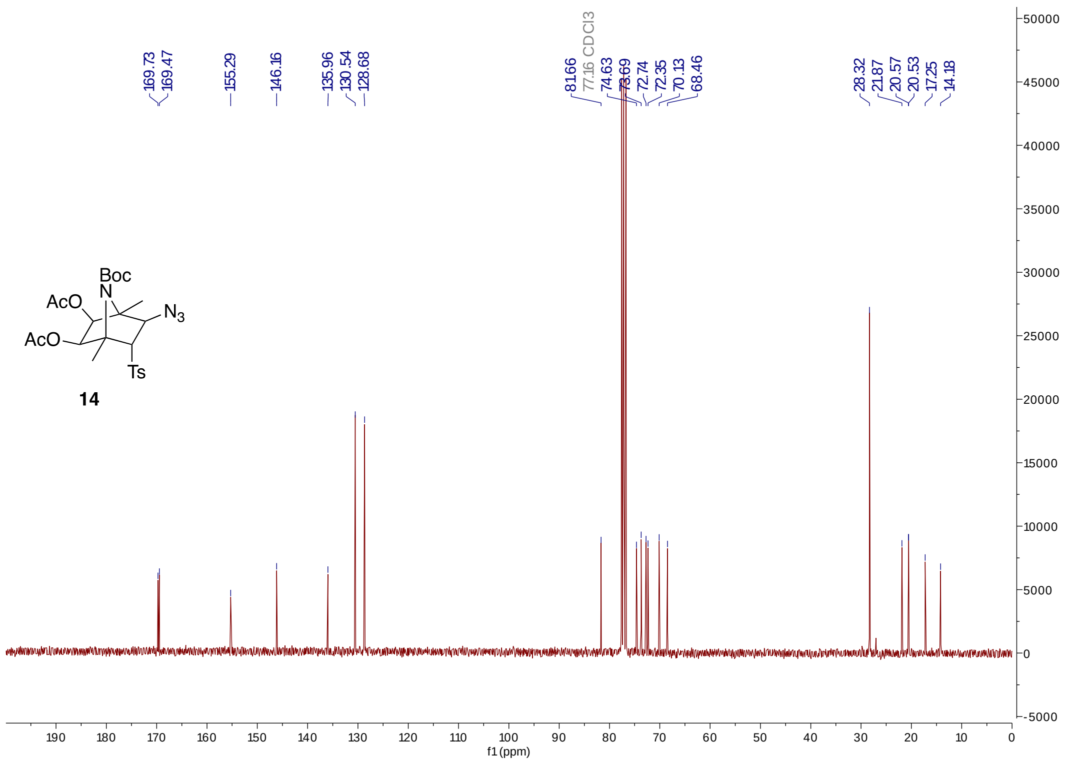

^{13}C-NMR, 76 MHz in $CDCl_3$ of **14**.

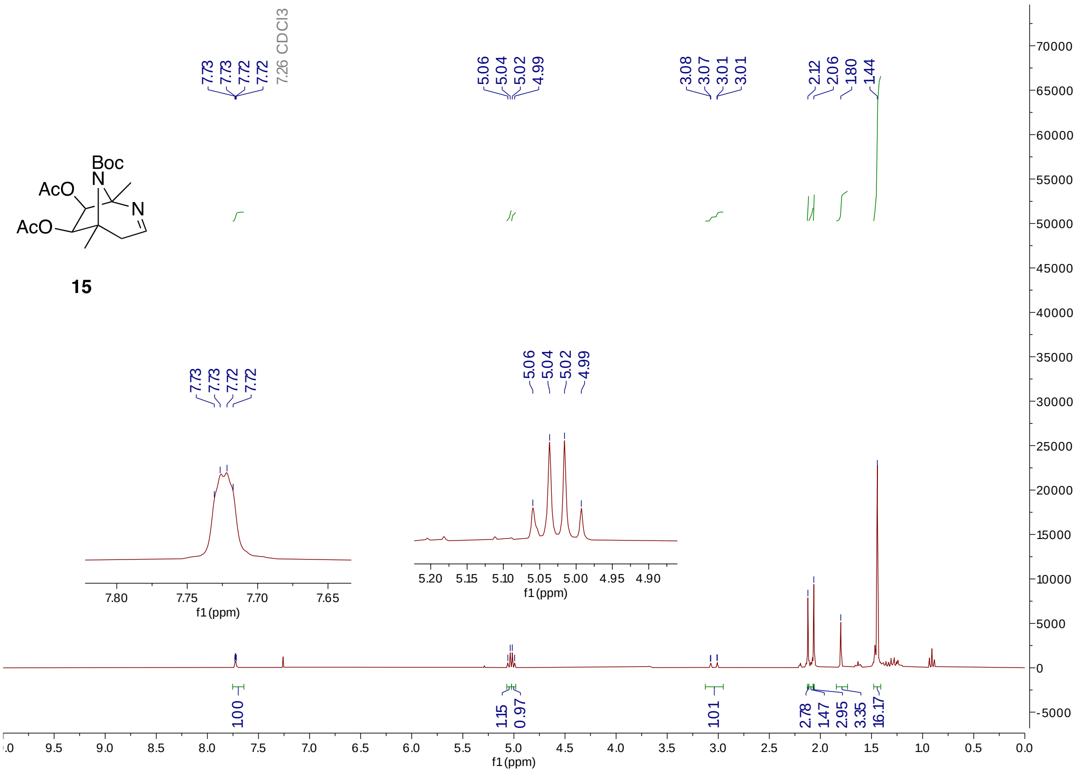

^{1}H-NMR, 300 MHz in $CDCl_3$ of **15**.

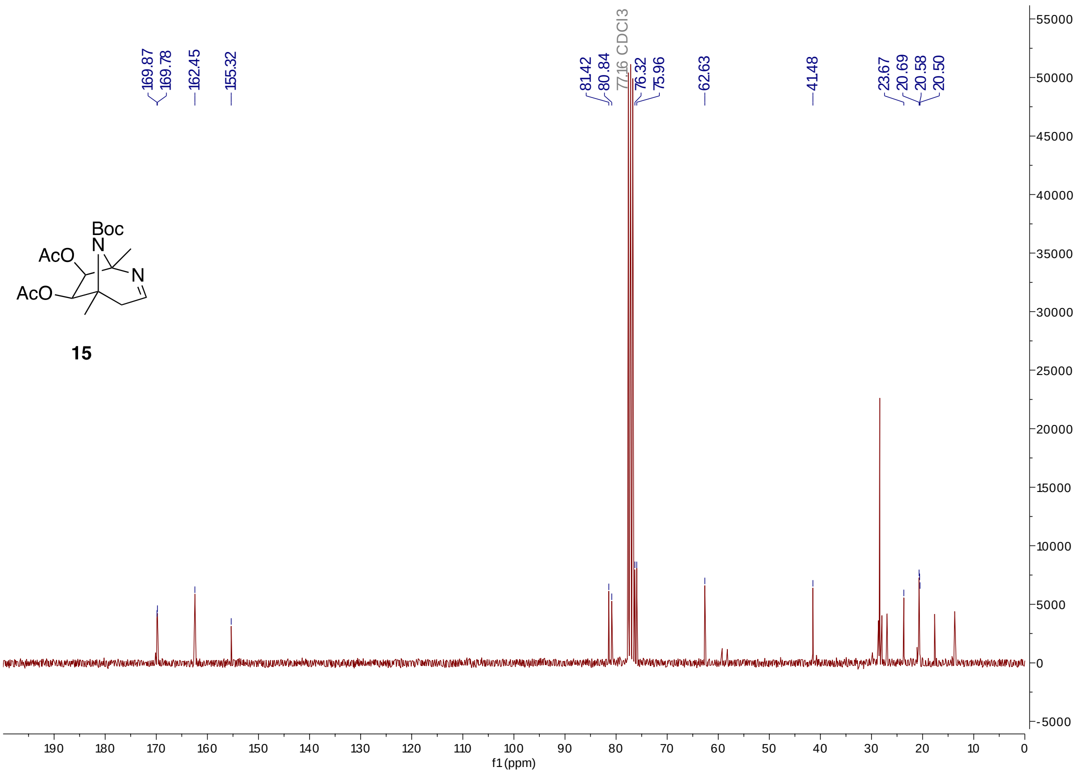

^{13}C-NMR, 76 MHz in $CDCl_3$ of **15**.

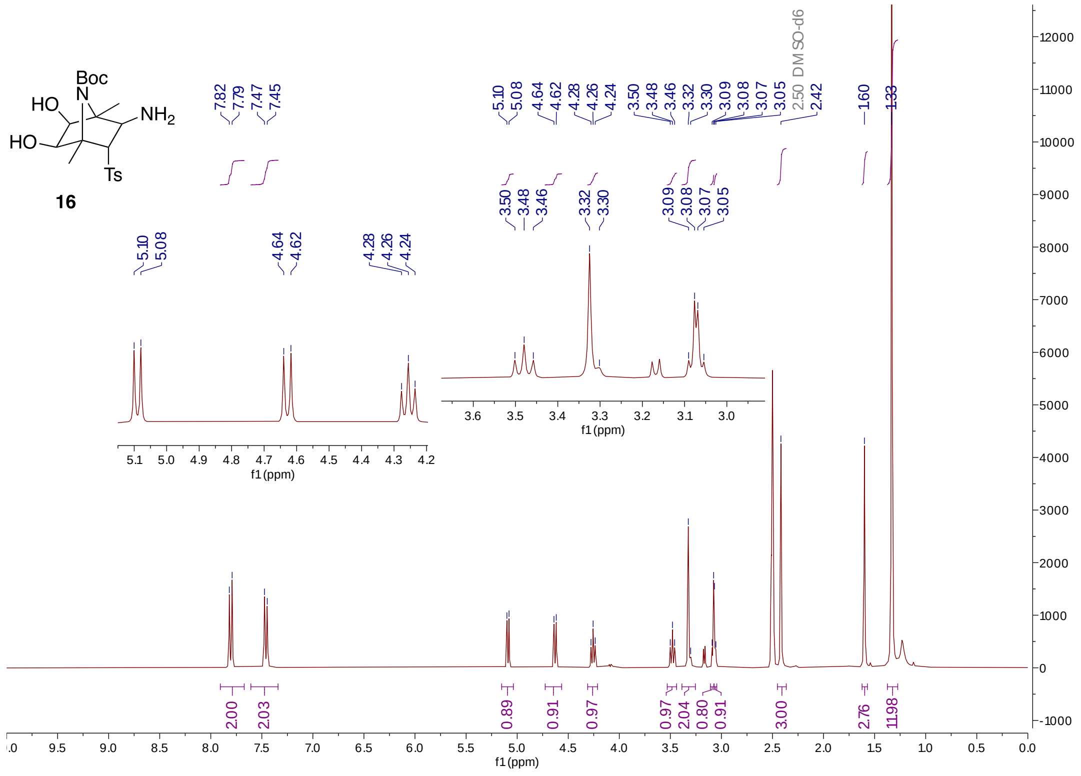

^{1}H-NMR, 300 MHz in DMSO-d_6 of **16**.

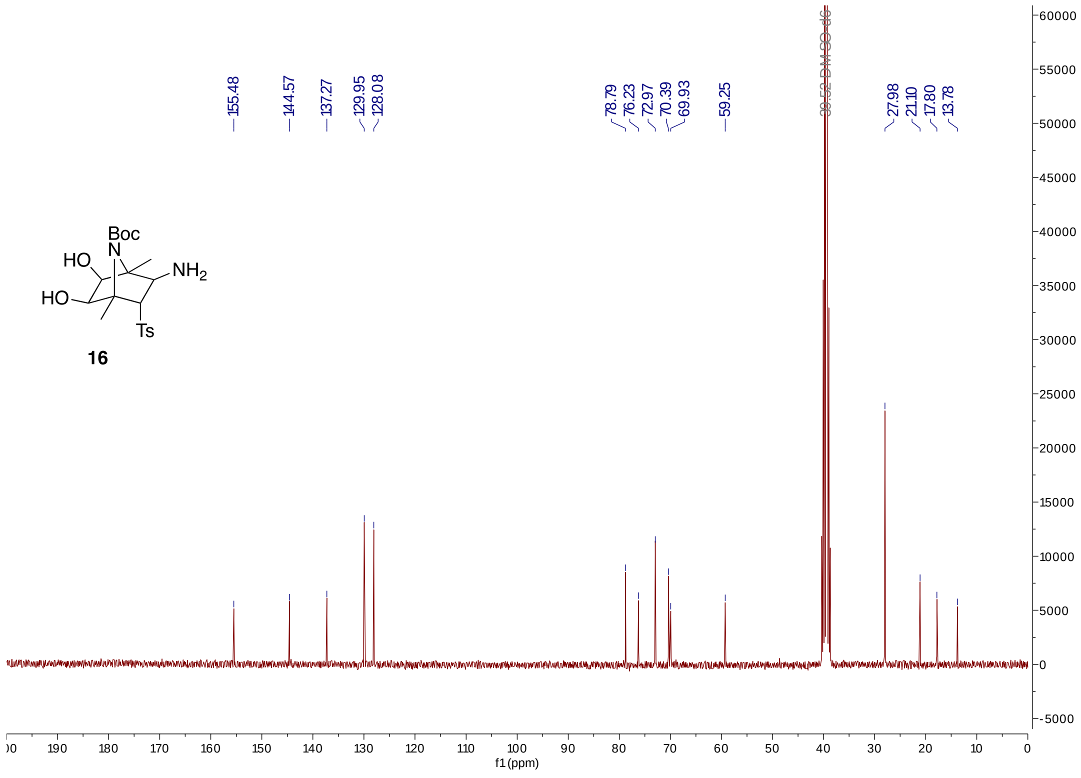

^{13}C-NMR, 76 MHz in DMSO-d_6 of **16**.

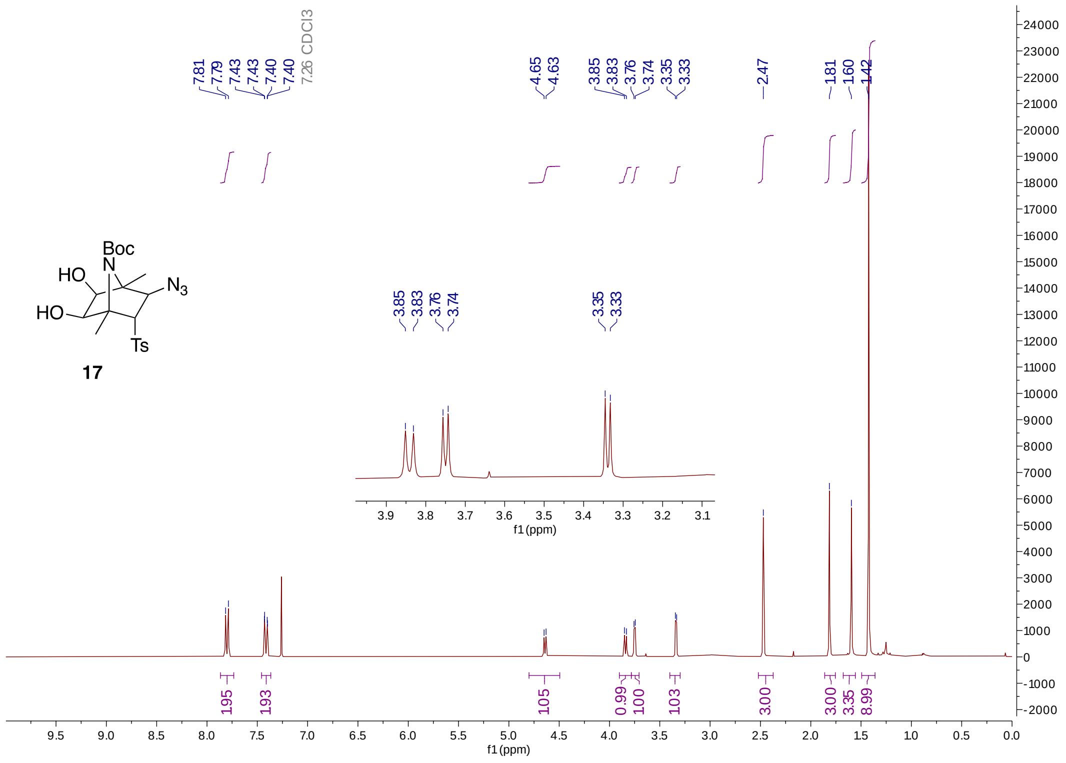

^{1}H-NMR, 300 MHz in $CDCl_3$ of **17**.

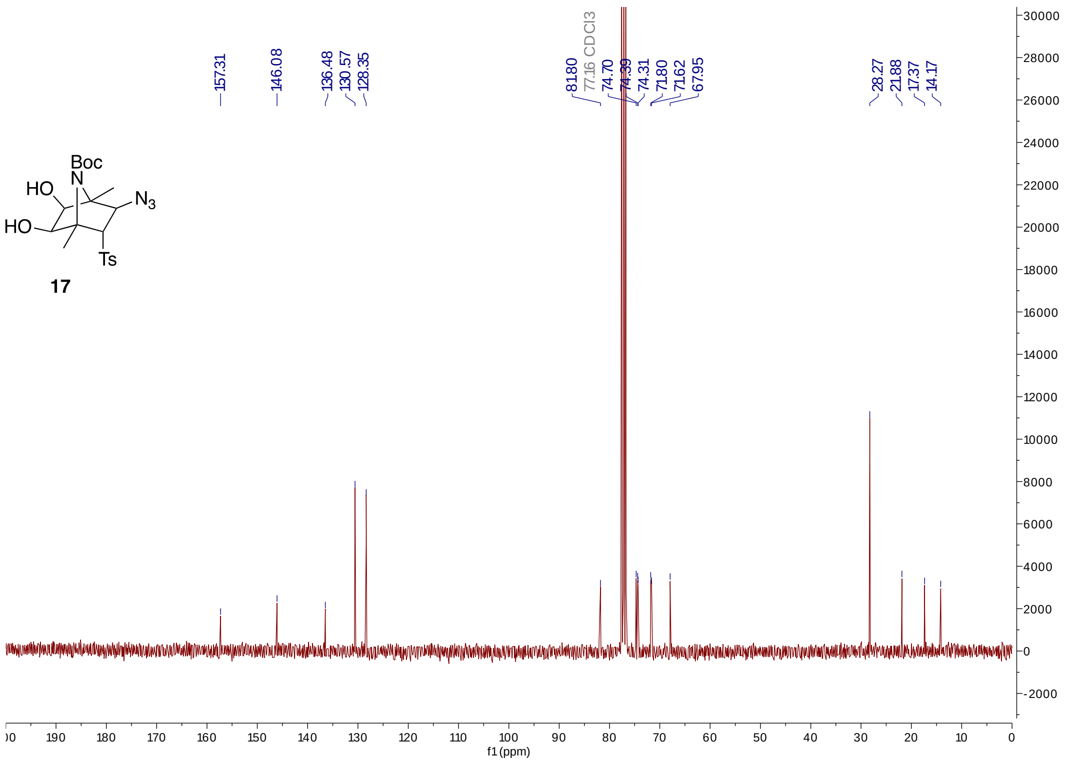

^{13}C-NMR, 76 MHz in $CDCl_3$ of **17**.

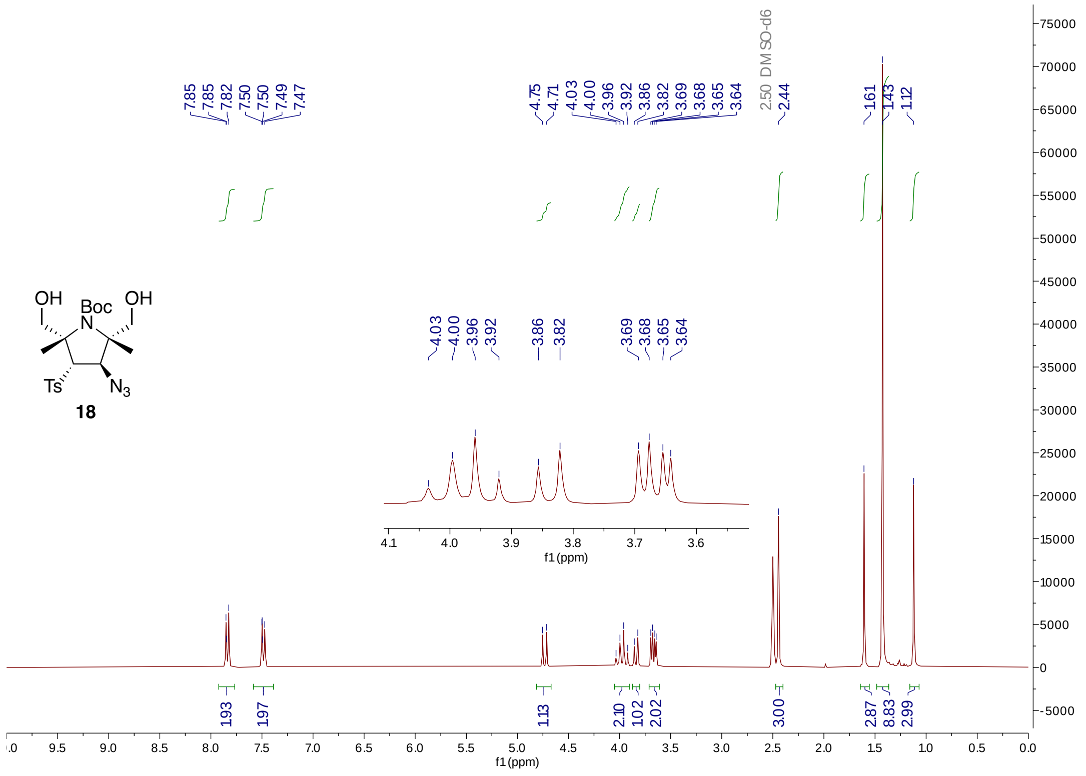

^{1}H-NMR, 300 MHz in DMSO-d_6 of **18**.

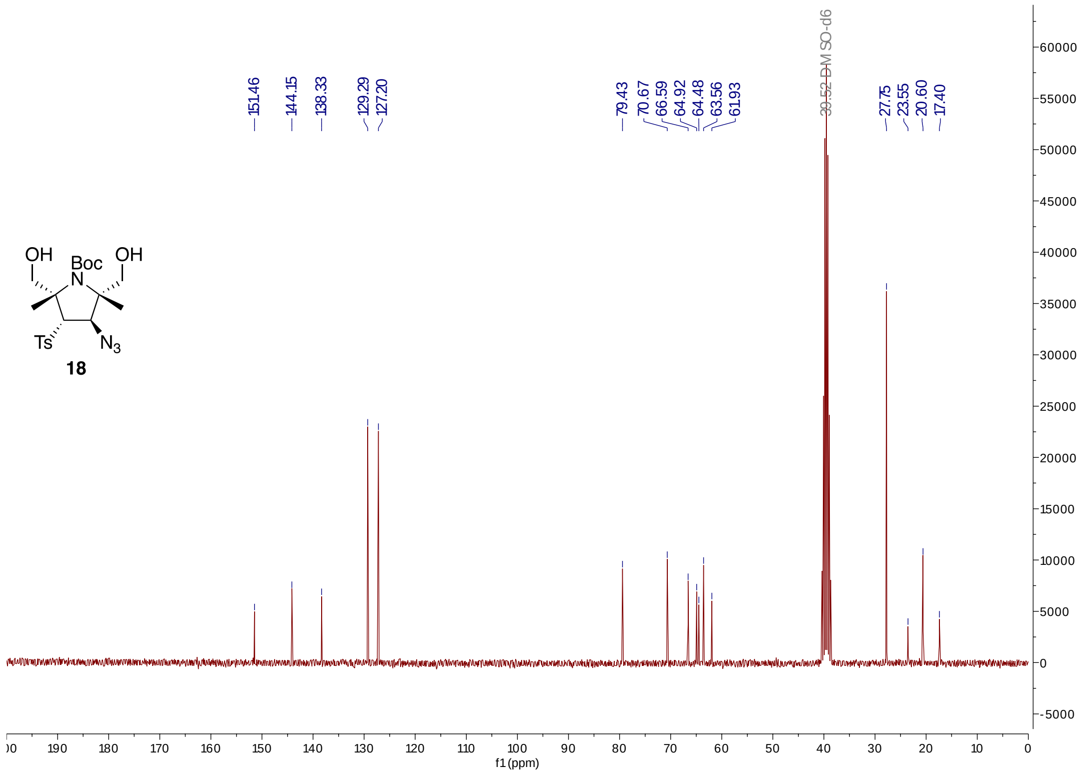

^{13}C-NMR, 76 MHz in DMSO-d_6 of **18**.

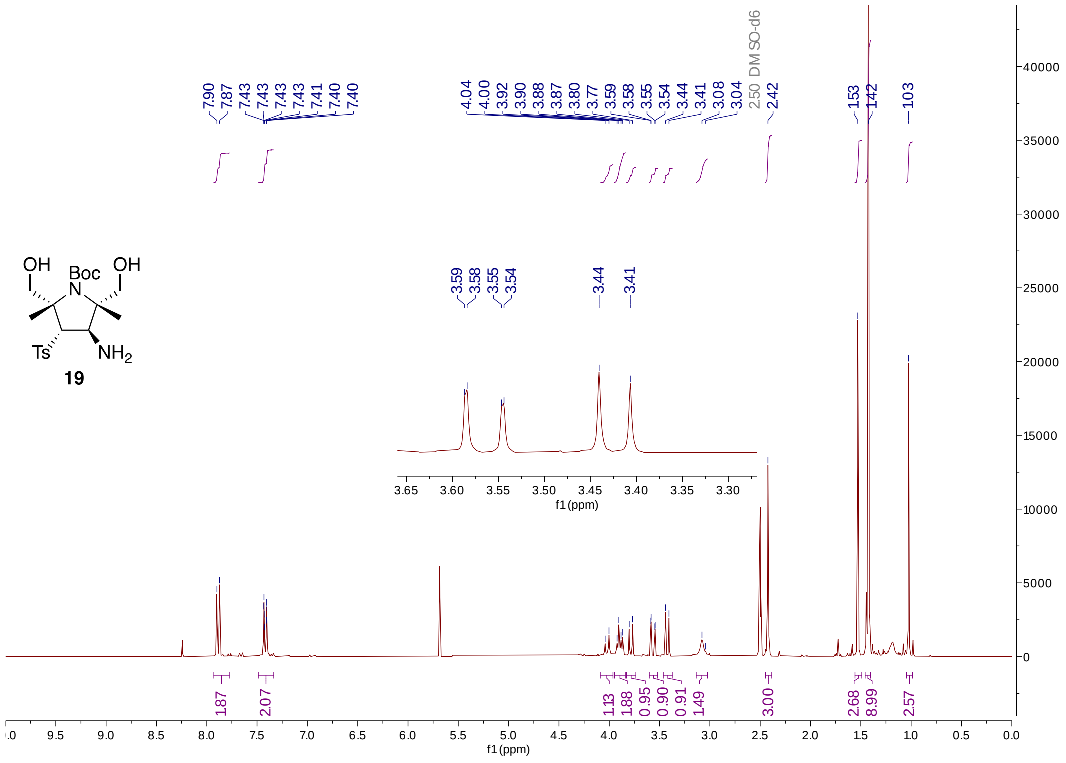

^{1}H-NMR, 300 MHz in DMSO-d_6 of **19**.

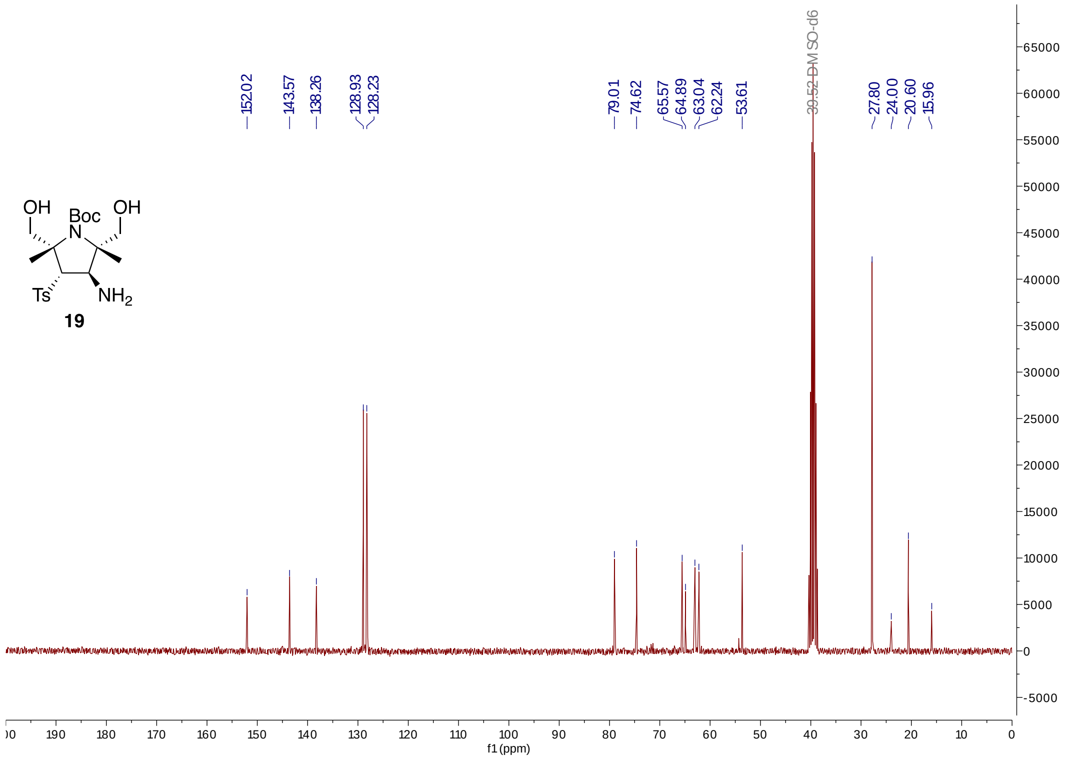

^{13}C-NMR, 76 MHz in DMSO-d_6 of **19**.

8. COSY and HSQC spectra for new compounds.

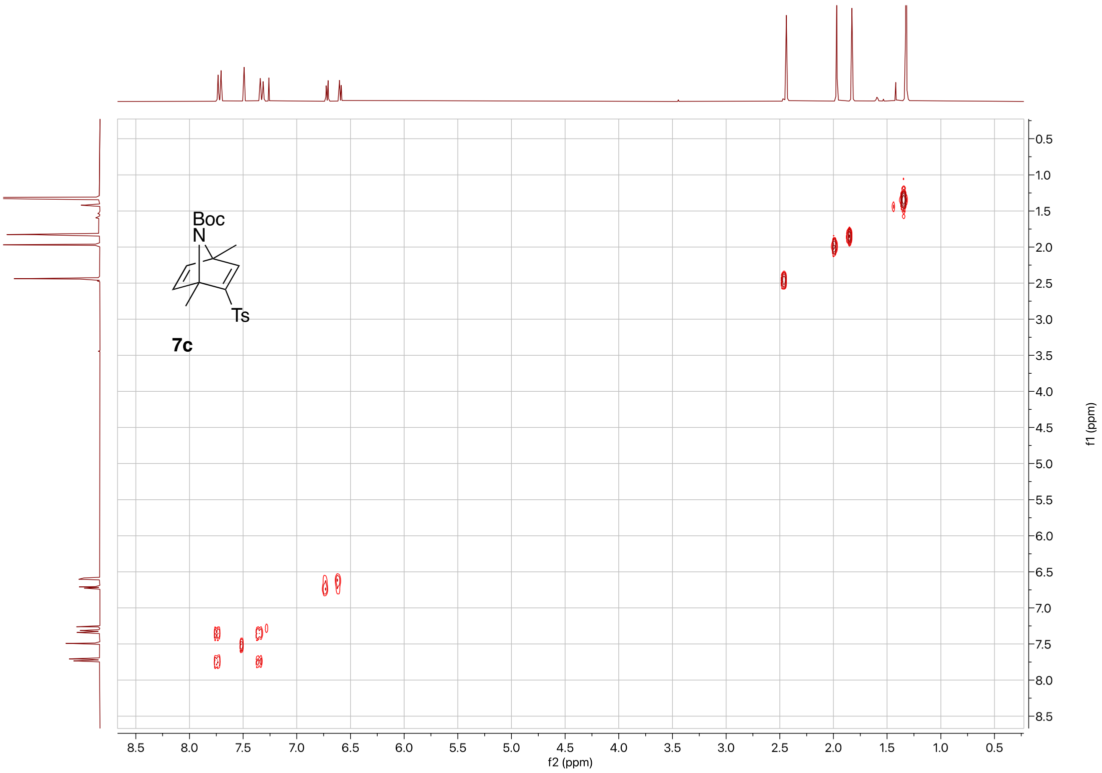

COSY, in $CDCl_3$ of **7c**.

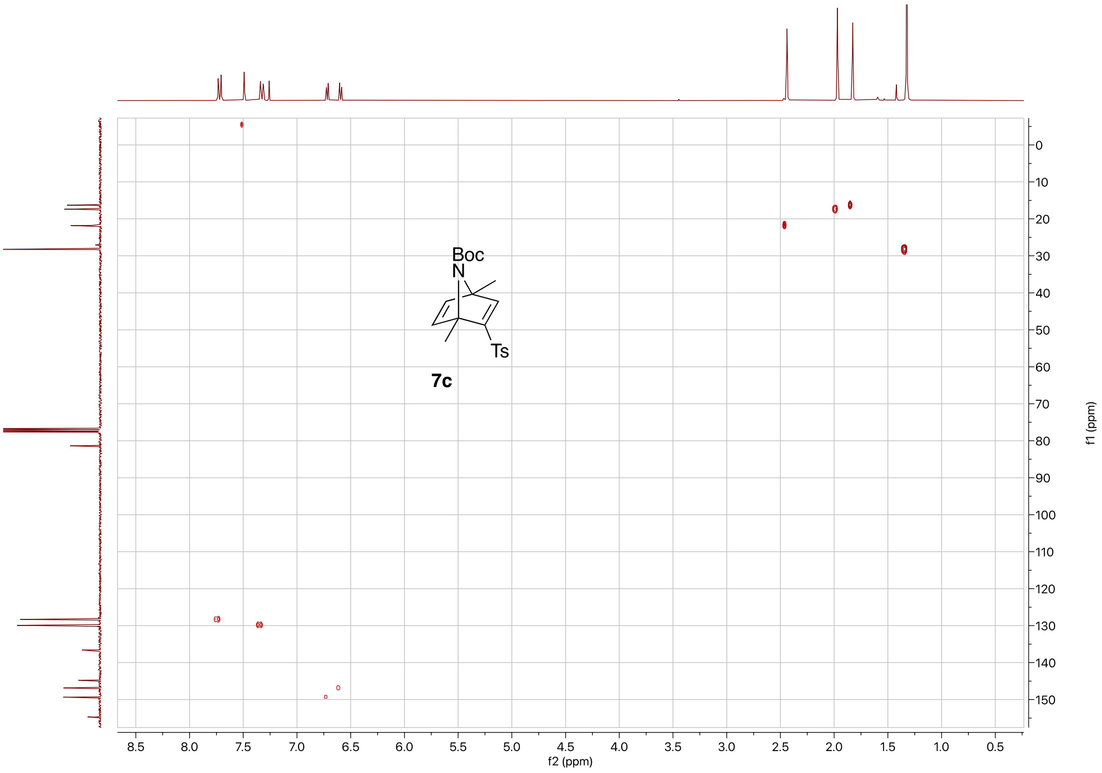

HSQC, in $CDCl_3$ of **7c**.

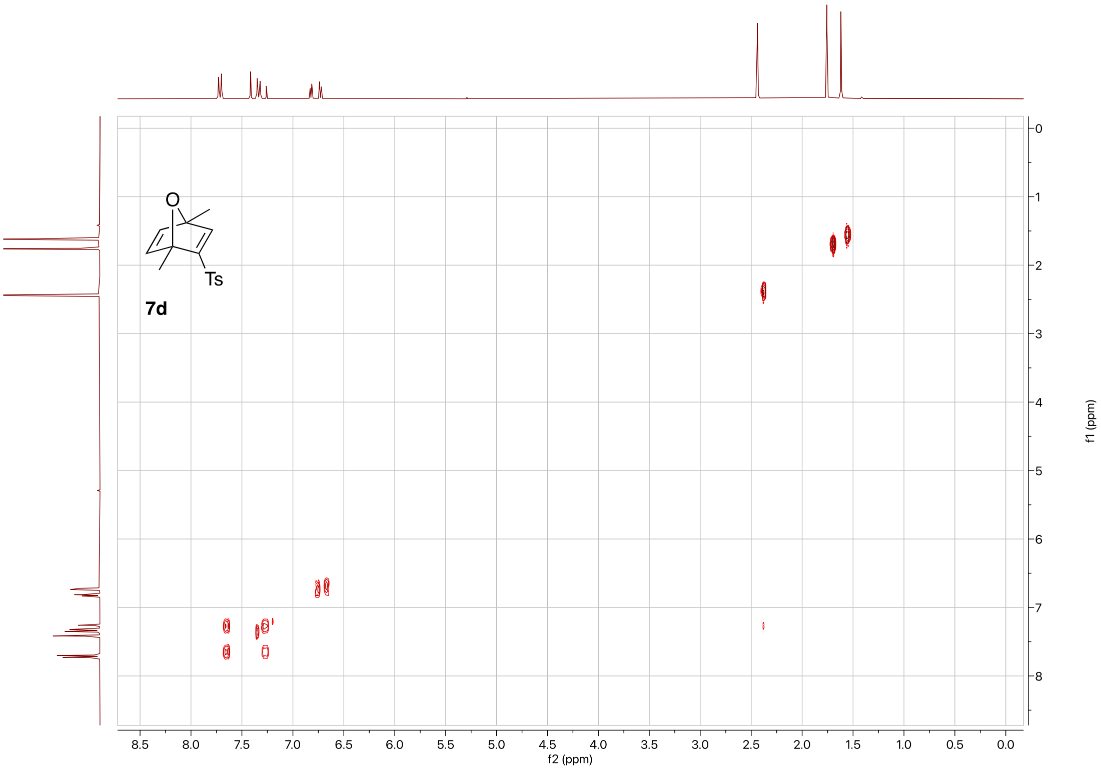

COSY, in $CDCl_3$ of **7d**.

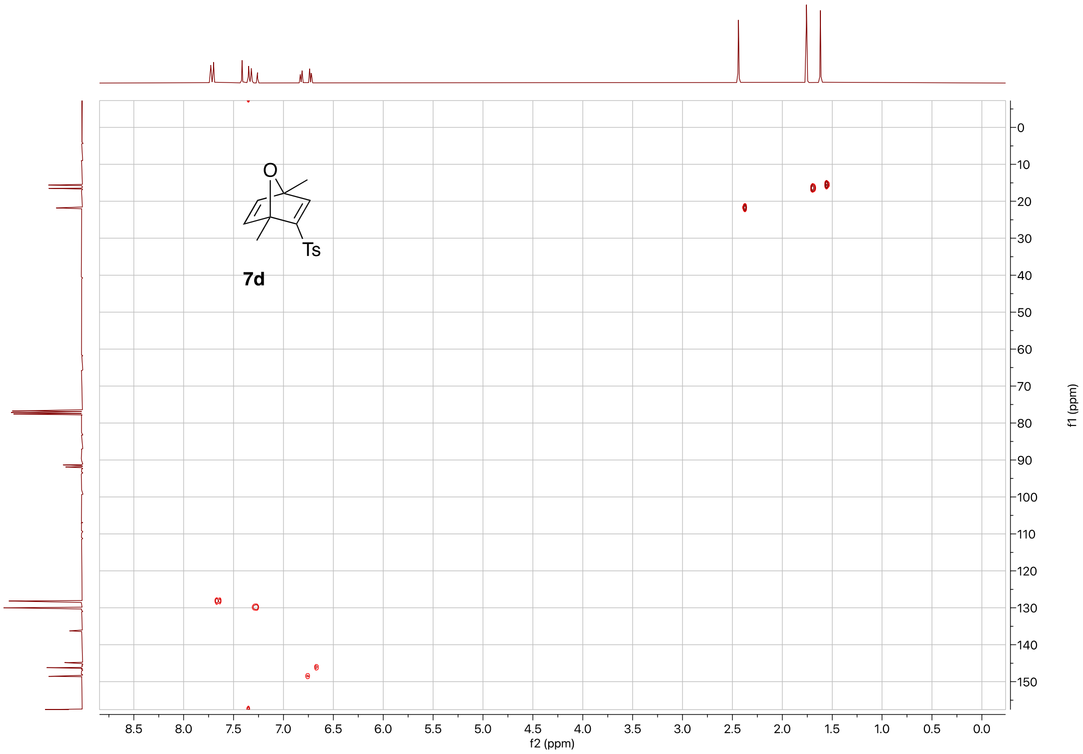

HSQC, in $CDCl_3$ of **7d**.

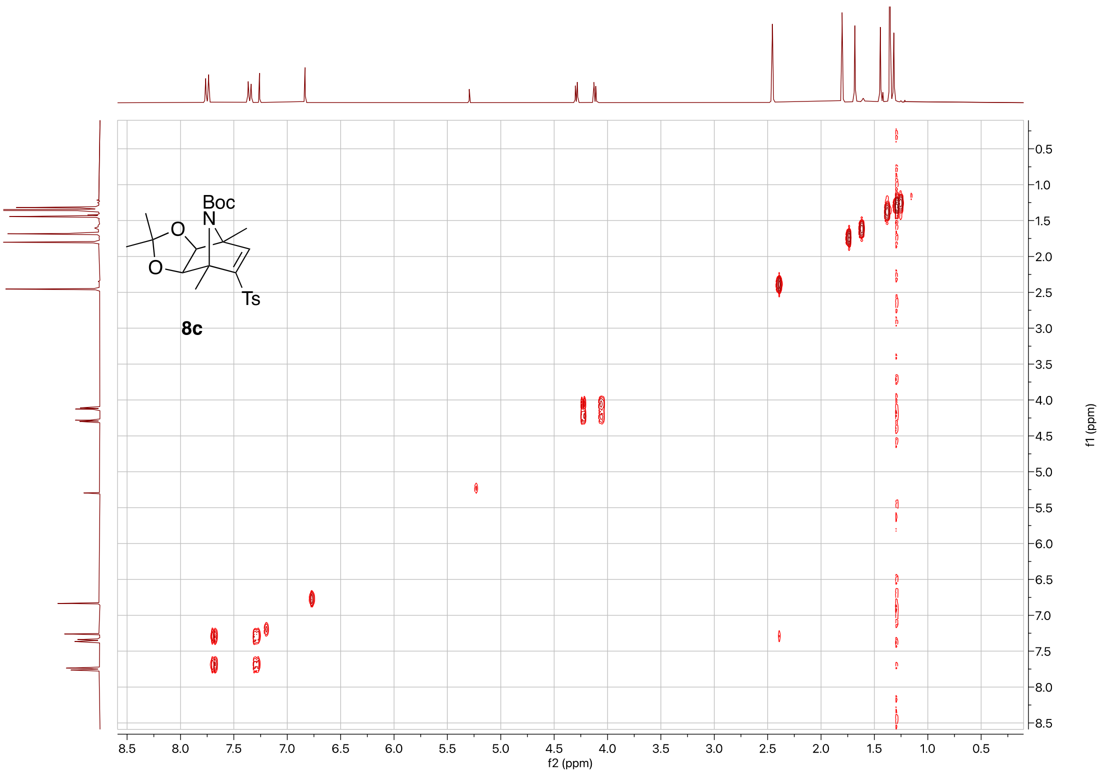

COSY, in $CDCl_3$ of **8c**.

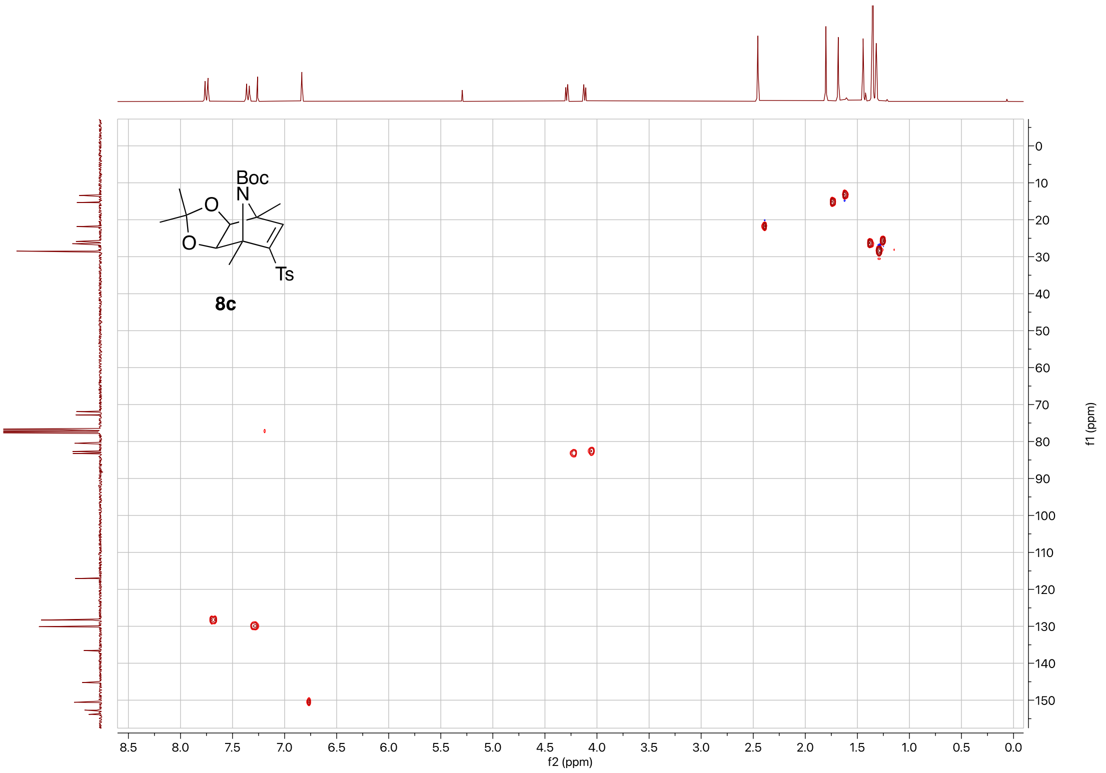

HSQC, in $CDCl_3$ of **8c**.

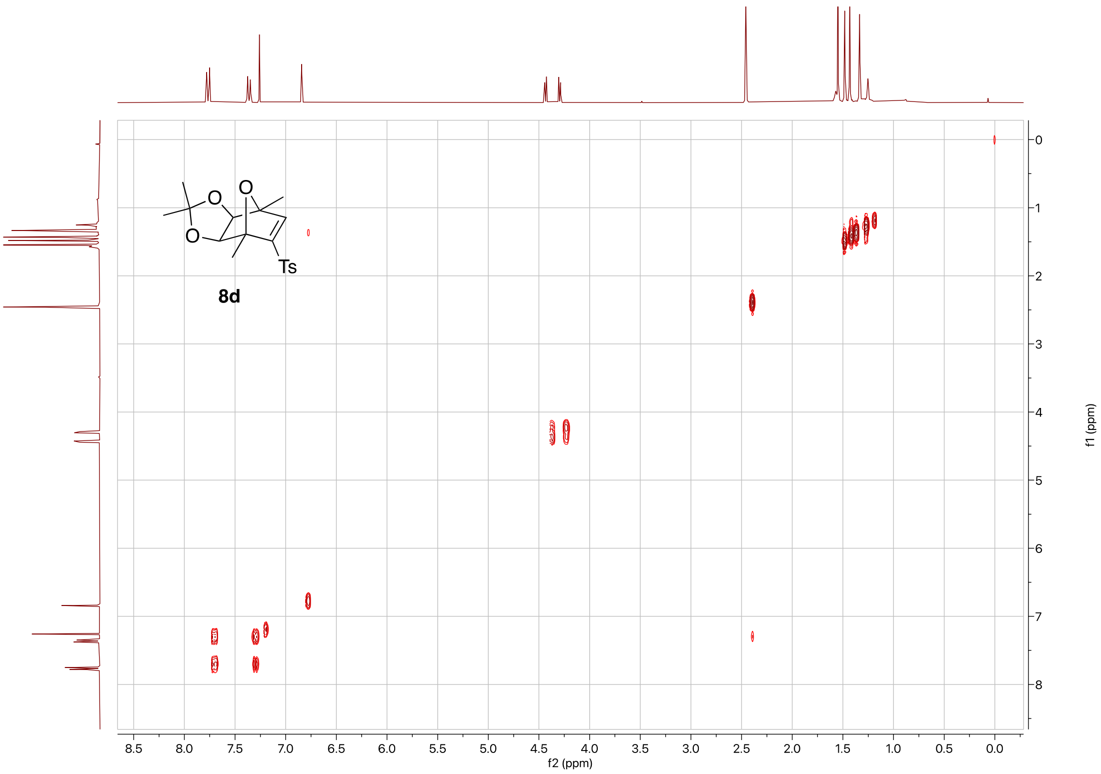

COSY, in $CDCl_3$ of **8d**.

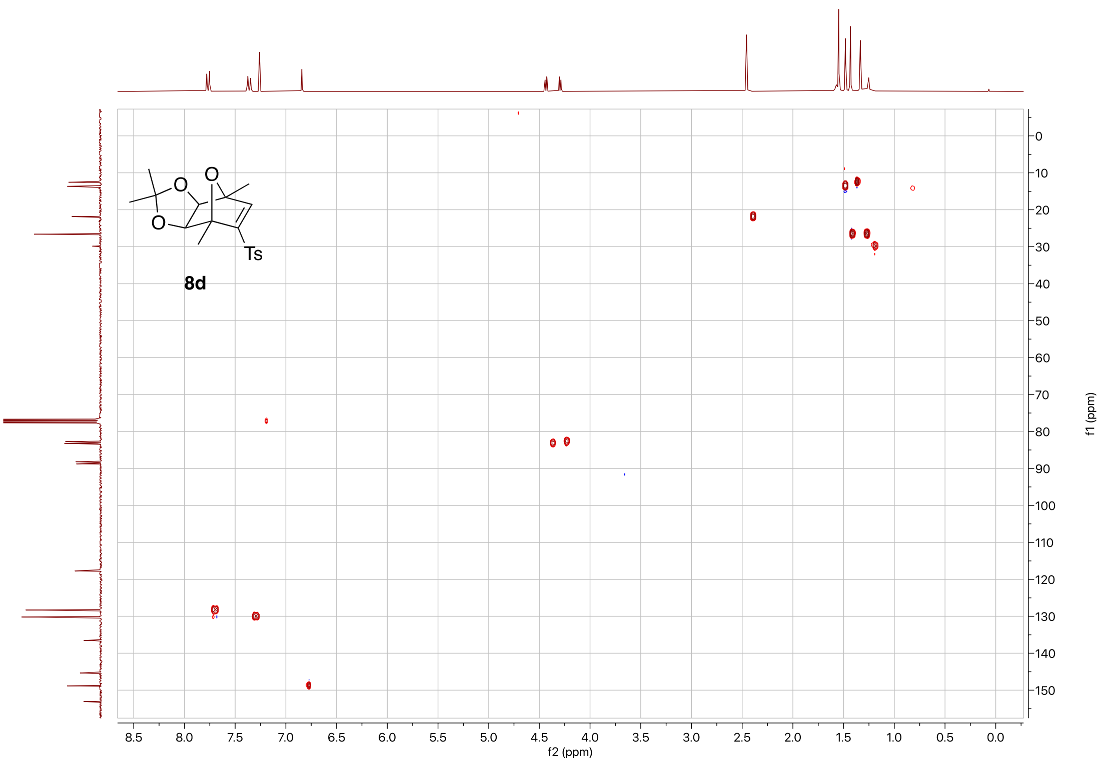

HSQC, in $CDCl_3$ of **8d**.

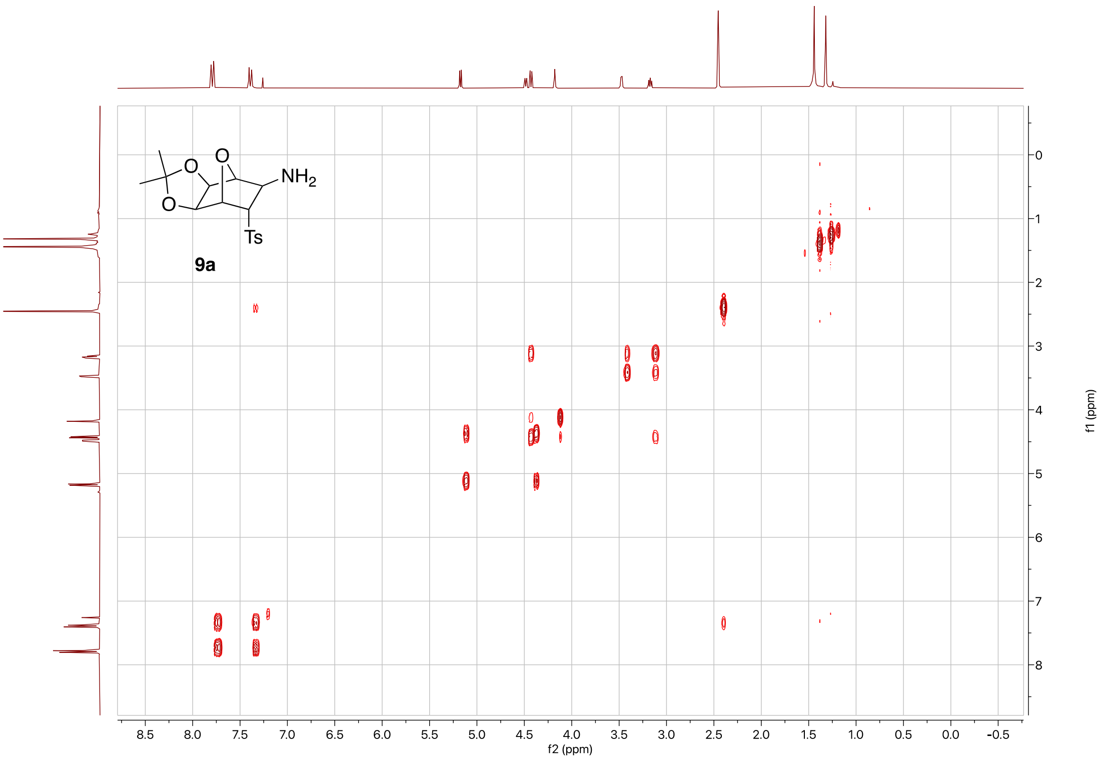

COSY, in $CDCl_3$ of **9a**.

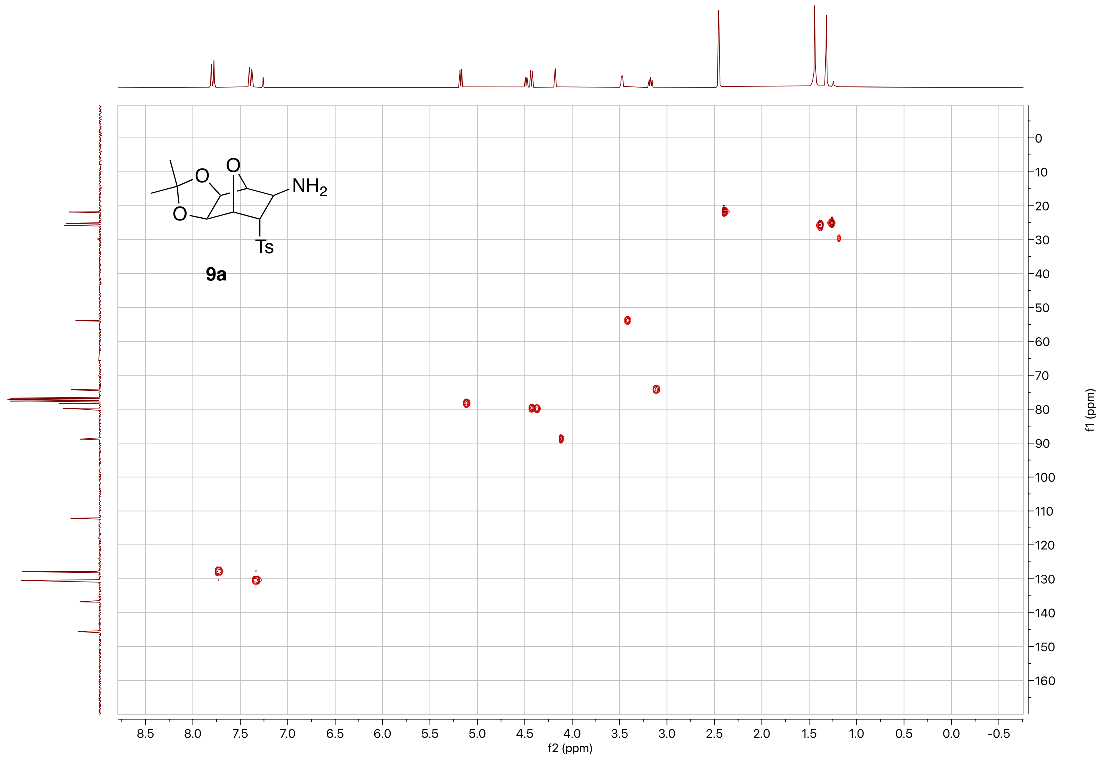

HSQC, in $CDCl_3$ of **9a**.

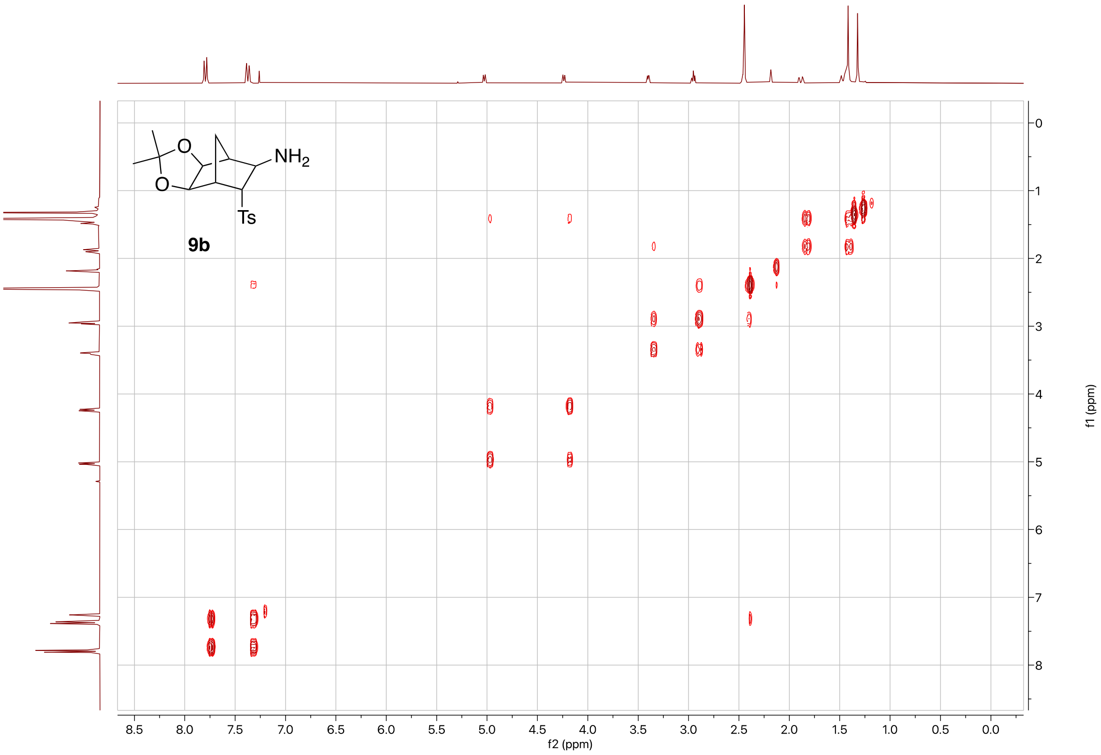

COSY, in $CDCl_3$ of **9b**.

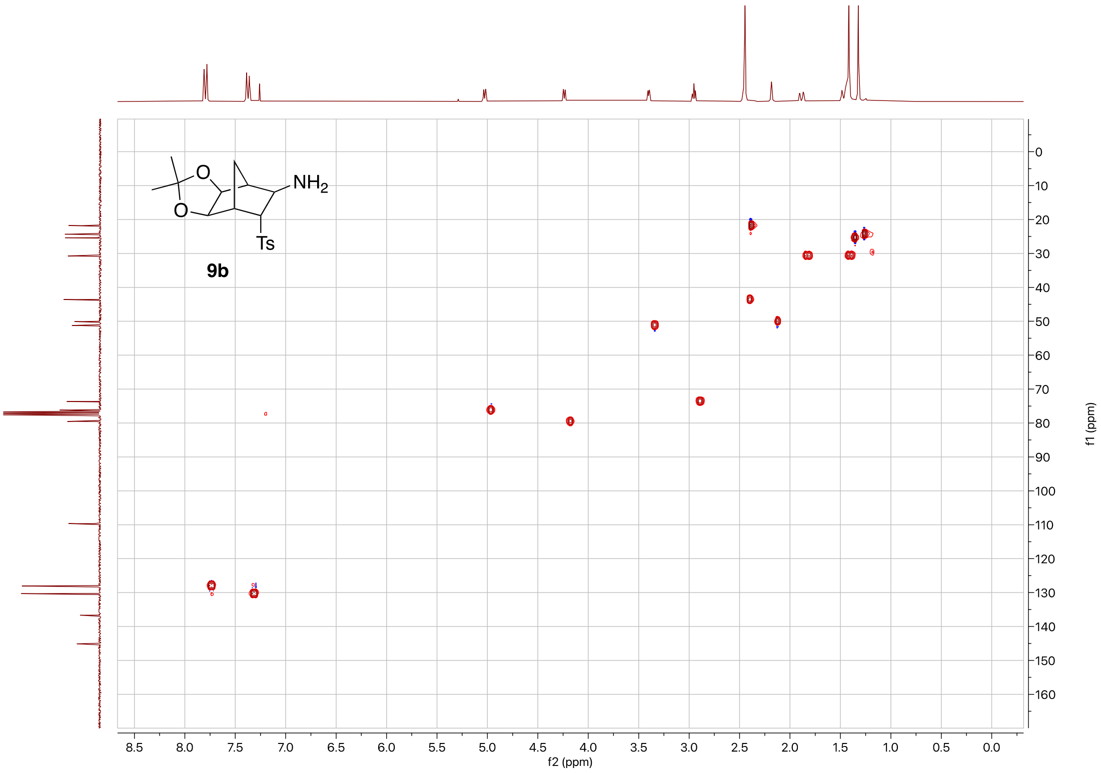

HSQC , in $CDCl_3$ of **9b**.

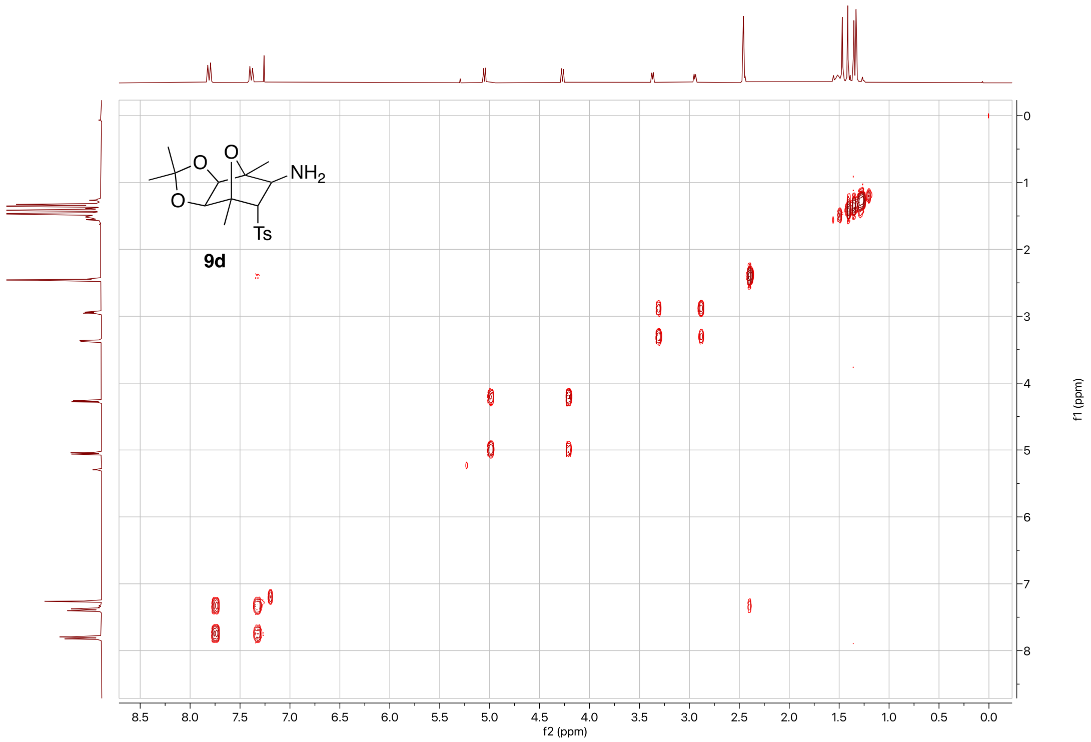

COSY, in $CDCl_3$ of **9d**.

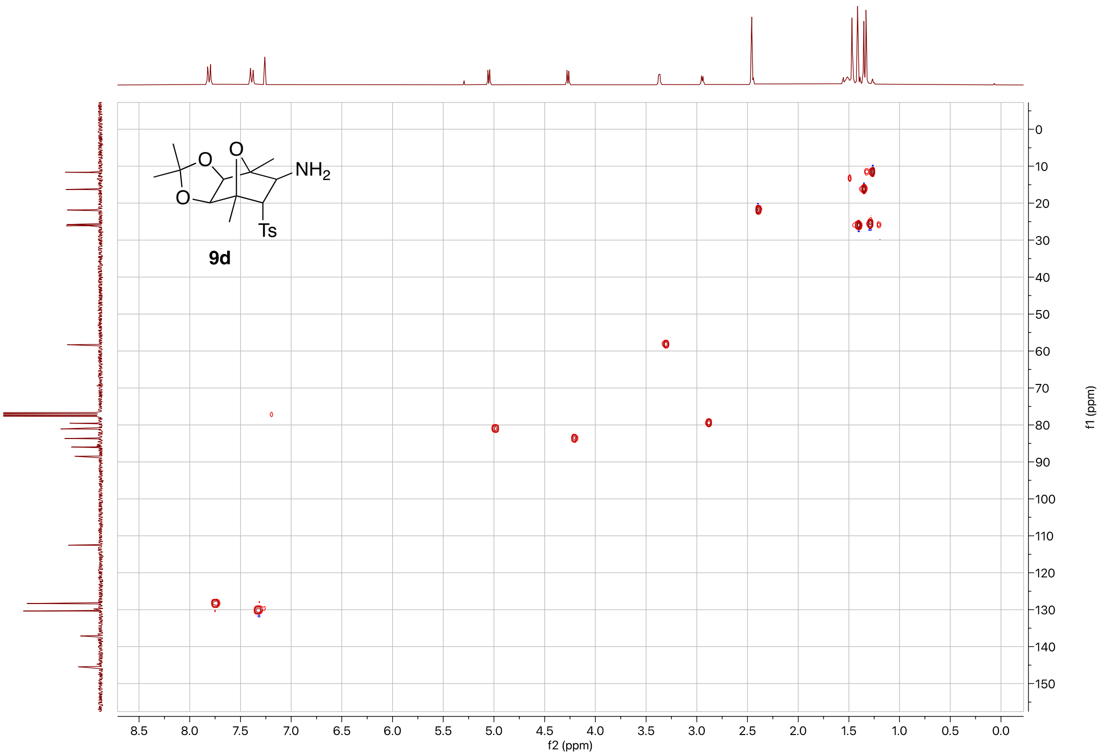

HSQC , in $CDCl_3$ of **9d**.

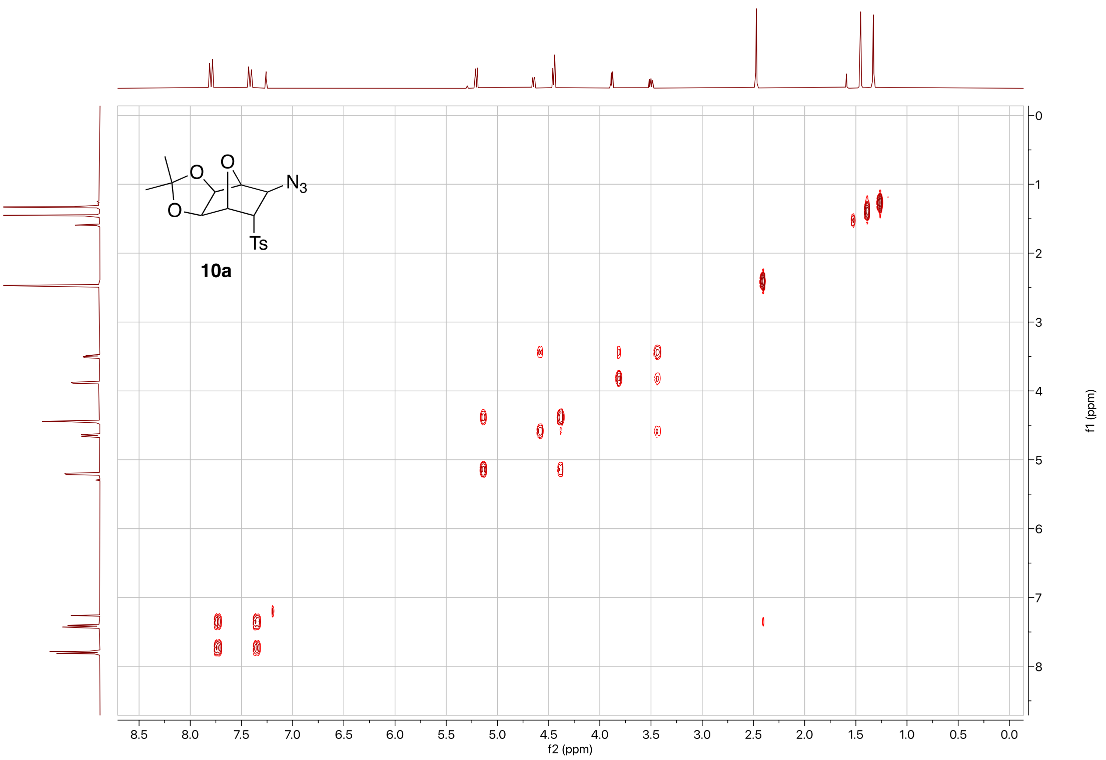

COSY, in $CDCl_3$ of **10a**.

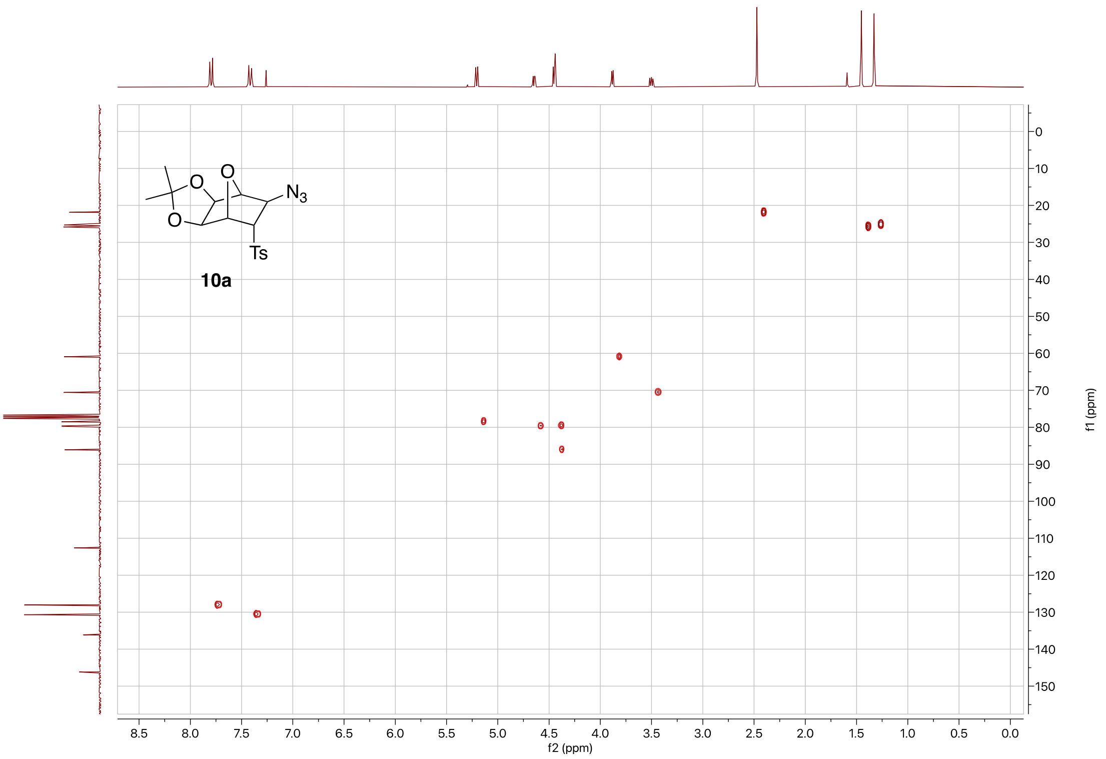

HSQC, in $CDCl_3$ of **10a**.

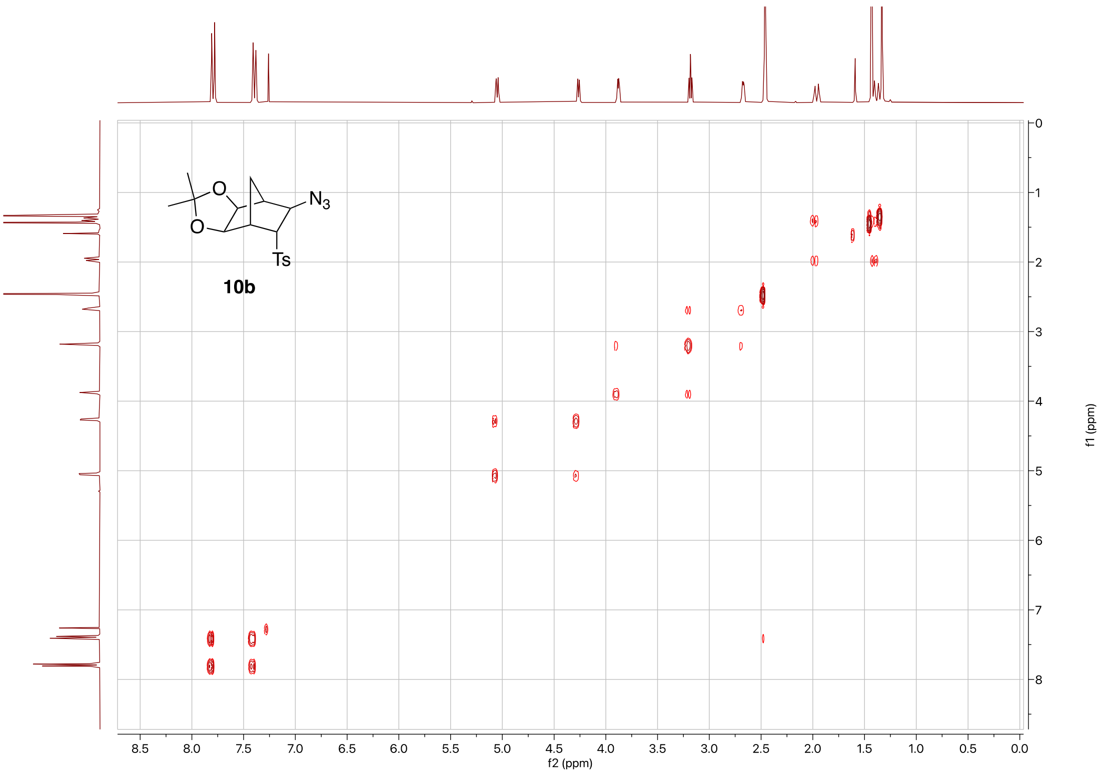

COSY, in $CDCl_3$ of **10b**.

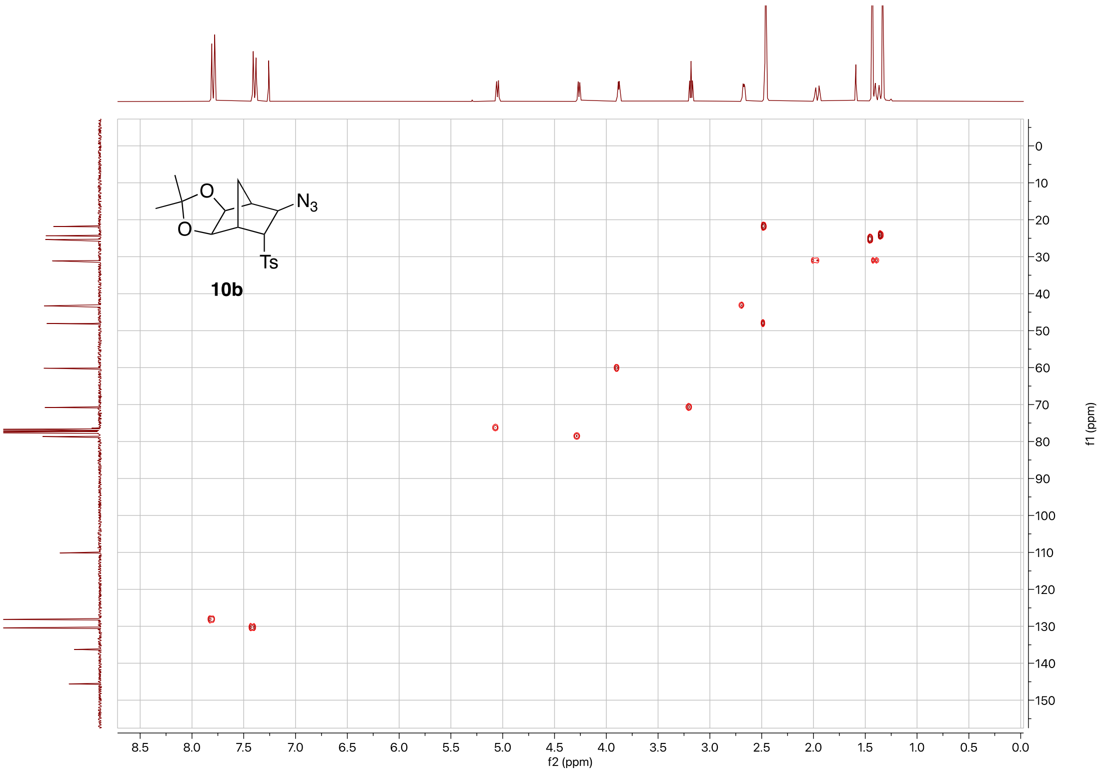

HSQC, in $CDCl_3$ of **10b**.

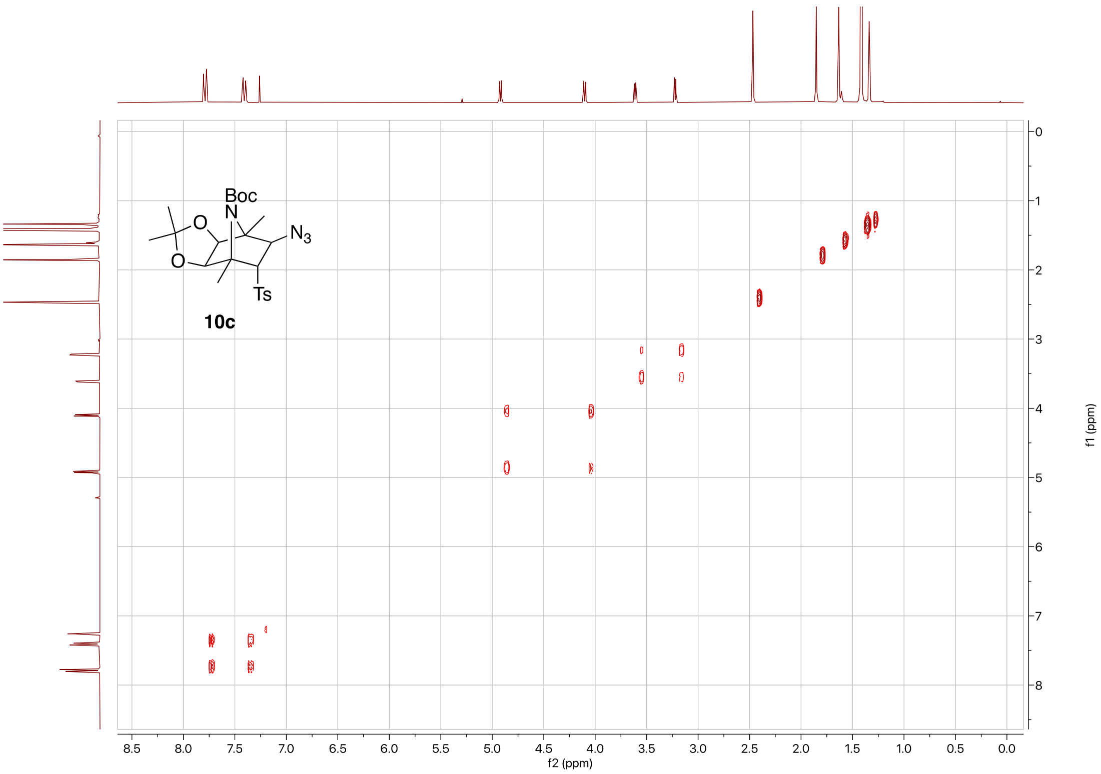

COSY, in $CDCl_3$ of **10c**.

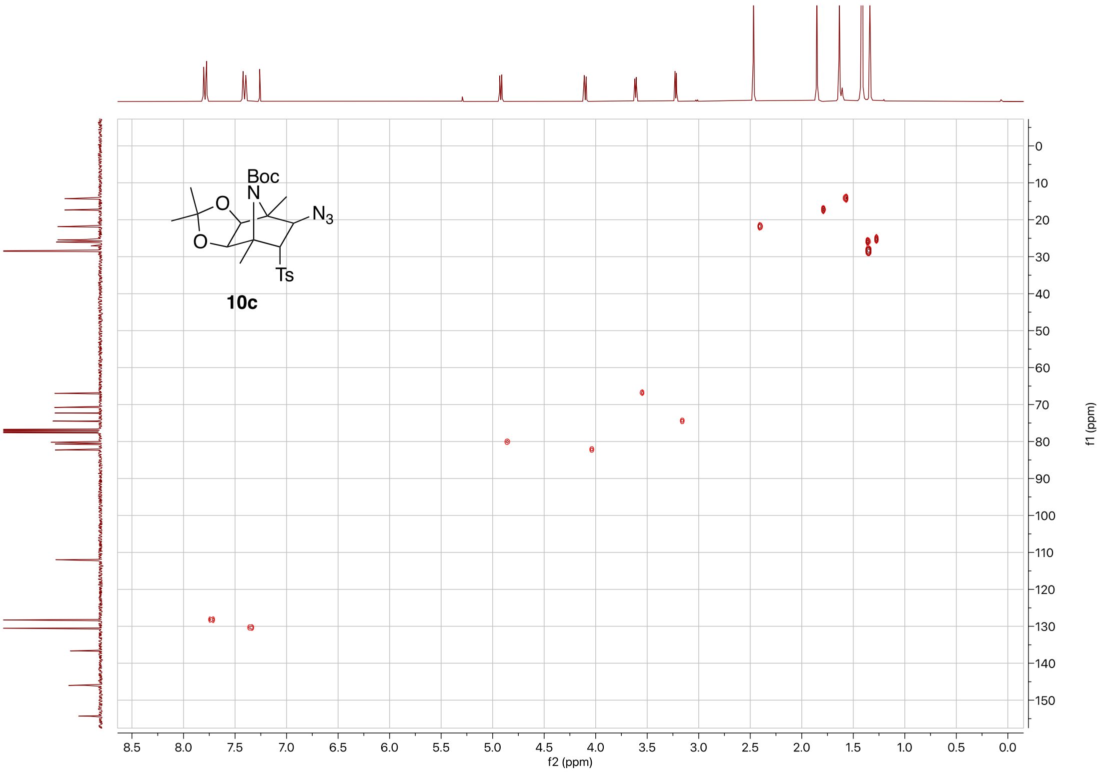

HSQC, in $CDCl_3$ of **10c**.

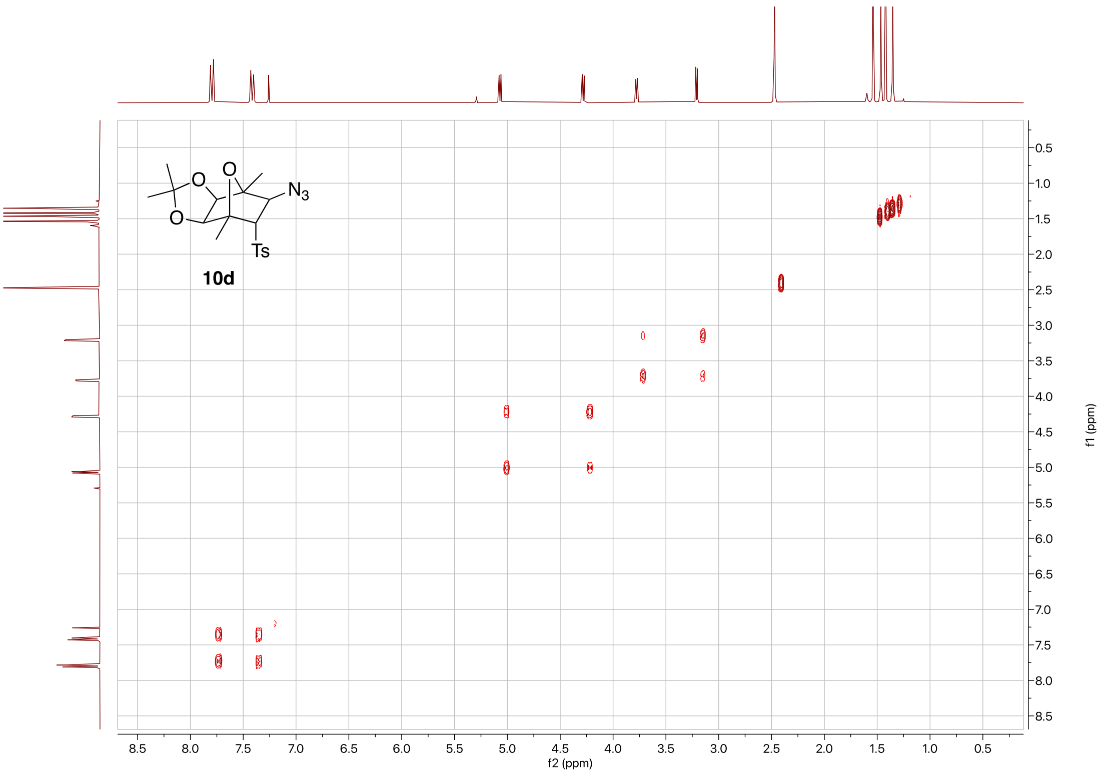

COSY, in $CDCl_3$ of **10d**.

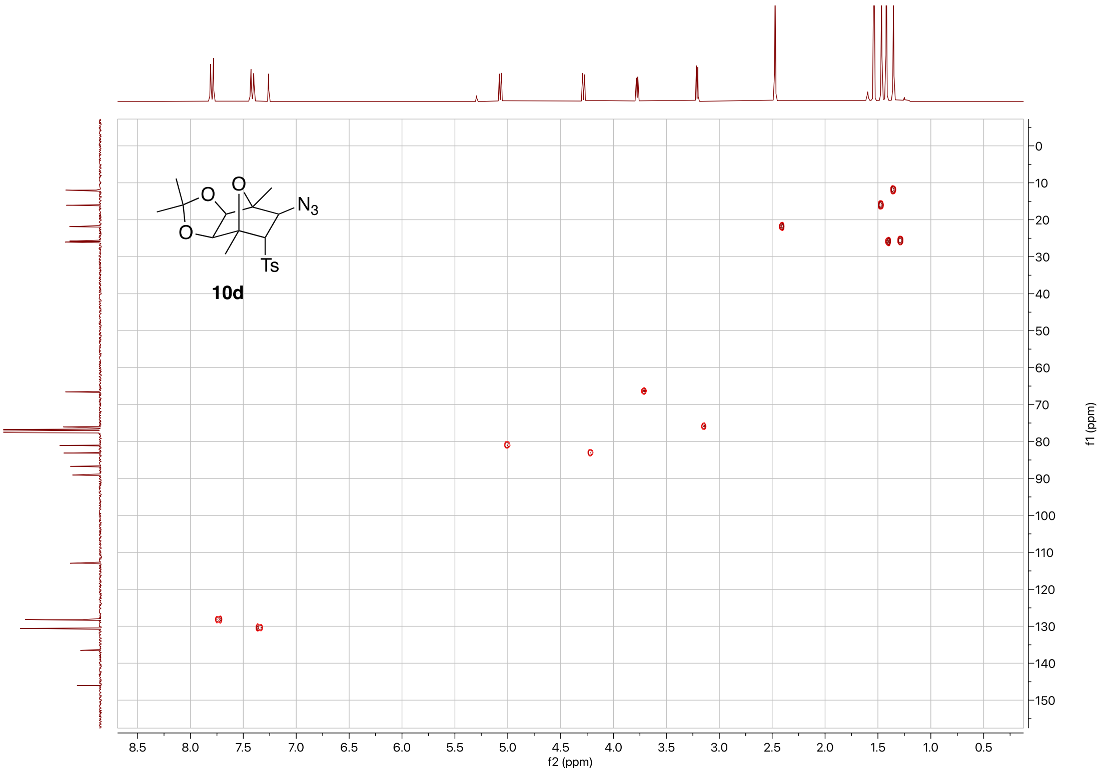

HSQC, in $CDCl_3$ of **10d**.

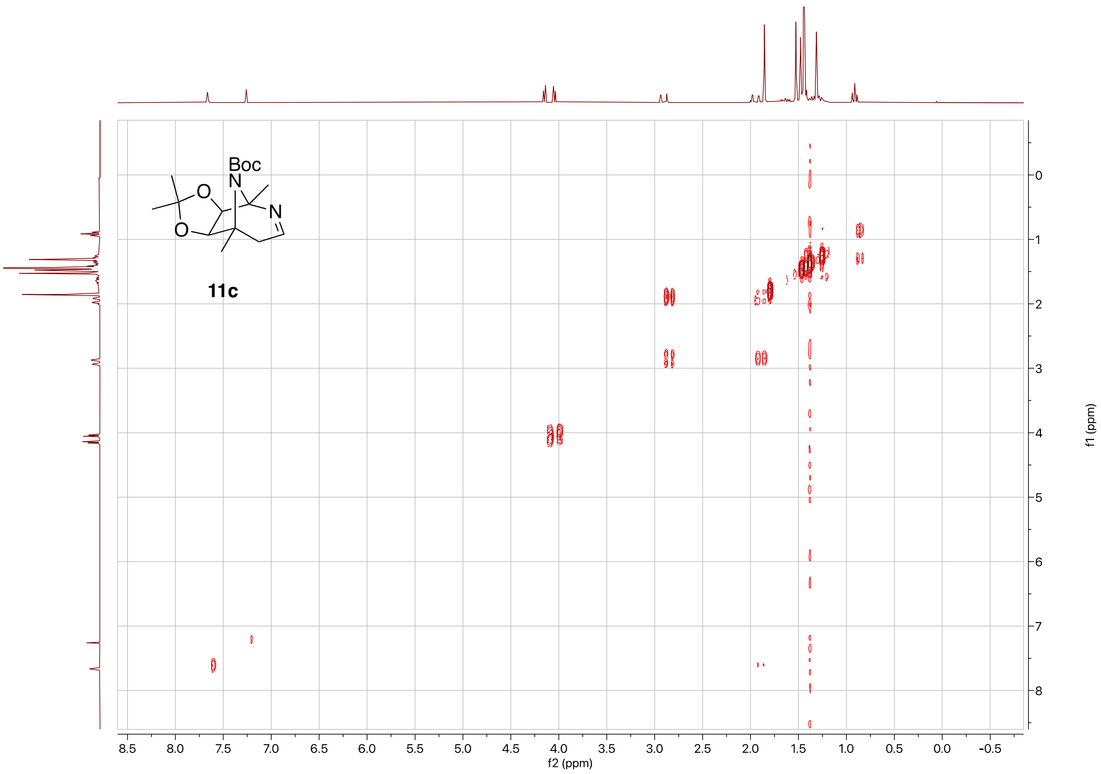

COSY, in $CDCl_3$ of **11c**.

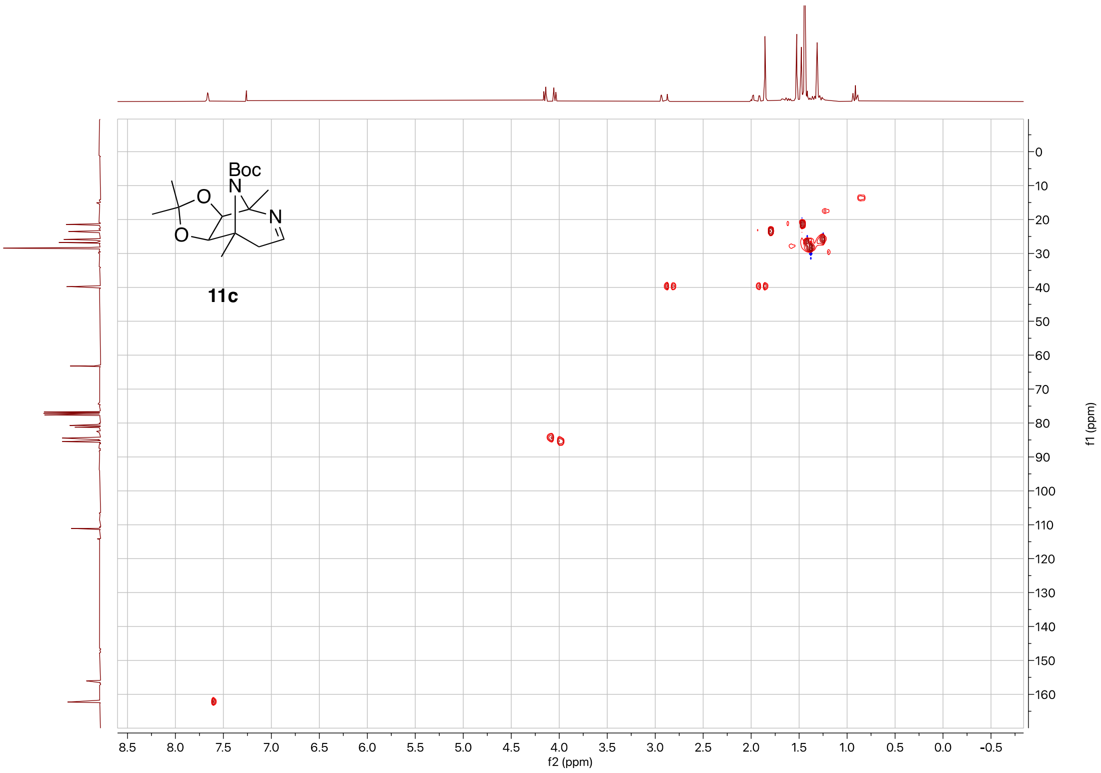

HSQC, in $CDCl_3$ of **11c**.

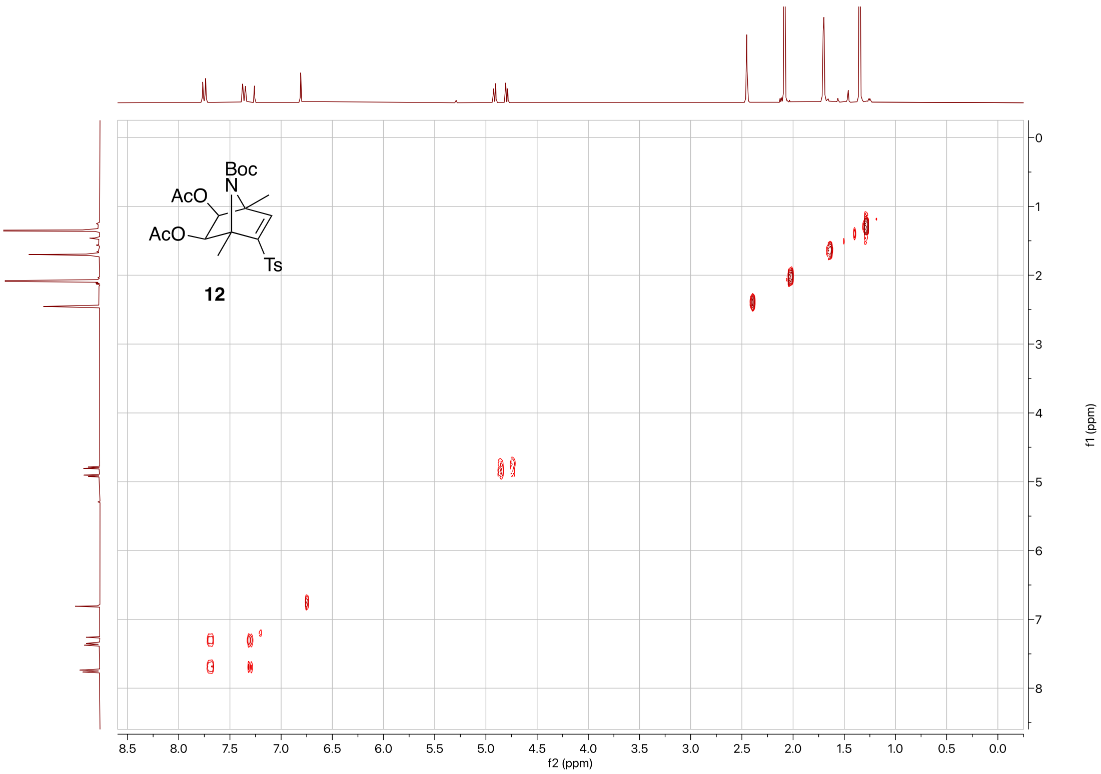

COSY, in $CDCl_3$ of **12**.

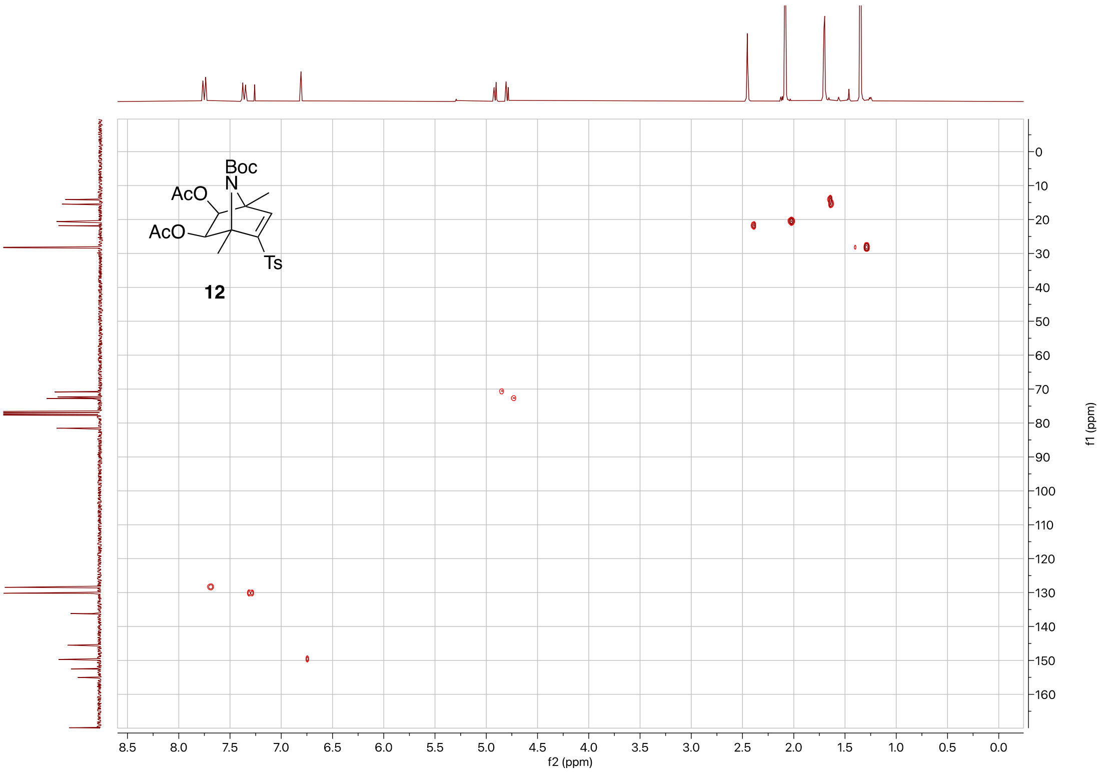

HSQC, in $CDCl_3$ of **12**.

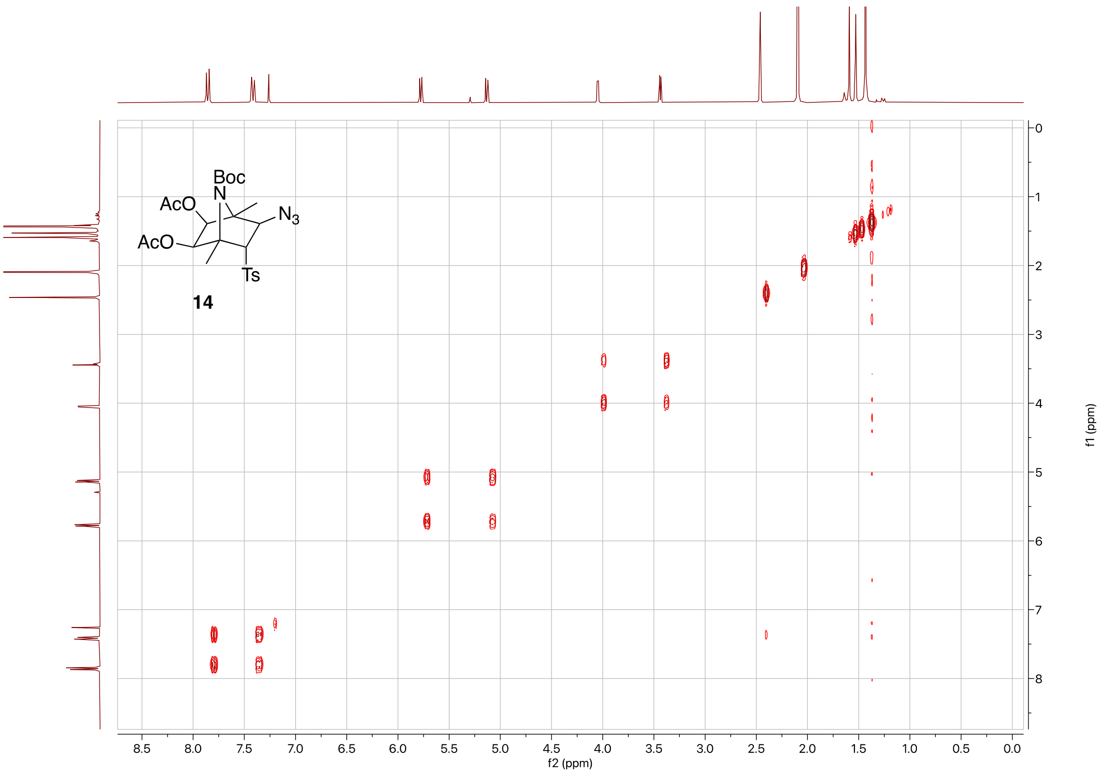

COSY, in $CDCl_3$ of **14**.

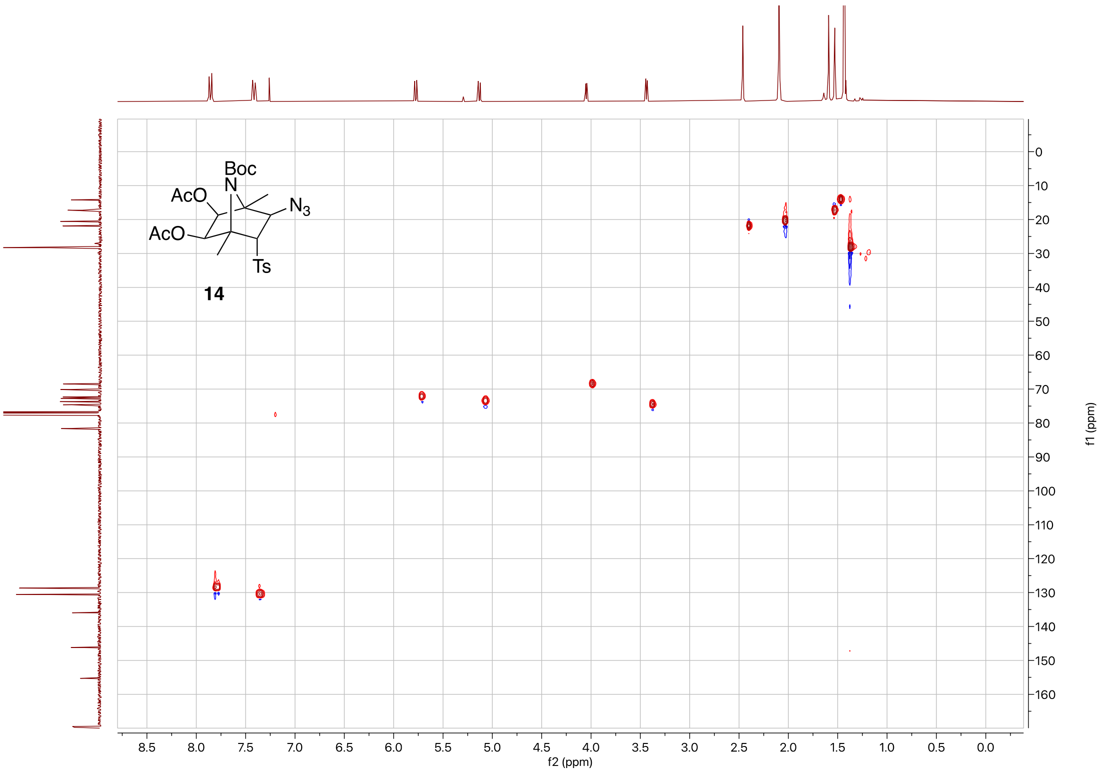

HSQC, in $CDCl_3$ of **14**.

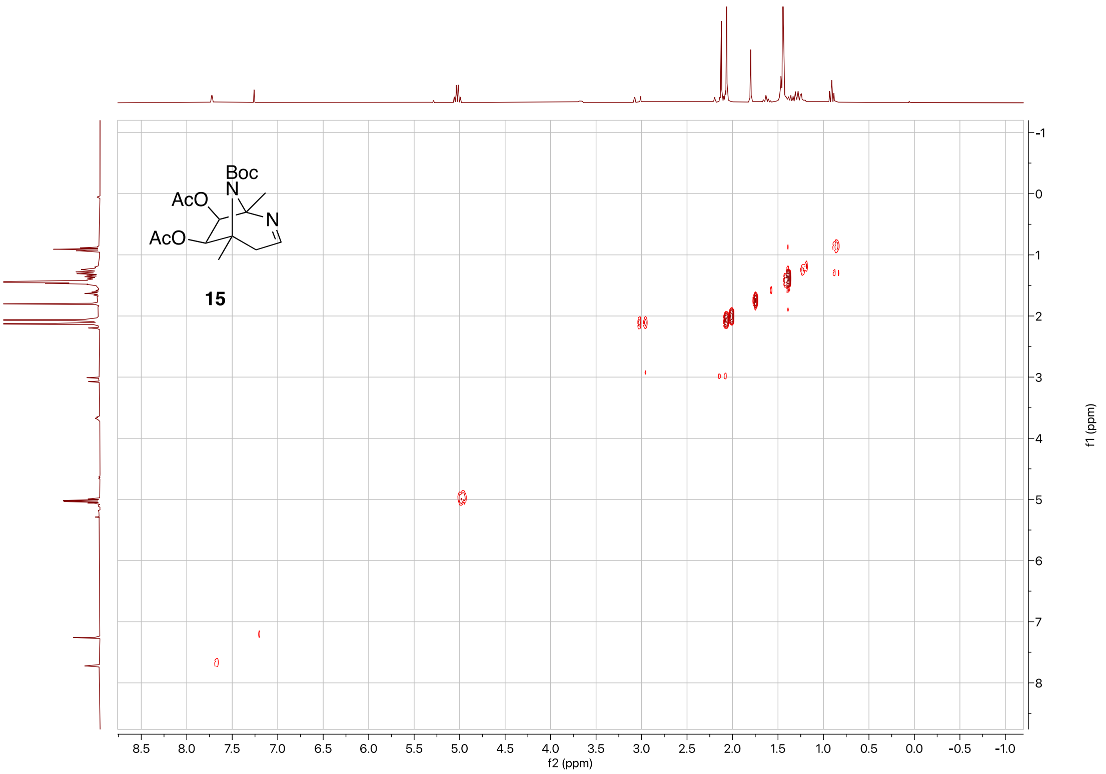

COSY, in $CDCl_3$ of **15**.

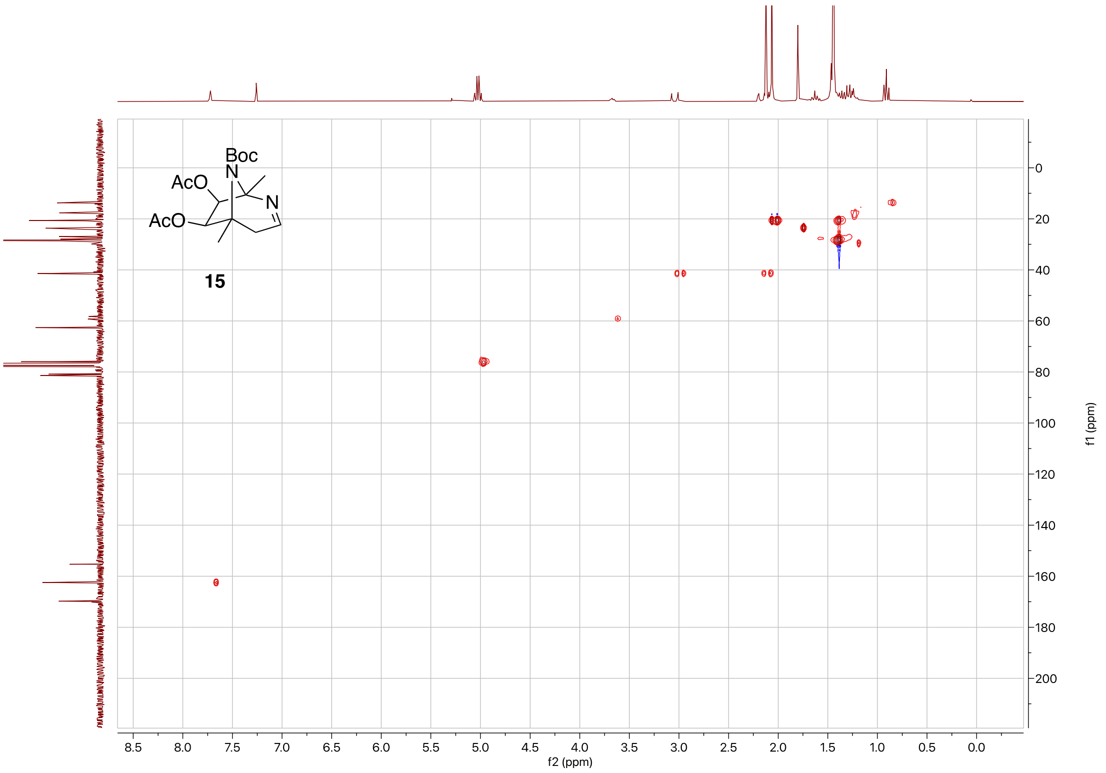

HSQC, in $CDCl_3$ of **15**.

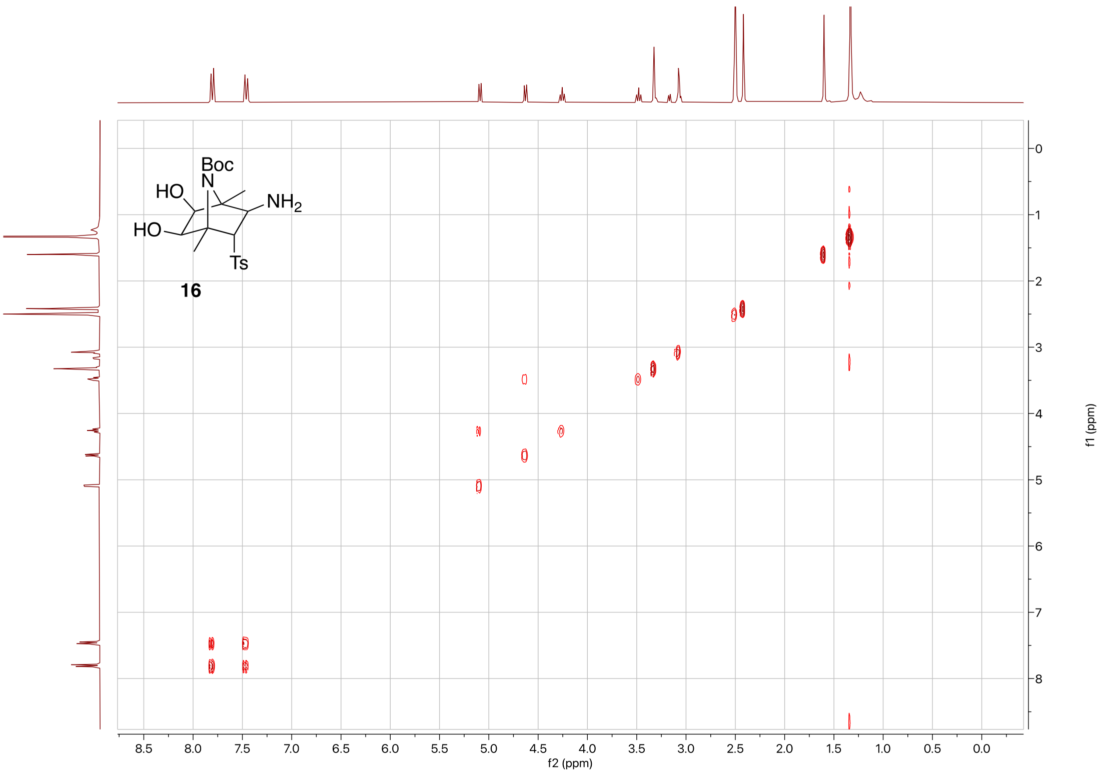

COSY, in DMSO-d_6 of **16**.

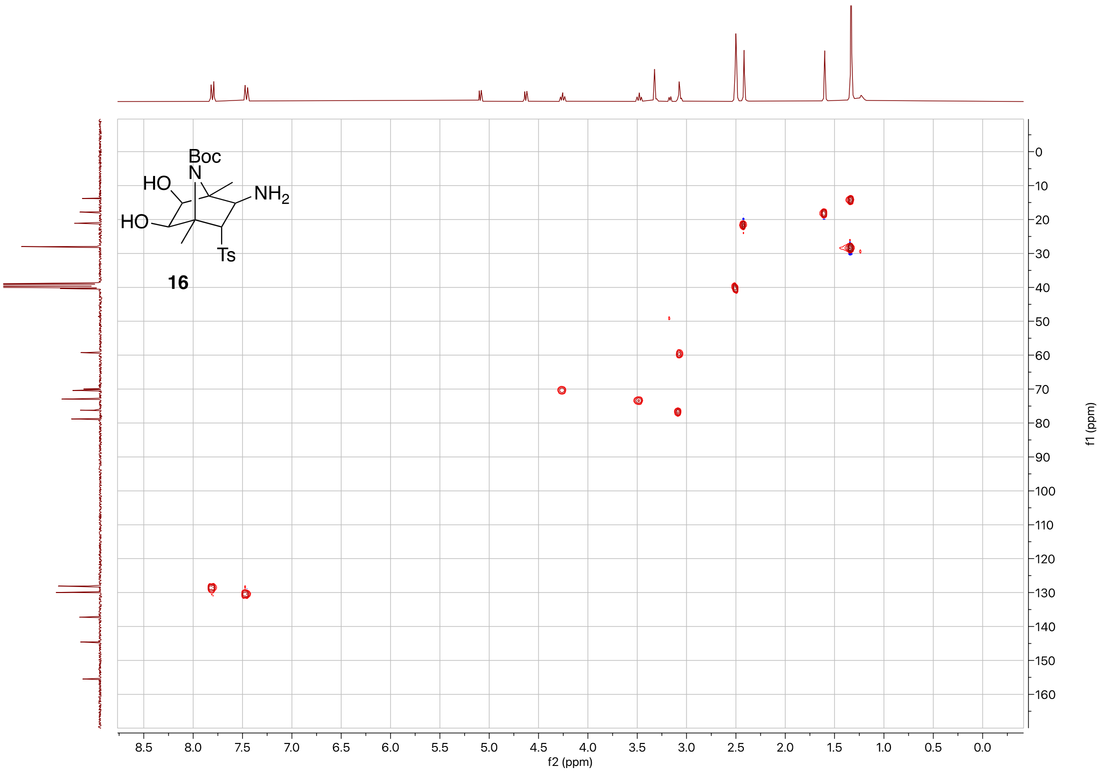

HSQC, in DMSO-d_6 of **16**.

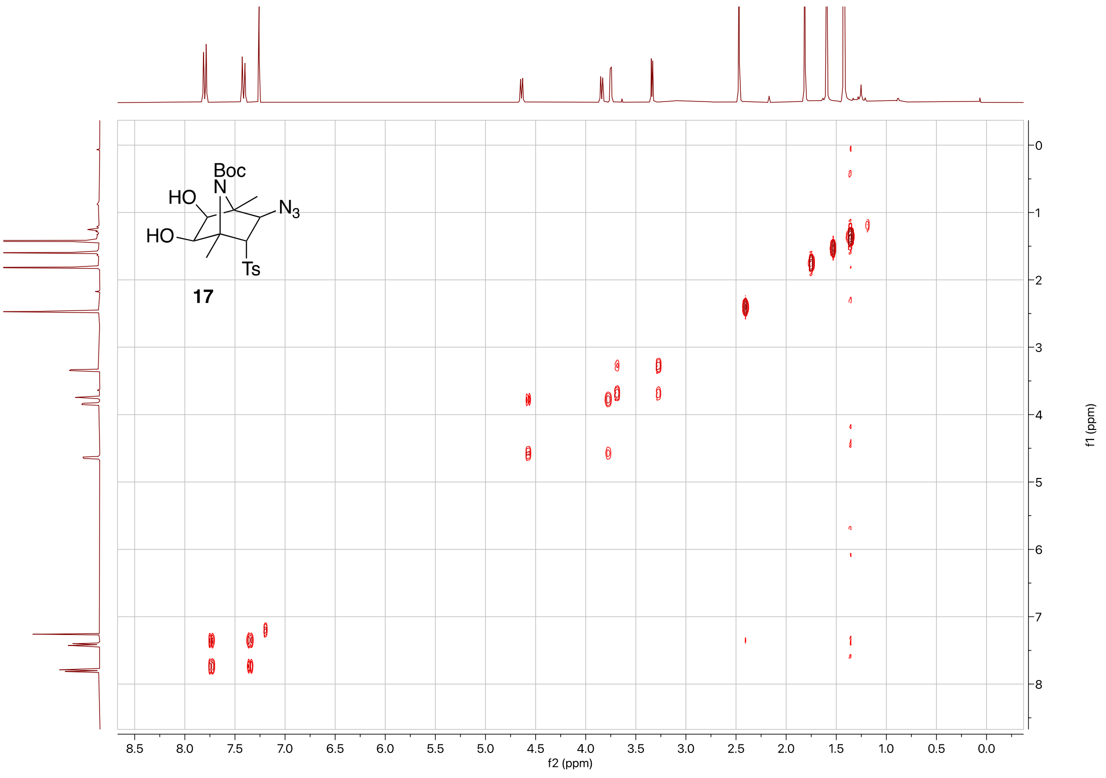

COSY, in $CDCl_3$ of **17**.

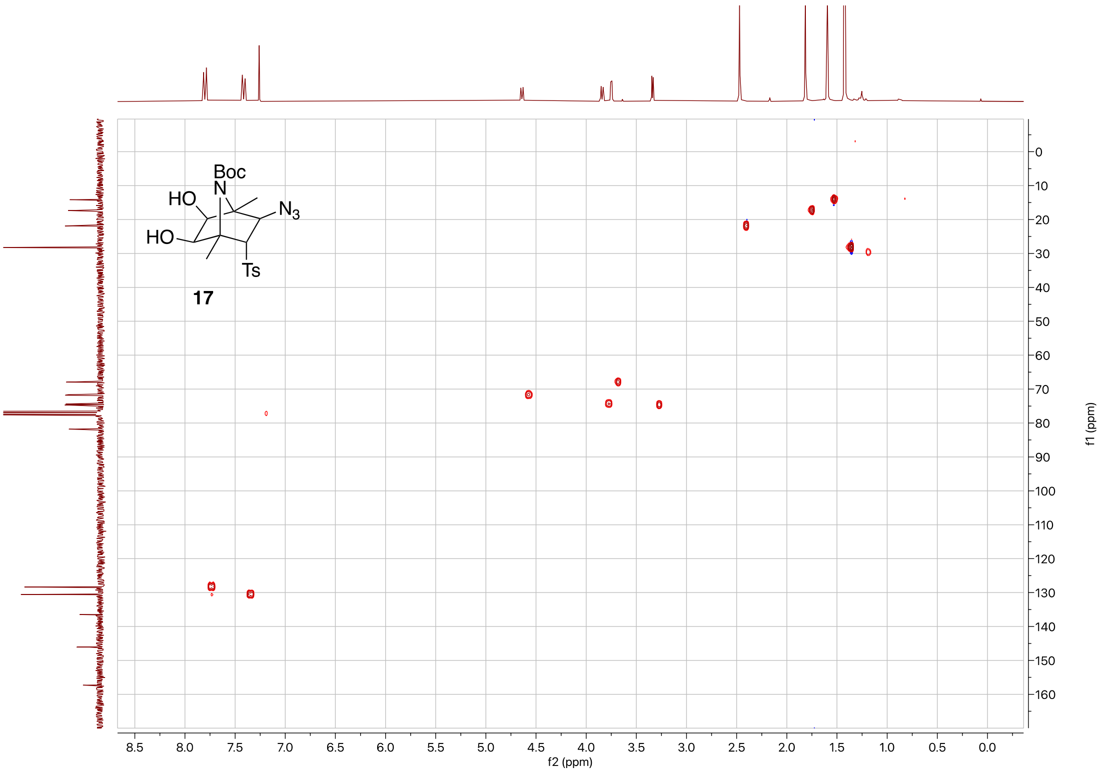

HSQC, in $CDCl_3$ of **17**.

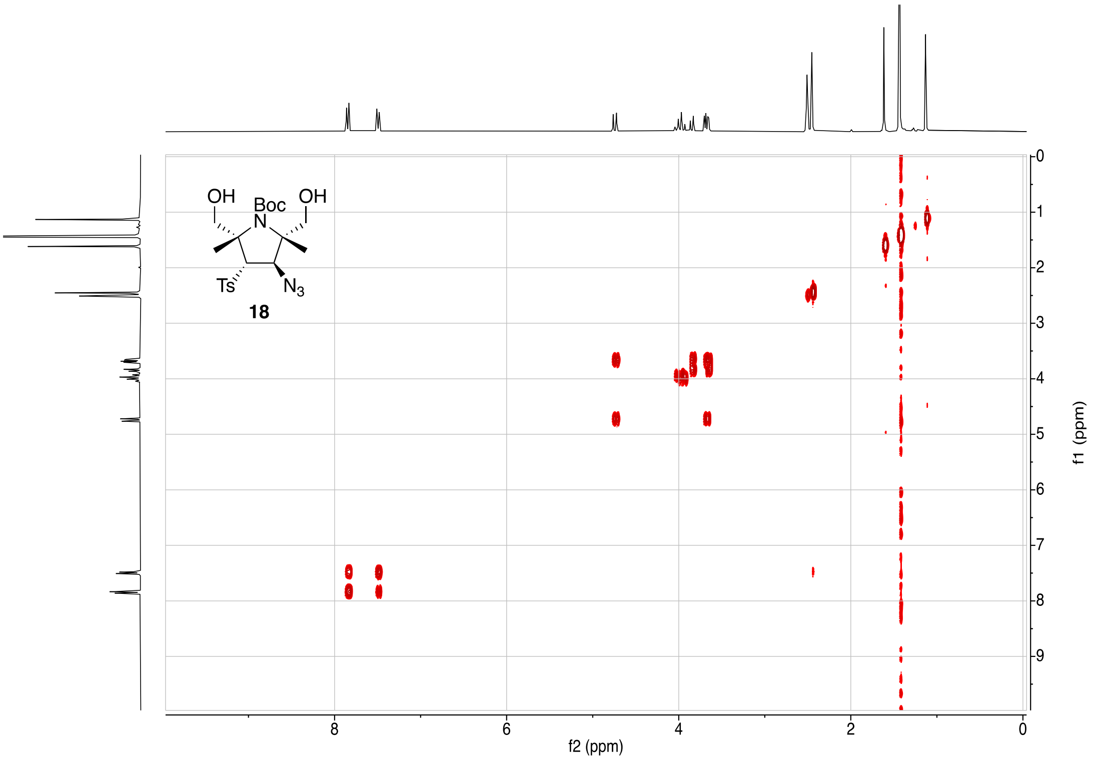

COSY, in DMSO-d_6 of **18**.

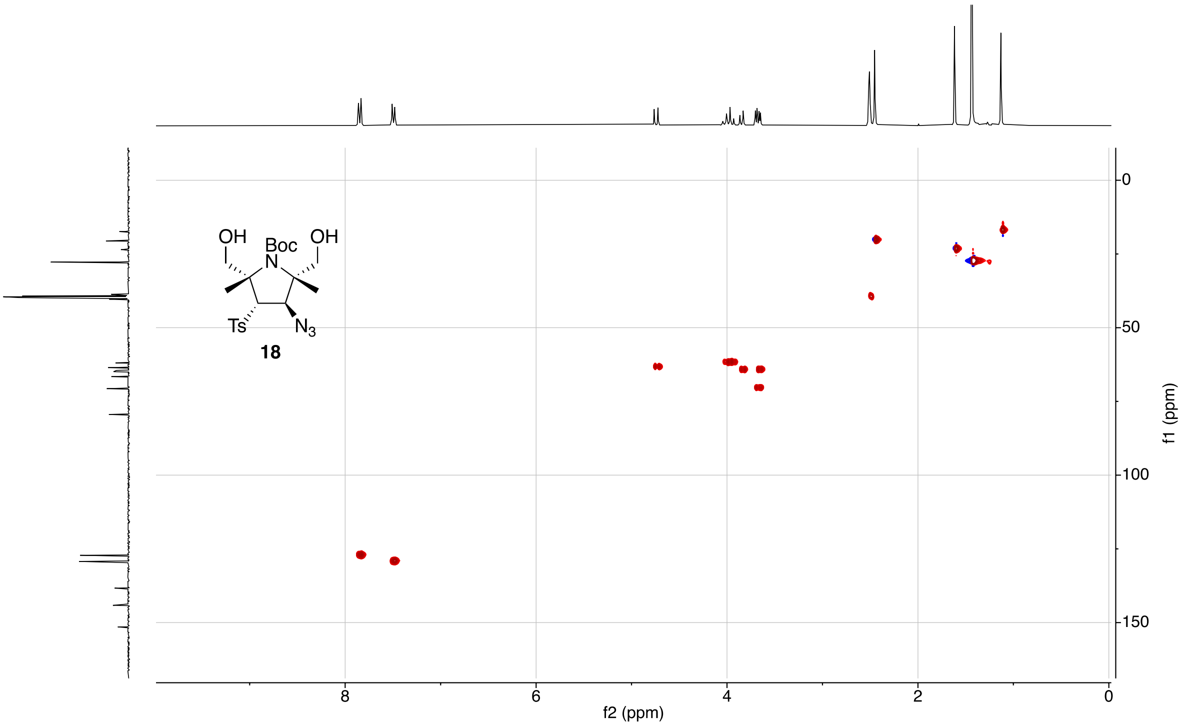

HSQC, in DMSO-d_6 of **18**.

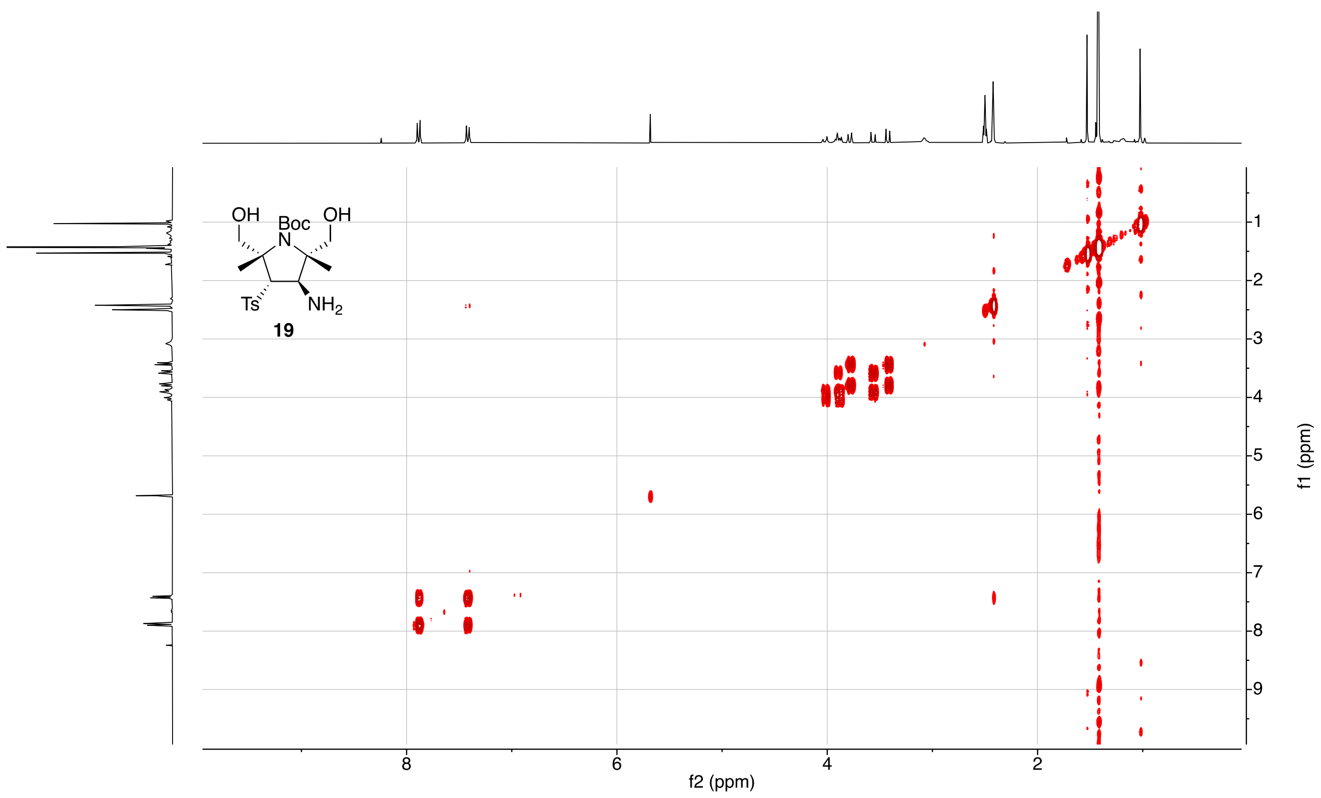

COSY, in DMSO-d_6 of **19**.

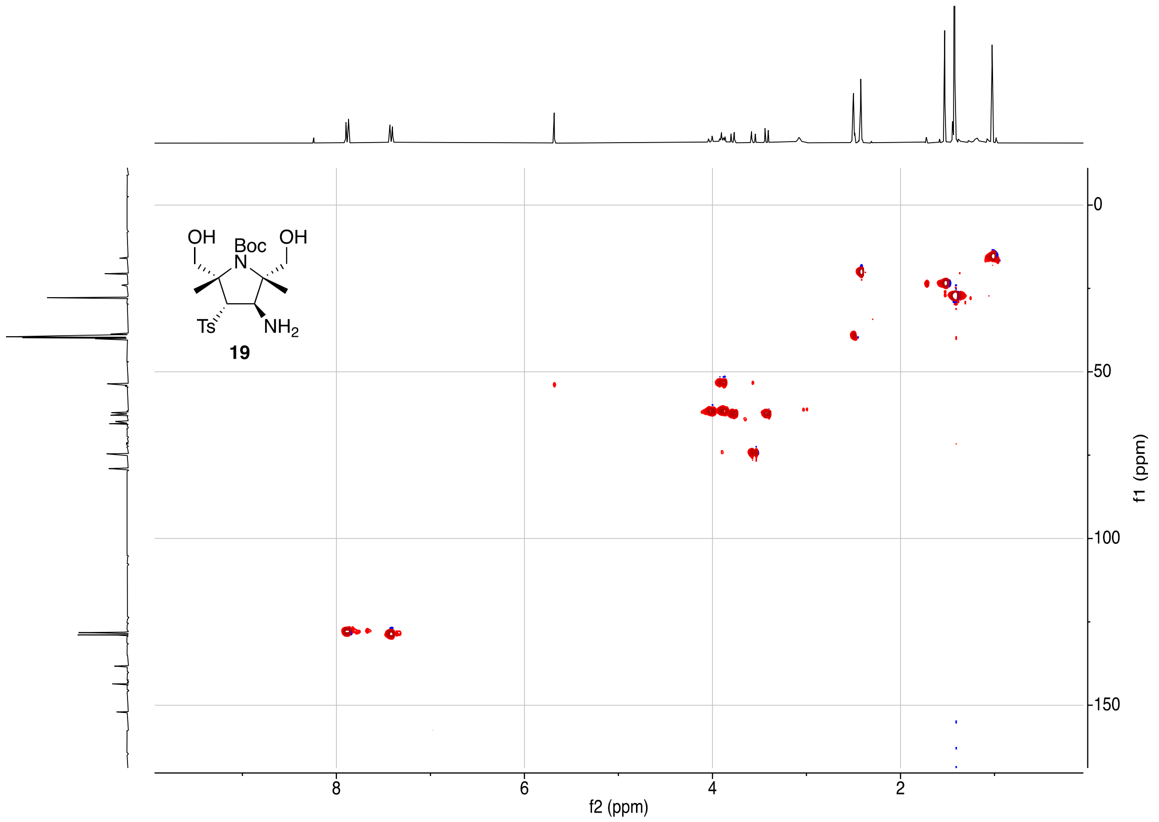

HSQC, in DMSO-d_6 of **19**.
